# One-Pot Synthesis of Aminodiperoxides from 1,5-Diketones, Geminal Bishydroperoxides and Ammonium Acetate

**DOI:** 10.3390/molecules30244703

**Published:** 2025-12-08

**Authors:** Yulia Yu. Belyakova, Viktoria E. Tsykunova, Peter S. Radulov, Lilya U. Dzhemileva, Roman A. Novikov, Alexey I. Ilovaisky, Ivan A. Yaremenko, Alexander O. Terent’ev

**Affiliations:** 1 N. D. Zelinsky Institute of Organic Chemistry, Russian Academy of Sciences, 47 Leninsky Prosp., 119991 Moscow, Russia; 2 All-Russian Research Institute for Phytopathology, 5 Institute Street, Odintsovo District, 143050 B. Vyazyomy, Moscow Region, Russia

**Keywords:** aminodiperoxides, peroxides, cancer, cytotoxicity, fungicidal activity

## Abstract

Herein, we report an efficient one-pot synthesis of bridged aminodiperoxides via a three-component reaction of 1,5-diketones with geminal bishydroperoxides and ammonium acetate. The synthesized aminodiperoxides are stable despite containing an unprotected secondary NH-group adjacent to two peroxide functionalities. Under optimal conditions, the reaction affords aminodiperoxides in high yields (up to 88%) with outstanding selectivity and high atom economy, thereby eliminating the need for column chromatographic purification. The synthesized aminodiperoxides exhibit potent cytotoxicity and remarkable selectivity toward Jurkat, K562, and A549 cancer cell lines, and are significantly superior to the clinically used anticancer agent camptothecin. Among all tested compounds, **3ec** is the most promising candidate, exhibiting high activity and selectivity toward all tested cell lines (Jurkat: CC_50_ = 12.9 µM, SI = 67.09; K562: CC_50_ = 19.6 µM, SI = 44.28; A549: CC_50_ = 48.2 µM, SI = 17.98). Furthermore, a novel class of fungicidal compounds has been discovered. The aminodiperoxides exhibit fungicidal activity against phytopathogenic fungi, in some cases comparable to the commercial fungicide Triadimefon.

## 1. Introduction

**Figure 1:**
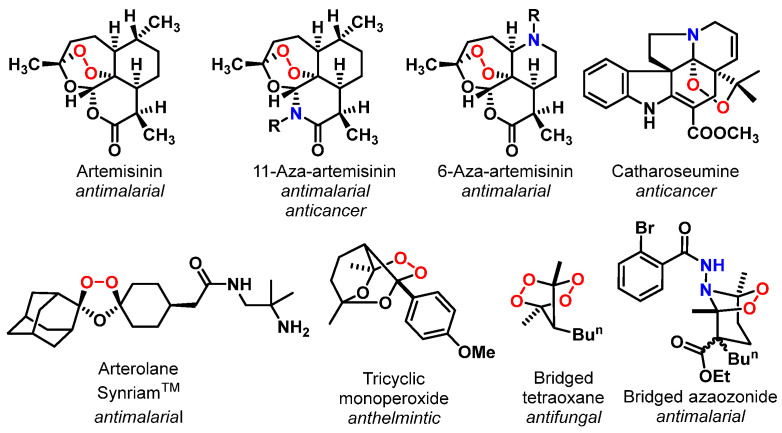
Bioactive cyclic organic peroxides.

Cyclic peroxides represent a structurally unique class of organic compounds with notable biological activities. Natural peroxide artemisinin and its semi-synthetic derivatives, as well as the synthetic ozonide arterolane exhibit high antimalarial activity [1,2] and are used as active components in modern antimalarial medicines. Moreover, synthetic peroxides demonstrate anthelmintic [3], antileishmanial [4], antimalarial [5], anticancer [6,7], antitubercular [8,9], fungicidal [10], and antiviral [11,12,13,14,15] activities. Progress in medicinal peroxide chemistry drives the development of novel types of peroxides. Thus, methods have been developed for the synthesis of stable bioactive cyclic peroxides, including 1,2-dioxolanes [16,17], 1,2,4-trioxolanes (ozonides) [18,19,20,21], 1,2,4,5-tetraoxanes [22,23]. In recent years, nitrogen-containing organic peroxides have attracted considerable attention. The incorporation of nitrogen into peroxide frameworks can substantially modify biological activity, primarily through the expansion of hydrogen-bonding interactions with biomolecules. For example, 11-aza-artemisinin and 6-aza-artemisinin (Figure 1) are more potent than artemisinin [2,24,25,26]. Recent studies have demonstrated that azaozonides exhibit antimalarial activity that is not observed in their parent ozonides [5]. Nevertheless, azaperoxides remain an unexplored area in both organic chemistry and medicinal chemistry.

**Scheme 1:**
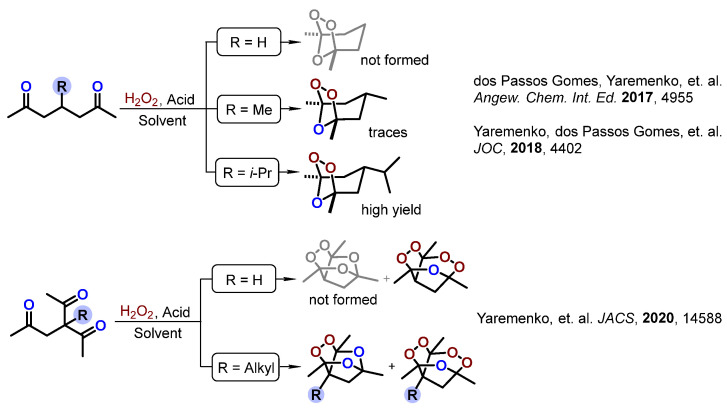
The non-obvious nature of peroxide assembly from diketones [20,21] and triketones [44].

The fundamental challenge in cyclic azaperoxide chemistry lies in their non-standard nature: the cycle contains two antagonistic fragments—an oxidizing O–O group and an oxidizable ‘NH’ or ‘NR’ group, while still avoiding self-oxidation. Although the first cyclic aminoperoxides were obtained more than 50 years ago, their synthetic methods remain limited. They include ozonolysis of alkenes [27] in the presence of primary amines; ozonolysis of vinyl ethers [28] in the presence of imines; ozonolysis of *O*-methylated dioximes [29,30,31]; cycloaminomethylation of H_2_O_2_ and hydroperoxides with 1,3,5-triaryl-1,3,5-triazinanes; O_2_ addition to iminium ion [32]; and the opening/recyclization of pentaoxa- and heptaoxaspiroalkanes in the presence of nitrogen source [33,34]. The most convenient starting substrates for the construction of azaperoxides are carbonyl compounds. However, even in this case, synthetic approaches remain limited. They are mainly based on the condensation of highly reactive ketones [35], strained alicyclic 1,5-diketones [36,37], acyclic 1,5-diketones [38,39], and triketones [40] with hydrogen peroxide and *N*-component. With regard to aminodiperoxides, it has been reported that Sm(NO_3_)_3_·6H_2_O-catalyzed three-component reaction of primary arylamines with geminal bishydroperoxides or 1,1′-peroxybis(1-hydroperoxycycloalkanes) and reactive pentane-1,5-dial affords bridged aminodiperoxides (bridged tetraoxazaspirocycloalkanes) [41,42]. These reactions require a catalyst, even though aldehydes are more reactive than ketones toward nucleophiles and can form peroxides more easily under mild conditions [43]. In the absence of a catalyst, peroxides either did not form or were formed in very low yields. Samarium probably coordinates the active species, thereby facilitating the assembly of the aminodiperoxide ring. In the case of the less reactive 1,5-diketone, the formation of an aminodiperoxide without the use of such a coordinating metal is not obvious. Moreover, even slight changes in the structure of the starting ketone can lead to unpredictable outcomes. Several examples of such sensitivity are shown in Scheme 1 [20,21,44].

It should be noted that the discovery of bioactive peroxides is challenging, too. The peroxide may either interact directly with the target or undergo a series of transformations to form products that subsequently act on the target. The O–O bond cleavage in the cyclic peroxide scaffold generates *O*-centred radicals that rapidly rearrange via β-scission to form *C*-centred radicals. This radical cascade underlies the powerful biological activity observed in peroxides [45]. This study addresses the above-mentioned fundamental challenges and reveals the potential of these unconventional compounds for medicinal chemistry.

**Figure 2:**
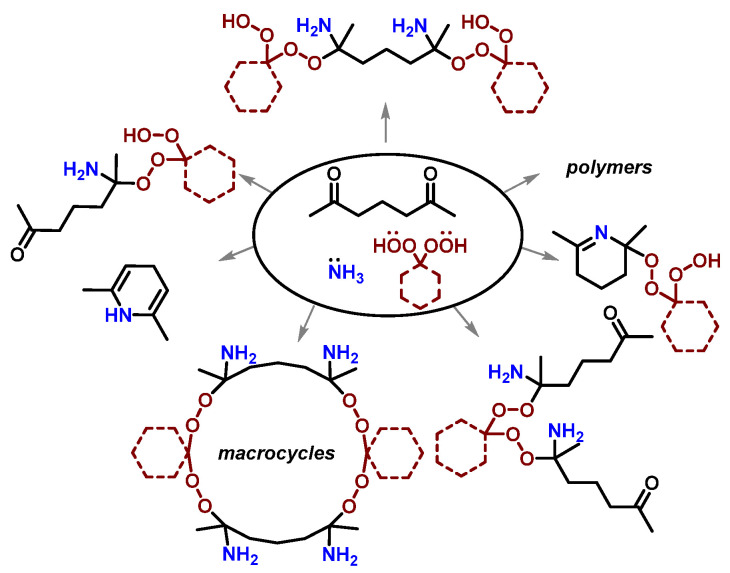
The complexity of the synthesis of aminodiperoxides from 1,5-diketones.

Herein, we investigated whether a selective three-component condensation of less reactive 1,5-diketones as carbonyl substrates, a geminal bishydroperoxides as the peroxide source, and NH-group source as an *N*-nucleophile could be achieved. This reaction system contains a carbonyl compound with two electrophilic centres and two different nucleophiles, including a geminal bishydroperoxide, which itself has two nucleophile sites. Such conditions are favourable for the formation of a complex mixture of products, including various dimeric and polymeric byproducts. The principal challenge of this transformation is the control of selectivity as illustrated by several number of possible products shown in Figure 2 [38,41,42]. 

**Scheme 2:**
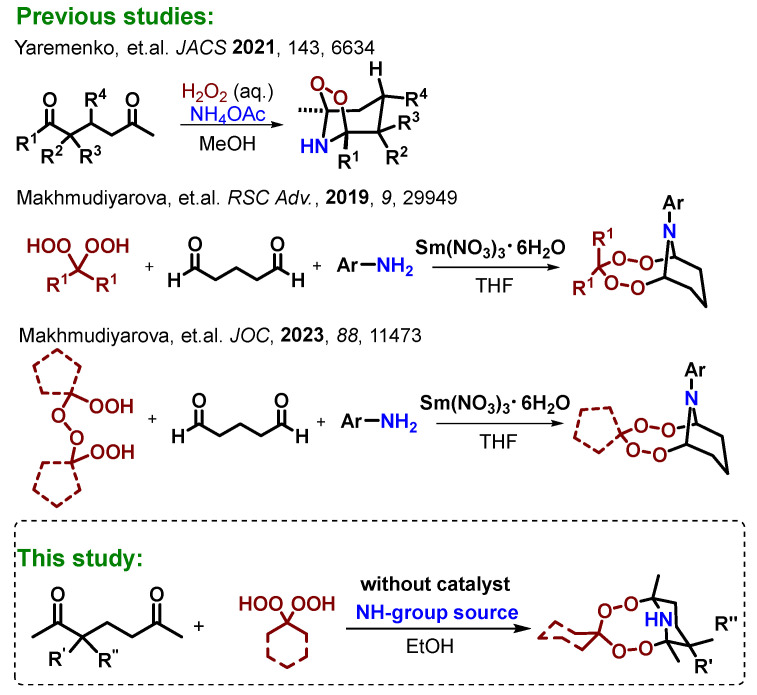
Synthesis of azadiperoxides [38,41,42].

In this work, the selective synthesis of bridged aminodiperoxides based on the three-component reaction of 1,5-diketones with geminal bishydroperoxides and NH-group source was successfully developed (Scheme 2).

It is surprising that a stable cycle formed with an unprotected secondary NH-group adjacent to two peroxide functionalities. The synthesized aminodiperoxides were tested against Jurkat, K562, and A549 cancer cell lines, and their fungicidal activity was evaluated against six phytopathogenic fungi.

## 2. Results and Discussion

### 2.1. Synthesis of the Aminodiperoxides

To establish the optimal conditions for the assembly of bridged aminodiperoxides, we selected the condensation of ethyl 2-acetyl-2-(4-chlorobenzyl)-5-oxohexanoate (**1l**) with 1,1-bishydroperoxycyclohexane (**2a**) and an NH-group source as a model reaction. The effect of the relative amounts of **2a** and NH-group source, the solvent, and the reaction time on the yield of aminodiperoxide product **3la** is shown in Table 1.

The initial procedure for the condensation of diketone **1l** with bishydroperoxide **2a** and an NH-group source was as follows: bishydroperoxide **2a** and the NH-group source (NH_3_(aq), NH_3_ in MeOH, NH_4_OAc, or HCOONH_4_) were added to a solution of diketone **1l** (0.200 g; 0.62 mmol) in MeOH, EtOH, or THF at room temperature. The reaction progress was monitored by TLC control. When aqueous ammonia was used as the NH-group source, aminodiperoxide **3la** was obtained in 38% yield (Table 1, entry 1), while using a methanolic ammonia, afforded it in 31% yield (Table 1, entry 2). Replacement of ammonia with ammonium acetate significantly increased the yield of **3la** (Table 1, entry 3). When THF used as the solvent, the complete conversion of the starting diketone **1l** was achieved within 4 h, affording product **3la** in 72% yield (Table 1, entry 4). Notably, when EtOH was used as the solvent, aminodiperoxide **3la** precipitated as a single diastereomer as white crystals without requiring further purification by column chromatography (Table 1, entry 5). The increasing of the reaction time from 1 h to 2 h and then to 24 h resulted in a significant increase in the yield of **3la** from 51% to 71% and 77%, respectively (Table 1, entries 6, 7). Surprisingly, when the reaction mixture was stirred at room temperature for 24 h and then kept at −22 °C overnight, the yield of aminodiperoxide **3la** increased by an additional 11%, reaching 88%. (Table 1, entry 8). In contrast, the use of ammonium formate afforded **3la** in 67% yield. (Table 1, entry 9). In all cases, no formation of aminodiperoxide **3la′** was observed.

Additionally, we performed ^1^H, ^13^C, ^15^N, and ^1^H-^15^N HMBC NMR monitoring of the discovered three-component reaction of 1,5-diketone **1l** with geminal bishydroperoxide **2a** and an NH-group source (NH_4_OAc) directly in an NMR tube, using EtOH-d_6_ as the solvent. The formation of the target aminodiperoxides **3la** + **3la′** proceeds rapidly after mixing the substrates and reagent. Within 10 minutes without stirring, the reaction was mostly complete (conversion of **1l** was ~80%), and after ~20–30 minutes, the ^1^H NMR signals of the starting diketone **1l** disappeared completely. Aminodiperoxides **3la** and **3la′** were formed as a mixture of two diastereomers in a ratio of **3la**:**3la′**~2.5:1. After two hours, the **3la**:**3la′** ratio changes only slightly, reaching ~3:1. Complete isomerization into the major diastereomer **3la** was observed after 20 h (see the Supplementary Materials).

**Scheme 3:**
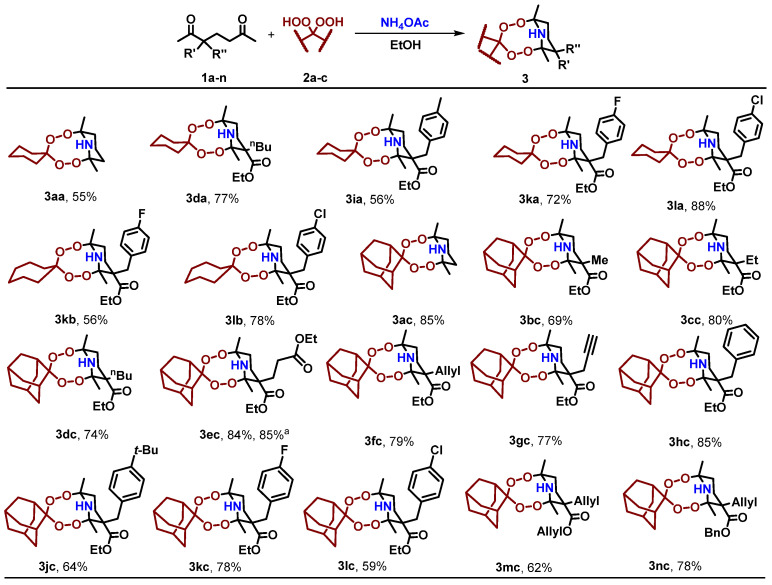
The scope of synthesized aminodiperoxides **3**. General procedure: NH_4_OAc (0.133–0.361 g, 1.73–4.68 mmol, 3 mol NH_4_OAc/1 mol of 1,5-diketone **1a**–**n**) and bishydroperoxide **2** (1.5 equiv) were successively added with stirring to a solution of 1,5-diketone **1** (0.200 g, 0.58–1.56 mmol, 1 equiv.) in EtOH (3 mL) at 20–25 °C. The reaction mixture was stirred at 20–25 °C for 24 h, then was kept at −22 °C overnight. ^a^ Scaled to 1.0 g of diketone **1e**.

With the optimal conditions in hand (Table 1, entry 8), we explored the scope and limitations of the aminodiperoxide assembly by entering diketones **1a**–**n** and bishydroperoxides **2a**–**c** into a three-component reaction with NH_4_OAc (Scheme 3). In all cases, aminodiperoxides were obtained as single diastereomers in high yields (up to 88% for aminodiperoxide **3la**). The scalability of the method was demonstrated by the synthesis of aminodiperoxide **3ec**, employing 1.0 g of diketone **1e** as the starting material, which afforded the product in 85% yield.

**Figure 3:**
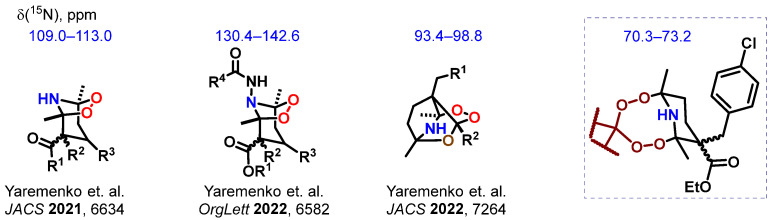
Summarized data of the ^15^N chemical shifts for various types of azaperoxides [38,39,40].

The structures of compounds **3** was confirmed by ^1^H and ^13^C NMR spectroscopy and HRMS data. In particular, for aminodiperoxide **3la**, the characteristic peaks in the ^1^H NMR spectrum appear as singlets at 1.41 and 1.62 ppm (s, 3H, CH_3_). In the ^13^C NMR spectrum, the characteristic signals are observed at 109.2 (OCO), and at 88.8, 94.1 ppm (CH_3_***C***NH). Additionally, 2D ^1^H–^15^N HMBC NMR experiments were performed for both diastereomers, and ^15^N chemical shifts were measured for this class of aminoperoxides (see the Supplementary Materials). For the major diastereomer **3la**, the ^15^N signal was observed at 70.3 ppm, whereas for the minor diastereomer **3la′** it appeared at 73.2 ppm. Interestingly, the ^15^N chemical shifts in the azamonoperoxides from our previous studies are significantly different and observed in the region typical for amides (Figure 3).

**Figure 4:**
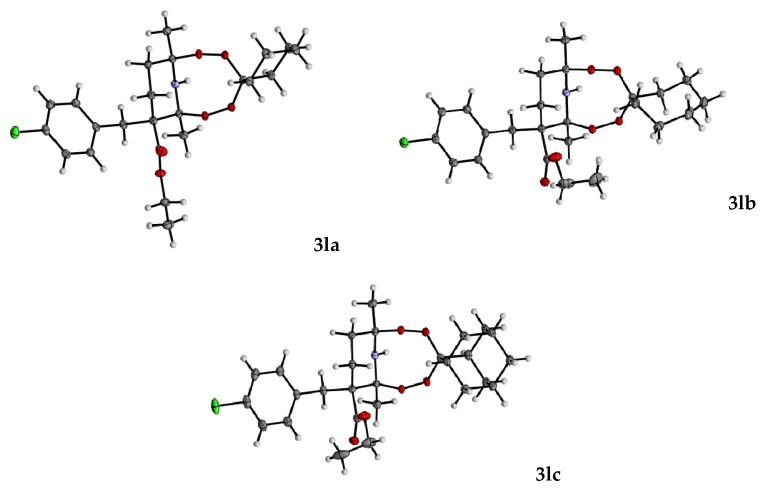
Molecular structures of **3la**, **3lb** and **3lc**. Atoms are presented as atomic displacement parameters (ADPs) ellipsoids (50% probability).

The stereochemical and NMR assignments were further confirmed by single-crystal X-ray analysis for **3la**, **3lb** and **3lc** (Figure 4).

**Scheme 4:**
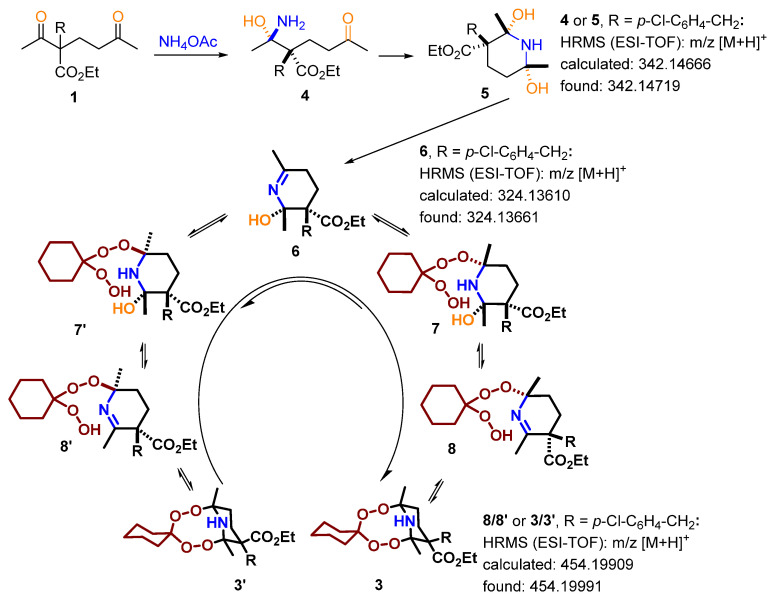
The proposed pathway for the assembly of aminodiperoxides **3** and **3′**.

Based on the literature reports on the synthesis of aminoperoxides from 1,5-diketones [38] and our experimental observations, we propose the following route for the assembly of aminodiperoxides (Scheme 4). The reaction begins with the nucleophilic addition of ammonia to one of the carbonyl groups of the starting 1,5-diketone **1**, forming intermediate **4**, followed by an intramolecular nucleophilic attack of the NH_2_ group on the second carbonyl group, yielding intermediate **5**. The reaction then proceeds through a series of elimination and addition steps: first, a water molecule is eliminated from **5** upon protonation of the OH-group, yielding imine **6**, which undergoes nucleophilic addition of the geminal bishydroperoxide **2** from one of two sides. To complete the formation of the peroxide ring, intermediate **7** or **7′** undergoes elimination of the water to form imines **8** or **8′**, respectively. Subsequent intramolecular nucleophilic attack by the OOH group on the imine carbon then affords aminodiperoxide **3** or **3′**. The minor diastereomer **3′** slowly transforms into the major diastereomer **3**. Since the formation of aminodiperoxides **3** proceeds very rapidly, it was not possible to detect the key intermediates by NMR spectroscopy. However, in the synthesis of aminodiperoxide **3la** (R = *p*-Cl-C_6_H_4_-CH_2_), high-resolution mass spectrometry enabled the detection of intermediates **4** or **5**, **6**, as well as **8**/**8′** or products **3**/**3′** (compounds **4** and **5**, **8**, **8′**, **3**, and **3′** have identical molecular formulas).

Since cyclic peroxides exhibit a wide range of biological activities, in the next step of this study, we investigated the synthesized aminodiperoxides **3** for their anticancer activity against Jurkat, K562, and A549 cancer cell lines, as well as their fungicidal activity against phytopathogenic fungi.

### 2.2. In Vitro Cytotoxicity of the Aminodiperoxides

The cytotoxicity of aminodiperoxides was evaluated against Jurkat (T-lymphoblastic leukemia) and K562 (chronic myeloid leukemia) oncohematologic cell lines, A549 non-small lung cancer cells, and HEK293 normal epithelial cells using flow cytometry. The results were compared with those for natural peroxide artemisinin, and the widely used cytostatic agent camptothecin (Table 2).

The obtained results indicate that the cytotoxicity of aminodiperoxides depends significantly on their structure. Compound **3ec**, bearing two ethoxycarbonyl groups and an adamantyl substituent, showed the highest activity and selectivity against all three cancer cells under investigation. Pronounced activity against Jurkat and K562 cell lines was also observed for aminodiperoxides **3fc**, **3mc**, and **3nc**, which contain allyl and adamantyl fragments. Interestingly, compound **3hc** bearing an unsubstituted benzyl moiety, and compound **3jc** with a *tert*-butyl substituent on the phenyl ring, demonstrated good activity and selectivity towards Jurkat and K562 cell lines, whereas the cytotoxicity of the halogen-substituted analogues **3kc** and **3lc** was significantly lower. Although aminodiperoxide **3ka** showed the best cytotoxicity (CC_50_ = 11.9 μM), it exhibited only moderate selectivity towards Jurkat cells and very low selectivity towards A549 non-small lung cancer cells. Overall, the cytotoxicity and selectivity of aminodiperoxides against A549 cells are comparable to those of artemisinin, while the cytotoxic activity and selectivity of the lead compounds towards Jurkat and K562 cell lines significantly exceed that of artemisinin. In all cases, aminodiperoxides were superior to camptothecin in both cytotoxicity and selectivity.

Aminodiperoxides **3ec**, **3fc**, **3hc**, **3jc**, **3mc**, and **3nc**, which were the most active and selective toward oncohematological cell lines, were further evaluated using real-time cell analysis (RTCA). RTCA assays allow for the monitoring of diverse cellular processes, including cell adhesion, cell morphology, receptor-mediated signalling, and cell proliferation. This method is now widely used to monitor the compound-mediated cytotoxicity [46,47].

A parameter termed cell index (CI) was derived [48] to represent cell status based on the measured electrical impedance. Cell death or toxicity-induced cell detachment, or cell rounding leads to a decrease in CI [46,47,48].

**Figure 5:**
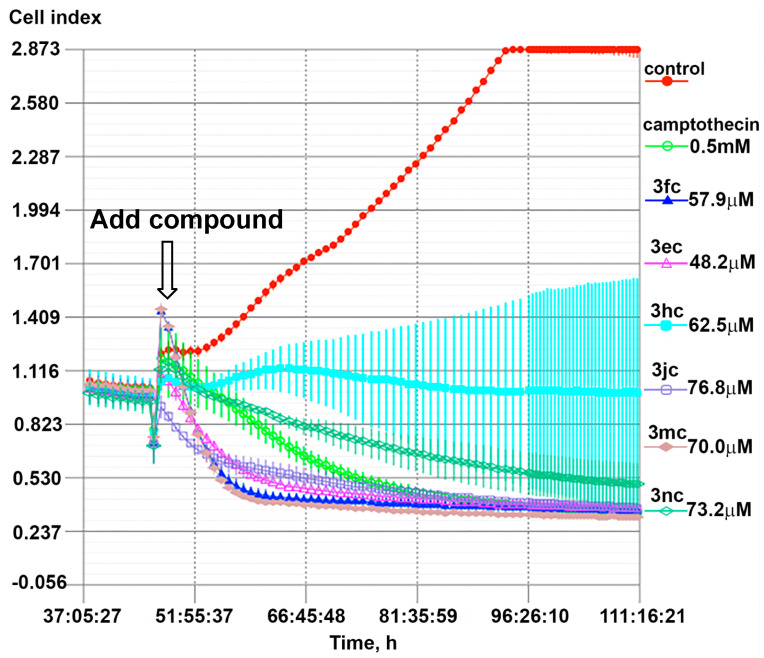
Real-time xCELLigence Cell Index Assay after treatment of A549 cells with test compounds. Each curve is an average of two wells ± SD. The panel is normalized to the time of compound addition. The control sample is highlighted in red.

In this study, we monitored changes in A549 cells’ adhesion to collagen type IV-treated substrates following treatment with aminodiperoxides **3ec**, **3fc**, **3hc**, **3jc**, **3mc**, and **3nc** (Figure 5). Camptothecin, a classical cytostatic, was used as a positive control due to its antitumor activity against a wide range of cell lines [49].

Analysis of the curves of the dependence of CI on the cultivation time of the A549 cell culture revealed a significant decrease in CI after the addition of aminodiperoxides. The observed uniform decrease in CI during incubation after the addition of both aminodiperoxides and camptothecin indicates the pronounced cytotoxicity of aminodiperoxides, especially compounds **3ec**, **3fc**, **3jc**, **3mc**, and **3nc**. These results confirm the antiproliferative potential of aminodiperoxides, but further investigation of the mechanism of cell death is necessary.

### 2.3. In Vitro Fungicidal Activity of the Aminodiperoxides

The aminodiperoxides **3** were tested against plant pathogenic fungi of various taxonomic classes that cause significant damage to agriculture and crop production—*Venturia inaequalis (V.i.)*; *Rhizoctonia solani (R.s.)*, *Fusarium oxysporum (F.o.)*, *Fusarium moniliforme (F.m.)*, *Bipolaris sorokiniana (B.s.)*, *Sclerotinia sclerotiorum (S.s.)*. The effect of the tested aminodiperoxides on the mycelium radial growth in the potato-saccharose agar was measured at the concentration of 30 mg/L. Triadimefon was used as a reference compound (Table 3).

Aminodiperoxide **3ia** was found to be most active against *V.i.* and showed activity comparable to that of the commercial fungicide Triadimefon. Aminodiperoxides **3ia** and **3gc** also demonstrated activity against *R.s.* comparable to Triadimefon. Compounds **3kb** and **3lb** showed slightly higher activity than Triadimefon against *B.s.* These results identify aminodiperoxides as a new class of fungicidal compounds. The preliminary findings will serve as the basis for more detailed future studies.

## 3. Materials and Methods

Caution: Although we have encountered no difficulties in working with the peroxides described below, the proper precautions, such as the use of shields, fume hoods, and the avoidance of transition metal salts, heating, and shaking, should be taken whenever possible.

NMR spectra were recorded on a commercial instrument (Bruker, Daltonic, Germany, 300.13 MHz for ^1^H, 75.48 MHz for ^13^C) in CDCl_3._ High resolution mass spectra (HRMS) were measured using electrospray ionization (ESI) (Bruker, Daltonic, Germany). The measurements were performed in a positive ion mode (interface capillary voltage 4500 V); the mass ratio was from *m*/*z* 50 to 3000 Da; external/internal calibration was performed with Electrospray Calibrant Solution. A syringe injection was used for solutions in MeCN (flow rate 3 µL/min). Nitrogen was applied as a dry gas; interface temperature was set at 180 °C. Elemental analysis (C, H, N) was carried out using a CHN analyzer (Leco TruSpec Micro, MI, USA), and calculated values confirm a purity of >95% for all biologically tested compounds. The TLC analysis was carried out on silica gel chromatography plates Alugram UV254 (Macherey-Nagel, Duren, Germany); Sorbent: Silica 60, specific surface (BET) ~500 m^2^/g, mean pore size 60 Å, specific pore volume 0.75 mL/g, particle size 5–17 µm; Binder: highly polymeric product, which is stable in almost all organic solvents and resistant towards aggressive visualization reagents. The melting points were determined on a Kofler hot-stage apparatus. Chromatography of triketones was performed on silica gel (0.060–0.200 mm, 60 A, CAS 7631-86-9 (Acros Organics, Geel, Belgium). Chromatography of peroxides was performed on silica gel (0.040–0.060 mm, 60 A, CAS 7631-86-9 (Acros Organics, Geel, Belgium).

2-Adamantanone, cyclohexanone, cycloheptanone, heptane-2,6-dione, ethyl acetoacetate, tert-butyl acetoacetate, allyl alcohol, benzyl alcohol, methyl vinyl ketone, CeCl_3_·7H_2_O, benzyl and alkyl halides, were purchased from Acros Organics, Geel, Belgium. Ammonium acetate (NH_4_OAc), ethanol (EtOH), ethyl acetate (EA), petroleum ether (PE) (40/70), methanol (MeOH), tetrahydrofuran (THF), H_2_O_2_ (35% aqueous solution), NH_4_OAc, HCOONH_4_, NH_3(aq)_, were purchased from commercial suppliers.

### 3.1. Synthesis of 1,5-Diketones ***1a**–**n***

1,5-Diketones **1a**–**l** [10,20,21] and **1m,n** were synthesized according to a known procedures. 1,5-diketones **1a**–**n** are known compounds.

### 3.2. Synthesis of Bishydroperoxides

**2a**–**c** Bishydroperoxides **2a**–**c** are known compounds. Bishydroperoxides **2a**–**c** were synthesized according to a known procedures [50].

### 3.3. Procedure for the Synthesis of Aminodiperoxide ***3la*** with Use NH_3_, Ammonium Salts and 1,1-Dihydroperoxycyclohexane ***2a*** (Table 1, Entries 1–9)

A 22% aq. solution of NH_3_ (0.26 mL, 3.08 mmol, 5 mol NH_3_/1 mol of **1l**) (entry 1), 7M methanolic solution of NH_3_ (0.44 mL, 3.08 mmol, 5 mol NH_3_/1 mol of **1l**) (entry 2), NH_4_OAc (0.142–0.237 g, 1.85–3.08 mmol, 3.0–5.0 mol NH_4_OAc/1 mol of **1l**) (entries 3–8) or HCOONH_4_ (0.117 g, 3.08 mmol, 3.0 mol HCOONH_4_/1 mol of **1l**) (entry 9) and 1,1-dihydroperoxycyclohexane **2a** (0.137 g, 0.92 mmol, 1.5 mol **2a**/1.0 mol of **1l**) were successively added with stirring to a solution of 1,5-diketone **1l** (0.200 g, 0.62 mmol) in MeOH (2–3 mL) (entries 1–3), THF (3 mL) (entry 4), EtOH (3 mL) (entries 5–9) at 20–25 °C.

For entries **1**–**7**, the reaction mixture was stirred at 20–25 °C for 0.5–24 h. Then CHCl_3_ (30 mL) and water (10 mL) were added. The organic layer was separated, and the aqueous layer was extracted with CHCl_3_ (3 × 30 mL). The combined organic phases were dried over MgSO_4_, and the solvent was removed in a vacuum of a water jet pump. Aminodiperoxide **3la** was isolated by chromatography on SiO_2_ using PE:EA mixture as the eluent with a gradient of ethyl acetate (EA) from 5 to 9 vol. % (entries 1–4). In entries 5–7, the precipitated crystals were filtered and washed with a cold EtOH/H_2_O mixture (40:60, *v*/*v*). Pure aminodiperoxide **3la** was obtained.

In the case of entries **8**, **9** the reaction mixture was stirred at 20–25 °C for 24 h, then was kept at −22 °C overnight. The precipitated crystals were filtered and washed with a cold EtOH/H_2_O mixture (40:60, *v*/*v*). Pure aminodiperoxide **3la** was obtained.

### 3.4. General Procedure for the Synthesis of Aminodiperoxides ***3aa***, ***3da***, ***3ia***, ***3ka***, ***3la***, ***3kb***, ***3lb***, ***3ac***–***3hc***, ***3jc***–***3nc*** from 1,5-Diketones ***1a**–**n*** and Corresponding Bishydroperoxides ***2a**–**c***

Ammonium acetate (0.133–0.361 g, 1.73–4.68 mmol, 3 mol NH_4_OAc/1 mol of 1,5-diketone **1a**–**n**) and corresponding 1,1-dihydroperoxycyclohexane **2a** (0.137–0.347 g, 0.92–2.34 mmol, 1.5 mol **2a**/1.0 mol of **1a,d,i,k,l**), 1,1-dihydroperoxycycloheptane **2b** (0.150–0.158 g, 0.92–0.97 mmol, 1.5 mol **2b**/1.0 mol of **1k,l**) or 2,2-dihydroperoxyadamantane **2c** (0.173–0.469 g, 0.87–2.34 mmol, 1.5 mol **2c**/1.0 mol of **1a**–**h**,**j**–**n**) were successively added with stirring to a solution of 1,5-diketone **1a**–**n** (0.200 g, 0.58–1.56 mmol) in EtOH (3 mL) at 20–25 °C. The reaction mixture was stirred at 20–25 °C for 24 h. Then the reaction mixture was kept at −22 °C overnight. In the case of **3aa**, **3ia**, **3ka**, **3la**, **3kb**, **3lb**, **3ac**–**3hc**, **3jc**–**3nc**: the precipitated crystals were filtered and washed with cold EtOH/H_2_O mixture (40:60, *v*/*v*). Pure aminodiperoxides **3aa**, **3ia**, **3ka**, **3la**, **3kb**, **3lb**, **3ac**–**3hc**, **3jc**–**3nc** were obtained.

In the case of **3da**, then CHCl_3_ (30 mL) and water (10 mL) were added. The organic layer was separated, and the aqueous layer was extracted with CHCl_3_ (3 × 30 mL). The combined organic phases were dried over MgSO_4_, and the solvent was removed in a vacuum of a water jet pump. Aminodiperoxide **3da** was isolated by chromatography on SiO_2_ using PE:EA (30:1) mixture as the eluent.

**Compounds**: **3aa**: 0.220 g, 0.85 mmol, yield 55%; **3da**: 0.233 g, 0.60 mmol, yield 77%; **3ia**: 0.159 g, 0.37 mmol, yield 56%; **3ka**: 0.204 g, 0.47 mmol, yield 72%; **3la**: 0.275 g, 0.54 mmol, yield 88%; **3kb**: 0.165 g, 0.37 mmol, yield 56%; **3lb**: 0.227 g, 0.49 mmol, yield 78%; **3ac**: 0.410 g, 1.32 mmol, yield 85%; **3bc**: 0.256 g, 0.65 mmol, yield 69%; **3cc**: 0.287 g, 0.70 mmol, yield 80%; **3dc**: 0.254 g, 0.58 mmol, yield 74%; **3ec**: 0.224 g, 0.47 mmol, yield 84%; **3fc**: 0.277 g, 0.66 mmol, yield 79%; **3gc**: 0.271 g, 0.65 mmol, yield 77%; **3hc**: 0.276 g, 0.59 mmol, yield 85%; **3jc**: 0.196 g, 0.37 mmol, yield 64%; **3kc**: 0.250 g, 0.51 mmol, yield 78%; **3lc**: 0.184 g, 0.36 mmol, yield 59%; **3mc**: 0.212 g, 0.49 mmol, yield 62%; **3nc**: 0.250 g, 0.52 mmol, yield 78%.

### 3.5. Synthesis of Aminodiperoxide ***3ec*** Scaled Up to 1.0 g of 1,5-Diketone ***1e***

Ammonium acetate (0.770 g, 9.99 mmol, 3 mol NH_4_OAc/1 mol of 1,5-diketone **1e**) and 2,2-dihydroperoxyadamantane **2c** (1.0 g, 4.99 mmol, 1.5 mol **2c**/1.0 mol of **1e**) were successively added with stirring to a solution of 1,5-diketone **1e** (1.0 g, 3.33 mmol) in EtOH (6 mL) at 20–25 °C. The reaction mixture was stirred at 20–25 °C for 24 h. Then the reaction mixture was kept at −22 °C overnight. The precipitated crystals were filtered and washed with a cold EtOH/H_2_O mixture (40:60, *v*/*v*). Pure aminodiperoxide **3ec** was obtained (1.37 g, 2.84 mmol, yield 85%).

(1R*,7S*)-1,7-Dimethyl-2,3,5,6-tetraoxa-11-azaspiro[bicyclo [5.3.1]undecane-4,1′-cyclohexane], **3aa**. White crystals. Mp = 46–48 °C. R_f_ = 0.41 (TLC, PE:EA, 10:1). ^1^H NMR (300.13 MHz, CDCl_3_), δ: 3.63 (br. s, 1H), 2.22–2.13 (m, 1H), 2.04–1.84 (m, 2H), 1.77–1.66 (m, 2H), 1.61–1.38 (m, 11H), 1.34 (s, 6H). ^13^C NMR (75.48 MHz, CDCl_3_), δ: 108.6, 89.6, 31.9, 31.1, 29.9, 26.8, 25.5, 22.9, 21.9, 16.8. Anal. Calcd for C_13_H_23_NO_4_: C, 60.68; H, 9.01; N, 5.44. Found: C, 60.79; H, 9.13; N, 5.55. HRMS (ESI-TOF): *m*/*z* [M + H]^+^: calculated for [C_13_H_24_NO_4_]^+^: 258.1700; found: 258.1691.
Ethyl (1R*,7S*,8S*)-8-butyl-1,7-dimethyl-2,3,5,6-tetraoxa-11-azaspiro[bicyclo [5.3.1]undecane-4,1′-cyclohexane]-8-carboxylate, **3da**. Colourless oil. R_f_ = 0.53 (TLC, PE:EA, 10:1). ^1^H NMR (300.13 MHz, CDCl_3_), δ: 4.26–4.14 (m, 1H), 4.13–4.00 (m, 1H), 3.41 (br. s, 1H), 2.52 (td, *J* = 14.5, 6.0 Hz, 1H), 2.28–2.06 (m, 2H), 1.88–1.73 (m, 3H), 1.70–1.10 (m, 23H), 0.88 (t, *J* = 7.1 Hz, 3H). ^13^C NMR (75.48 MHz, CDCl_3_), δ: 173.5, 109.2, 94.1, 88.9, 60.7, 50.3, 31.2, 30.4, 30.0, 28.4, 26.7, 25.6, 23.2, 23.0, 21.9, 21.3, 20.8, 14.3, 14.1. Anal. Calcd for C_20_H_35_NO_6_: C, 62.31; H, 9.15; N, 3.63. Found: C, 62.48; H, 9.28; N, 3.82. HRMS (ESI-TOF): *m*/*z* [M +H]^+^: calculated for [C_20_H_36_NO_6_]^+^: 386.2537; found: 386.2527.
Ethyl (1R*,7S*,8R*)-1,7-dimethyl-8-(4-methylbenzyl)-2,3,5,6-tetraoxa-11-azaspiro[bicyclo [5.3.1]undecane-4,1′-cyclohexane]-8-carboxylate, **3ia**. White crystals. Mp = 95–96 °C. R_f_ = 0.58 (TLC, PE:EA, 10:1). ^1^H NMR (300.13 MHz, CDCl_3_), δ: 7.05 (d, *J* = 7.9 Hz, 2H), 6.97 (d, *J* = 7.9 Hz, 2H), 4.38–4.16 (m, 1H), 4.15–4.02 (m, 1H), 3.45 (br. s, 1H), 3.40 (d, *J* = 13.6 Hz, 1H), 3.01 (d, *J* = 13.6 Hz, 1H), 2.41 (td, *J* = 14.4, 4.3 Hz, 1H), 2.30 (s, 3H), 2.23–2.02 (m, 2H), 1.81 (td, *J* = 14.4, 4.3 Hz, 1H), 1.69–1.35 (m, 16H), 1.24 (t, *J* = 7.1 Hz, 3H). ^13^C NMR (75.48 MHz, CDCl_3_), δ: 173.0, 136.3, 134.1, 129.7, 129.2, 109.2, 94.2, 88.8, 60.8, 51.2, 36.4, 31.2, 30.0, 28.7, 26.7, 25.6, 23.0, 21.9, 21.4, 21.1, 20.6, 14.2. Anal. Calcd for C_24_H_35_NO_6_: C, 66.49; H, 8.14; N, 3.23. Found: C, 66.60; H, 8.22; N, 3.31. HRMS (ESI-TOF): *m*/*z* [M + H]^+^: calculated for [C_24_H_36_NO_6_]^+^: 434.2537; found: 434.2532.
Ethyl (1R*,7S*,8R*)-8-(4-fluorobenzyl)-1,7-dimethyl-2,3,5,6-tetraoxa-11-azaspiro[bicyclo [5.3.1]undecane-4,1′-cyclohexane]-8-carboxylate, **3ka**. White crystals. Mp = 134–135 °C. R_f_ = 0.50 (TLC, PE:EA, 10:1). ^1^H NMR (300.13 MHz, CDCl_3_), δ: 7.10–7.00 (m, 2H), 6.98–6.88 (m, 2H), 4.29–4.15 (m, 1H), 4.14–4.00 (m, 1H), 3.48 (br. s, 1H), 3.39 (d, *J* = 13.7 Hz, 1H), 3.03 (d, *J* = 13.7 Hz, 1H), 2.43 (td, *J* = 14.3, 4.3 Hz, 1H), 2.23–2.07 (m, 2H), 1.78 (td, *J* = 14.3, 4.4 Hz, 1H), 1.68–1.37 (m, 16H), 1.22 (t, *J* = 7.1 Hz, 3H). ^13^C NMR (75.48 MHz, CDCl_3_), δ: 172.7, 161.8 (d, ^1^*J*_CF_ = 244.3 Hz), 132.7 (d, ^4^*J*_CF_ = 3.4 Hz), 131.2 (d, ^3^*J*_CF_ = 7.7 Hz), 115.2 (d, ^2^*J*_CF_ = 21.0 Hz), 109.2, 94.1, 88.8, 61.0, 51.2, 35.9, 31.2, 30.0, 28.7, 26.7, 25.6, 22.9, 21.9, 21.3, 20.5, 14.2. Anal. Calcd for C_23_H_32_FNO_6_: C, 63.14; H, 7.37; F, 4.34; N, 3.20. Found: C, 63.28; H, 7.45; F, 4.41; N, 3.30. HRMS (ESI-TOF): *m*/*z* [M + H]^+^: calculated for [C_23_H_33_FNO_6_]^+^: 438.2286; found: 438.2298.
Ethyl (1R*,7S*,8R*)-8-(4-chlorobenzyl)-1,7-dimethyl-2,3,5,6-tetraoxa-11-azaspiro[bicyclo [5.3.1]undecane-4,1′-cyclohexane]-8-carboxylate, **3la**. White crystals. Mp = 131–132 °C. R_f_ = 0.37 (TLC, PE:EA, 10:1). ^1^H NMR (300.13 MHz, CDCl_3_), δ: 7.21 (d, *J* = 8.4 Hz, 2H), 7.02 (d, *J* = 8.4 Hz, 2H), 4.28–4.15 (m, 1H), 4.13–4.01 (m, 1H), 3.47 (br. s, 1H), 3.39 (d, *J* = 13.6 Hz, 1H), 3.02 (d, *J* = 13.6 Hz, 1H), 2.44 (td, *J* = 14.3, 4.3 Hz, 1H), 2.24–2.08 (m, 2H), 1.76 (td, *J* = 14.3, 4.3 Hz, 1H), 1.68–1.33 (m, 16H), 1.22 (t, *J* = 7.1 Hz, 3H). ^13^C NMR (75.48 MHz, CDCl_3_), δ: 172.8, 135.8, 132.7, 131.2, 128.6, 109.2, 94.1, 88.8, 61.0, 51.2, 36.1, 31.2, 30.0, 28.7, 26.7, 25.6, 22.9, 21.9, 21.3, 20.5, 14.2. Anal. Calcd for C_23_H_32_ClNO_6_: C, 60.85; H, 7.11; Cl, 7.81; N, 3.09. Found: C, 61.00; H, 7.28; Cl, 8.02; N, 3.19. HRMS (ESI-TOF): *m*/*z* [M + H]^+^: calculated for [C_23_H_33_ClNO_6_]^+^: 454.1991; found: 454.1991.
Ethyl (1R*,7S*,8R*)-8-(4-fluorobenzyl)-1,7-dimethyl-2,3,5,6-tetraoxa-11-azaspiro[bicyclo [5.3.1]undecane-4,1′-cycloheptane]-8-carboxylate, **3kb**. White crystals. Mp = 113–115 °C. R_f_ = 0.51 (TLC, PE:EA, 10:1). ^1^H NMR (300.13 MHz, CDCl_3_), δ: 7.09–7.00 (m, 2H), 6.97–6.87 (m, 2H), 4.26–4.02 (m, 2H), 3.49 (br. s, 1H), 3.38 (d, *J* = 13.7 Hz, 1H), 3.03 (d, *J* = 13.7 Hz, 1H), 2.41 (td, *J* = 14.2, 4.7 Hz, 1H), 2.34–2.23 (m, 2H), 1.77 (td, *J* = 14.2, 4.7 Hz, 1H), 1.59–1.38 (m, 18H), 1.22 (t, *J* = 7.1 Hz, 3H). ^13^C NMR (75.48 MHz, CDCl_3_), δ: 172.8, 161.9 (d,^1^*J*_CF_ = 245.0 Hz), 131.3 (d, ^4^*J*_CF_ = 3.4 Hz), 131.2 (d, ^3^*J*_CF_ = 7.7 Hz), 115.3 (d, ^2^*J*_CF_ = 21.0 Hz), 114.7, 94.1, 88.8, 61.0, 51.2, 35.9, 35.4, 31.5, 30.5, 30.3, 28.7, 26.6, 23.4, 22.4, 21.3, 20.4, 14.2. Anal. Calcd for C_24_H_34_FNO_6_: C, 63.84; H, 7.59; F, 4.21; N, 3.10. Found: C, 63.99; H, 7.65; F, 4.32; N, 3.19. HRMS (ESI-TOF): *m*/*z* [M + K]^+^: calculated for [C_24_H_34_FNKO_6_]^+^: 490.2002; found: 490.1989.
Ethyl (1R*,7S*,8R*)-8-(4-chlorobenzyl)-1,7-dimethyl-2,3,5,6-tetraoxa-11-azaspiro[bicyclo [5.3.1]undecane-4,1′-cycloheptane]-8-carboxylate, **3lb**. White crystals. Mp = 149–151 °C. R_f_ = 0.48 (TLC, PE:EA, 10:1). ^1^H NMR (300.13 MHz, CDCl_3_), δ: 7.21 (d, *J* = 8.1 Hz, 2H), 7.01 (d, *J* = 8.1 Hz, 2H), 4.28–4.01 (m, 2H), 3.48 (br. s, 1H), 3.39 (d, *J* = 13.6 Hz, 1H), 3.03 (d, *J* = 13.6 Hz, 1H), 2.42 (td, *J* = 14.4, 4.7 Hz, 1H), 2.35–2.24 (m, 2H), 1.84–1.36 (m, 19H), 1.23 (t, *J* = 7.1 Hz, 3H). ^13^C NMR (75.48 MHz, CDCl_3_), δ: 172.8, 135.7, 132.7, 131.2, 128.6, 114.7, 94.1, 88.7, 61.0, 51.2, 36.1, 35.4, 31.5, 30.5, 30.3, 28.7, 26.6, 23.4, 22.4, 21.3, 20.5, 14.2. Anal. Calcd for C_24_H_34_ClNO_6_: C, 61.60; H, 7.32; Cl, 7.57; N, 2.99. Found: C, 61.71; H, 7.42; Cl, 7.65; N, 3.09. HRMS (ESI-TOF): *m*/*z* [M + H]^+^: calculated for [C_24_H_35_ClNO_6_]^+^: 468.2147; found: 468.2136.
(1R*,7S*)-1,7-dimethyl-2,3,5,6-tetraoxa-11-azaspiro[bicyclo [5.3.1]undecane-4,1′-cyclohexane], **3ac**. White crystals. Mp = 123–125 °C. R_f_ = 0.64 (TLC, PE:EA, 10:1). ^1^H NMR (300.13 MHz, CDCl_3_), δ: 3.66 (br. s, 1H), 2.97–3.05 (m, 1H), 1.42–2.09 (m, 19H), 1.35 (s, 6H). ^13^C NMR (75.48 MHz, CDCl_3_), δ: 110.6, 89.5, 37.7, 34.6, 33.6, 33.3, 32.1, 30.8, 27.4, 27.1, 17.0. Anal. Calcd for C_17_H_27_NO_4_: C, 65.99; H, 8.80; N, 4.53. Found: C, 66.12; H, 8.98; N, 4.65. HRMS (ESI-TOF): *m*/*z* [M + H]^+^: calculated for [C_17_H_28_NO_4_]^+^: 310.2013; found: 310.2016.
Ethyl (1S*,1′R*,2R*,5R*,7′S*,8′S*)-1′,7′,8′-trimethyl-2′,3′,5′,6′-tetraoxa-11′-azaspiro[adamantane-2,4′-bicyclo [5.3.1]undecane]-8′-carboxylate, **3bc**. White crystals. Mp = 100–102 °C. R_f_ = 0.57 (TLC, PE:EA, 10:1). ^1^H NMR (300.13 MHz, CDCl_3_), δ: 4.26–3.98 (m, 2H), 3.42 (br. s, 1H), 3.07–2.96 (m, 1H), 2.77–2.58 (m, 1H), 2.06–1.93 (m, 4H), 1.86–1.71 (m, 4H), 1.70–1.51 (m, 8H), 1.47 (s, 3H), 1.40–1.29 (m, 6H), 1.24 (t, *J* = 7.1 Hz, 3H). ^13^C NMR (75.48 MHz, CDCl_3_), δ: 174.2, 111.1, 93.4, 88.7, 60.8, 46.6, 37.7, 34.6, 34.5, 33.6, 33.2, 33.0, 30.8, 28.6, 27.4, 26.7, 26.5, 21.6, 20.1, 14.2. Anal. Calcd for C_21_H_33_NO_6_: C, 63.78; H, 8.41; N, 3.54. Found: C, 63.92; H, 8.52; N, 3.63. HRMS (ESI-TOF): *m*/*z* [M + H]^+^: calculated for [C_21_H_34_NO_6_]^+^: 396.2381; found: 396.2379.
Ethyl (1S*,1′R*,2R*,5R*,7′S*,8′S*)-8′-ethyl-1′,7′-dimethyl-2′,3′,5′,6′-tetraoxa-11′-azaspiro[adamantane-2,4′-bicyclo [5.3.1]undecane]-8′-carboxylate, **3cc**. White crystals. Mp = 131–133 °C. R_f_ = 0.68 (TLC, PE:EA, 10:1). ^1^H NMR (300.13 MHz, CDCl_3_), δ:4.30–3.98 (m, 2H). 3.41 (br. s, 1H), 3.10–2.97 (m, 1H), 2.49 (td, *J* = 14.6, 5.8 Hz, 1H), 2.11–1.40 (m, 21H), 1.33 (s, 3H), 1.24 (t, *J* = 7.1 Hz, 3H), 0.74 (t, *J* = 7.5 Hz, 3H). ^13^C NMR (75.48 MHz, CDCl_3_), δ: 173.3, 111.0, 93.8, 88.6, 60.5, 50.7, 37.6, 34.5, 34.4, 33.5, 33.1, 32.9, 30.7, 28.2, 27.3, 26.6, 23.2, 21.1, 19.9, 14.2, 8.6. Anal. Calcd for C_22_H_35_NO_6_: C, 64.52; H, 8.61; N, 3.42. Found: C, 64.61; H, 8.73; N, 3.50. HRMS (ESI-TOF): *m*/*z* [M + H]^+^: calculated for [C_22_H_36_NO_6_]^+^: 410.2537; found: 410.2528.
Ethyl (1S*,1′R*,2R*,5R*,7′S*,8′S*)-8′-butyl-1′,7′-dimethyl-2′,3′,5′,6′-tetraoxa-11′-azaspiro[adamantane-2,4′-bicyclo [5.3.1]undecane]-8′-carboxylate, **3dc**. White crystals. Mp = 107–108 °C. R_f_ = 0.73 (TLC, PE:EA, 10:1). ^1^H NMR (300.13 MHz, CDCl_3_), δ: 4.28–4.13 (m, 1H), 4.11–3.98 (m, 1H), 3.41 (br. s, 1H), 3.11–2.92 (m, 1H), 2.77–2.58 (m, 1H), 2.06– 1.10 (m, 31H), 0.88 (t, *J* = 7.1 Hz, 3H). ^13^C NMR (75.48 MHz, CDCl_3_), δ: 173.4, 111.0, 93.8, 88.6, 60.5, 50.3, 37.6, 34.5, 34.4, 33.5, 33.1, 32.9, 30.7, 30.2, 28.4, 27.3, 26.6, 23.1, 21.1, 20.6, 14.2, 14.0. Anal. Calcd for C_24_H_39_NO_6_: C, 65.88; H, 8.98; N, 3.20. Found: C, 65.99; H, 9.11; N, 3.33. HRMS (ESI-TOF): *m*/*z* [M + H]^+^: calculated for [C_24_H_40_NO_6_]^+^: 438.2850; found: 438.2842.
Ethyl (1S*,1′R*,2R*,5R*,7′S*,8′R*)-8′-(3-ethoxy-3-oxopropyl)-1′,7′-dimethyl-2′,3′,5′,6′-tetraoxa-11′-azaspiro[adamantane-2,4′-bicyclo [5.3.1]undecane]-8′-carboxylate, **3ec**. White crystals. Mp = 107–109 °C. R_f_ = 0.37 (TLC, PE:EA, 10:1). ^1^H NMR (300.13 MHz, CDCl_3_), δ: 4.00–4.28 (m, 4H), 3.40 (br. s, 1H), 3.07–2.98 (m, 1H), 2.65–2.48 (m, 1H), 2.38–2.25 (m, 1H), 2.18 (td, *J* = 14.0, 4.6 Hz, 2H), 2.08–1.91 (m, 5H), 1.91–1.45 (m, 15H), 1.35 (s, 3H), 1.25 (t, *J* = 7.1 Hz, 3H), 1.24 (t, *J* = 7.1 Hz, 3H). ^13^C NMR (75.48 MHz, CDCl_3_), δ: 173.1, 172.7, 111.0, 93.5, 88.5, 60.8, 60.5, 49.8, 37.5, 34.4, 34.4, 33.5, 33.1, 32.9, 30.7, 29.6, 28.2, 27.3, 26.5, 25.6, 21.1, 20.7, 14.19, 14.15. Anal. Calcd for C_25_H_39_NO_8_: C, 62.35; H, 8.16; N, 2.91. Found: C, 62.49; H, 8.30; N, 3.05. HRMS (ESI-TOF): *m*/*z* [M + H]^+^: calculated for [C_25_H_40_NO_8_]^+^: 482.2748; found: 482.2749.
Ethyl (1S*,1′R*,2R*,5R*,7′S*,8′R*)-8′-allyl-1′,7′-dimethyl-2′,3′,5′,6′-tetraoxa-11′-azaspiro[adamantane-2,4′-bicyclo [5.3.1]undecane]-8′-carboxylate, **3fc**. White crystals. Mp = 108–109 °C. R_f_ = 0.48 (TLC, PE:EA, 10:1). ^1^H NMR (300.13 MHz, CDCl_3_), δ: 5.62–5.45 (m, 1H), 5.15–4.98 (m, 2H), 4.28–4.00 (m, 2H), 3.42 (br. s, 1H), 3.07–2.98 (m, 1H), 2.83–2.72 (m, 1H), 2.62–2.39 (m, 2H), 2.07–1.93 (m, 4H), 1.91–1.45 (m, 15H), 1.35 (s, 3H), 1.24 (t, *J* = 7.1 Hz, 3H). ^13^C NMR (75.48 MHz, CDCl_3_), δ: 172.8, 133.5, 118.3, 111.0, 93.3, 88.5, 60.7, 49.9, 37.5, 35.4, 34.5, 34.4, 33.5, 33.1, 32.9, 30.6, 28.0, 27.3, 26.6, 21.2, 21.0, 14.2. Anal. Calcd for C_23_H_35_NO_6_: C, 65.54; H, 8.37; N, 3.32. Found: C, 65.54; H, 8.37; N, 3.32. HRMS (ESI-TOF): *m*/*z* [M + H]^+^: calculated for [C_23_H_36_NO_6_]^+^: 422.2537; found: 422.2531.
Ethyl (1S*,1′R*,2R*,5R*,7′S*,8′R*)-1′,7′-dimethyl-8′-(prop-2-yn-1-yl)-2′,3′,5′,6′-tetraoxa-11′-azaspiro[adamantane-2,4′-bicyclo [5.3.1]undecane]-8′-carboxylate, **3gc**. White crystals. Mp = 134–135 °C. R_f_ = 0.63 (TLC, PE:EA, 10:1). ^1^H NMR (300.13 MHz, CDCl_3_), δ: 4.20–4.04 (m, 2H), 3.10 (br. s, 1H), 3.10–2.90 (m, 2H), 2.78–2.66 (m, 1H), 2.59 (td, *J* = 13.6, 5.6 Hz, 1H), 2.07–1.92 (m, 5H), 1.87–1.50 (m, 12H), 1.41 (s, 3H), 1.33 (s, 3H), 1.26 (t, *J* = 7.1 Hz, 3H). ^13^C NMR (75.48 MHz, CDCl_3_), δ: 171.9, 111.2, 92.7, 88.6, 80.1, 71.3, 61.2, 49.8, 37.6, 34.6, 34.5, 33.6, 33.2, 33.0, 30.8, 28.4, 27.4, 26.6, 22.1, 21.7, 21.1, 14.3. Anal. Calcd for C_23_H_33_NO_6_: C, 65.85; H, 7.93; N, 3.34. Found: C, 65.99; H, 8.11; N, 3.49. HRMS (ESI-TOF): *m*/*z* [M + H]^+^: calculated for [C_23_H_34_NO_6_]^+^: 420.2381; found: 420.2378.
Ethyl (1S*,1′R*,2R*,5R*,7′S*,8′R*)-8′-benzyl-1′,7′-dimethyl-2′,3′,5′,6′-tetraoxa-11′-azaspiro[adamantane-2,4′-bicyclo [5.3.1]undecane]-8′-carboxylate, **3hc**. White crystals. Mp = 163–164 °C. R_f_ = 0.69 (TLC, PE:EA, 10:1). ^1^H NMR (300.13 MHz, CDCl_3_), δ: 7.34–7.20 (m, 3H), 7.16–7.08 (m, 2H), 4.32–4.00 (m, 2H), 3.49 (br. s, 1H), 3.48 (d, *J* = 13.7 Hz, 1H), 3.17–2.98 (m, 2H), 2.44 (td, *J* = 14.3, 4.6 Hz, 1H), 2.10–1.96 (m, 4H), 1.91–1.52 (m, 15H), 1.45 (s, 3H), 1.25 (t, *J* = 7.1 Hz, 3H). ^13^C NMR (75.48 MHz, CDCl_3_), δ: 173.1, 137.4, 129.9, 128.4, 126.7, 111.1, 94.0, 88.7, 60.8, 51.3, 37.7, 36.8, 34.6, 34.5, 33.6, 33.2, 33.0, 30.8, 28.8, 27.4, 26.8, 21.3, 20.6, 14.2. Anal. Calcd for C_27_H_37_NO_6_: C, 68.77; H, 7.91; N, 2.97. Found: C, 68.88; H, 7.99; N, 3.09. HRMS (ESI-TOF): *m*/*z* [M + H]^+^: calculated for [C_27_H_38_NO_6_]^+^: 472.2694; found: 472.2674.
Ethyl (1S*,1′R*,2R*,5R*,7′S*,8′R*)-8′-(4-(tert-butyl)benzyl)-1′,7′-dimethyl-2′,3′,5′,6′-tetraoxa-11′-azaspiro[adamantane-2,4′-bicyclo [5.3.1]undecane]-8′-carboxylate, **3jc**. White crystals. Mp = 135–137 °C. R_f_ = 0.59 (TLC, PE:EA, 10:1). ^1^H NMR (300.13 MHz, CDCl_3_), δ: 7.24 (d, *J* = 8.4 Hz, 2H), 7.00 (d, *J* = 8.2 Hz, 2H), 4.29–4.16 (m, 1H), 4.14–4.02 (m, 1H), 3.38 (br. s, 1H), 3.41 (d, *J* = 13.7 Hz, 1H), 3.06–2.96 (m, 2H), 2.44 (td, *J* = 14.3, 4.6 Hz, 1H), 2.10–1.52 (m, 19H), 1.45 (s, 3H), 1.29 (s, 9H), 1.25 (t, *J* = 7.1 Hz, 3H). ^13^C NMR (75.48 MHz, CDCl_3_), δ: 173.1, 149.4, 134.2, 129.5, 125.3, 111.1, 94.0, 88.7, 60.8, 51.2, 37.7, 36.3, 34.6, 34.5, 33.6, 33.2, 33.0, 31.5, 30.8, 28.8, 27.4, 26.8, 21.4, 20.6, 14.2. Anal. Calcd for C_31_H_45_NO_6_: C, 70.56; H, 8.60; N, 2.65. Found: C, 70.71; H, 8.75; N, 2.79. HRMS (ESI-TOF): *m*/*z* [M + H]^+^: calculated for [C_31_H_46_NO_6_]^+^: 528.3320; found: 528.3316.
Ethyl (1S*,1′R*,2R*,5R*,7′S*,8′R*)-8′-(4-fluorobenzyl)-1′,7′-dimethyl-2′,3′,5′,6′-tetraoxa-11′-azaspiro[adamantane-2,4′-bicyclo [5.3.1]undecane]-8′-carboxylate, **3kc**. White crystals. Mp = 165–166 °C. R_f_ = 0.58 (TLC, PE:EA, 10:1). ^1^H NMR (300.13 MHz, CDCl_3_), δ: 7.10–7.00 (m, 2H), 6.98–6.87 (m, 2H), 4.31–4.14 (m, 1H), 4.11–3.96 (m, 1H), 3.51 (br. s, 1H), 3.40 (d, *J* = 13.7 Hz, 1H), 3.12–2.93 (m, 2H), 2.41 (td, *J* = 14.2, 4.7 Hz, 1H), 2.11–1.38 (m, 22H), 1.22 (t, *J* = 7.1 Hz, 3H). ^13^C NMR (75.48 MHz, CDCl_3_), δ: 172.8, 161.9 (d, ^1^*J*_CF_ = 245.0 Hz), 131.3 (d, ^4^*J*_CF_ = 3.4 Hz), 131.2 (d, ^3^*J*_CF_ = 7.7 Hz), 115.3 (d, ^2^*J*_CF_ = 21.0 Hz), 111.0, 93.8, 88.5, 60.7, 51.2, 37.5, 35.7, 34.5, 34.4, 33.5, 33.1, 32.9, 30.7, 28.6, 27.3, 26.6, 21.1, 20.3, 14.1. Anal. Calcd for C_27_H_36_FNO_6_: C, 66.24; H, 7.41; F, 3.88; N, 2.86. Found: C, 66.35; H, 7.52; F, 3.99; N, 2.98. HRMS (ESI-TOF): *m*/*z* [M + H]^+^: calculated for [C_27_H_37_FNO_6_]^+^: 490.2599; found: 490.2585.
Ethyl (1S*,1′R*,2R*,5R,7′S*,8′R*)-8′-(4-chlorobenzyl)-1′,7′-dimethyl-2′,3′,5′,6′-tetraoxa-11′-azaspiro[adamantane-2,4′-bicyclo [5.3.1]undecane]-8′-carboxylate, **3lc**. White crystals. Mp = 167–169 °C. R_f_ = 0.41 (TLC, PE:EA, 10:1). ^1^H NMR (300.13 MHz, CDCl_3_), δ: 7.21 (d, *J* = 8.3 Hz, 2H), 7.01 (d, *J* = 8.3 Hz, 2H), 4.30–3.95 (m, 2H), 3.48 (br. s, 1H), 3.41 (d, *J* = 13.3 Hz, 1H), 3.09–2.92 (m, 2H), 2.42 (td, *J* = 14.3, 4.7 Hz, 1H), 2.10–1.50 (m, 19H), 1.46–1.34 (m, 3H), 1.22 (t, *J* = 7.1 Hz, 3H). ^13^C NMR (75.48 MHz, CDCl_3_), δ: 172.9, 135.9, 132.6, 131.3, 128.6, 111.1, 93.9, 88.6, 60.9, 51.3, 37.6, 36.0, 34.6, 34.5, 33.6, 33.2, 33.0, 30.8, 28.7, 27.4, 26.7, 21.2, 20.5, 14.2. Anal. Calcd for C_27_H_36_ClNO_6_: C, 64.09; H, 7.17; Cl, 7.01; N, 2.77. Found: C, 64.21; H, 7.30; Cl, 7.19; N, 2.88. HRMS (ESI-TOF): *m*/*z* [M + H]^+^: calculated for [C_27_H_37_ClNO_6_]^+^: 506.2304; found: 506.2300.
Allyl (1S*,1′R*,2R*,5R*,7′S*,8′R*)-8′-allyl-1′,7′-dimethyl-2′,3′,5′,6′-tetraoxa-11′-azaspiro[adamantane-2,4′-bicyclo [5.3.1]undecane]-8′-carboxylate, **3mc**. White crystals. Mp = 79–81 °C. R_f_ = 0.66 (TLC, PE:EA, 10:1). ^1^H NMR (300.13 MHz, CDCl_3_), δ: 5.97–5.80 (m, 1H), 5.64–5.44 (m, 1H), 5.37–5.28 (m, 1H), 5.23–5.17 (m, 1H), 5.14–5.00 (m, 2H), 4.66 (dd, *J* = 13.5, 5.5 Hz, 1H), 4.52 (dd, *J* = 13.5, 5.5 Hz, 1H), 3.41 (br. s, 1H), 3.07–2.97 (m, 1H), 2.79 (dd, *J* = 14.0, 5.9 Hz, 1H), 2.64–2.42 (m, 2H), 2.07–1.45 (m, 16H), 1.50 (s, 3H), 1.45 (s, 3H). ^13^C NMR (75.48 MHz, CDCl_3_), δ: 172.6, 133.4, 132.4, 118.6, 117.8, 111.1, 93.5, 88.7, 65.4, 50.3, 37.7, 35.6, 34.6, 34.5, 33.6, 33.2, 33.0, 30.8, 28.1, 27.4, 26.7, 21.3, 21.1. Anal. Calcd for C_24_H_35_NO_6_: C, 66.49; H, 8.14; N, 3.23. Found: C, 66.61; H, 8.22; N, 3.35. HRMS (ESI-TOF): *m*/*z* [M + H]^+^: calculated for [C_24_H_36_NO_6_]^+^: 434.2537; found: 434.2529.
Benzyl (1S*,1′R*,2R*,5R*,7′S*,8′R*)-8′-allyl-1′,7′-dimethyl-2′,3′,5′,6′-tetraoxa-11′-azaspiro[adamantane-2,4′-bicyclo [5.3.1]undecane]-8′-carboxylate, **3nc**. White crystals. Mp = 148–149 °C. R_f_ = 0.58 (TLC, PE:EA, 10:1). ^1^H NMR (300.13 MHz, CDCl_3_), δ: 7.40–7.28 (m, 5H), 5.61–5.39 (m, 1H), 5.25–4.95 (m, 4H), 3.44 (br. s, 1H), 3.09–2.94 (m, 1H), 2.81 (dd, *J* = 14.1, 6.0 Hz, 1H), 2.45–2.65 (m, 2H), 2.07–1.93 (m, 4H), 1.89–1.44 (m, 15H), 1.44 (s, 3H). ^13^C NMR (75.48 MHz, CDCl_3_), δ: 172.9, 136.3, 133.4, 128.5, 128.0, 127.9, 118.7, 111.1, 93.6, 88.7, 66.7, 50.3, 37.7, 35.7, 34.6, 34.5, 33.6, 33.2, 33.1, 30.8, 28.1, 27.4, 26.7, 21.3, 21.2. Anal. Calcd for C_28_H_37_NO_6_: C, 69.54; H, 7.71; N, 2.90. Found: C, 69.70; H, 7.82; N, 2.99. HRMS (ESI-TOF): *m*/*z* [M + H]^+^: calculated for [C_28_H_38_NO_6_]^+^: 484.2694; found: 484.2683.

### 3.6. Evaluation of Cytotoxic Activity and Selectivity

#### 3.6.1. Cell Culture

Jurkat (Catalogue No. 88042803), K562 (Catalogue No. 89121407), A549 (Catalogue No. 86012804), and HEK293 (Catalogue No. 85120602) cell lines were obtained from the European Authentic Cell Culture Collection (ECACC, Wiltshire, UK) and further cultured according to established standard protocols and sterile methods. Cells were cultured in RPMI 1640 (for Jurkat and K562) and DMEM (for A549 and HEK293) media (Gibco, Waltham, MA, USA) supplemented with 4 μM glutamine, 10% FBS (Sigma, St. Louis, MO, USA) and 100 U/mL penicillin-streptomycin (Sigma). All cell types were cultured in a humidified atmosphere of 5% CO_2_ at 37 °C. The cells were subcultured at two-day intervals at a seeding density of 1 × 10^5^ cells per 24-well plate in RPMI with 10% FBS. The cells were then seeded into 24-well plates at a density of 5 × 10^4^ cells per well and incubated overnight.

#### 3.6.2. In Vitro Cytotoxicity

Dissolution of the compounds tested was initially performed in dimethyl sulfoxide (DMSO) with an initial solution of 100 mM in 10% DMSO. The solution was then diluted in complete culture medium, namely Dulbecco’s Modified Eagle Medium (DMEM) (Gibco, Waltham, MA, USA), or Roswell Park Memorial Institute medium (RPMI) (Gibco, Waltham, MA, USA). Substances were added at concentrations of 100, 10, 1, and 0.1 μM on the day after seeding and incubated for 24 h. The assessment of cell viability was conducted by employing 7-AAD (7-aminoactinomycin D) dye (eBioscience™, Thermo Fisher Scientific, Waltham, MA, USA). Following incubation with the test compounds, the cells were harvested, washed with phosphate-buffered saline (PBS), and subjected to centrifugation at 400× *g* for five minutes. The cell sediment was resuspended in 200 μL of staining buffer for flow cytometry (PBS without calcium and magnesium, 2.5% FBS) and stained with 1 mM 7-AAD dye solution for 15 min in the dark at room temperature. Subsequently, all experimental and control samples were analyzed on a BD FACSAria™ III Cell Sorter flow cytometer (BD, Franklin Lakes, NJ, USA). The CC_50_ values characterizing cytotoxicity parameters (i.e., the concentration of compound required for 50% inhibition of cell viability in vitro) were calculated, logC versus % inhibition was plotted, and statistical data processing was performed using Excel and GraphPad Prism v.8.0.2 (San Diego, CA, USA, 2019). Data obtained in three independent experiments were expressed as the mean of three measurements for each concentration ± standard deviation, relative to control values (0.1% DMSO), which were taken as 100%.

#### 3.6.3. Real-Time Cell Analysis (RTCA)

RTCA was performed using iCELLigence system (ACEA Biosciences Inc., San Diego, CA, USA), equipped with microelectronic plates (E-Plates) integrated with gold microelectrode arrays on a glass substrate in the bottom of the wells. The A549 cells (5 × 10^4^ cells/well) in 450 µL DMEM were seeded in each well of the E-Plate L8. After seeding the cells, the plate was left in a CO_2_ incubator for 30 min to synchronize cell attachment to the substrate. The test compounds and camptothecin in DMSO solutions were added to E-Plate L8 after approximately 48 h. The DMSO concentration did not exceed 0.1% (maximum: 0.2%). All wells and all compound concentrations contained the same amount of DMSO. The impedance was recorded in 1 h intervals. The cell index (i.e., cell-electrode impedance of the E-Plate well) was calculated using xCELLigence RTCA Software Pro Version 2.8 as (R_n_ − R_b_)/15, where R_b_ is the background impedance of the well measured with medium alone and R_n_ is the impedance of the well measured at any time (t) with cells present [51].

#### 3.6.4. Statistics

The normality of the distribution of the results obtained was checked. The Chi-square test was used for this purpose. The data were expressed as mean ± standard deviation. Student’s *t*-test was used for statistical comparison of results. A *p*-value less than 0.01 and less than 0.05 was considered statistically significant. Regression analysis and stepwise analysis of variance (ANOVA) were used for statistical analysis.

### 3.7. Bioassay of Fungicidal Activity

The antifungal activities were tested according to the conventional procedure [52] with 6 phytopathogenic fungi from different taxonomic classes: *Venturia inaequalis (V.i.)*, *Rhizoctonia solani (R.s.)*, *Fusarium oxysporum (F.o.)*, *Fusarium moniliforme (F.m.)*, *Bipolaris sorokiniana (B.s.)*, *Sclerotinia sclerotiorum (S.s.)*. The effect of the chemicals on mycelial radial growth was determined by dissolving a concentration 3 mg × mL^−1^ in acetone and suspending aliquots in potato-saccharose agar at 50 °C to achieve final concentrations of 0.3–30 µg × mL^−1^. The final acetone concentration of both fungicide-containing and control samples was 10 mL × L^−1^. Petri dishes containing 15 mL of the agar medium were inoculated by placing 2 mm mycelial agar discs on the agar surface. Plates were incubated at 25 °C, and radial growth was measured after 72 h. The mixed medium without a sample was used as the blank control. Three replicates of each test were carried out. The mycelium elongation diameter (mm) of fungi settlements was measured after 72 h of culture. The growth inhibition rates were calculated with the following equation: *I* = [(D_C_ − D_T_)/D_C_] ×100%. Here, I is the growth inhibition rates (%), D_C_ is the control settlement diameter (mm), and D_T_ is the treatment group fungi settlement diameter (mm). Commercially available agricultural fungicide Triadimefon was used as positive control.

## 4. Conclusions

A one-pot atom-economical and cost-effective reaction system for the assembly of aminodiperoxides via a three-component reaction of 1,5-diketones with geminal bishydroperoxides and ammonium acetate has been developed. This approach does not require catalyst or harsh conditions. Under optimal conditions, a wide range of aminodiperoxides was obtained in yields up to 88%. The synthesized aminodiperoxides demonstrated cytotoxicity and selectivity against Jurkat, K562, and A549 cancer cell lines and were significantly superior to cytostatic camptothecin. Furthermore, the aminodiperoxides exhibited fungicidal activity against phytopathogenic fungi of various taxonomic classes, in some cases comparable to the commercial fungicide Triadimefon. These findings highlight the potential of these unconventional compounds for medicinal chemistry and provide a new source for the development of anticancer and antifungal agents *via* the structural modification of aminodiperoxides. The results will be valuable for the discovery of new bioactive compounds and for the general design of multicomponent reactions involving electrophiles and nitrogen nucleophiles.

## Figures and Tables

**Table 1:** Optimization of the Reaction Conditions ^[a]^.

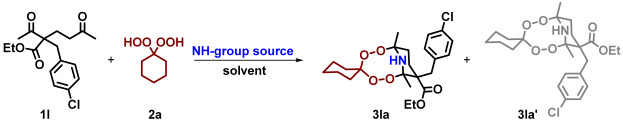						
**Entry**	**Mol of 2a/** **1 mol of 1a**	**NH-Group Source (eq. vs. 1l)**	**Solvent (mL)**	**Time, h**	**Temperature**	**Isolated Yield of 3la, %**
1 ^[b]^	1.5	NH_3_(aq) (5 eq.)	MeOH (2 mL)	1	20–25 °C	38
2 ^[b]^	1.5	NH_3(MeOH)_ (5 eq.)	MeOH (2 mL)	1	20–25 °C	31
3 ^[b]^	1.5	NH_4_OAc (5 eq.)	MeOH (3 mL)	1	20–25 °C	80
4 ^[b]^	1.5	NH_4_OAc (5 eq.)	THF (3 mL)	4	20–25 °C	72
5 ^[c]^	1.5	NH_4_OAc (3 eq.)	EtOH (3 mL)	1	20–25 °C	51
6 ^[c]^	1.5	NH_4_OAc (3 eq.)	EtOH (3 mL)	2	20–25 °C	71
7 ^[c]^	1.5	NH_4_OAc (3 eq.)	EtOH (3 mL)	24	20–25 °C	77
8 ^[c]^	1.5	NH_4_OAc (3 eq.)	EtOH (3 mL)	24	24 h at 20–25 °C then kept at −22 °C overnight ^[d]^	88
9 ^[c]^	1.5	HCOONH_4_ (3 eq.)	EtOH (3 mL)	24	24 h at 20–25 °C then kept at −22 °C overnight ^[d]^	67

^[a]^ A 22% aq. solution of NH_3_ (5 mol NH_3_/1 mol **1l**), 7M methanolic solution of NH_3_ (5 mol NH_3_/1 mol **1l**), NH_4_OAc (3.0–5.0 mol NH_4_OAc/1 mol **1l**) or HCOONH_4_ (3.0 mol HCOONH_4_/1 mol **1l**) and 1,1-dihydroperoxycyclohexane (**2a**) (1.5 mol **2a**/1.0 mol **1l**) were successively added with stirring to a solution of 1,5-diketone **1l** (0.200 g, 0.62 mmol; 1 eq.) in MeOH (2–3 mL), THF (3 mL), EtOH (3 mL) at 20–25 °C. The reaction mixture was stirred at 20–25 °C for 1–24 h. ^[b]^ The reaction mixture was extracted with CHCl_3_. ^[c]^ The resulting precipitate was filtered off and washed with a cold EtOH/H_2_O mixture (40:60, *v*/*v*). ^[d]^ The reaction mixture was stirred at 20–25 °C for 24 h, then was kept at −22 °C overnight.

**Table 2:** The cytotoxic activity of aminodiperoxides against Jurkat, K562, and A549 cancer cell lines and HEK293 normal cells (CC_50_ values, mean ± SD) ^[a]^.

Compd	Jurkat Cells CC_50_ [µM] ± SD	SI ^[b]^	K562 Cells CC_50_ [µM] ± SD	SI ^[c]^	A549 Cells CC_50_ [µM] ± SD	SI ^[d]^	HEK293 Cells CC_50_ [µM] ± SD
**3da**	26.3 ± 3.3	17.17	33.2 ± 2.8	13.61	45.0 ± 3.6	10.04	452.2 ± 13.4
**3ia**	41.9 ± 2.9	7.53	58.7 ± 4.9	5.37	87.7 ± 6.2	3.59	315.2 ± 12.8
**3ka**	11.9 ± 1.1	10.42	34.2 ± 2.8	3.62	65.8 ± 5.5	1.88	123.5 ± 10.5
**3la**	40.7 ± 3.2	5.79	43.6 ± 4.1	5.41	56.8 ± 5.2	4.15	235.9 ± 11.3
**3kb**	29.8 ± 1.9	10.76	48.4 ± 3.9	6.62	82.1 ± 7.7	3.9	320.3 ± 25.3
**3lb**	29.2 ± 1.9	14.78	47.7 ± 4.8	9.05	67.2 ± 5.9	6.43	432.0 ± 15.8
**3bc**	96.9 ± 7.2	10.12	150.2 ± 10.8	6.53	176.5 ± 15.7	5.56	980.5 ± 30.5
**3cc**	22.7 ± 3.9	38.54	47.6 ± 2.8	18.37	98.2 ± 7.1	8.92	875.3 ± 18.9
**3dc**	64.9 ± 5.8	13.61	78.3 ± 7.1	11.24	95.0 ± 10.2	9.26	880.0 ± 25.4
**3ec**	12.9 ± 1.4	67.09	19.6 ± 2.5	44.28	48.2 ± 3.7	17.98	866.1 ± 17.9
**3fc**	13.2 ± 1.1	43.86	26.1 ± 1.9	22.17	57.9 ± 4.3	10.01	579.0 ± 27.2
**3gc**	100.8 ± 9.5	9.8	154.2 ± 11.3	6.41	145.4 ± 12.2	6.8	987.9 ± 28.1
**3hc**	14.6 ± 0.9	53.58	28.7 ± 1.9	27.34	62.5 ± 2.3	12.54	784.4 ± 11.5
**3jc**	14.2 ± 1.3	49.27	26.0 ± 1.8	26.9	76.8 ± 8.9	9.1	698.1 ± 18.9
**3kc**	58.3 ± 4.9	13.06	98.3 ± 3.8	7.74	90.0 ± 7.6	8.46	761.1 ± 18.4
**3lc**	60.5 ± 5.1	7.49	77.4 ± 6.4	5.85	99.5 ± 8.2	4.55	453.3 ± 28.1
**3mc**	17.1 ± 1.7	46.38	31.3 ± 2.5	25.32	70.0 ± 5.5	11.31	791.3 ± 15.3
**3nc**	15.6 ± 2.1	54.76	34.4 ± 2.3	24.75	73.2 ± 6.4	11.65	852.6 ± 18.3
Artemisinin	83.7 ± 9.4	4.78	80.4 ± 6.6	4.97	95.6 ± 8.2	4.18	399.7 ± 25.4
Camptothecin	576.7 ± 44.2	1.19	569.9 ± 40.6	1.2	499.7 ± 38.9	1.37	685.8 ± 50.2

^[a]^ Flow cytometry, incubation time 24 h.; ^[b]^ Selectivity Index = CC_50_ (HEK293)/CC_50_ (Jurkat); ^[c]^ Selectivity Index = CC_50_ (HEK293)/CC_50_ (K562); ^[d]^ Selectivity Index = CC_50_ (HEK293)/CC_50_ (A549).

**Table 3:** Inhibition of mycelium growth of pathogenic fungi by aminodiperoxides **3**.

Run	Cmpd	Mycelium Growth Inhibition (I), % (C = 30 mg/L)					
		*V.i.*	*R.s.*	*F.o.*	*F.m.*	*B.s.*	*S.s.*
1	**3da**	30	35	9	25	30	11
2	**3ia**	40	44	9	31	51	18
3	**3ka**	22	21	6	20	44	13
4	**3la**	26	28	15	18	52	16
5	**3kb**	26	16	6	25	48	17
6	**3lb**	16	35	9	21	48	14
7	**3dc**	30	35	9	22	44	13
8	**3gc**	18	41	17	33	51	18
9	**3kc**	24	16	0	23	34	10
10	**3lc**	14	15	0	11	5	7
11	Triadimefon	41	43	77	87	44	61

## Data Availability

The data presented in this study are available in insert article and Supplementary Materials.

## Supplementary Materials

The following supporting information can be downloaded at https://www.mdpi.com/article/10.3390/molecules30244703/s1, ^1^H-, ^13^C-NMR, and HRMS of isolated compounds, single-crystal X-ray data for compounds **3la**, **3lb**, and **3lc** (PDF). Deposition numbers 2480403 (for **3la**), 2480408 (for **3lb**), and 2480409 (for **3lc**) contain the supplementary crystallographic data for this paper. These data are provided free of charge by the joint Cambridge Crystallographic Data Centre and Fachinformationszentrum Karlsruhe Access Structures service. The authors have cited additional references within the Supporting Information [53,54,55,56,57].
