# Supplementary material for: One-Pot Synthesis of Aminodiperoxides from 1,5-Diketones, Geminal Bishydroperoxides and Ammonium Acetate
Source: Molecules. 2025 Dec 8;30(24):4703. doi: 10.3390/molecules30244703 (PMC12736285; doi:10.3390/molecules30244703)

checkCIF/PLATON report

Structure factors have been supplied for datablock(s) bl-1273

THIS REPORT IS FOR GUIDANCE ONLY. IF USED AS PART OF A REVIEW PROCEDURE FOR PUBLICATION, IT SHOULD NOT REPLACE THE EXPERTISE OF AN EXPERIENCED CRYSTALLOGRAPHIC REFEREE.

No syntax errors found. CIF dictionary Interpreting this report

Datablock: bl-1273

```
Bond precision:   C-C = 0.0016 A           Wavelength=1.54184

Cell:            a=11.88540(5)    b=7.38572(3)       c=25.62494(10)
                 alpha=90         beta=90.7407(4)    gamma=90
Temperature:     100 K

                 Calculated                 Reported
Volume           2249.227(16)               2249.225(16)
Space group      P 21/n                     P 1 21/n 1
Hall group       -P 2yn                     -P 2yn
Moiety formula   C23 H32 Cl N O6            C23 H32 Cl N O6
Sum formula      C23 H32 Cl N O6            C23 H32 Cl N O6
Mr               453.95                     453.94
Dx,g cm-3        1.341                      1.341
Z                4                          4
Mu (mm-1)        1.836                      1.836
F000             968.0                      968.0
F000'            972.32
h,k,lmax         15,9,32                    15,9,32
Nref             4887                       4885
Tmin,Tmax        0.838,0.964                0.855,1.000
Tmin'            0.802

Correction method= # Reported T Limits: Tmin=0.855 Tmax=1.000
AbsCorr = MULTI-SCAN

Data completeness= 1.000            Theta(max)= 79.584

                                                     wR2(reflections)=
R(reflections)= 0.0340( 4876)                        0.0862( 4885)

S = 1.067                  Npar= 287
```

The following ALERTS were generated. Each ALERT has the format
test-name_ALERT_alert-type_alert-level.
Click on the hyperlinks for more details of the test.

Alert level G

```
PLAT142_ALERT_4_G s.u. on b - Axis Small or Missing .............    0.00003 Ang.
PLAT143_ALERT_4_G s.u. on c - Axis Small or Missing .............    0.00010 Ang.
PLAT145_ALERT_4_G s.u. on beta     Small or Missing .............     0.0004 Degree
PLAT395_ALERT_2_G Deviating  X-O-Y   Angle From 120 for O1       .      109.3 Degree
PLAT395_ALERT_2_G Deviating  X-O-Y   Angle From 120 for O2       .      107.1 Degree
PLAT395_ALERT_2_G Deviating  X-O-Y   Angle From 120 for O3       .      106.7 Degree
PLAT395_ALERT_2_G Deviating  X-O-Y   Angle From 120 for O4       .      108.9 Degree
PLAT793_ALERT_4_G Model has Chirality at C2          (Centro SPGR)          S Verify
PLAT793_ALERT_4_G Model has Chirality at C3          (Centro SPGR)          R Verify
PLAT793_ALERT_4_G Model has Chirality at C6          (Centro SPGR)          R Verify
PLAT912_ALERT_4_G Missing # of FCF Reflections Above STh/L=  0.600          3 Note
PLAT961_ALERT_5_G Dataset Contains no Negative Intensities .......     Please Check
PLAT978_ALERT_2_G Number C-C Bonds with Positive Residual Density.         18 Info
```

0 **ALERT level A** = Most likely a serious problem - resolve or explain
0 **ALERT level B** = A potentially serious problem, consider carefully
0 **ALERT level C** = Check. Ensure it is not caused by an omission or oversight
13 **ALERT level G** = General information/check it is not something unexpected

0 ALERT type 1 CIF construction/syntax error, inconsistent or missing data
5 ALERT type 2 Indicator that the structure model may be wrong or deficient
0 ALERT type 3 Indicator that the structure quality may be low
7 ALERT type 4 Improvement, methodology, query or suggestion
1 ALERT type 5 Informative message, check

It is advisable to attempt to resolve as many as possible of the alerts in all categories. Often the minor alerts point to easily fixed oversights, errors and omissions in your CIF or refinement strategy, so attention to these fine details can be worthwhile. In order to resolve some of the more serious problems it may be necessary to carry out additional measurements or structure refinements. However, the purpose of your study may justify the reported deviations and the more serious of these should normally be commented upon in the discussion or experimental section of a paper or in the "special_details" fields of the CIF. checkCIF was carefully designed to identify outliers and unusual parameters, but every test has its limitations and alerts that are not important in a particular case may appear. Conversely, the absence of alerts does not guarantee there are no aspects of the results needing attention. It is up to the individual to critically assess their own results and, if necessary, seek expert advice.

Publication of your CIF in IUCr journals

A basic structural check has been run on your CIF. These basic checks will be run on all CIFs submitted for publication in IUCr journals (*Acta Crystallographica*, *Journal of Applied Crystallography*, *Journal of Synchrotron Radiation*); however, if you intend to submit to *Acta Crystallographica Section C* or *E* or *IUCrData*, you should make sure that full publication checks are run on the final version of your CIF prior to submission.

Publication of your CIF in other journals

Please refer to the *Notes for Authors* of the relevant journal for any special instructions relating to CIF submission.

PLATON version of 20/01/2022; check.def file version of 19/01/2022

Datablock bl-1273 - ellipsoid plot

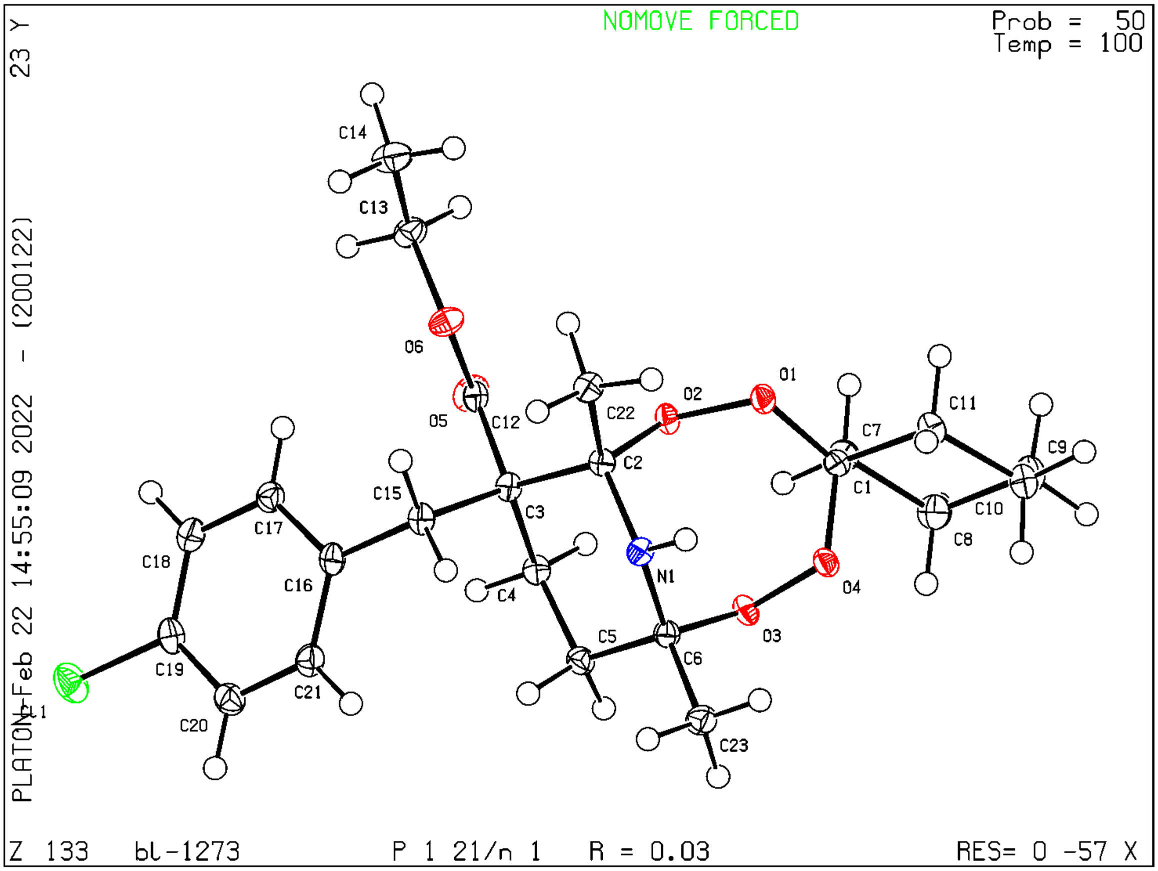

Supplement: Supplementary file 1 [file molecules-30-04703-s001.zip › 3la_checkcif.pdf]
