# Supplementary material for: One-Pot Synthesis of Aminodiperoxides from 1,5-Diketones, Geminal Bishydroperoxides and Ammonium Acetate
Source: Molecules. 2025 Dec 8;30(24):4703. doi: 10.3390/molecules30244703 (PMC12736285; doi:10.3390/molecules30244703)

checkCIF/PLATON report

Structure factors have been supplied for datablock(s) BL-1352

THIS REPORT IS FOR GUIDANCE ONLY. IF USED AS PART OF A REVIEW PROCEDURE FOR PUBLICATION, IT SHOULD NOT REPLACE THE EXPERTISE OF AN EXPERIENCED CRYSTALLOGRAPHIC REFEREE.

No syntax errors found. CIF dictionary Interpreting this report

Datablock: BL-1352

Bond precision: C-C = 0.0017 A Wavelength=1.54184

Cell:	a=13.10198(9)	b=14.61105(10)	c=12.85876(9)
	alpha=90	beta=106.5782(8)	gamma=90
Temperature:	103 K		

	Calculated	Reported
Volume	2359.27(3)	2359.27(3)
Space group	P 21/c	P 1 21/c 1
Hall group	-P 2ybc	-P 2ybc
Moiety formula	C24 H34 Cl N O6	C24 H34 Cl N O6
Sum formula	C24 H34 Cl N O6	C24 H34 Cl N O6
Mr	467.97	467.97
Dx,g cm-3	1.317	1.317
Z	4	4
Mu (mm-1)	1.766	1.766
F000	1000.0	1000.0
F000'	1004.39	
h,k,lmax	16,18,16	16,18,16
Nref	5159	5140
Tmin,Tmax	0.568,0.899	0.348,1.000
Tmin'	0.408	

Correction method= # Reported T Limits: Tmin=0.348 Tmax=1.000
AbsCorr = GAUSSIAN

Data completeness= 0.996 Theta(max)= 79.996

R(reflections)= 0.0343(4879) wR2(reflections)= 0.0888(5140)

S = 1.085 Npar= 304

The following ALERTS were generated. Each ALERT has the format
test-name_ALERT_alert-type_alert-level.
Click on the hyperlinks for more details of the test.

Alert level C

```
PLAT220_ALERT_2_C NonSolvent   Resd 1  C   Ueq(max)/Ueq(min) Range        3.2 Ratio
PLAT415_ALERT_2_C Short Inter D-H..H-X       H5A      ..H11      .       2.12 Ang.
                                              x,3/2-y,1/2+z  =     4_576 Check
PLAT420_ALERT_2_C D-H Bond Without Acceptor  N11      --H11      .     Please Check
```

Alert level G

```
PLAT002_ALERT_2_G Number of Distance or Angle Restraints on AtSite          5 Note
PLAT142_ALERT_4_G s.u. on b - Axis Small or Missing .............    0.00010 Ang.
PLAT143_ALERT_4_G s.u. on c - Axis Small or Missing .............    0.00009 Ang.
PLAT171_ALERT_4_G The CIF-Embedded .res File Contains EADP Records          2 Report
PLAT176_ALERT_4_G The CIF-Embedded .res File Contains SADI Records          2 Report
PLAT230_ALERT_2_G Hirshfeld Test Diff for    C21A     --C22A     .        5.5 s.u.
PLAT301_ALERT_3_G Main Residue  Disorder ..............(Resd    1)         6% Note
PLAT395_ALERT_2_G Deviating  X-O-Y   Angle From 120 for O1       .      109.1 Degree
PLAT395_ALERT_2_G Deviating  X-O-Y   Angle From 120 for O2       .      106.5 Degree
PLAT395_ALERT_2_G Deviating  X-O-Y   Angle From 120 for O8       .      108.3 Degree
PLAT395_ALERT_2_G Deviating  X-O-Y   Angle From 120 for O9       .      109.2 Degree
PLAT793_ALERT_4_G Model has Chirality at C3          (Centro SpGr)          R Verify
PLAT793_ALERT_4_G Model has Chirality at C6          (Centro SpGr)          R Verify
PLAT793_ALERT_4_G Model has Chirality at C7          (Centro SpGr)          S Verify
PLAT860_ALERT_3_G Number of Least-Squares Restraints .............          2 Note
PLAT912_ALERT_4_G Missing # of FCF Reflections Above STh/L=  0.600         18 Note
PLAT969_ALERT_5_G The 'Henn et al.' R-Factor-gap value ..........       3.329 Note
              Predicted wR2: Based on SigI**2  2.67 or SHELX Weight  8.19
PLAT978_ALERT_2_G Number C-C Bonds with Positive Residual Density.         18 Info
```

0 **ALERT level A** = Most likely a serious problem - resolve or explain
0 **ALERT level B** = A potentially serious problem, consider carefully
3 **ALERT level C** = Check. Ensure it is not caused by an omission or oversight
18 **ALERT level G** = General information/check it is not something unexpected

0 ALERT type 1 CIF construction/syntax error, inconsistent or missing data
10 ALERT type 2 Indicator that the structure model may be wrong or deficient
2 ALERT type 3 Indicator that the structure quality may be low
8 ALERT type 4 Improvement, methodology, query or suggestion
1 ALERT type 5 Informative message, check

It is advisable to attempt to resolve as many as possible of the alerts in all categories. Often the minor alerts point to easily fixed oversights, errors and omissions in your CIF or refinement strategy, so attention to these fine details can be worthwhile. In order to resolve some of the more serious problems it may be necessary to carry out additional measurements or structure refinements. However, the purpose of your study may justify the reported deviations and the more serious of these should normally be commented upon in the discussion or experimental section of a paper or in the "special_details" fields of the CIF. checkCIF was carefully designed to identify outliers and unusual parameters, but every test has its limitations and alerts that are not important in a particular case may appear. Conversely, the absence of alerts does not guarantee there are no aspects of the results needing attention. It is up to the individual to critically assess their own results and, if necessary, seek expert advice.

Publication of your CIF in IUCr journals

A basic structural check has been run on your CIF. These basic checks will be run on all CIFs submitted for publication in IUCr journals (*Acta Crystallographica*, *Journal of Applied Crystallography*, *Journal of Synchrotron Radiation*); however, if you intend to submit to *Acta Crystallographica Section C* or *E* or *IUCrData*, you should make sure that full publication checks are run on the final version of your CIF prior to submission.

Publication of your CIF in other journals

Please refer to the *Notes for Authors* of the relevant journal for any special instructions relating to CIF submission.

PLATON version of 02/02/2025; check.def file version of 02/02/2025

Datablock BL-1352 - ellipsoid plot

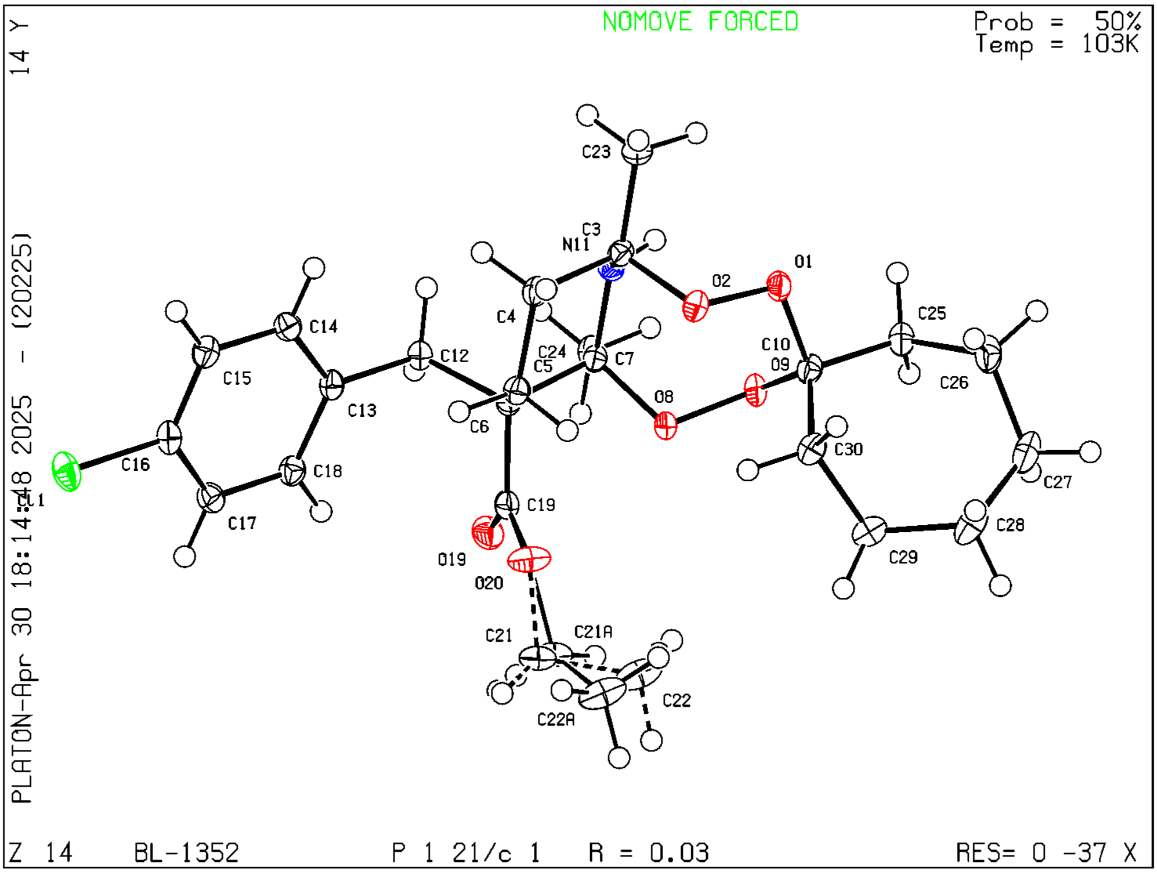

Supplement: Supplementary file 1 [file molecules-30-04703-s001.zip › 3lb_checkcif-BL-1352.pdf]
