# Supplementary material for: One-Pot Synthesis of Aminodiperoxides from 1,5-Diketones, Geminal Bishydroperoxides and Ammonium Acetate
Source: Molecules. 2025 Dec 8;30(24):4703. doi: 10.3390/molecules30244703 (PMC12736285; doi:10.3390/molecules30244703)
Supplement: Supplementary file 1 [file molecules-30-04703-s001.zip › Supporting_information_19_11_25.pdf]

Supporting Information

One-pot Synthesis of Aminodiperoxides from 1,5-Diketones, geminal Bishydroperoxides and Ammonium Acetate

Yulia Yu. Belyakova,[1] Viktoria E. Tsykunova,[1] Peter S. Radulov,[1] Lilya U. Dzhemileva,[1] Roman A. Novikov,[1] Alexey I. Ilovaisky,[1,2] Ivan A. Yaremenko*[1] and Alexander O. Terent'ev*[1]

[1] N. D. Zelinsky Institute of Organic Chemistry, Russian Academy of Sciences, 47 Leninsky prosp., Moscow, 119991, Russian Federation, e-mail: yaremenko@ioc.ac.ru, terentev@ioc.ac.ru

[2] All-Russian Research Institute for Phytopathology, Russian Federation, 143050 Moscow Region, B. Vyazyomy

Table of contents

NMR spectra of aminodiperoxides **3aa**, **3da**, **3ia**, **3ka**, **3la**, **3kb**, **3lb**, **3ac–3hc**, **3jc-3nc** 3

NMR monitoring of the three-component reaction of 1,5-diketone **1l** with geminal bishydroperoxide and NH_4OAc in EtOH-*d6* 43

HRMS spectra of aminodiperoxides **3aa**, **3da**, **3ia**, **3ka**, **3la**, **3kb**, **3lb**, **3ac–3hc**, **3jc-3nc** 54

HRMS spectra of intermediates **4**, **5**, **6**, and **8/8'** or products **3/3'** 74

X-Ray data of **3la**, **3lb** and **3lc** 78

References 114

NMR spectra of aminodiperoxides 3aa, 3da, 3ia, 3ka, 3la, 3kb, 3lb, 3ac–3hc, 3jc-3nc

^{1}H NMR (300.13 MHz, CDCl$_3$). (1*R,7*S**)-1,7-dimethyl-2,3,5,6-tetraoxa-11-azaspiro[bicyclo[5.3.1]undecane-4,1'-cyclohexane], 3aa**

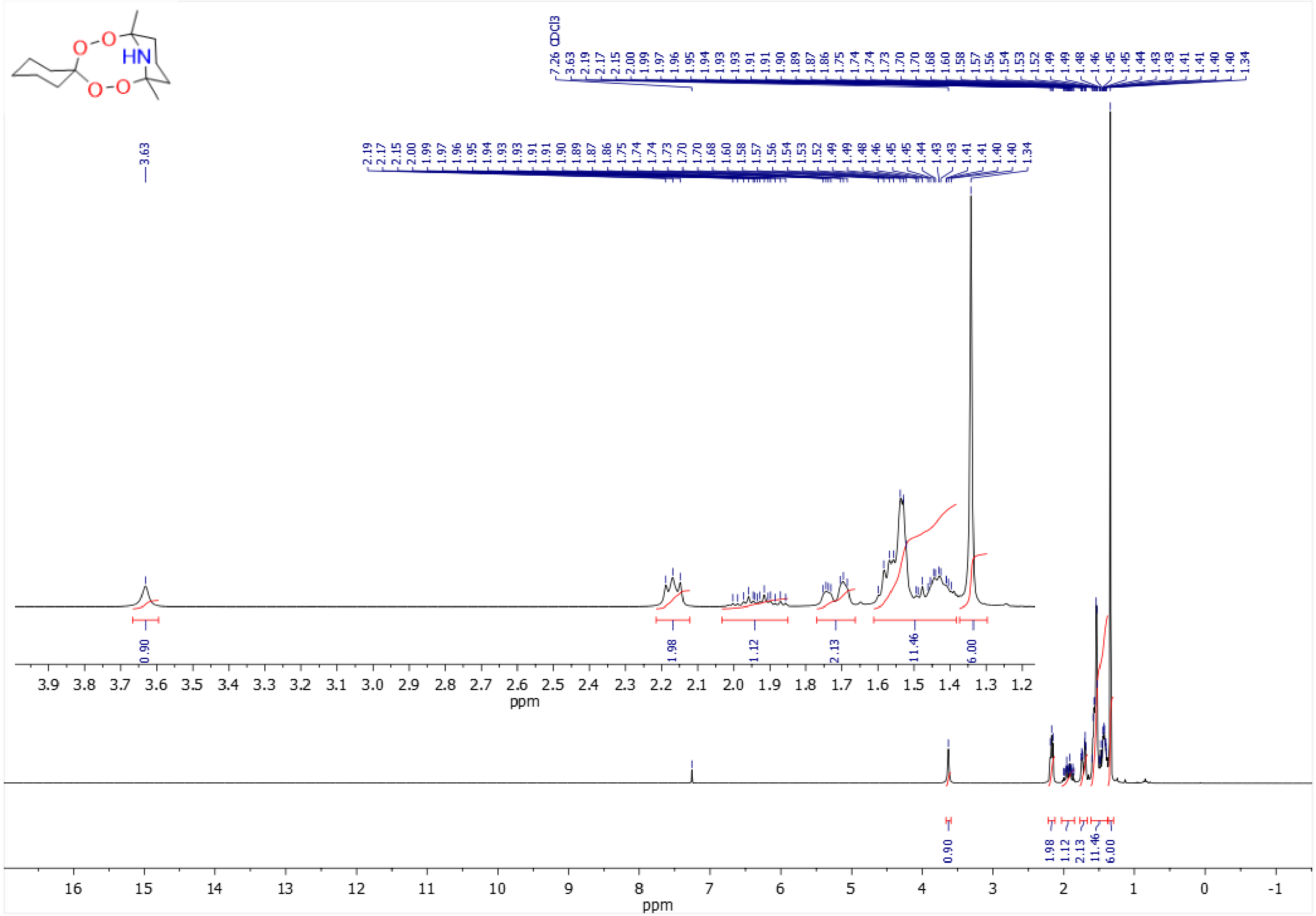

^{13}C NMR (75.48 MHz, CDCl3). (1*R,7*S**)-1,7-dimethyl-2,3,5,6-tetraoxa-11-azaspiro[bicyclo[5.3.1]undecane-4,1'-cyclohexane], 3aa**

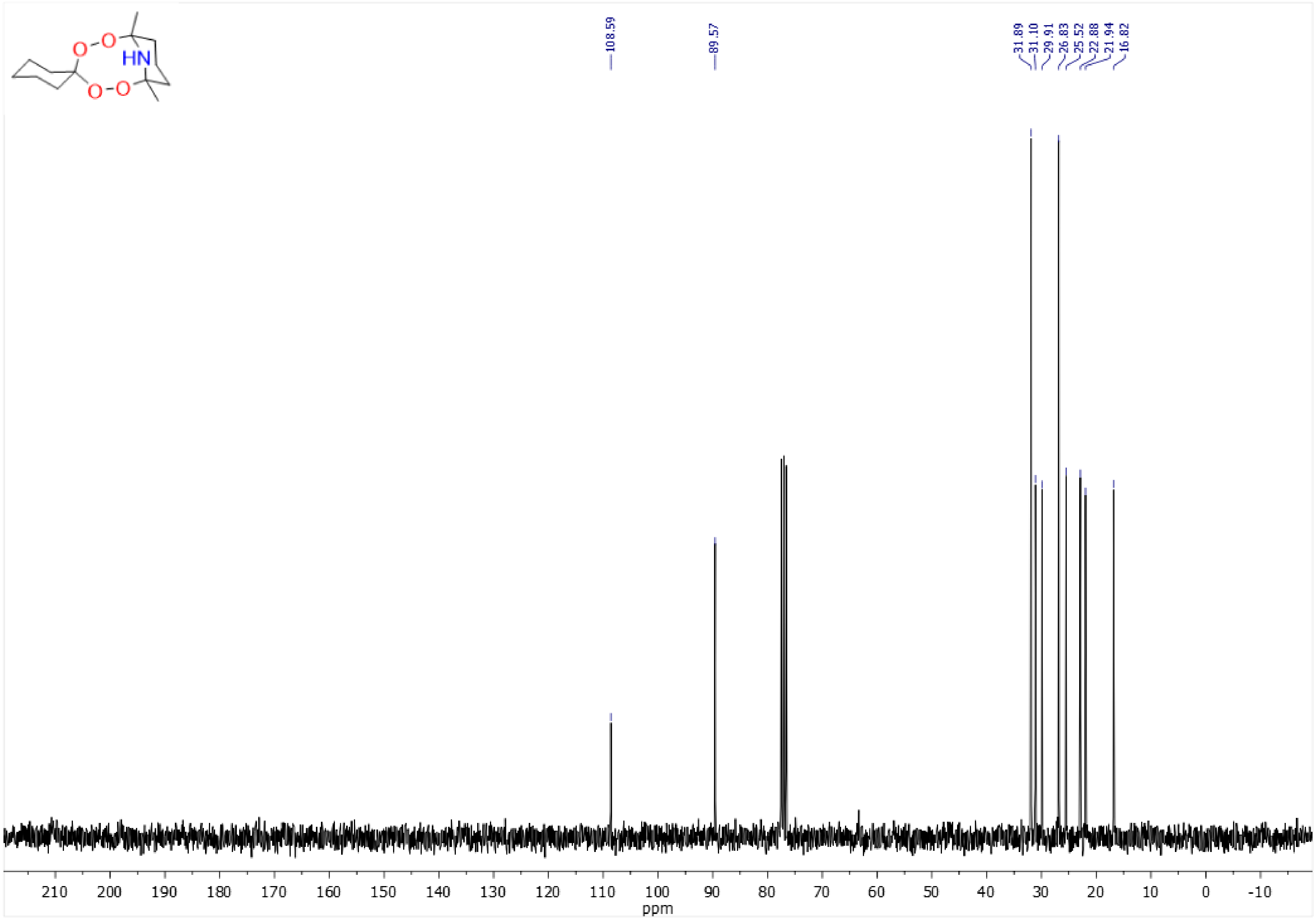

^{1}H NMR (300.13 MHz, $CDCl_3$). Ethyl (1*R,7*S**,8*S**)-8-butyl-1,7-dimethyl-2,3,5,6-tetraoxa-11-azaspiro[bicyclo[5.3.1]undecane-4,1'-cyclohexane]-8-carboxylate, 3da**

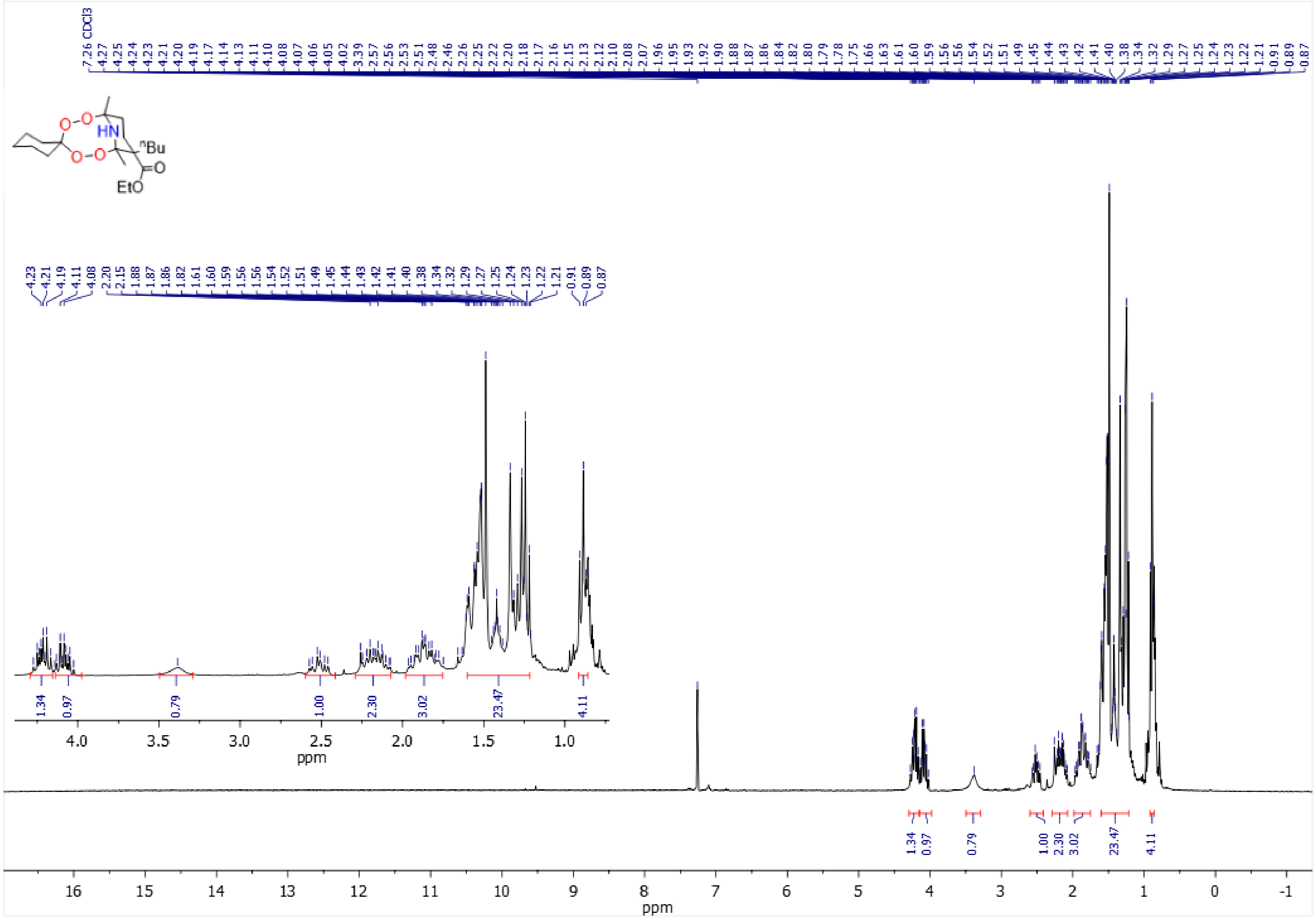

^{13}C NMR (75.48 MHz, $CDCl_3$). Ethyl (1*R,7*S**,8*S**)-8-butyl-1,7-dimethyl-2,3,5,6-tetraoxa-11-azaspiro[bicyclo[5.3.1]undecane-4,1'-cyclohexane]-8-carboxylate, 3da**

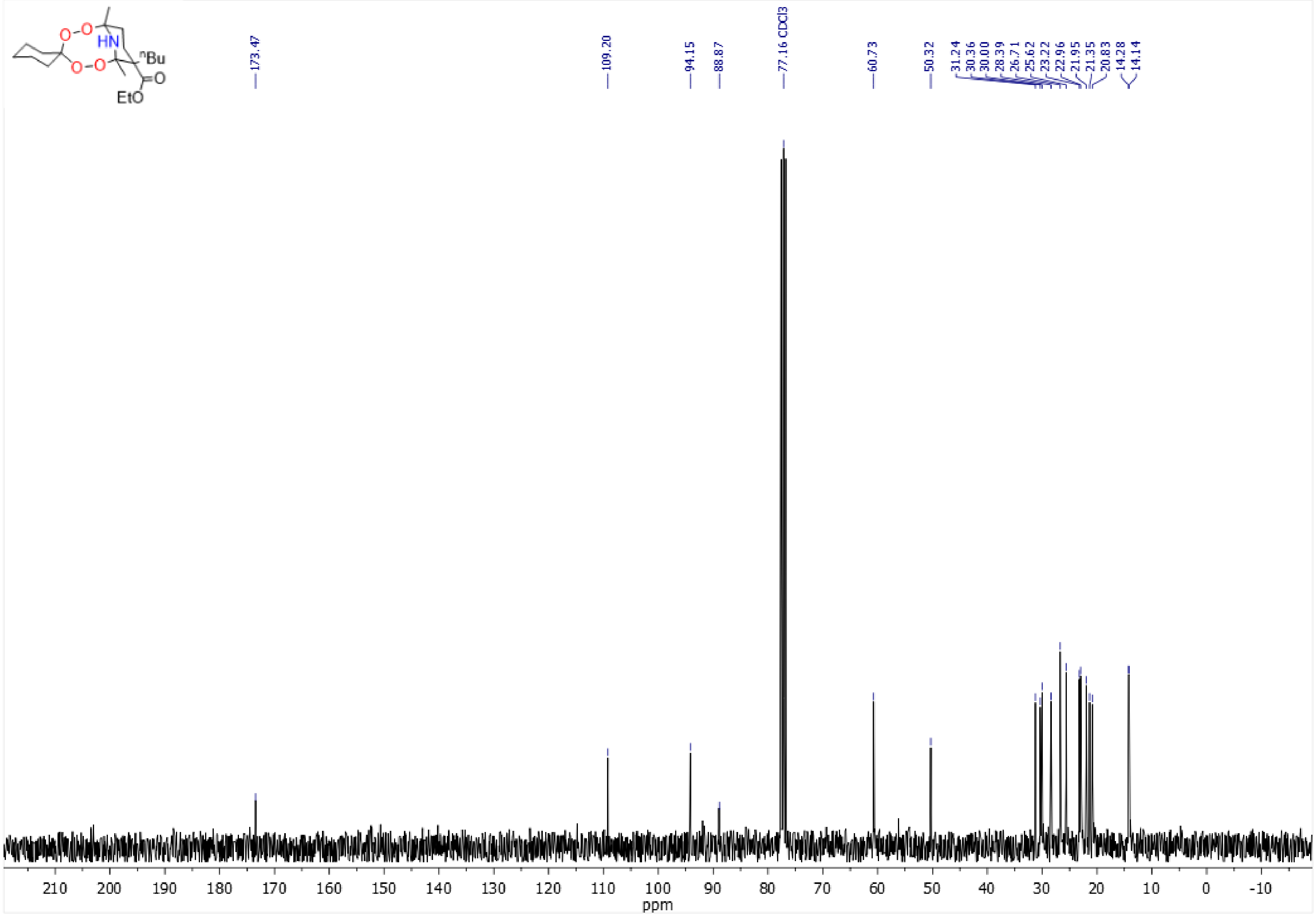

^{1}H NMR (300.13 MHz, $CDCl_3$). Ethyl (1*R,7*S**,8*R**)-1,7-dimethyl-8-(4-methylbenzyl)-2,3,5,6-tetraoxa-11-azaspiro[bicyclo[5.3.1]undecane-4,1'-cyclohexane]-8-carboxylate, 3ha**

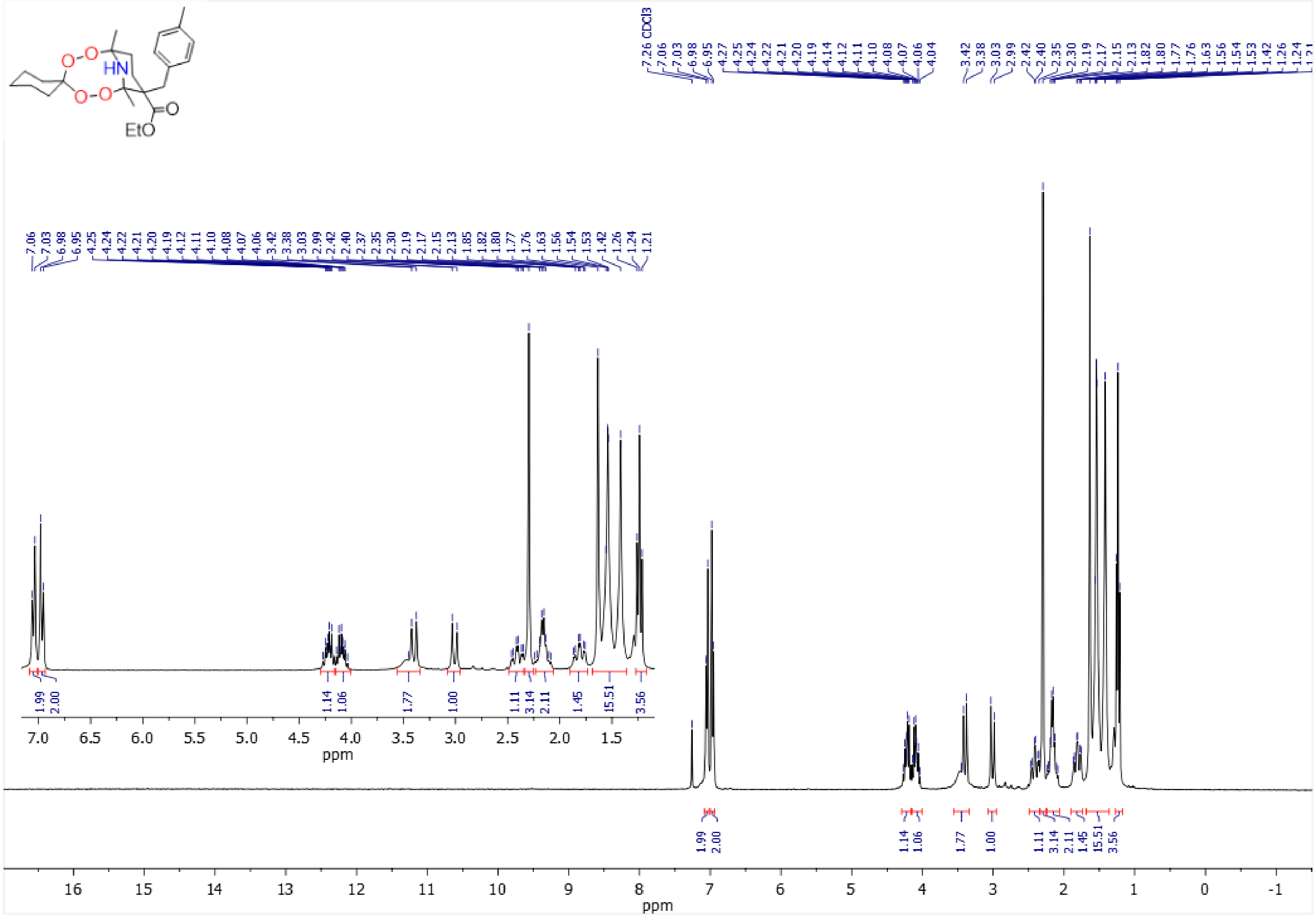

^{13}C NMR (75.48 MHz, $CDCl_3$). Ethyl (1*R,7*S**,8*R**)-1,7-dimethyl-8-(4-methylbenzyl)-2,3,5,6-tetraoxa-11-azaspiro[bicyclo[5.3.1]undecane-4,1'-cyclohexane]-8-carboxylate, 3ha**

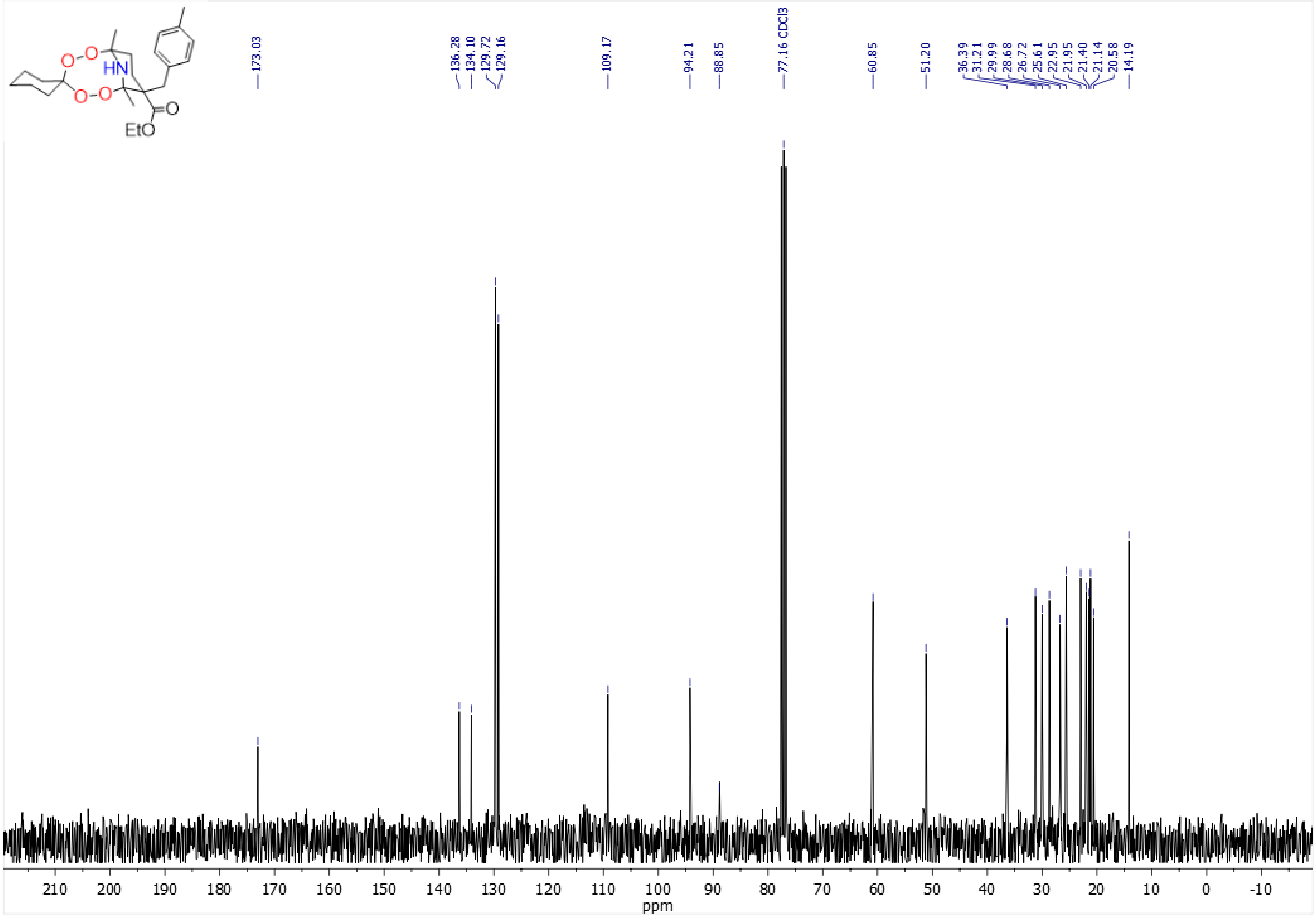

^{1}H NMR (300.13 MHz, $CDCl_3$). Ethyl (1*R,7*S**,8*R**)-8-(4-fluorobenzyl)-1,7-dimethyl-2,3,5,6-tetraoxa-11-azaspiro[bicyclo[5.3.1]undecane-4,1'-cyclohexane]-8-carboxylate, 3ka**

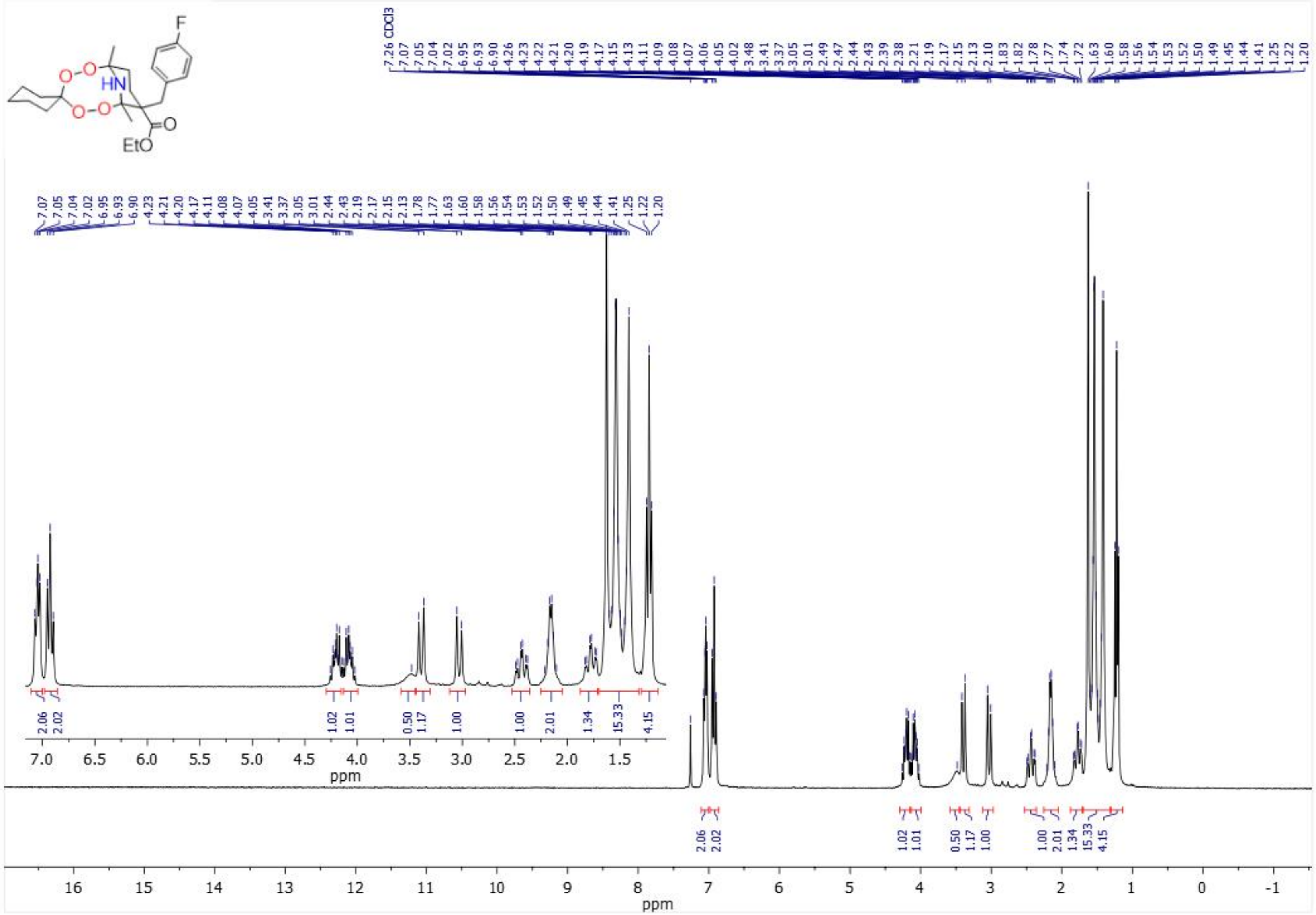

^{13}C NMR (75.48 MHz, CDCl₃). Ethyl (1*R,7*S**,8*R**)-8-(4-fluorobenzyl)-1,7-dimethyl-2,3,5,6-tetraoxa-11-azaspiro[bicyclo[5.3.1]undecane-4,1'-cyclohexane]-8-carboxylate, 3ka**

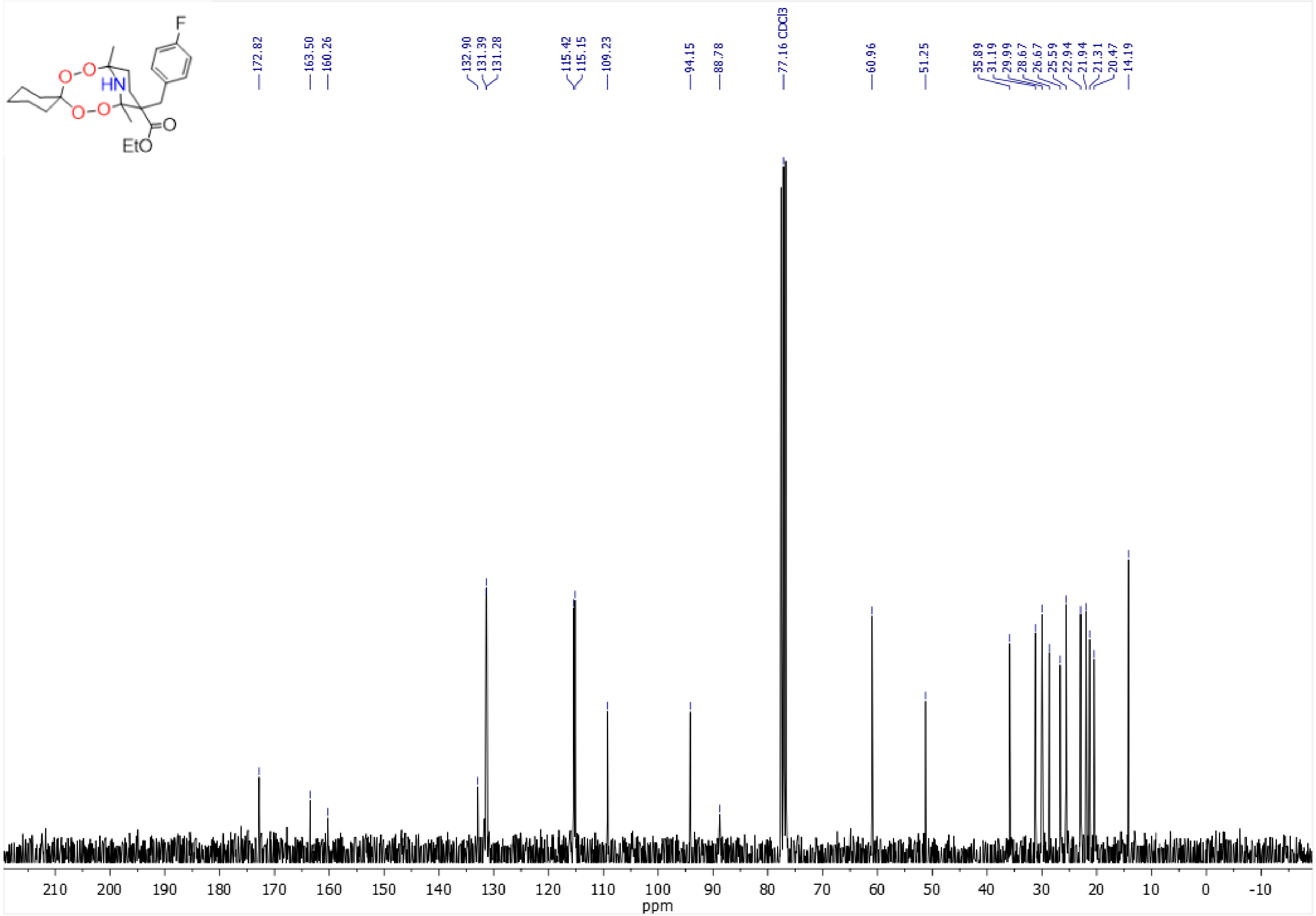

^{1}H NMR (300.13 MHz, CDCl3). Ethyl (1*R,7*S**,8*R**)-8-(4-chlorobenzyl)-1,7-dimethyl-2,3,5,6-tetraoxa-11-azaspiro[bicyclo[5.3.1]undecane-4,1'-cyclohexane]-8-carboxylate, 3ka**

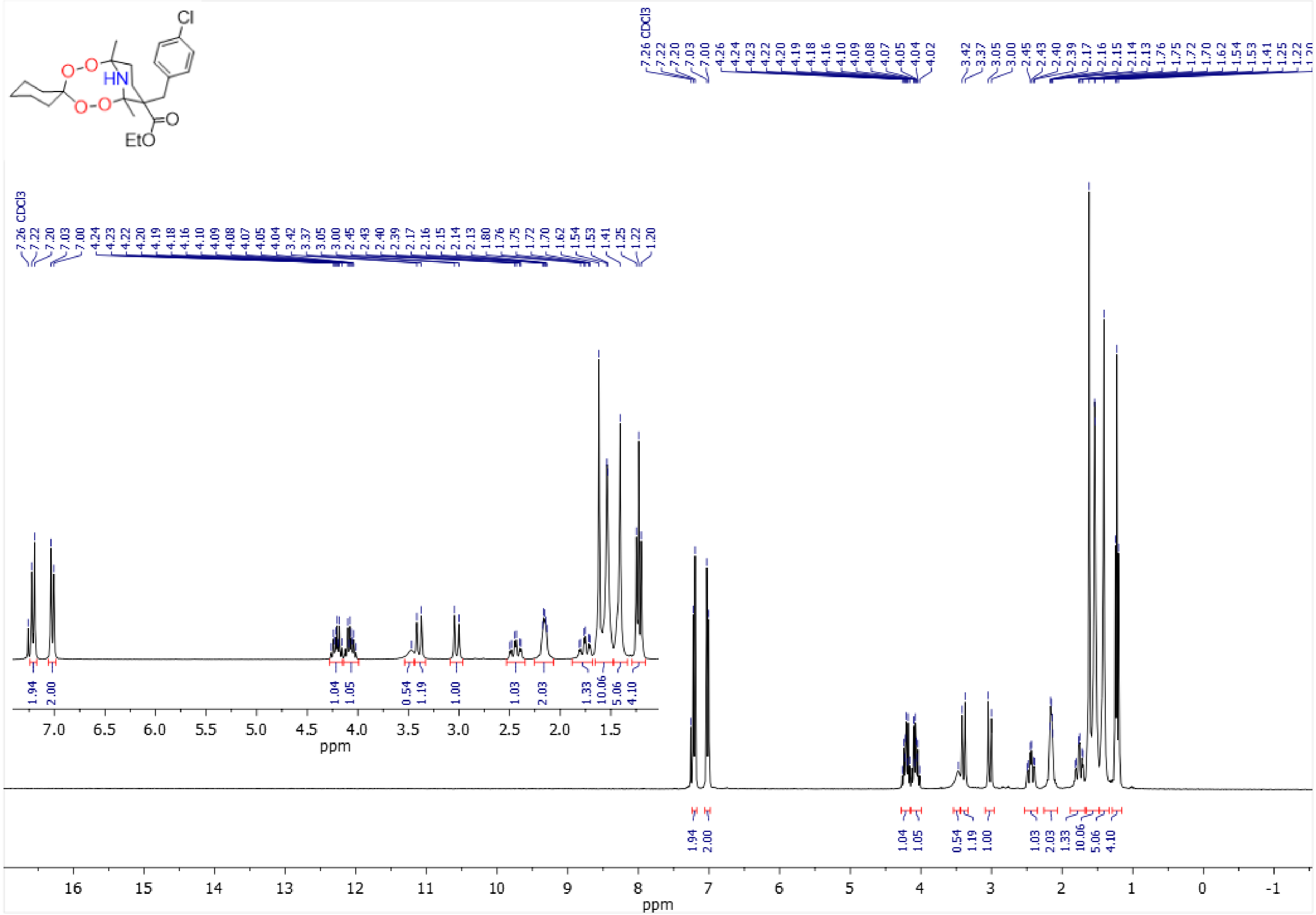

^{13}C NMR (75.48 MHz, $CDCl_3$). Ethyl (1*R,7*S**,8*R**)-8-(4-chlorobenzyl)-1,7-dimethyl-2,3,5,6-tetraoxa-11-azaspiro[bicyclo[5.3.1]undecane-4,1'-cyclohexane]-8-carboxylate, 3ka**

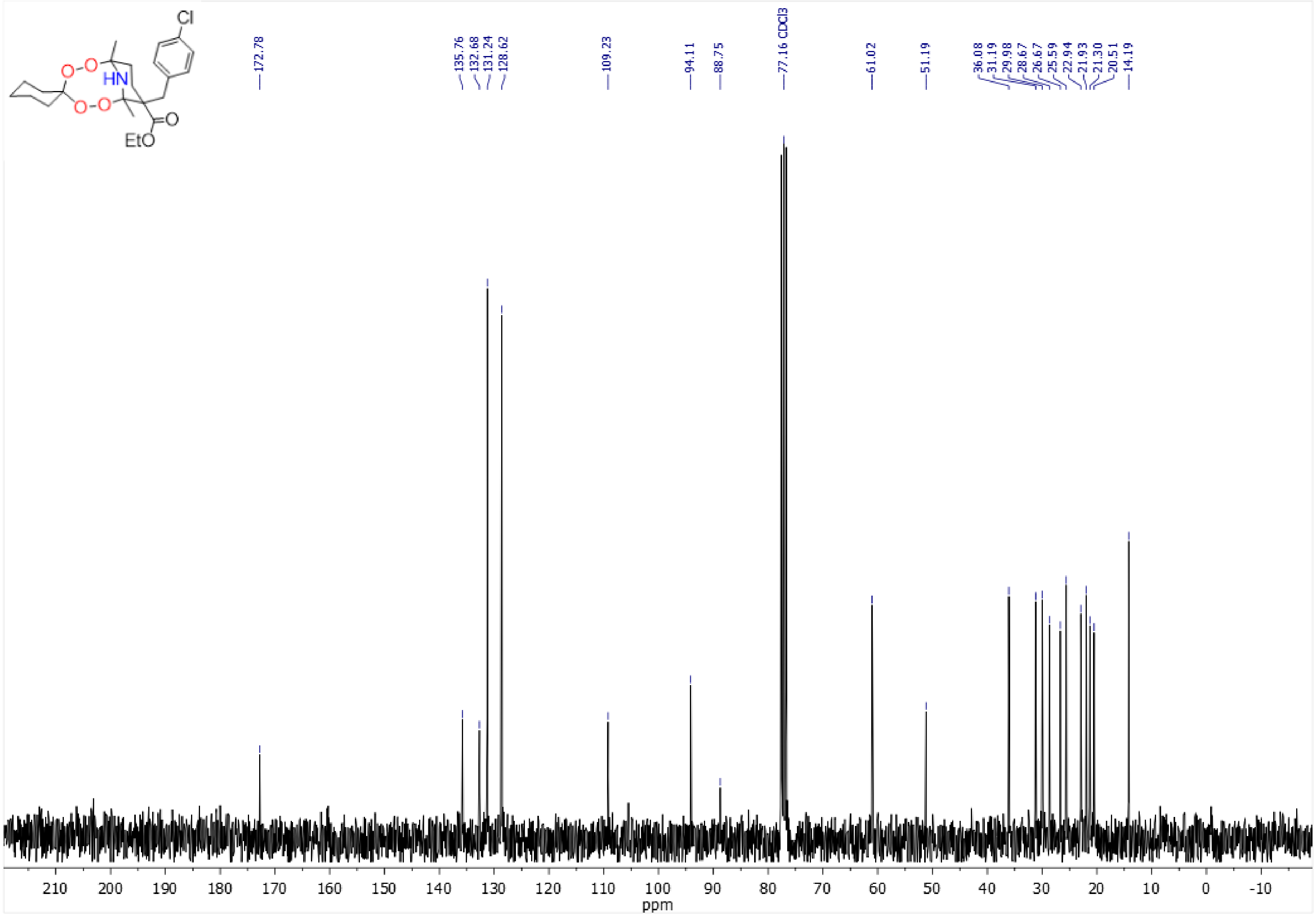

^{1}H NMR (300.13 MHz, $CDCl_3$). Ethyl (1*R,7*S**,8*R**)-8-(4-fluorobenzyl)-1,7-dimethyl-2,3,5,6-tetraoxa-11-azaspiro[bicyclo[5.3.1]undecane-4,1'-cycloheptane]-8-carboxylate, 3kb**

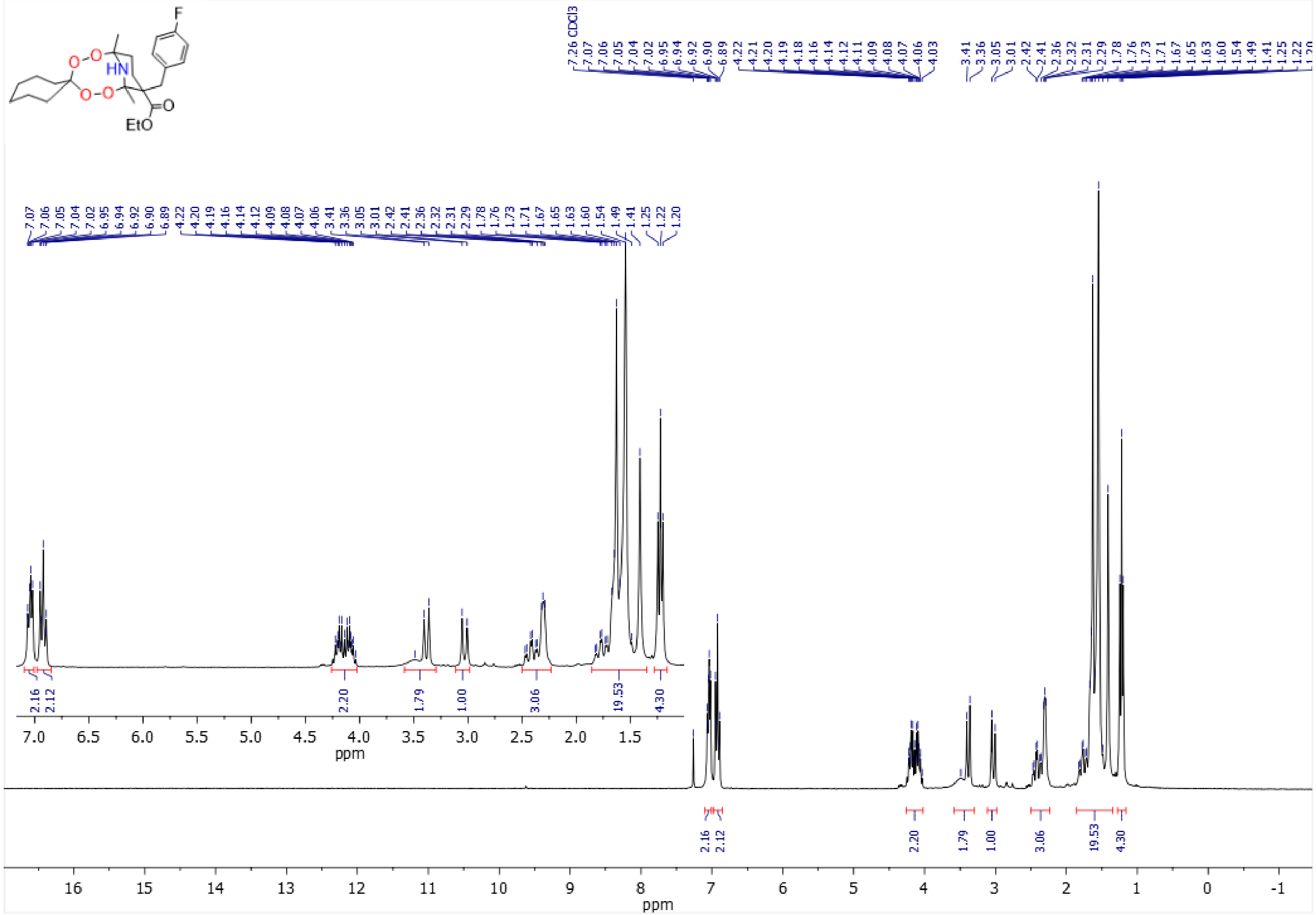

^{13}C NMR (75.48 MHz, CDCl$_3$). Ethyl (1*R,7*S**,8*R**)-8-(4-fluorobenzyl)-1,7-dimethyl-2,3,5,6-tetraoxa-11-azaspiro[bicyclo[5.3.1]undecane-4,1'-cycloheptane]-8-carboxylate, 3kb**

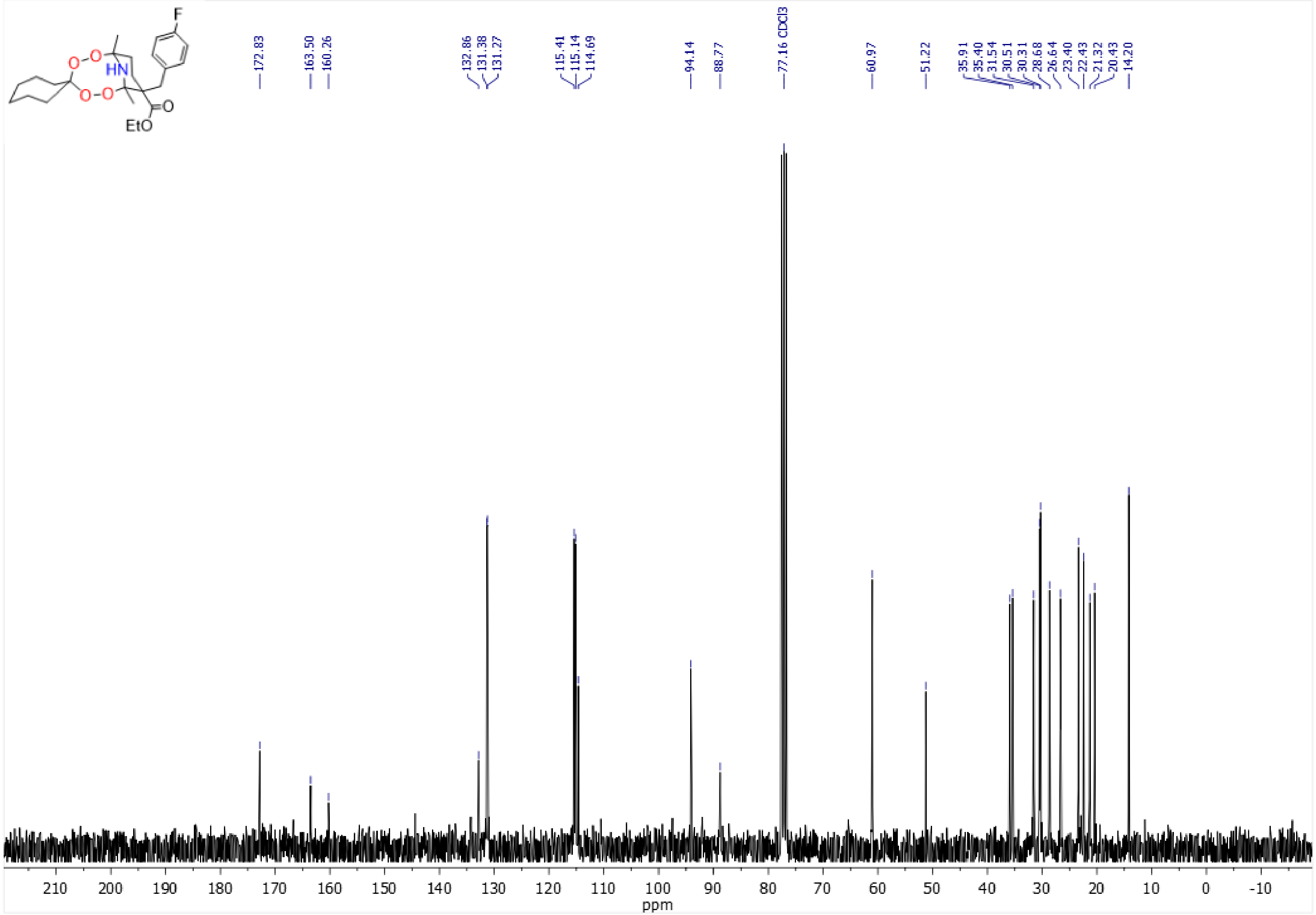

^{1}H NMR (300.13 MHz, $CDCl_3$). Ethyl (1*R**,7*S**,8*R**)-8-(4-chlorobenzyl)-1,7-dimethyl-2,3,5,6-tetraoxa-11-azaspiro[bicyclo[5.3.1]undecane-4,1'-cycloheptane]-8-carboxylate, 3lb

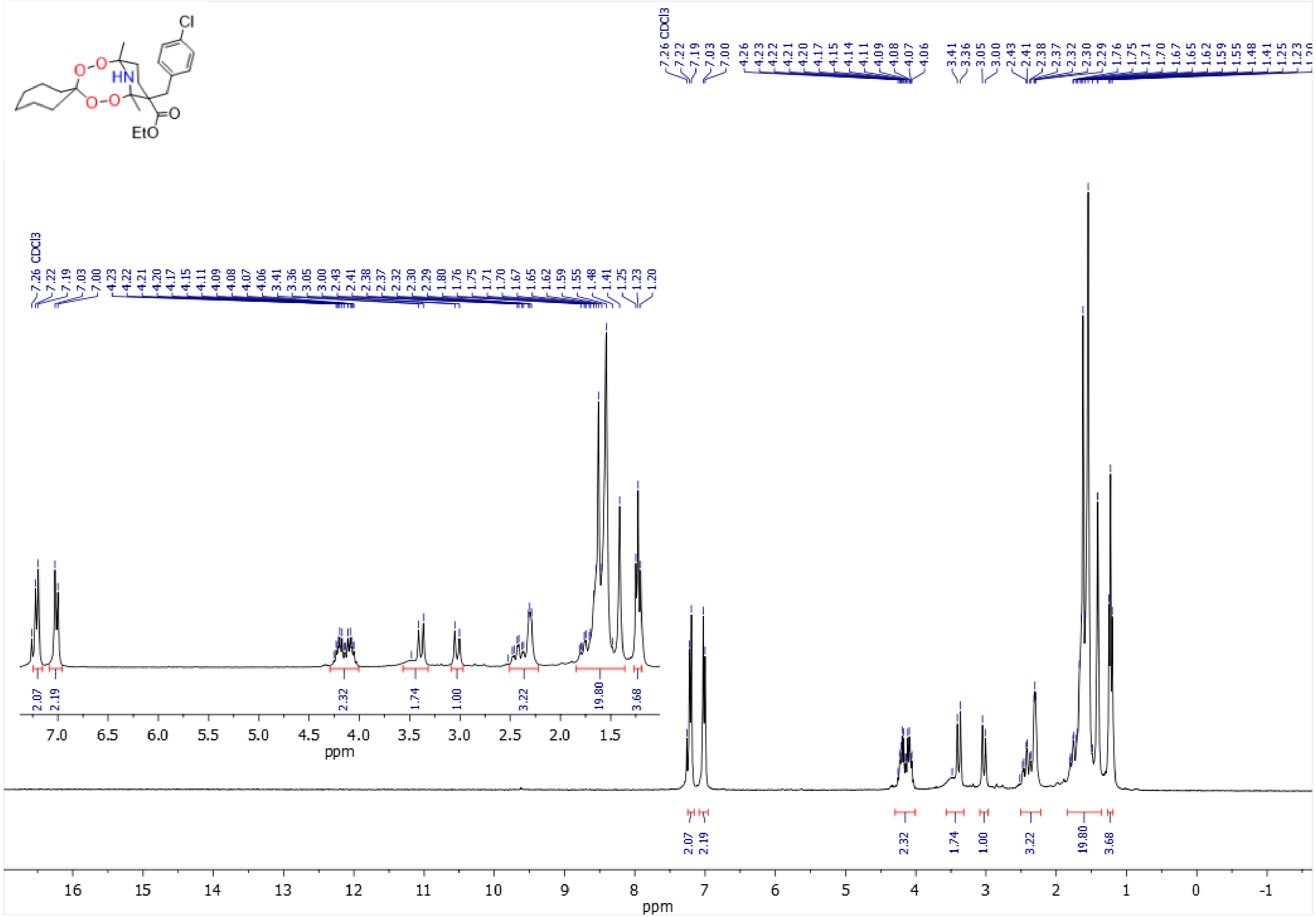

^{13}C NMR (75.48 MHz, $CDCl_3$). Ethyl (1*R,7*S**,8*R**)-8-(4-chlorobenzyl)-1,7-dimethyl-2,3,5,6-tetraoxa-11-azaspiro[bicyclo[5.3.1]undecane-4,1'-cycloheptane]-8-carboxylate, 3lb**

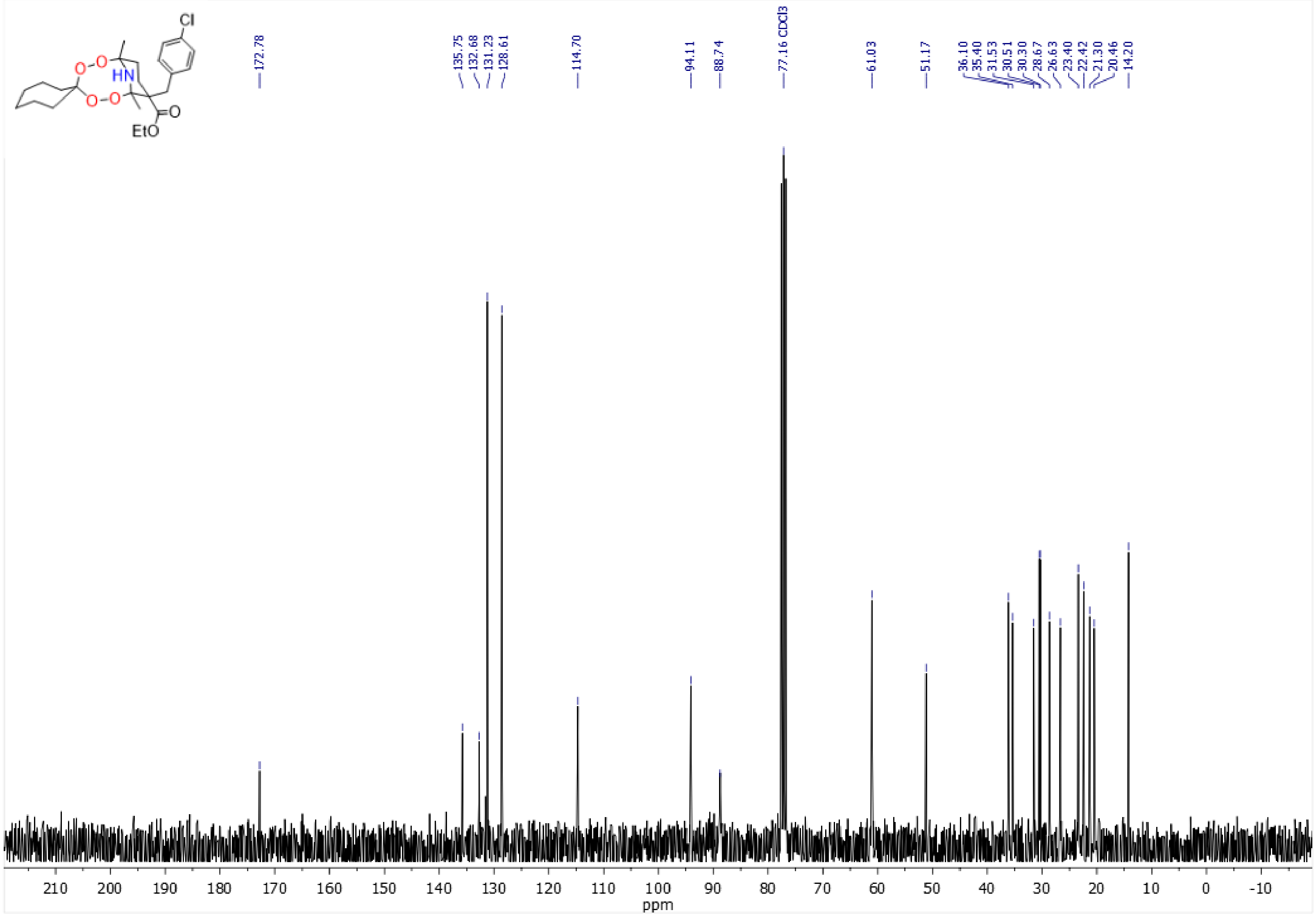

^{1}H NMR (300.13 MHz, $CDCl_3$). (1*S,1'*R**,2*S**,5*R**,7'*S**)-1',7'-dimethyl-2',3',5',6'-tetraoxa-11'-azaspiro[adamantane-2,4'-bicyclo[5.3.1]undecane], 3ac**

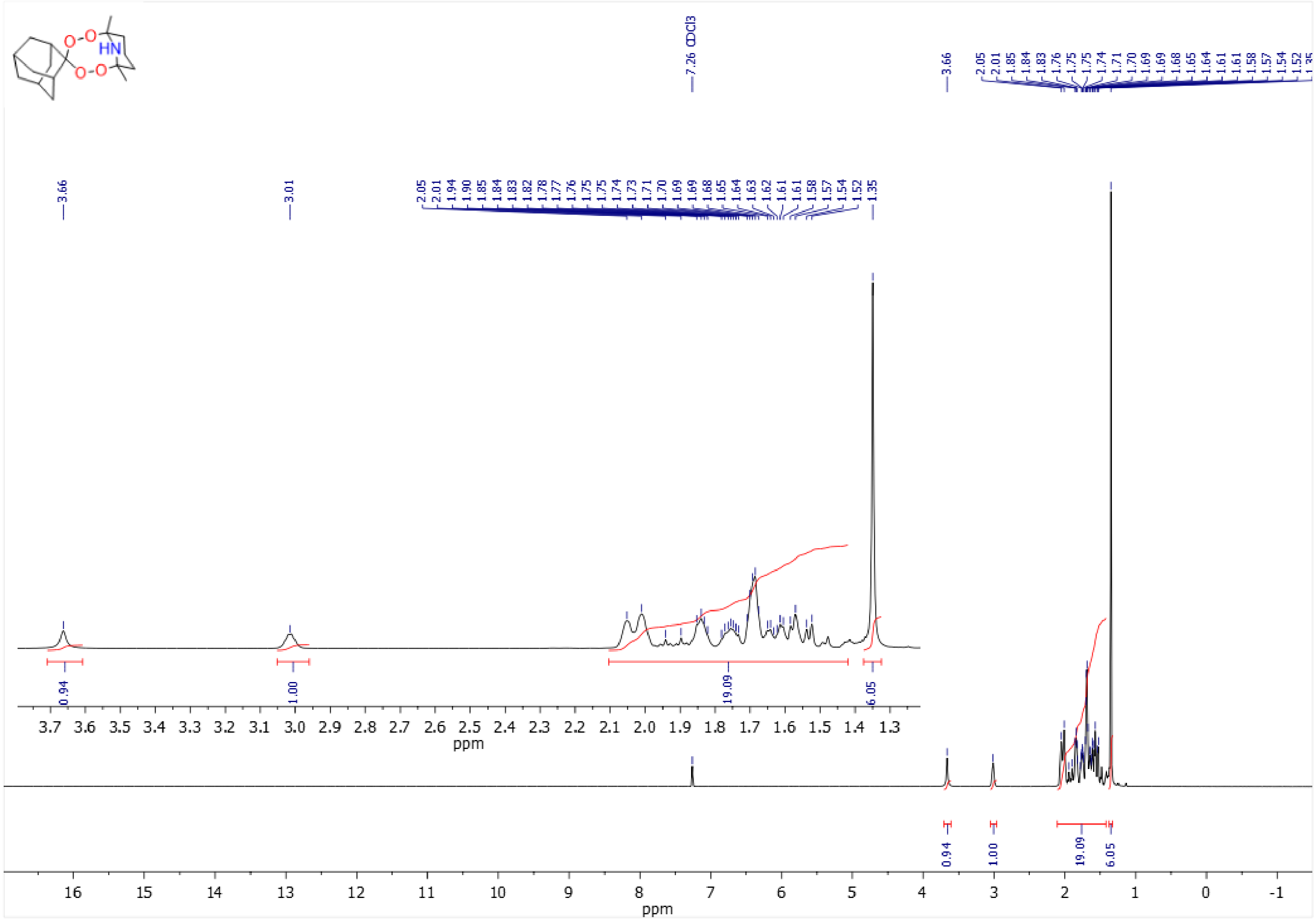

^{13}C NMR (75.48 MHz, $CDCl_3$). (1*S,1'*R**,2*S**,5*R**,7'*S**)-1',7'-dimethyl-2',3',5',6'-tetraoxa-11'-azaspiro[adamantane-2,4'-bicyclo[5.3.1]undecane], 3ac**

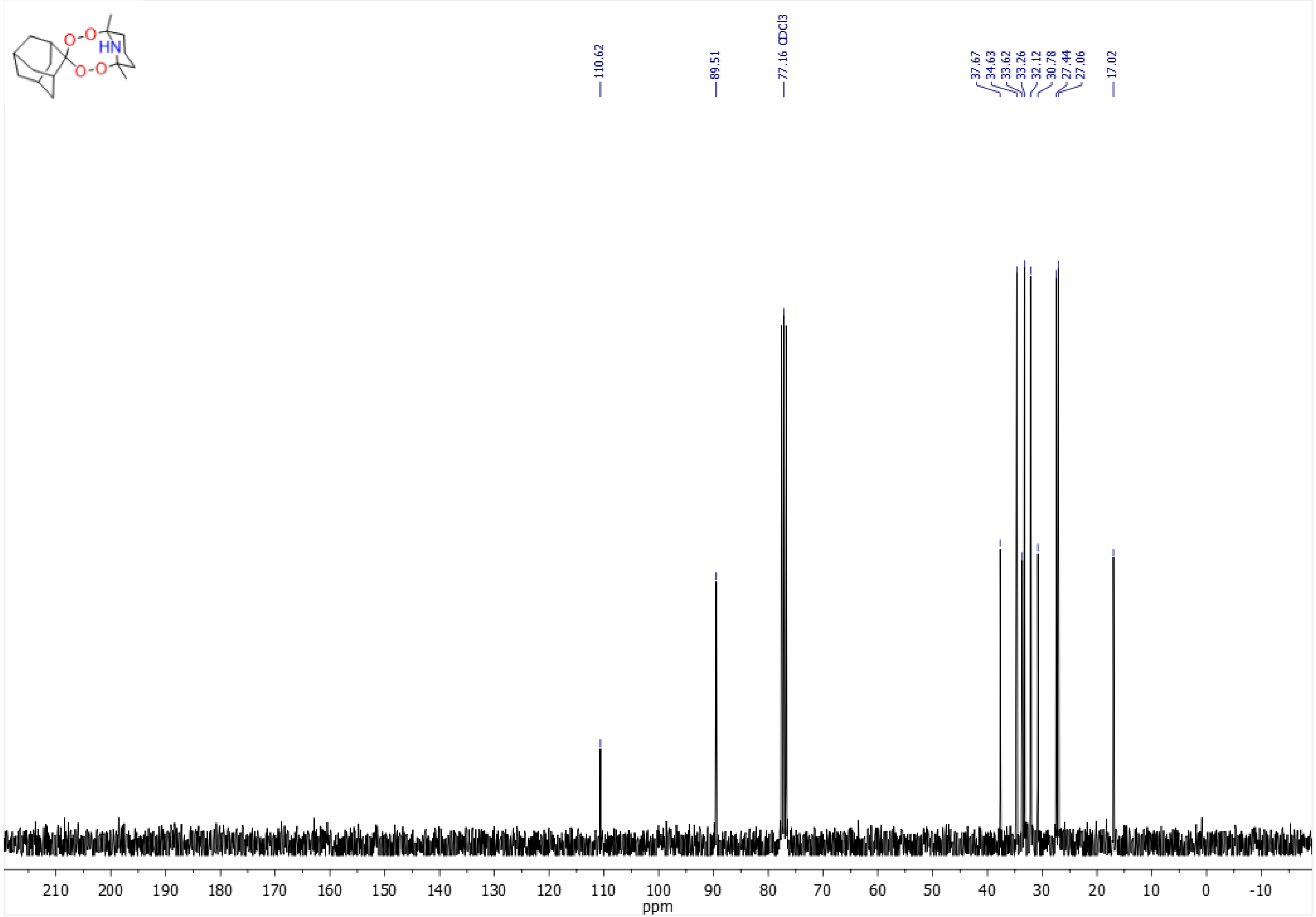

^{1}H NMR (300.13 MHz, CDCl$_3$). Ethyl (1*S,1'*R**,2*R**,5*R**,7'*S**,8'*S**)-1',7',8'-trimethyl-2',3',5',6'-tetraoxa-11'-azaspiro[adamantane-2,4'-bicyclo[5.3.1]undecane]-8'-carboxylate, 3bc**

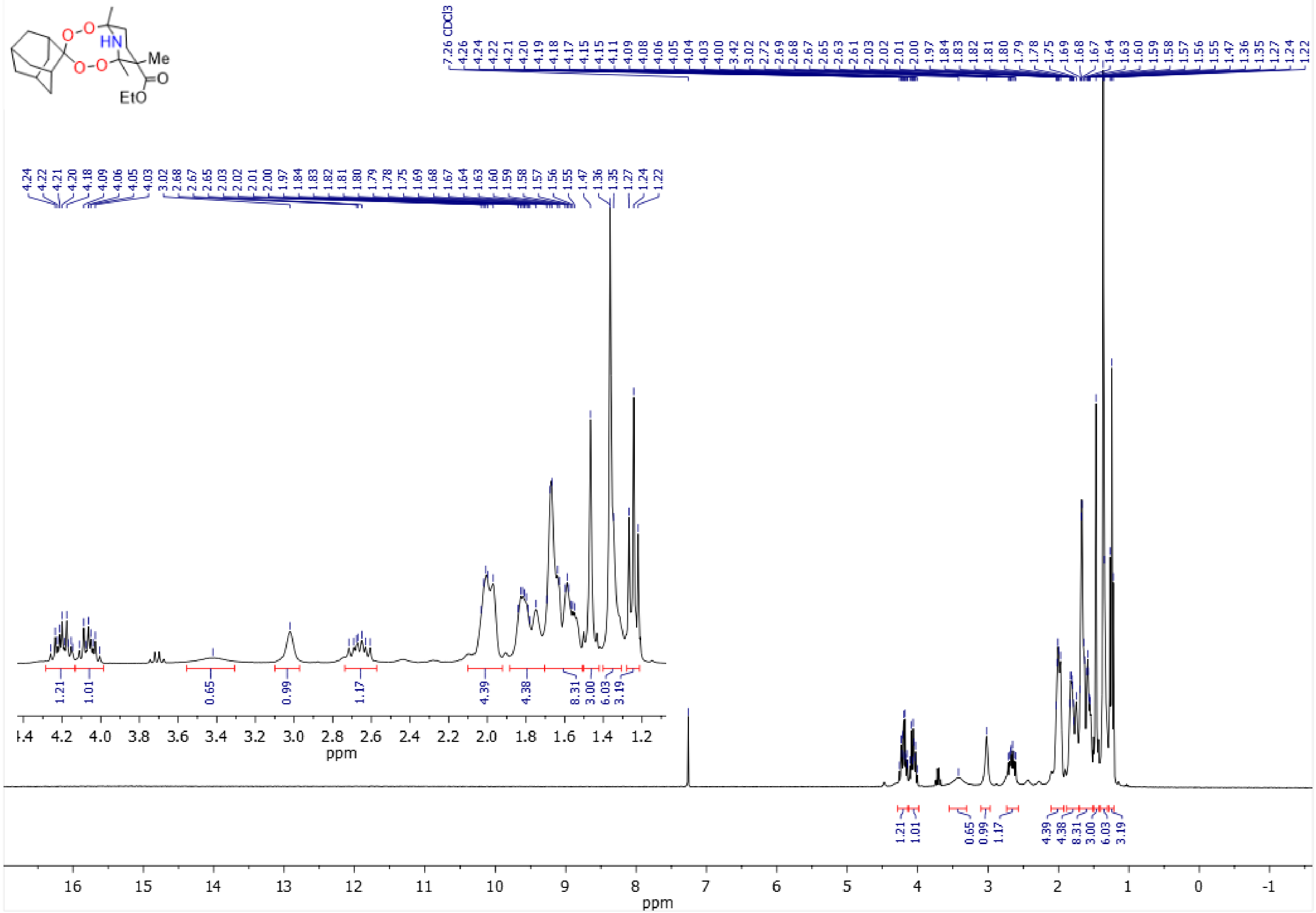

^{13}C NMR (75.48 MHz, CDCl$_3$). Ethyl (1*S,1'*R**,2*R**,5*R**,7'*S**,8'*S**)-1',7',8'-trimethyl-2',3',5',6'-tetraoxa-11'-azaspiro[adamantane-2,4'-bicyclo[5.3.1]undecane]-8'-carboxylate, 3bc**

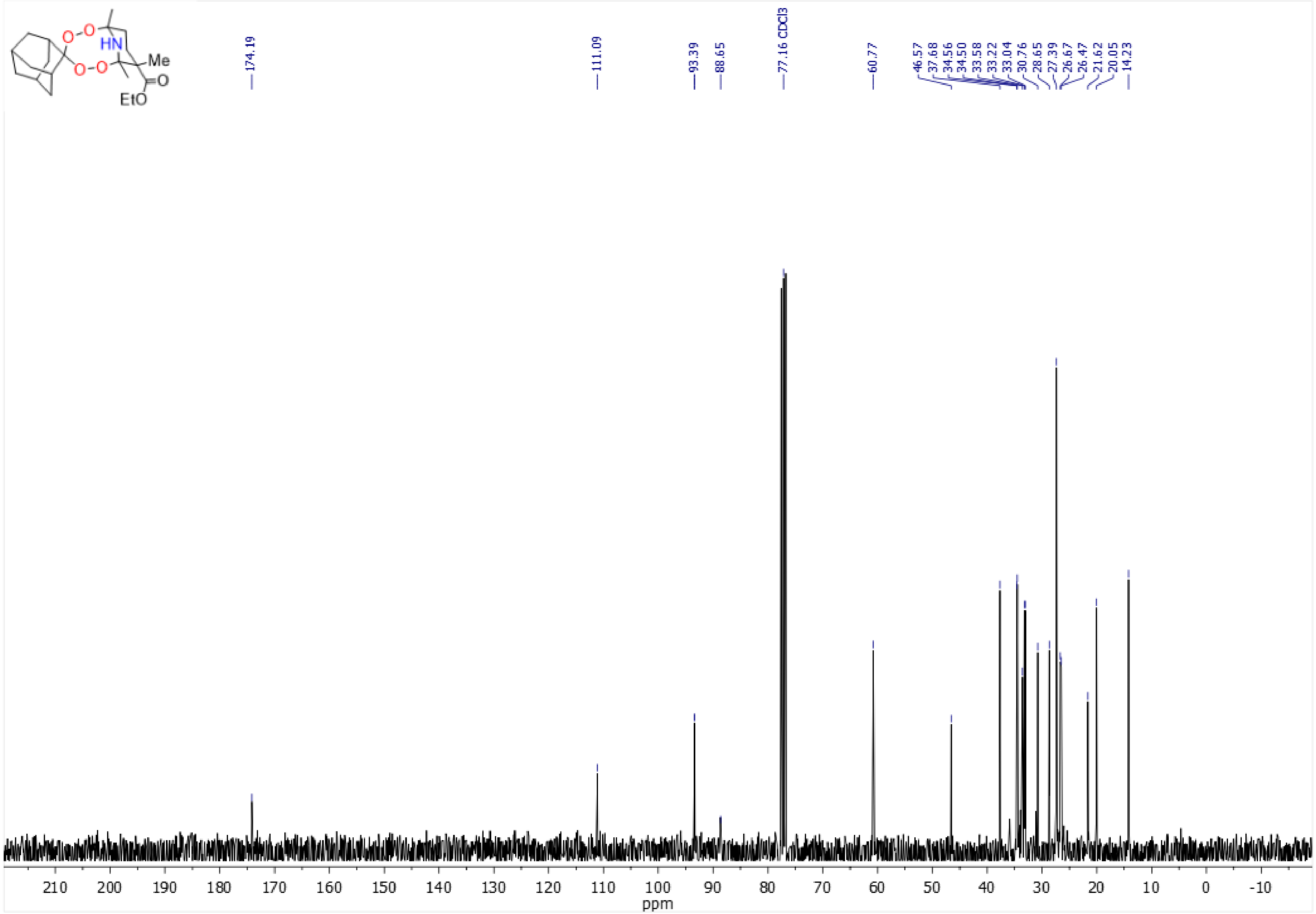

^{1}H NMR (300.13 MHz, CDCl3). Ethyl (1*S,1'*R**,2*R**,5*R**,7'*S**,8'*S**)-8'-ethyl-1',7'-dimethyl-2',3',5',6'-tetraoxa-11'-azaspiro[adamantane-2,4'-bicyclo[5.3.1]undecane]-8'-carboxylate, 3cc**

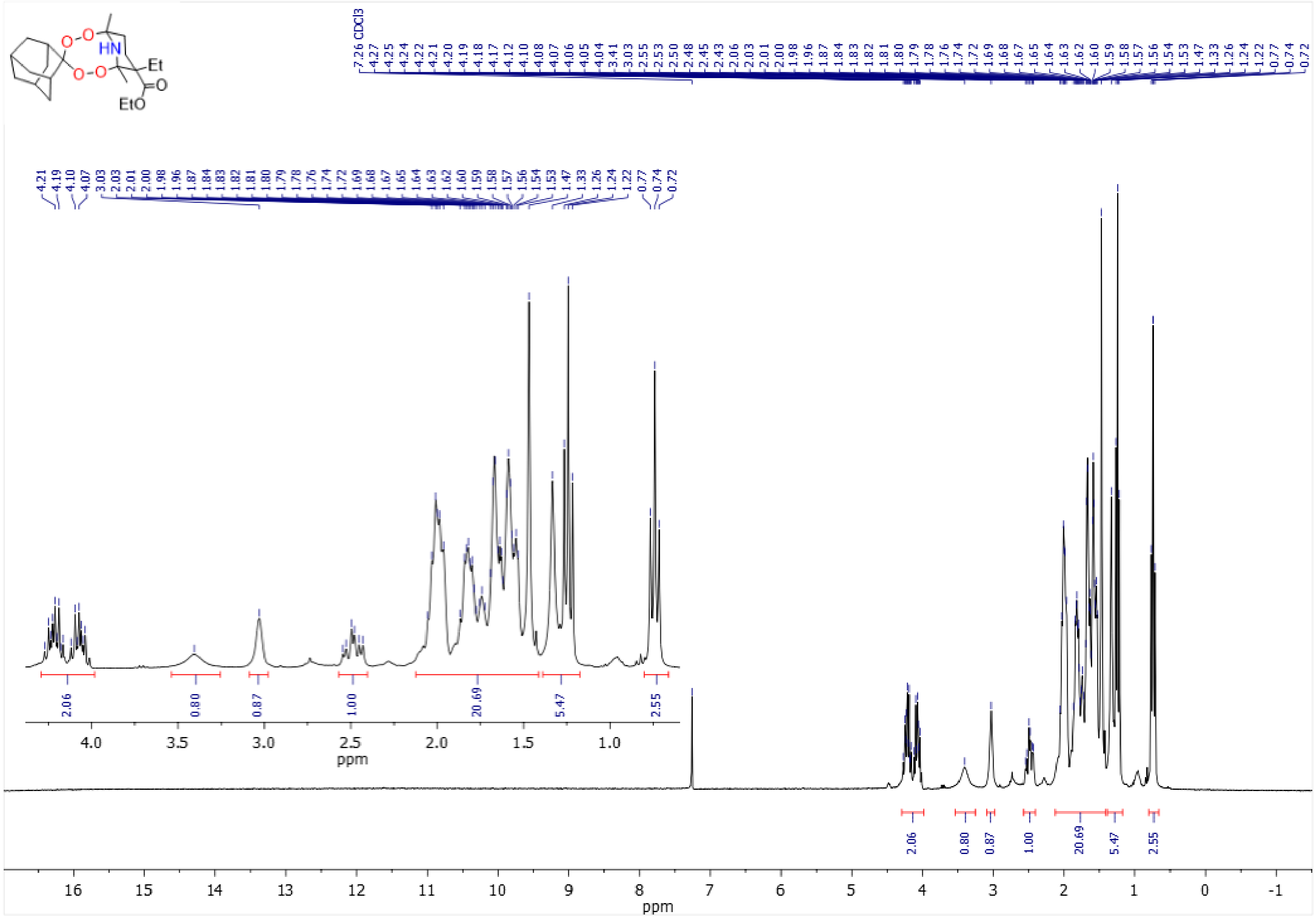

^{13}C NMR (75.48 MHz, $CDCl_3$). Ethyl (1*S,1'*R**,2*R**,5*R**,7'*S**,8'*S**)-8'-ethyl-1',7'-dimethyl-2',3',5',6'-tetraoxa-11'-azaspiro[adamantane-2,4'-bicyclo[5.3.1]undecane]-8'-carboxylate, 3cc**

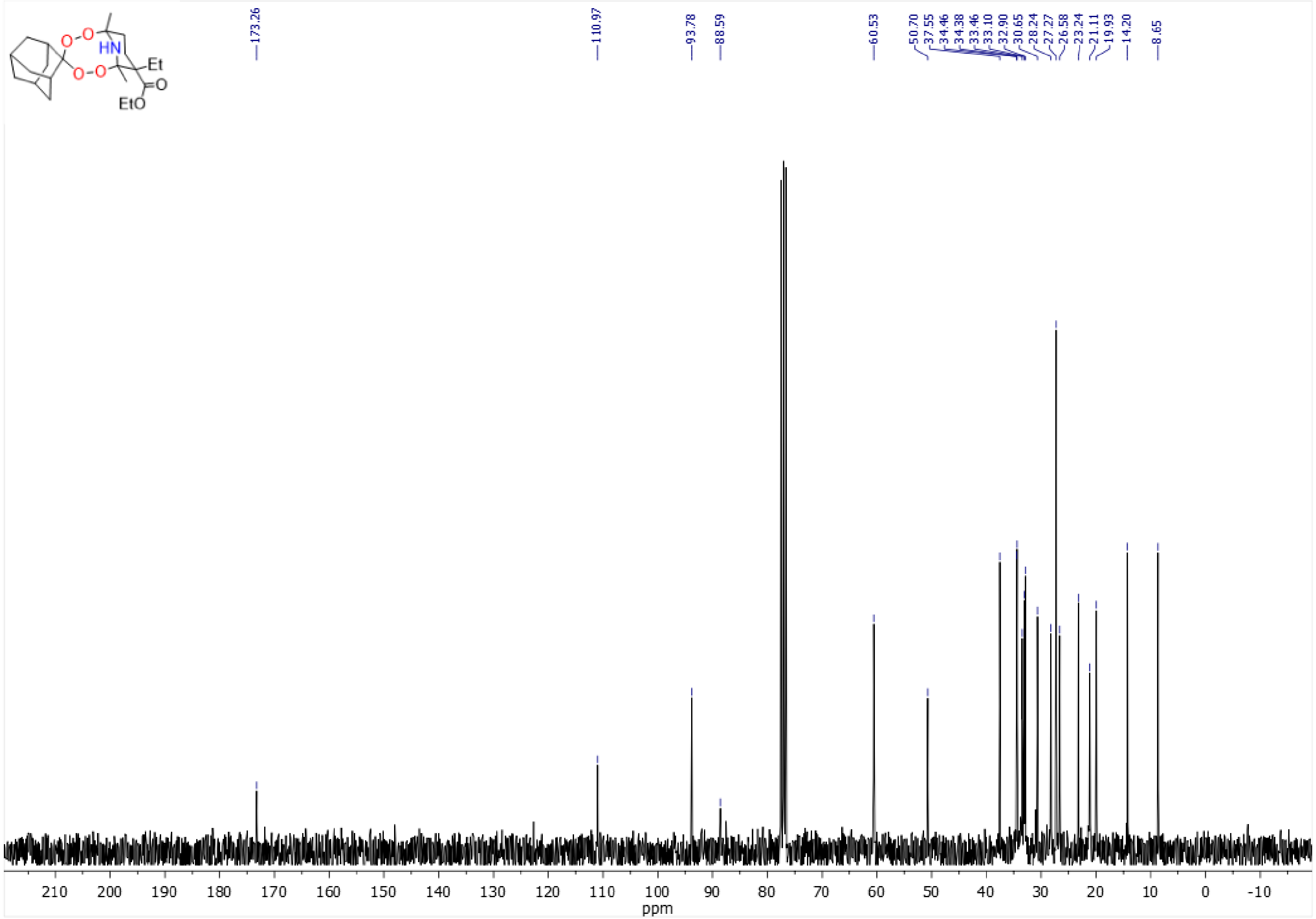

^{1}H NMR (300.13 MHz, $CDCl_3$). Ethyl (1*S**,1'*R**,2*R**,5*R**,7'*S**,8'*S**)-8'-butyl-1',7'-dimethyl-2',3',5',6'-tetraoxa-11'-azaspiro[adamantane-2,4'-bicyclo[5.3.1]undecane]-8'-carboxylate, 3dc

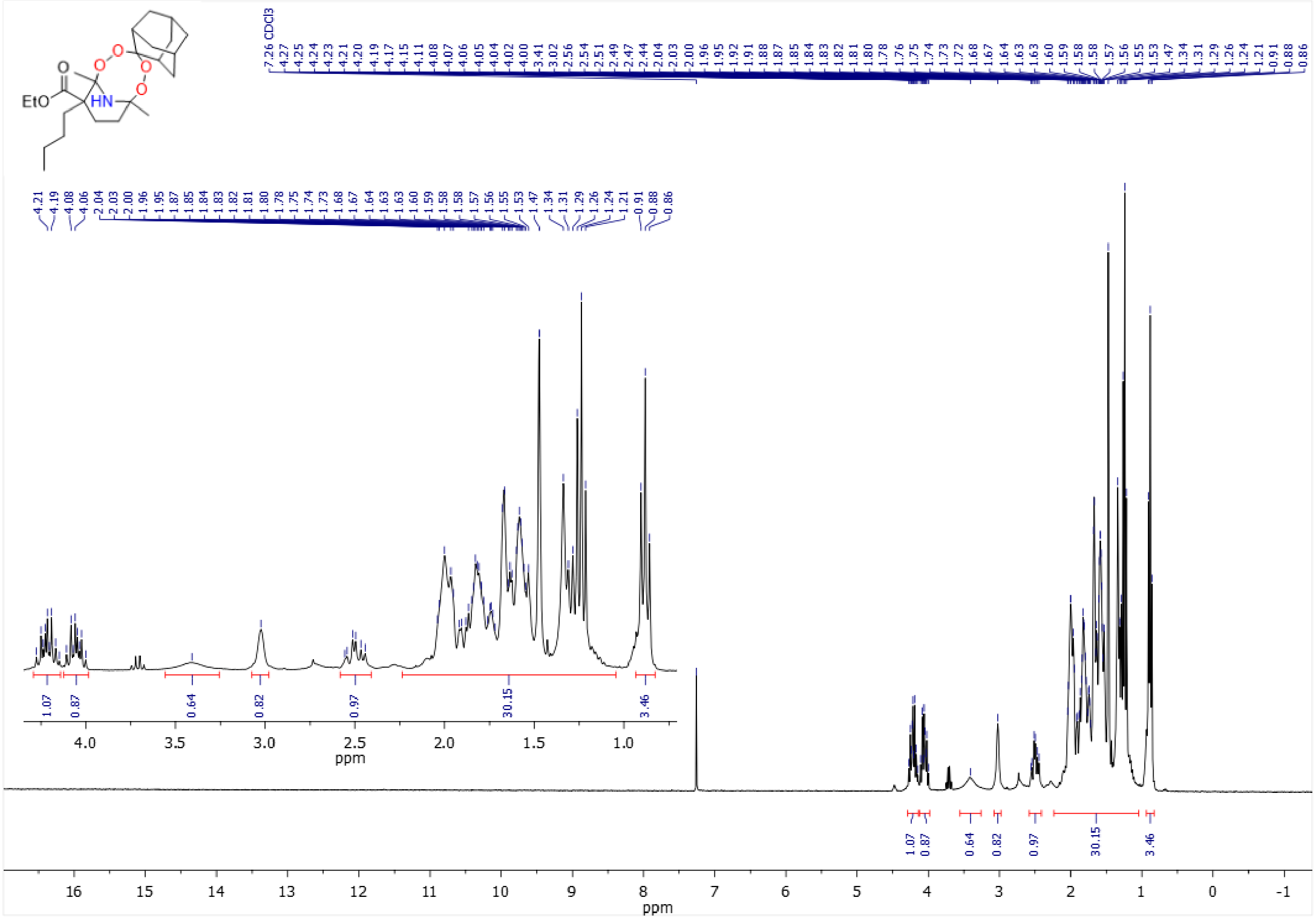

^{13}C NMR (75.48 MHz, $CDCl_3$). Ethyl (1*S,1'*R**,2*R**,5*R**,7'*S**,8'*S**)-8'-butyl-1',7'-dimethyl-2',3',5',6'-tetraoxa-11'-azaspiro[adamantane-2,4'-bicyclo[5.3.1]undecane]-8'-carboxylate, 3dc**

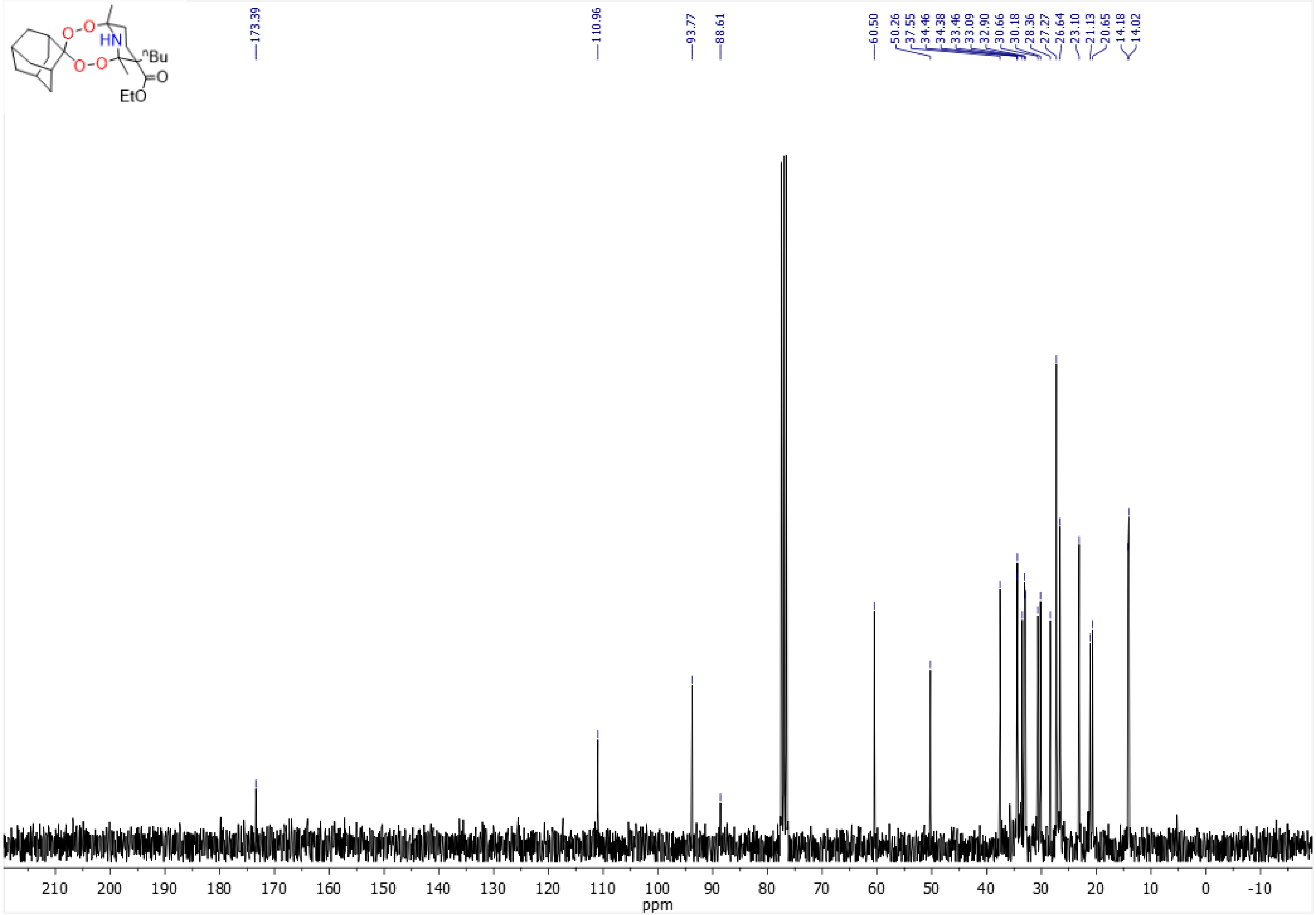

^{1}H NMR (300.13 MHz, $CDCl_3$). Ethyl (1*S**,1'*R**,2*R**,5*R**,7'*S**,8'*R**)-8'-(3-ethoxy-3-oxopropyl)-1',7'-dimethyl-2',3',5',6'-tetraoxa-11'-azaspiro[adamantane-2,4'-bicyclo[5.3.1]undecane]-8'-carboxylate, 3ec

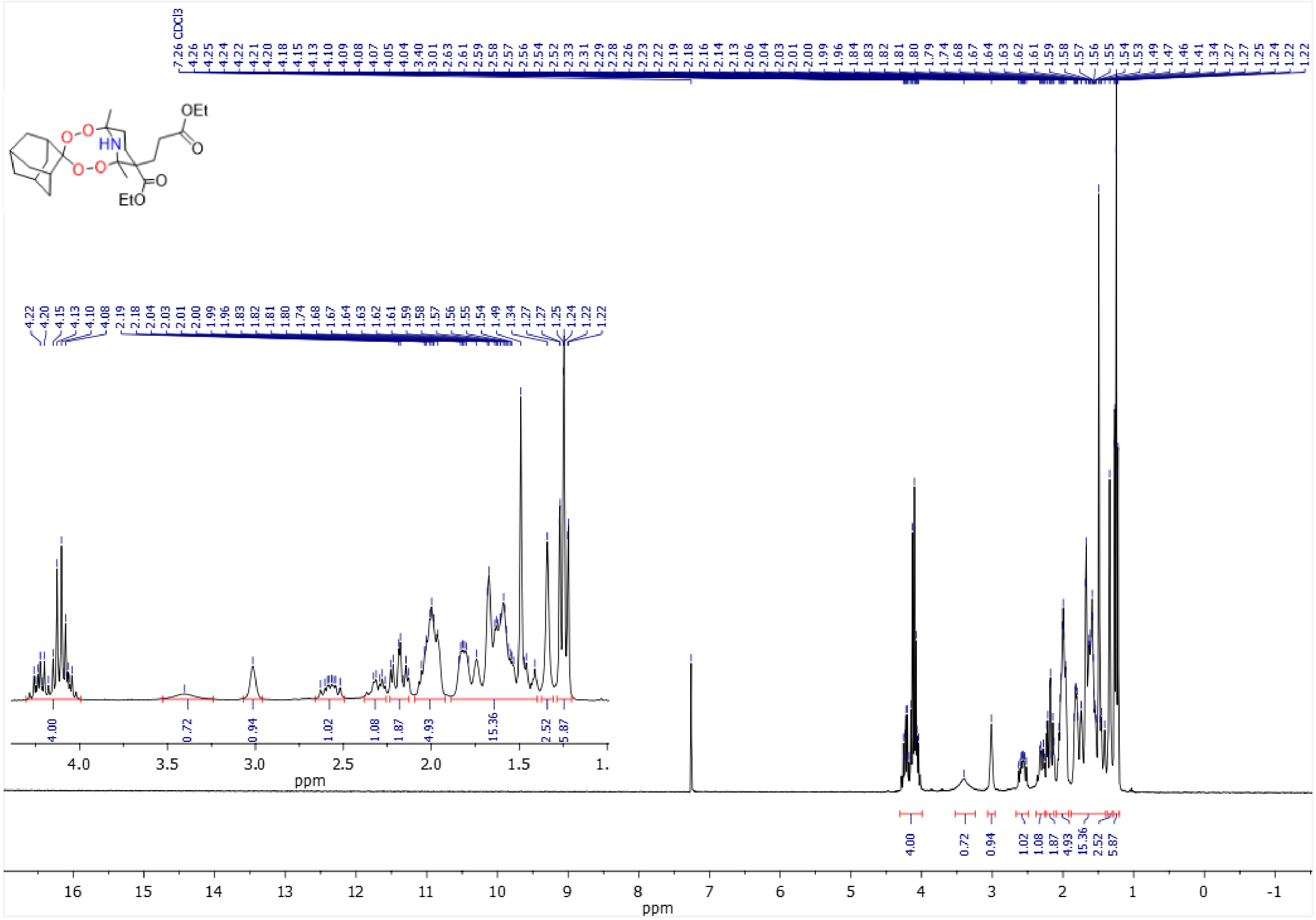

^{13}C NMR (75.48 MHz, CDCl$_3$). Ethyl (1*S,1'*R**,2*R**,5*R**,7'*S**,8'*R**)-8'-(3-ethoxy-3-oxopropyl)-1',7'-dimethyl-2',3',5',6'-tetraoxa-11'-azaspiro[adamantane-2,4'-bicyclo[5.3.1]undecane]-8'-carboxylate, 3ec**

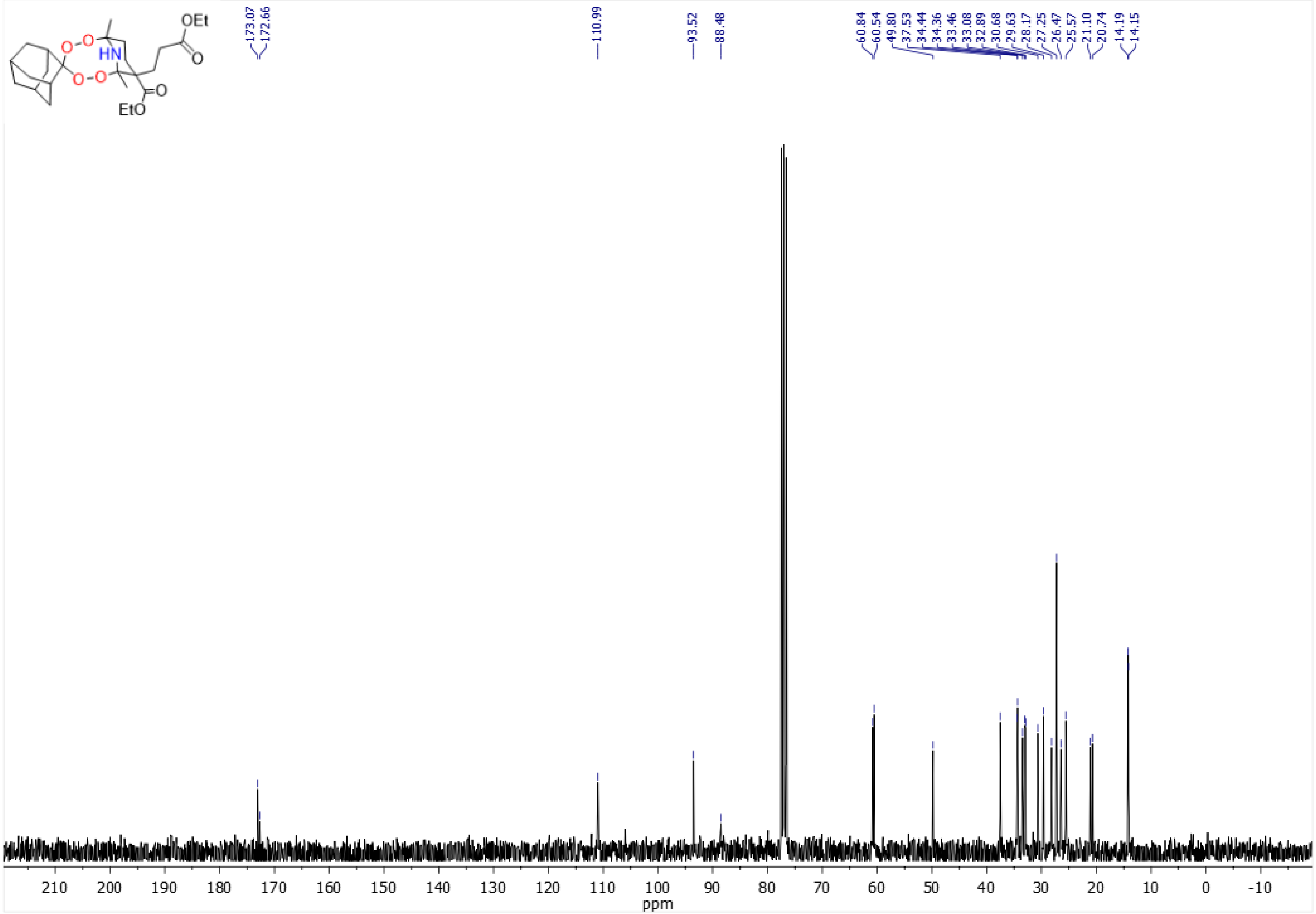

^{1}H NMR (300.13 MHz, CDCl$_3$). Ethyl (1*S,1'*R**,2*R**,5*R**,7'*S**,8'*R**)-8'-allyl-1',7'-dimethyl-2',3',5',6'-tetraoxa-11'-azaspiro[adamantane-2,4'-bicyclo[5.3.1]undecane]-8'-carboxylate, 3fc**

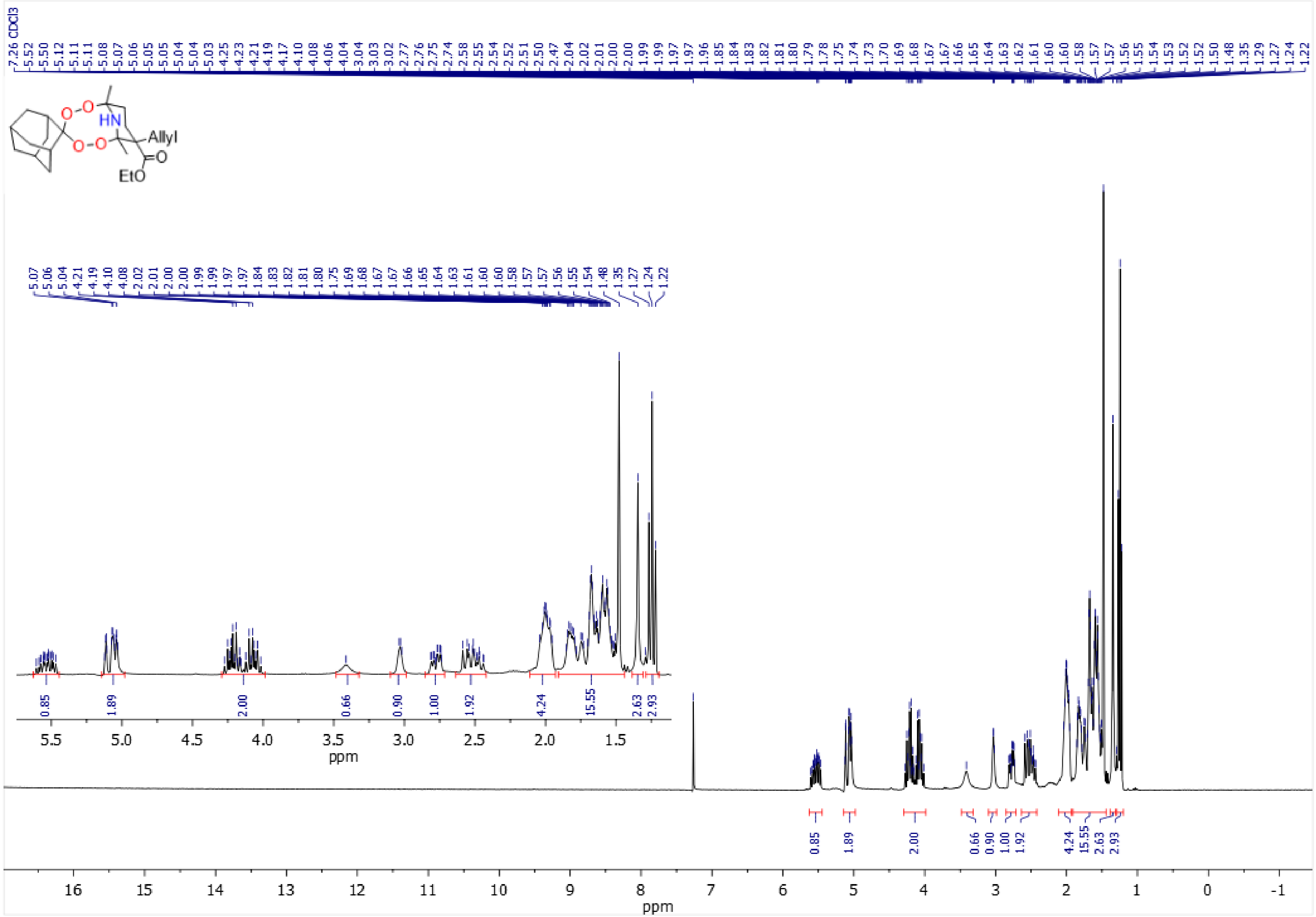

^{13}C NMR (75.48 MHz, $CDCl_3$). Ethyl (1*S,1'*R**,2*R**,5*R**,7'*S**,8'*R**)-8'-allyl-1',7'-dimethyl-2',3',5',6'-tetraoxa-11'-azaspiro[adamantane-2,4'-bicyclo[5.3.1]undecane]-8'-carboxylate, 3fc**

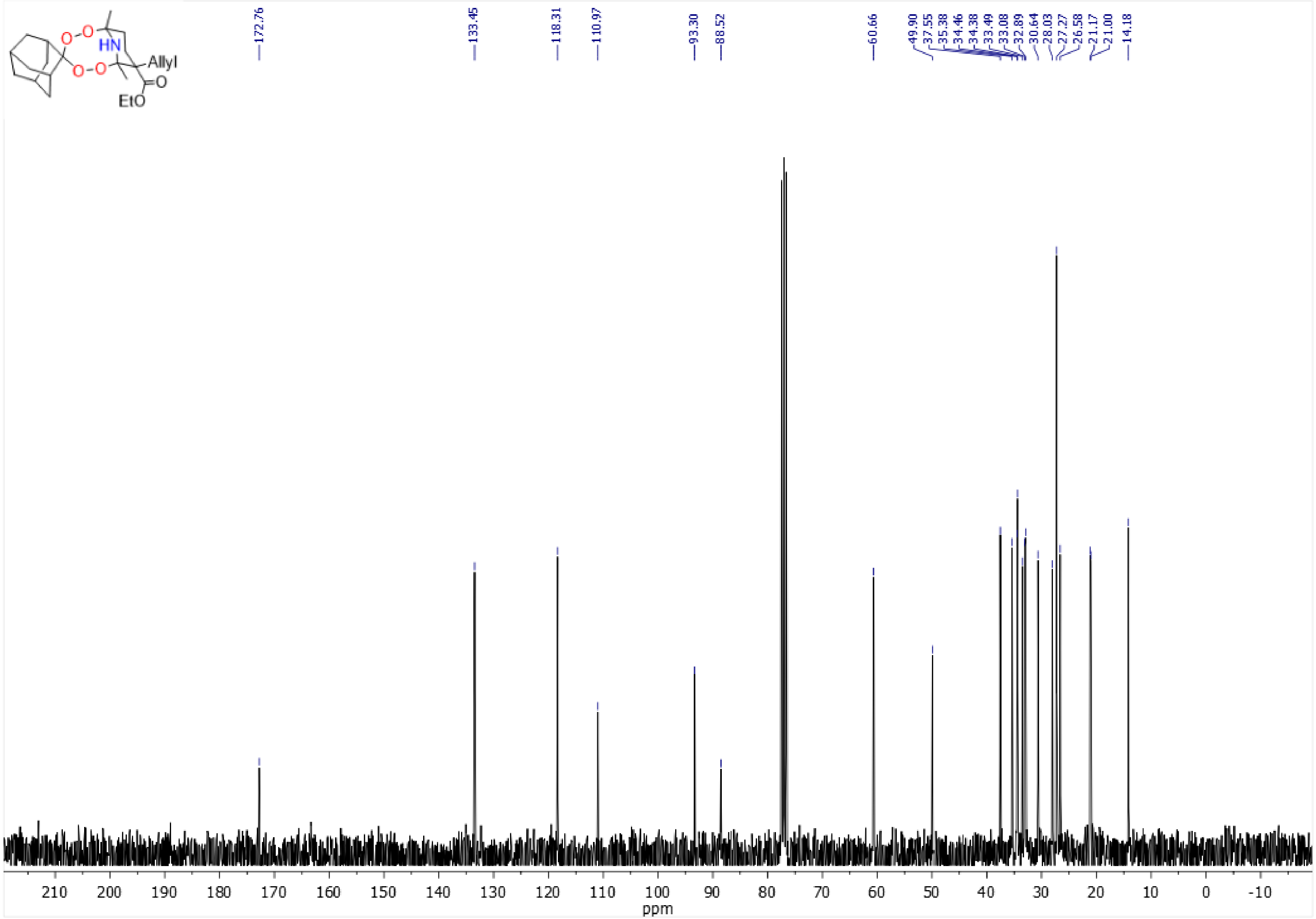

^{1}H NMR (300.13 MHz, CDCl$_3$). Ethyl (1*S,1'*R**,2*R**,5*R**,7'*S**,8'*R**)-1',7'-dimethyl-8'-(prop-2-yn-1-yl)-2',3',5',6'-tetraoxa-11'-azaspiro[adamantane-2,4'-bicyclo[5.3.1]undecane]-8'-carboxylate, 3gc**

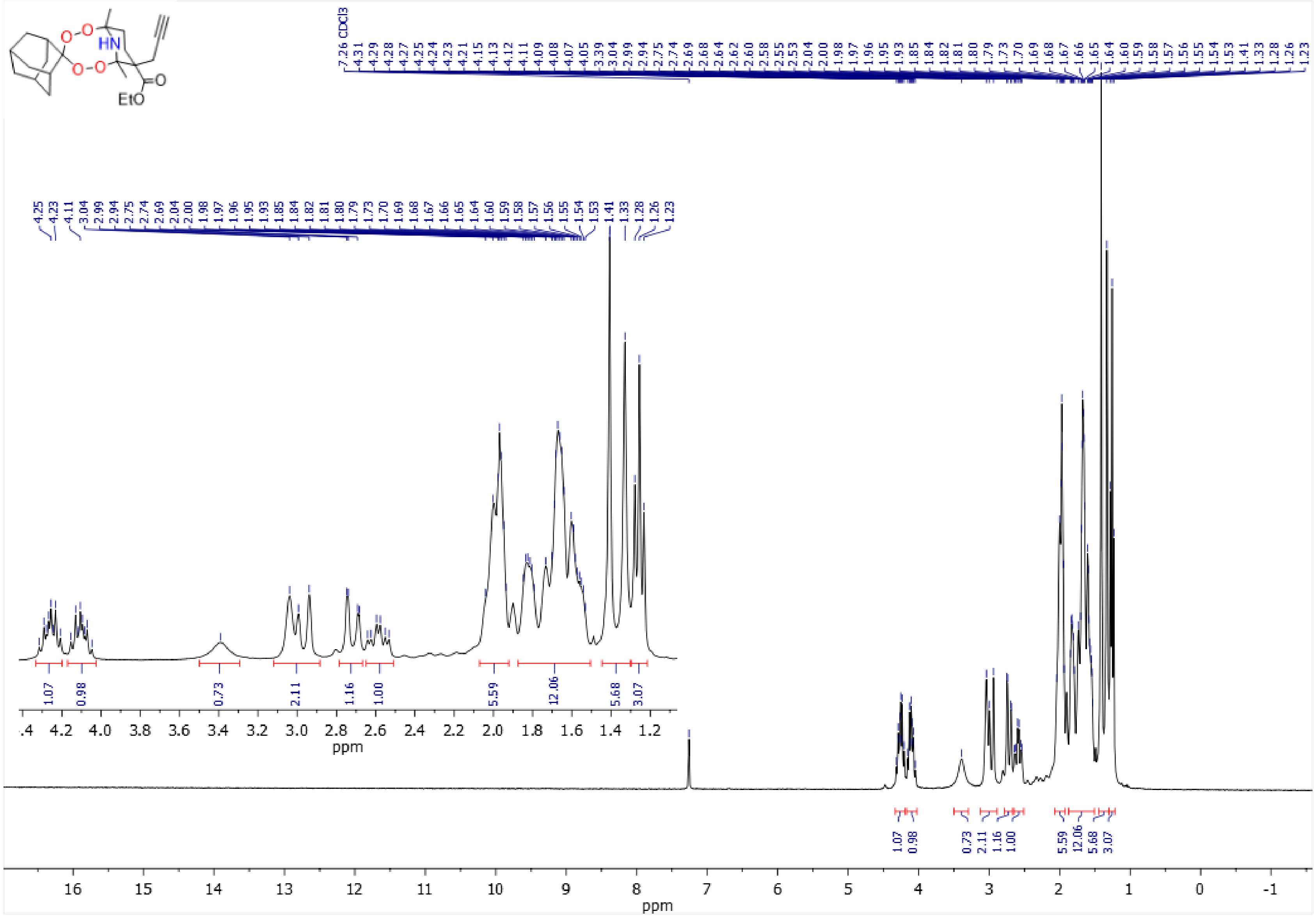

^{13}C NMR (75.48 MHz, CDCl$_3$). Ethyl (1*S,1'*R**,2*R**,5*R**,7'*S**,8'*R**)-1',7'-dimethyl-8'-(prop-2-yn-1-yl)-2',3',5',6'-tetraoxa-11'-azaspiro[adamantane-2,4'-bicyclo[5.3.1]undecane]-8'-carboxylate, 3gc**

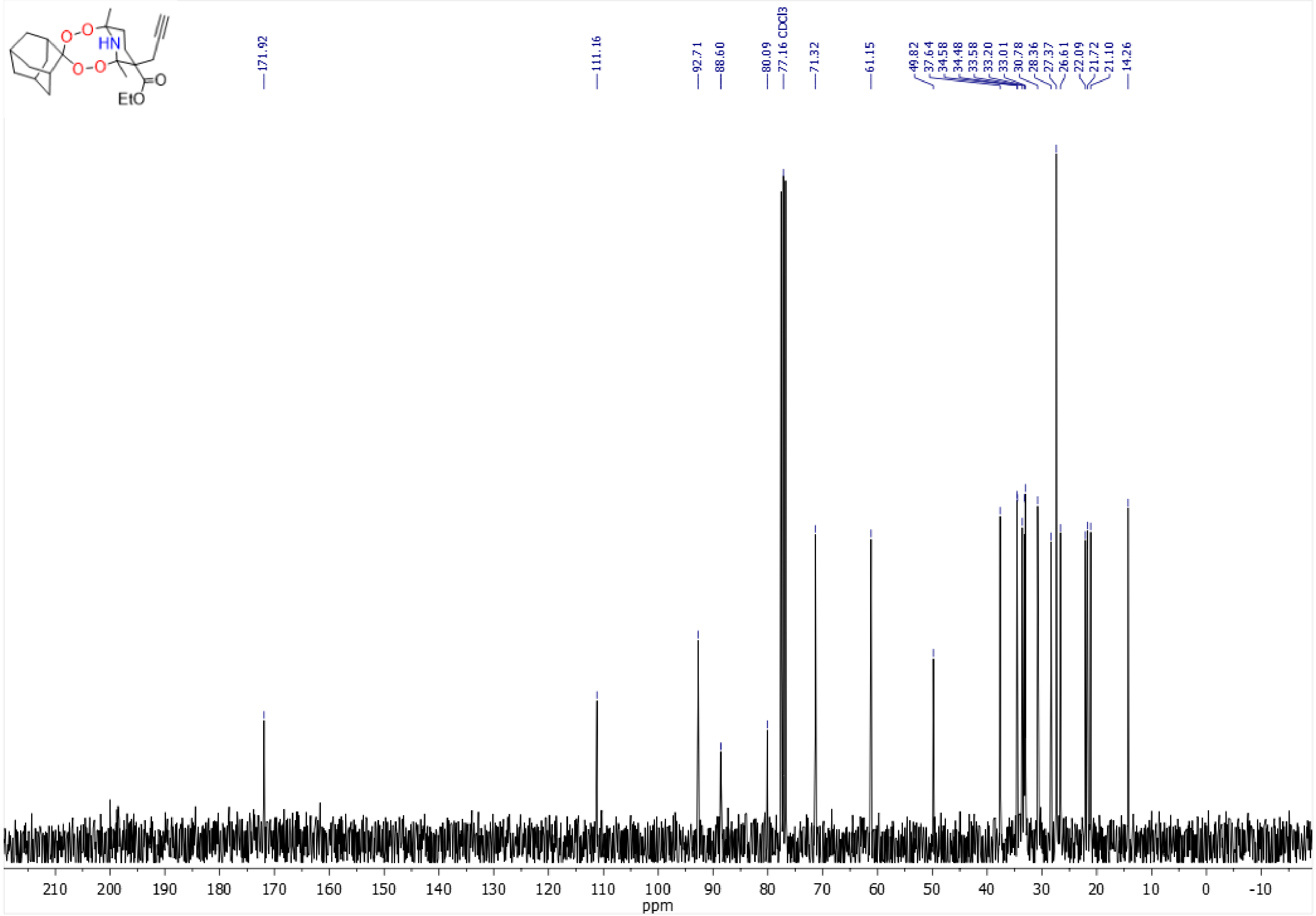

^{1}H NMR (300.13 MHz, $CDCl_3$). Ethyl (1*S,1'*R**,2*R**,5*R**,7'*S**,8'*R**)-8'-benzyl-1',7'-dimethyl-2',3',5',6'-tetraoxa-11'-azaspiro[adamantane-2,4'-bicyclo[5.3.1]undecane]-8'-carboxylate, 3hc**

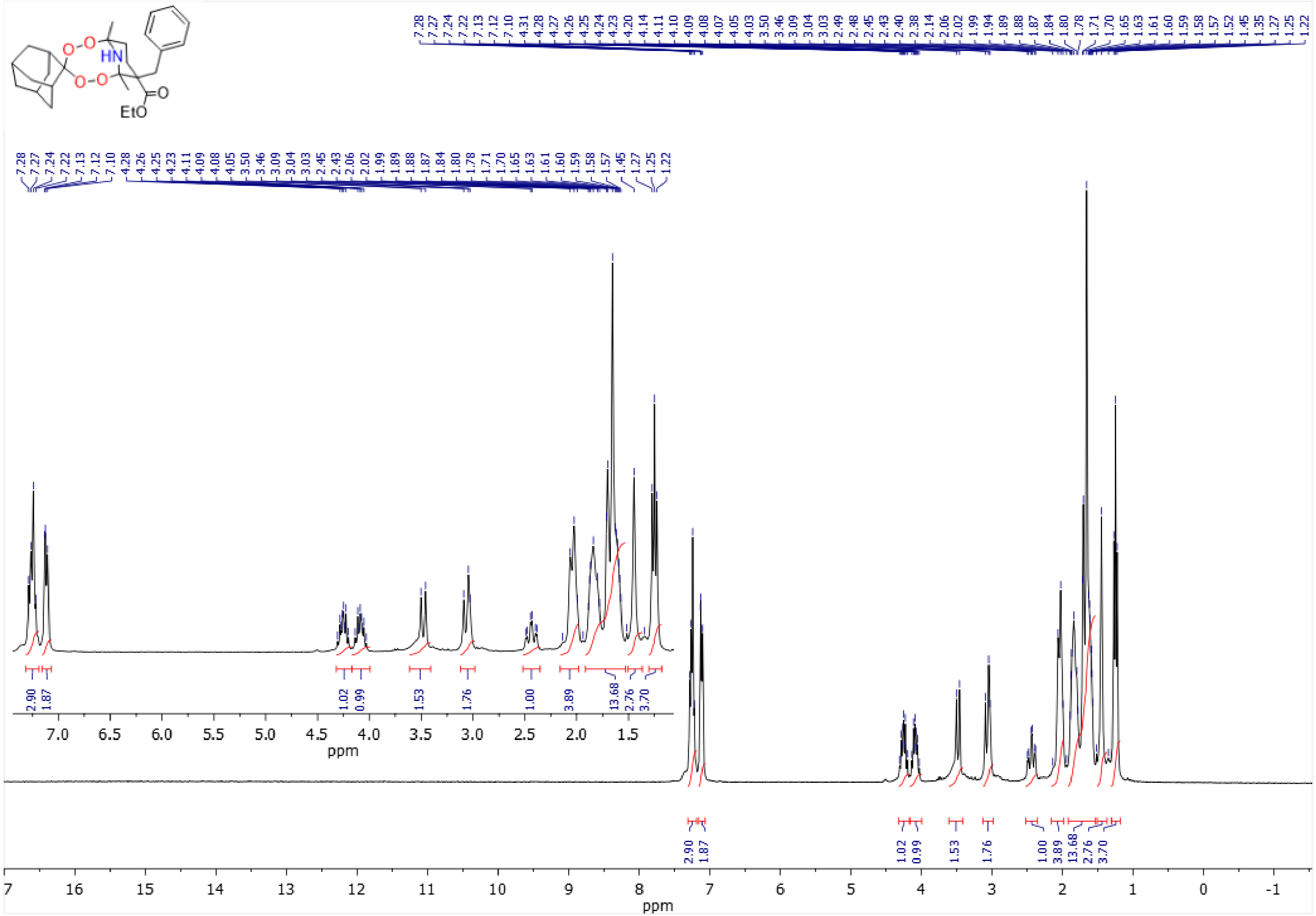

^{13}C NMR (75.48 MHz, CDCl$_3$). Ethyl (1*S,1'*R**,2*R**,5*R**,7'*S**,8'*R**)-8'-benzyl-1',7'-dimethyl-2',3',5',6'-tetraoxa-11'-azaspiro[adamantane-2,4'-bicyclo[5.3.1]undecane]-8'-carboxylate, 3hc**

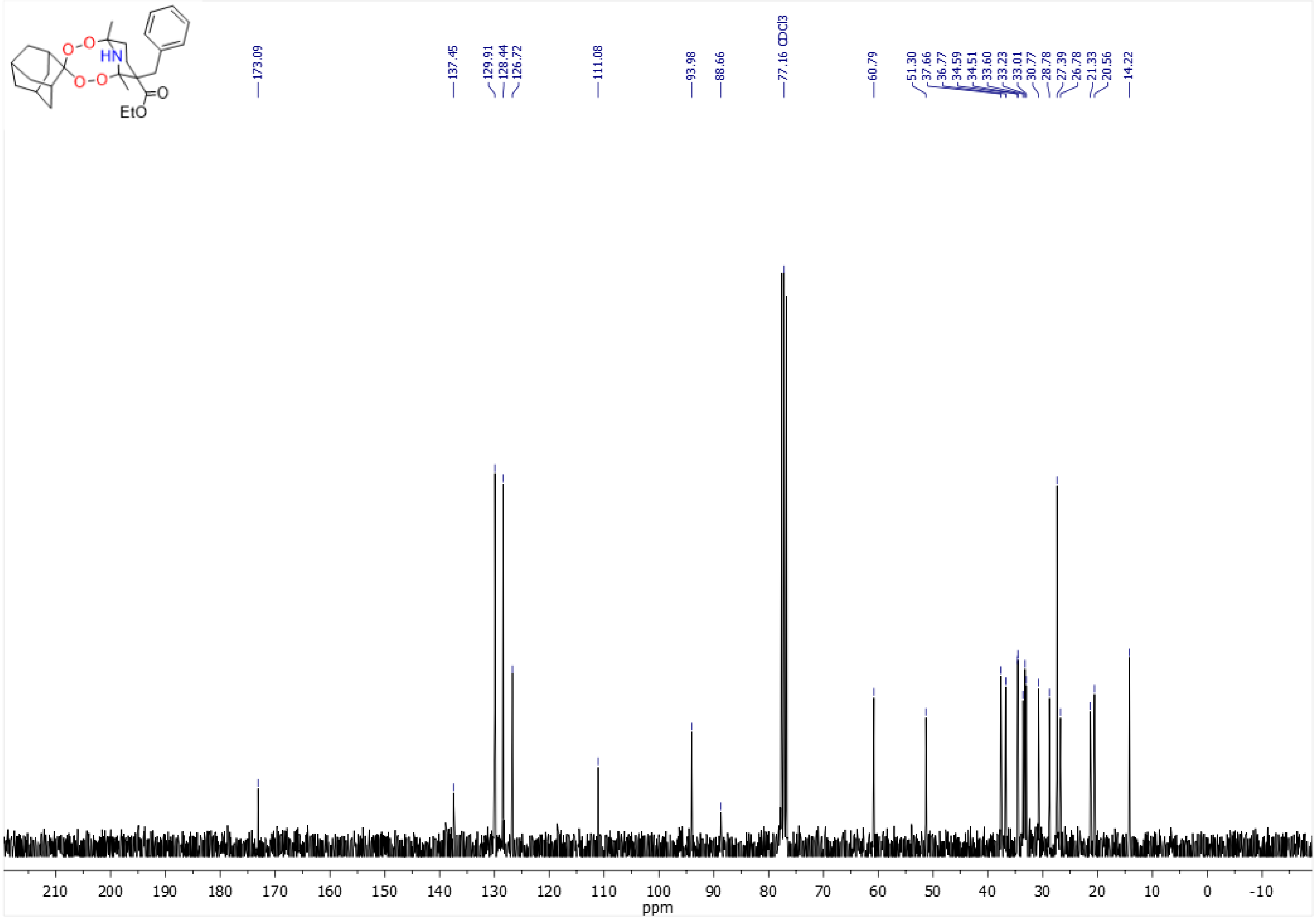

^{1}H NMR (300.13 MHz, CDCl$_3$). Ethyl (1*S,1'*R**,2*R**,5*R**,7'*S**,8'*R**)-8'-(4-(tert-butyl)benzyl)-1',7'-dimethyl-2',3',5',6'-tetraoxa-11'-azaspiro[adamantane-2,4'-bicyclo[5.3.1]undecane]-8'-carboxylate, 3jc**

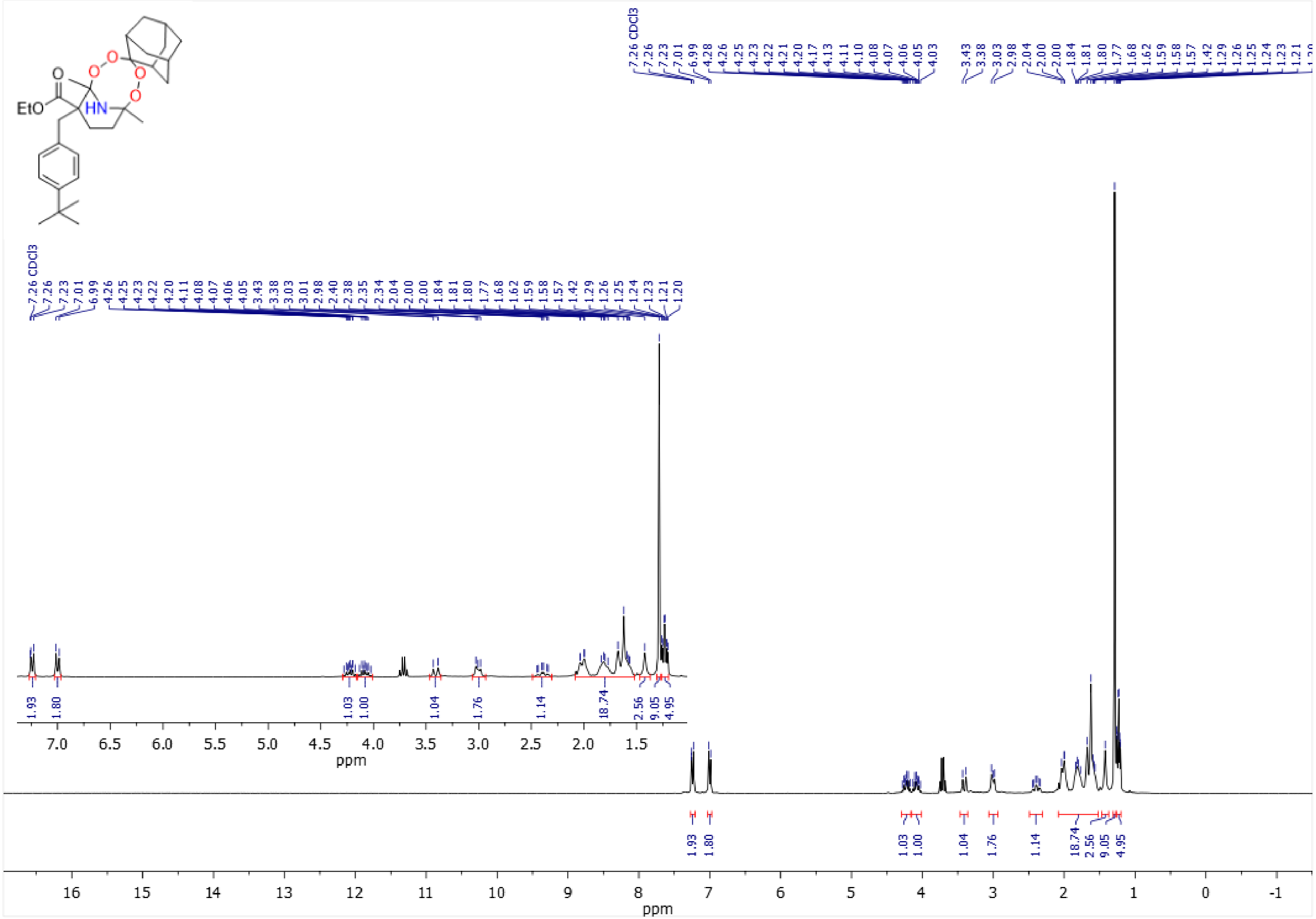

^{13}C NMR (75.48 MHz, CDCl$_3$). Ethyl (1*S,1'*R**,2*R**,5*R**,7'*S**,8'*R**)-8'-(4-(tert-butyl)benzyl)-1',7'-dimethyl-2',3',5',6'-tetraoxa-11'-azaspiro[adamantane-2,4'-bicyclo[5.3.1]undecane]-8'-carboxylate, 3jc**

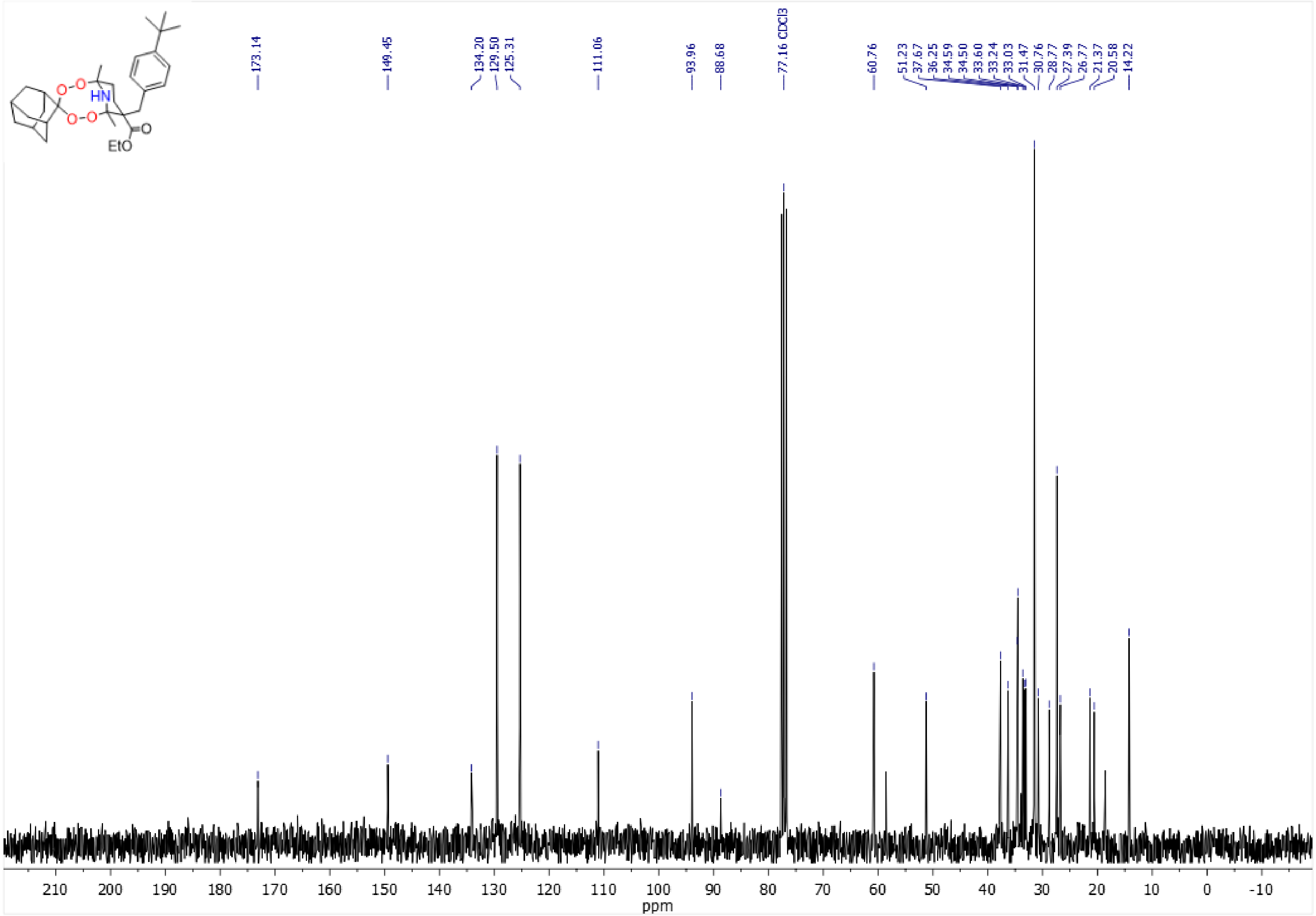

^{1}H NMR (300.13 MHz, $CDCl_3$). Ethyl (1*S,1'*R**,2*R**,5*R**,7'*S**,8'*R**)-8'-(4-fluorobenzyl)-1',7'-dimethyl-2',3',5',6'-tetraoxa-11'-azaspiro[adamantane-2,4'-bicyclo[5.3.1]undecane]-8'-carboxylate, 3kc**

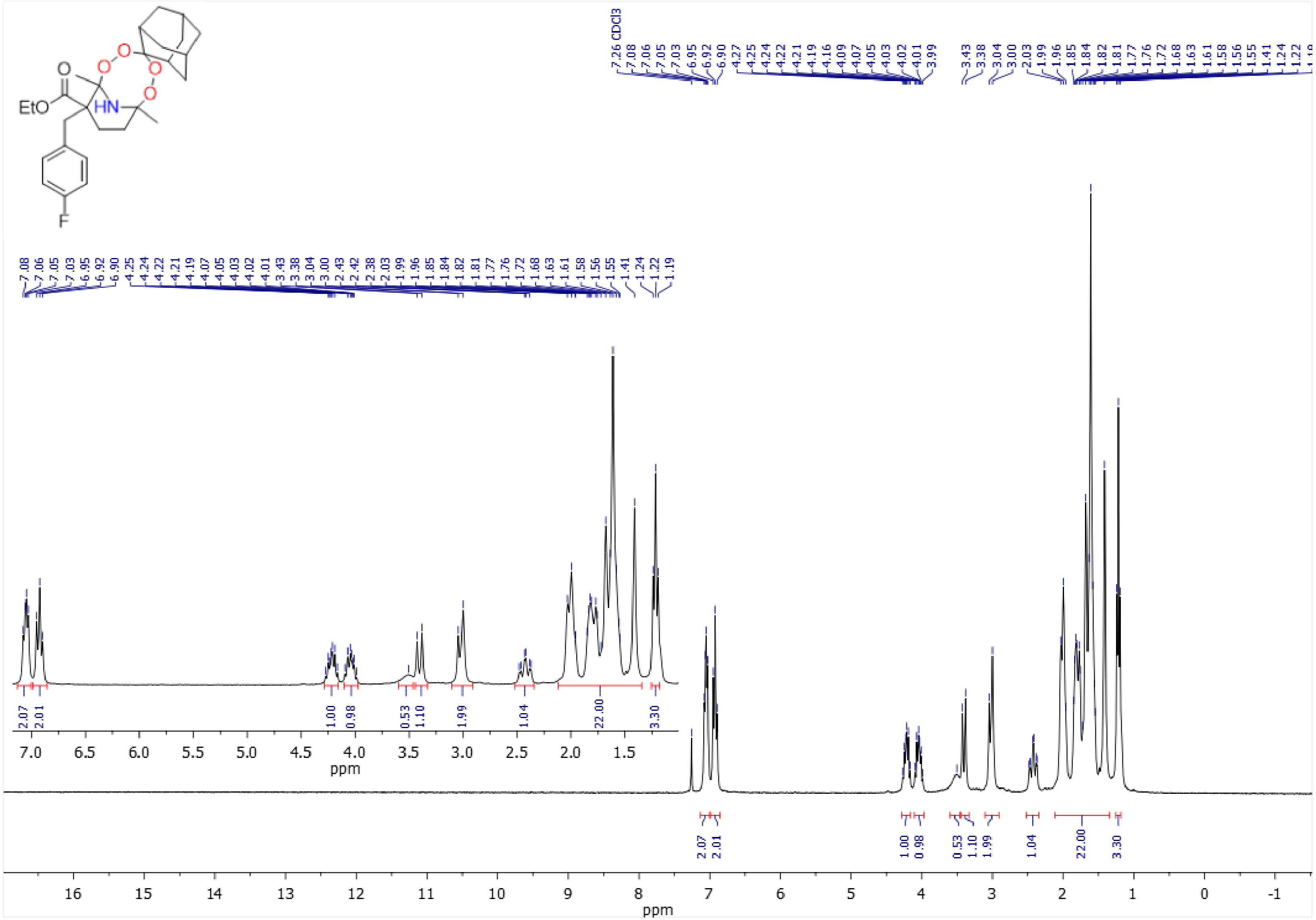

^{13}C NMR (75.48 MHz, CDCl$_3$). Ethyl (1*S,1'*R**,2*R**,5*R**,7'*S**,8'*R**)-8'-(4-fluorobenzyl)-1',7'-dimethyl-2',3',5',6'-tetraoxa-11'-azaspiro[adamantane-2,4'-bicyclo[5.3.1]undecane]-8'-carboxylate, 3kc**

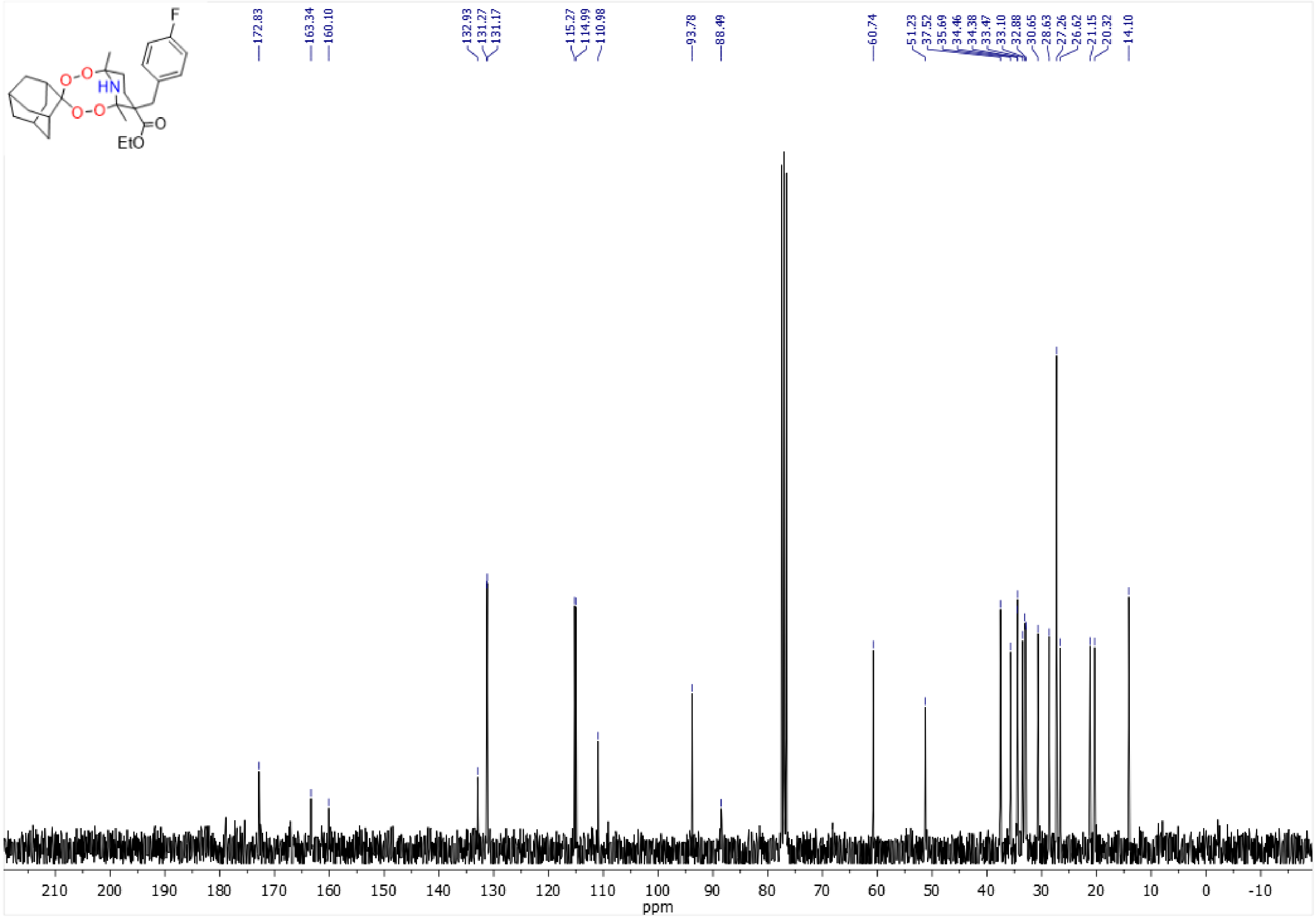

^{1}H NMR (300.13 MHz, CDCl$_3$). Ethyl (1*S,1'*R**,2*R**,5*R*,7'*S**,8'*R**)-8'-(4-chlorobenzyl)-1',7'-dimethyl-2',3',5',6'-tetraoxa-11'-azaspiro[adamantane-2,4'-bicyclo[5.3.1]undecane]-8'-carboxylate, 3lc**

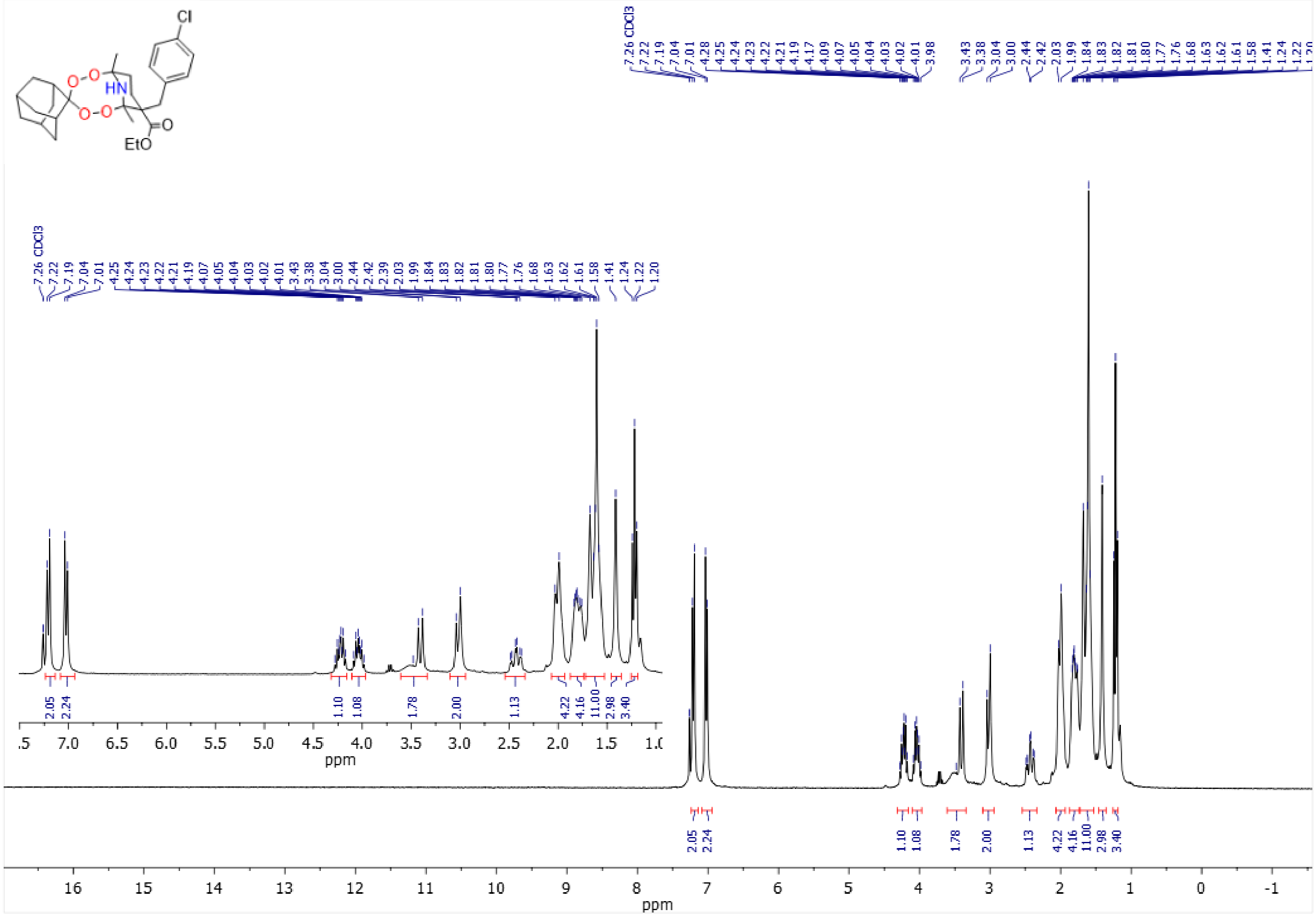

^{13}C NMR (75.48 MHz, CDCl$_3$). Ethyl (1*S,1'*R**,2*R**,5*R*,7'*S**,8'*R**)-8'-(4-chlorobenzyl)-1',7'-dimethyl-2',3',5',6'-tetraoxa-11'-azaspiro[adamantane-2,4'-bicyclo[5.3.1]undecane]-8'-carboxylate, 3lc**

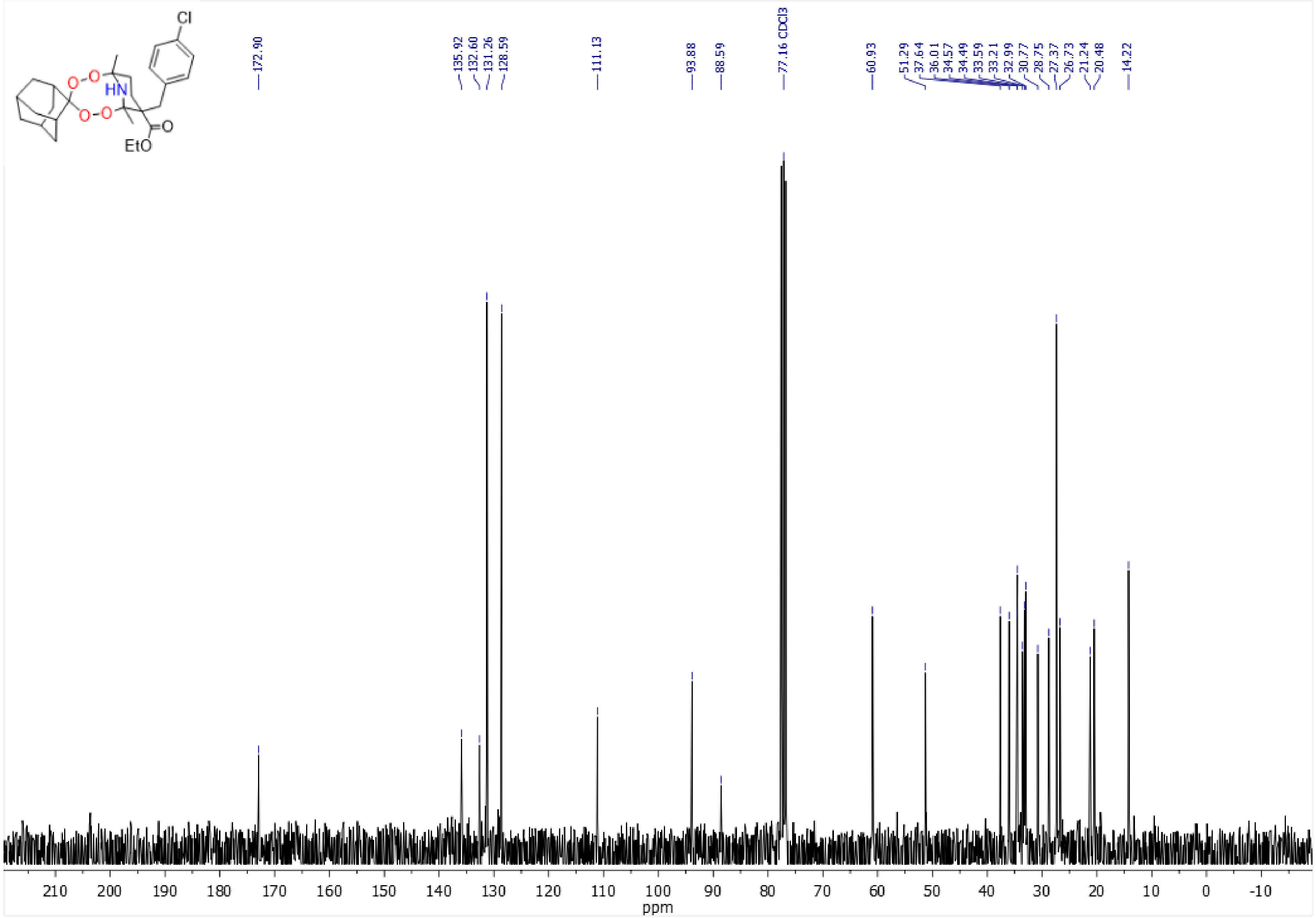

^{1}H NMR (300.13 MHz, CDCl₃). Allyl (1*S,1'*R**,2*R**,5*R**,7'*S**,8'*R**)-8'-allyl-1',7'-dimethyl-2',3',5',6'-tetraoxa-11'-azaspiro[adamantane-2,4'-bicyclo[5.3.1]undecane]-8'-carboxylate, 3mc**

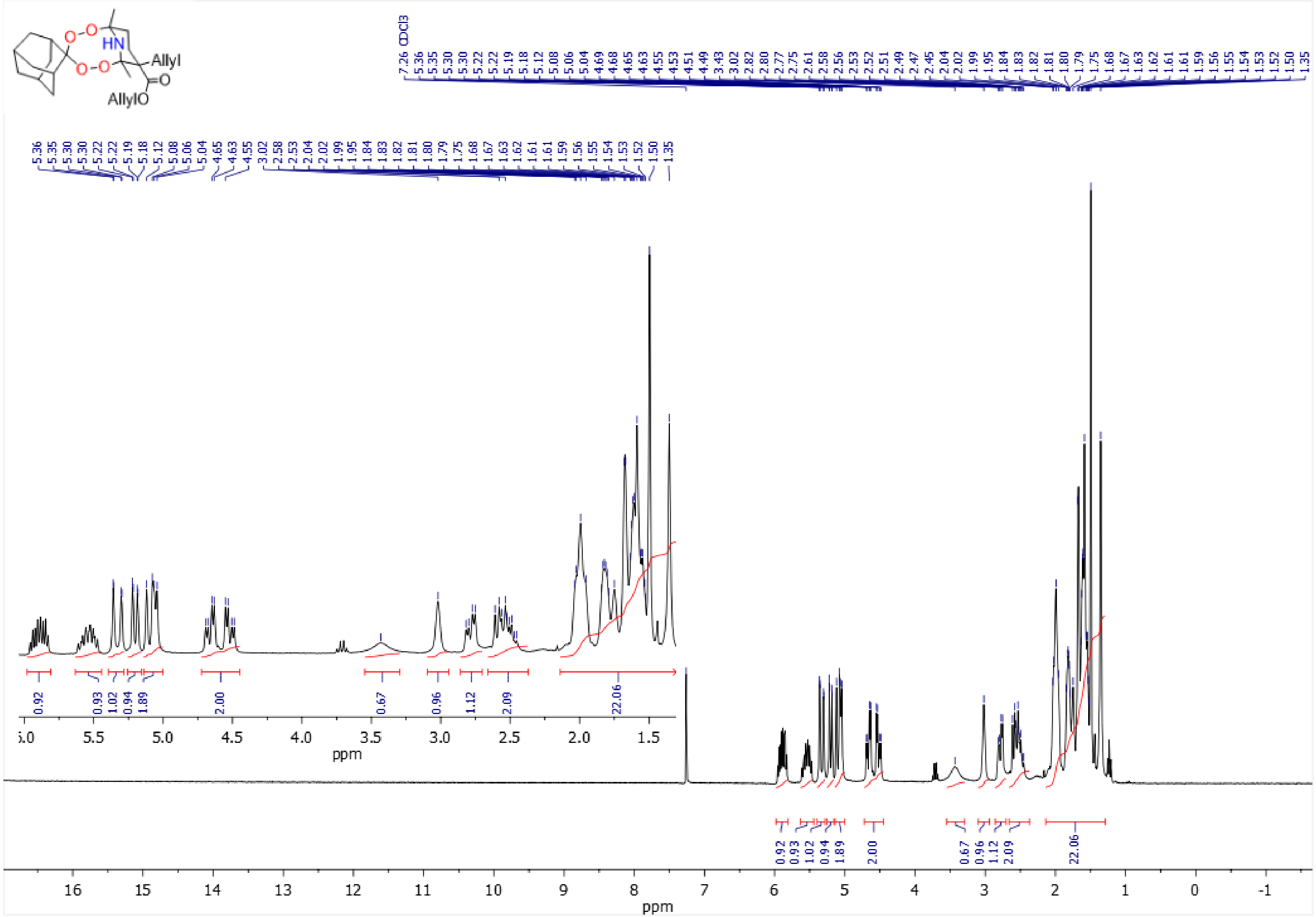

^{13}C NMR (75.48 MHz, CDCl$_3$). Allyl (1*S,1'*R**,2*R**,5*R**,7'*S**,8'*R**)-8'-allyl-1',7'-dimethyl-2',3',5',6'-tetraoxa-11'-azaspiro[adamantane-2,4'-bicyclo[5.3.1]undecane]-8'-carboxylate, 3mc**

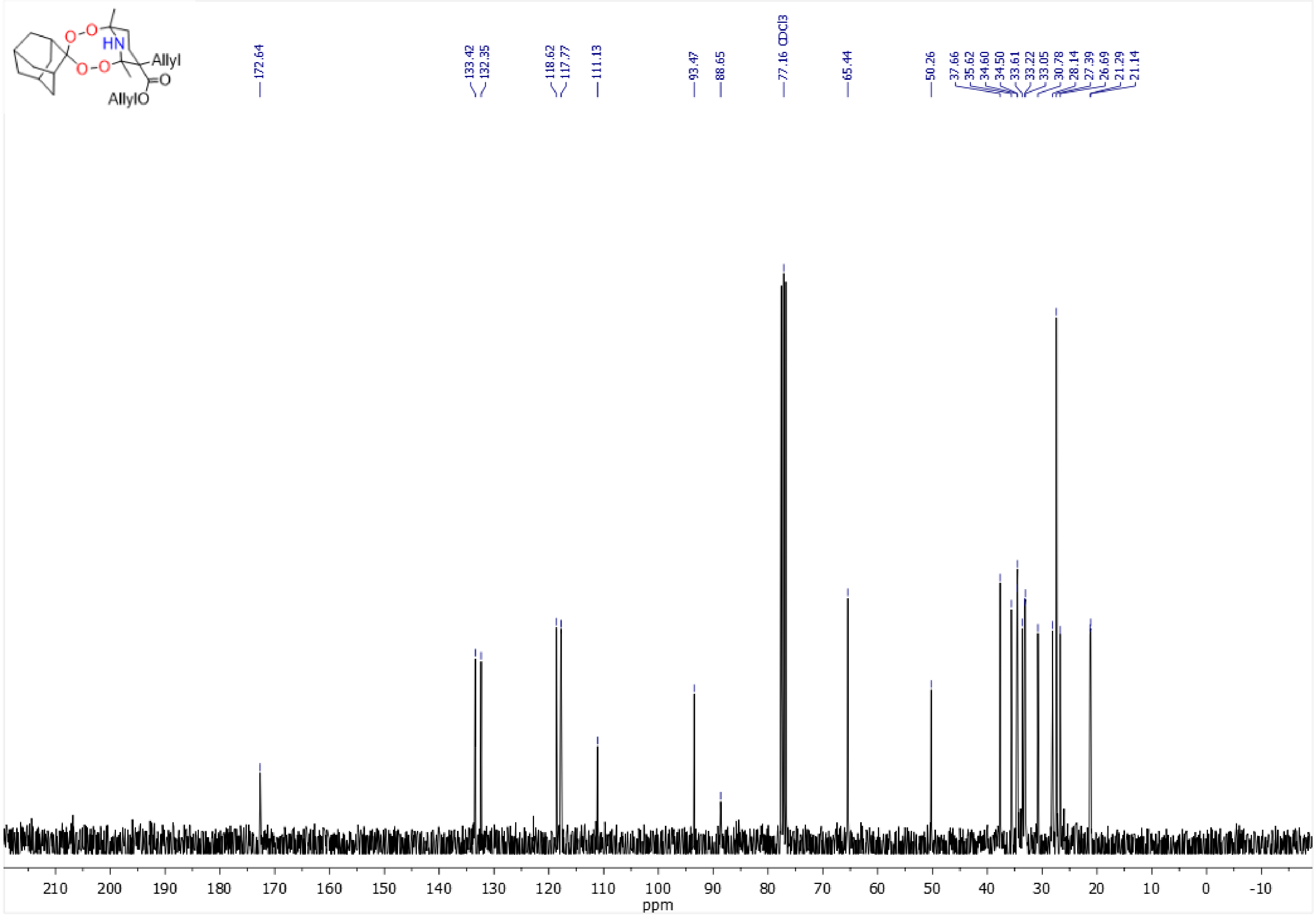

^{1}H NMR (300.13 MHz, CDCl3). Benzyl (1*S,1'*R**,2*R**,5*R**,7'*S**,8'*R**)-8'-allyl-1',7'-dimethyl-2',3',5',6'-tetraoxa-11'-azaspiro[adamantane-2,4'-bicyclo[5.3.1]undecane]-8'-carboxylate, 3nc**

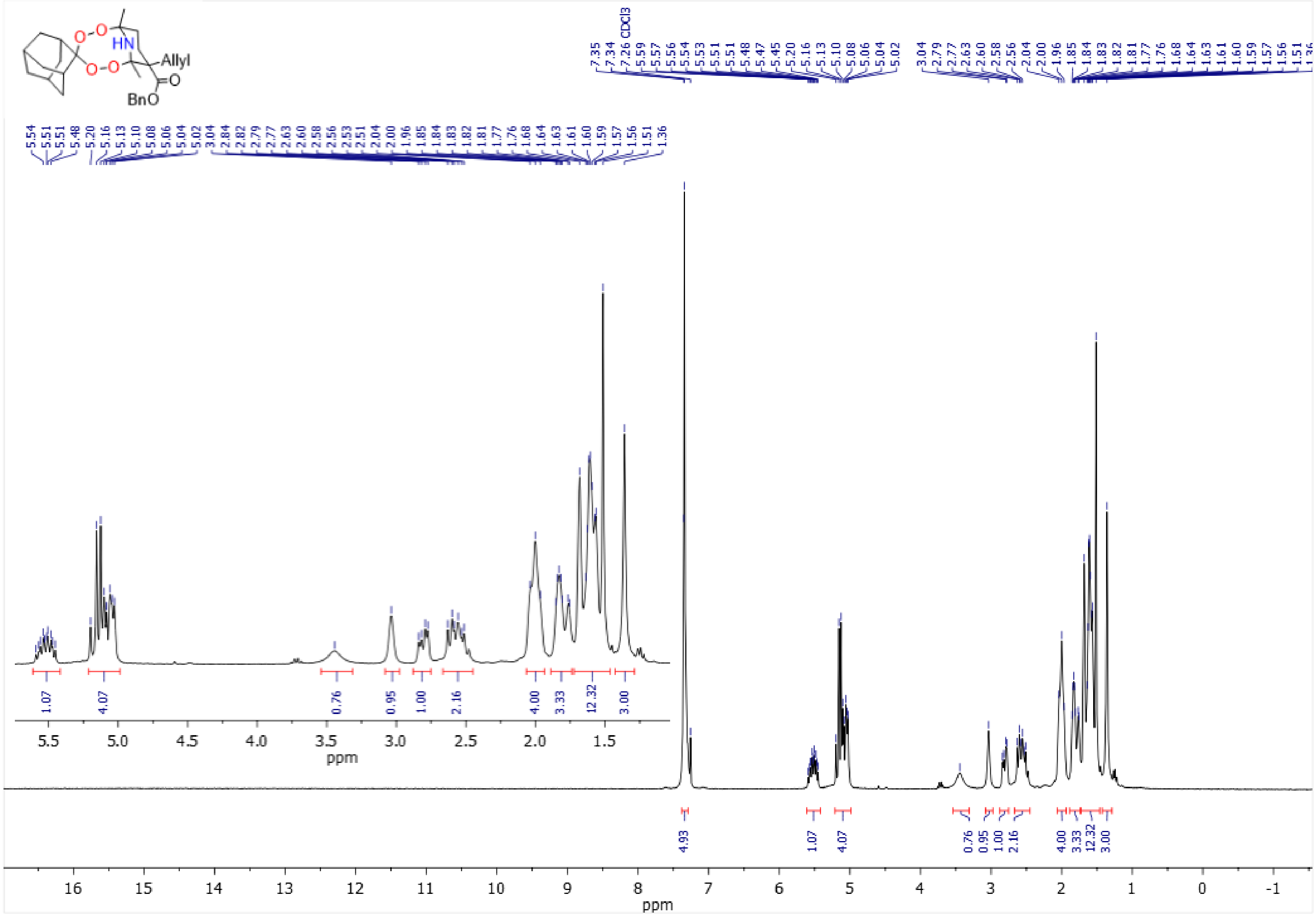

^{13}C NMR (75.48 MHz, CDCl₃). Benzyl (1*S,1'*R**,2*R**,5*R**,7'*S**,8'*R**)-8'-allyl-1',7'-dimethyl-2',3',5',6'-tetraoxa-11'-azaspiro[adamantane-2,4'-bicyclo[5.3.1]undecane]-8'-carboxylate, 3nc**

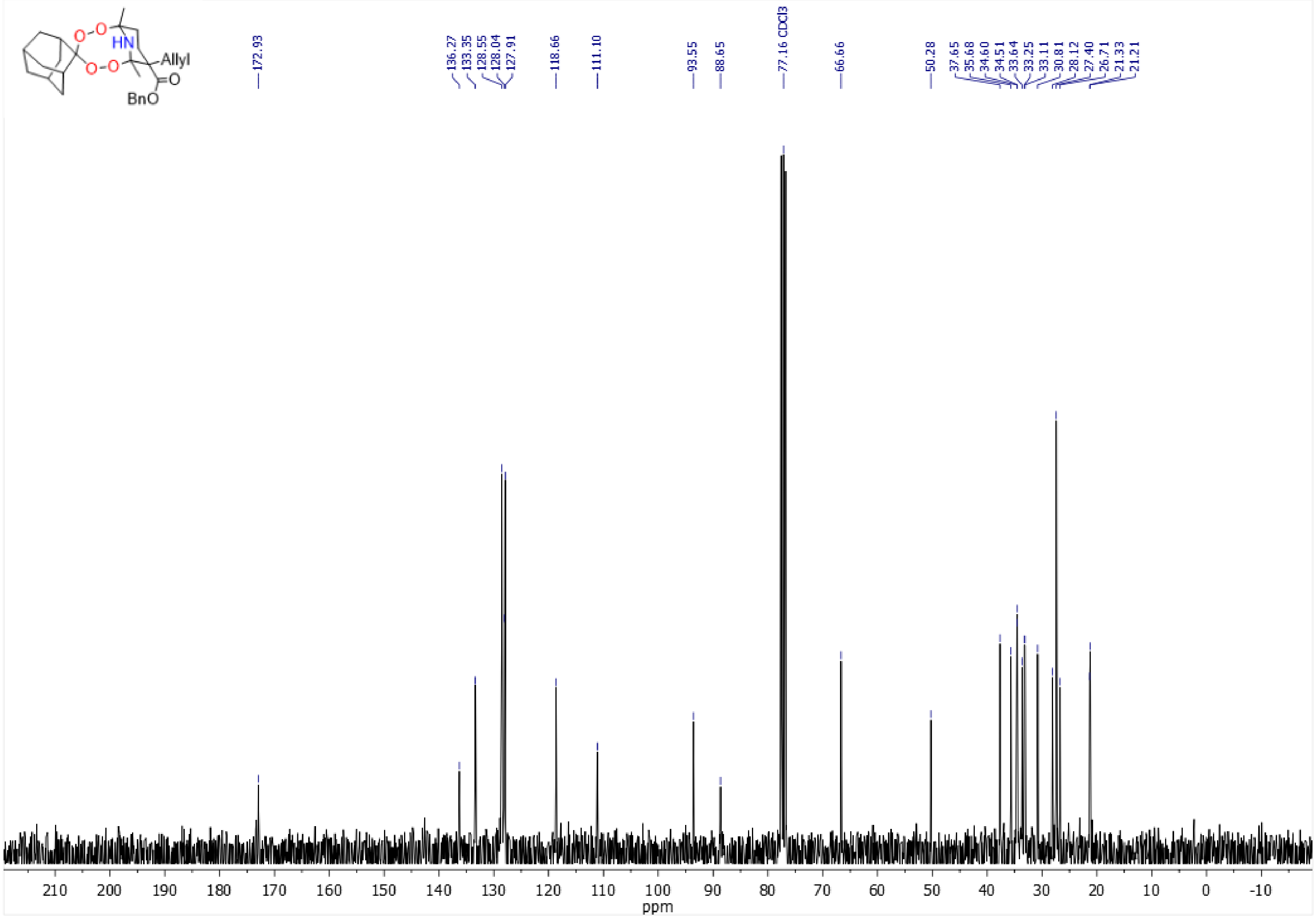

NMR monitoring of the three-component reaction of 1,5-diketone 1l with geminal bishydroperoxide and NH_4OAc in EtOH-*d6*

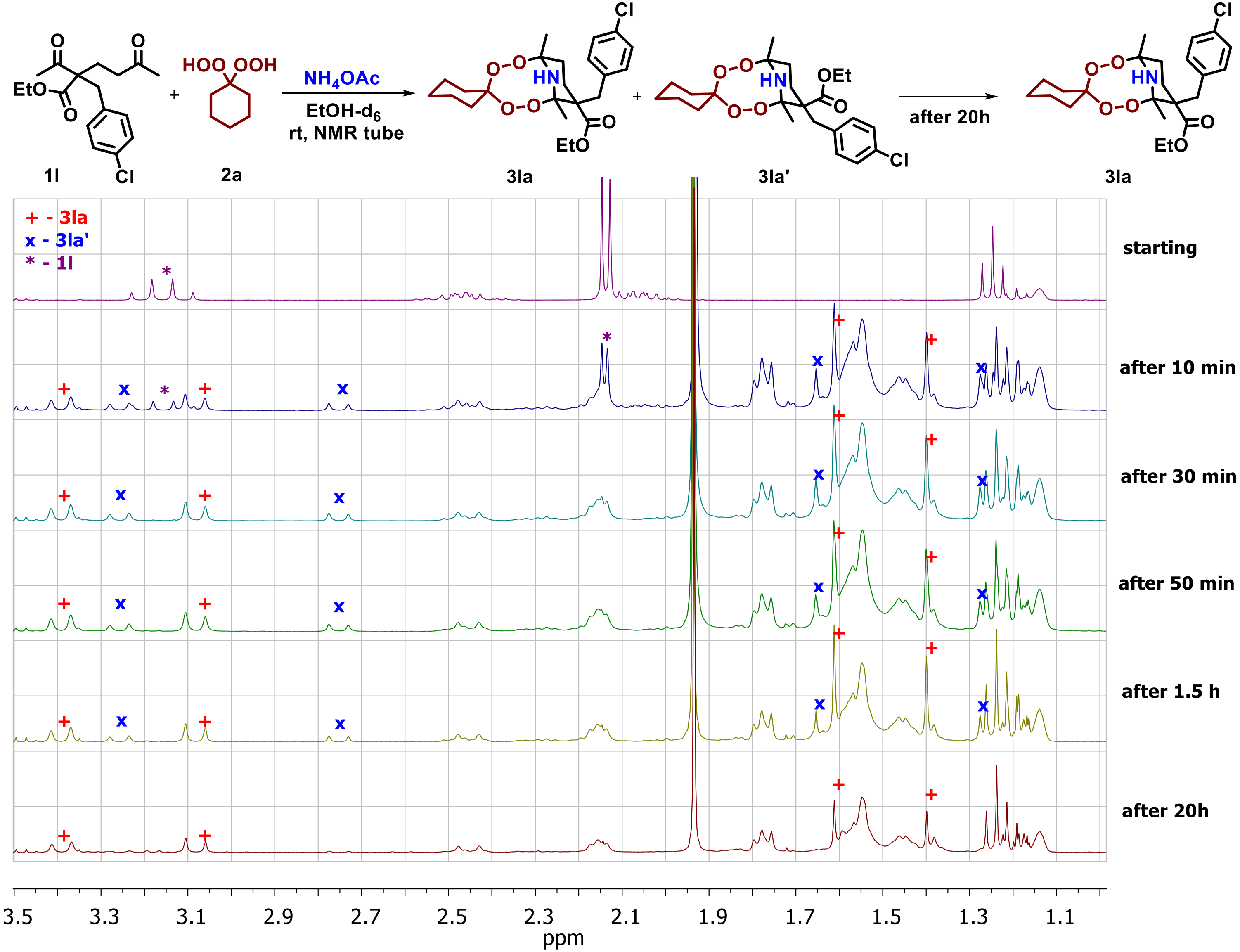

^{1}H NMR (300.13 MHz, EtOH-d_6). Starting diketone 1l

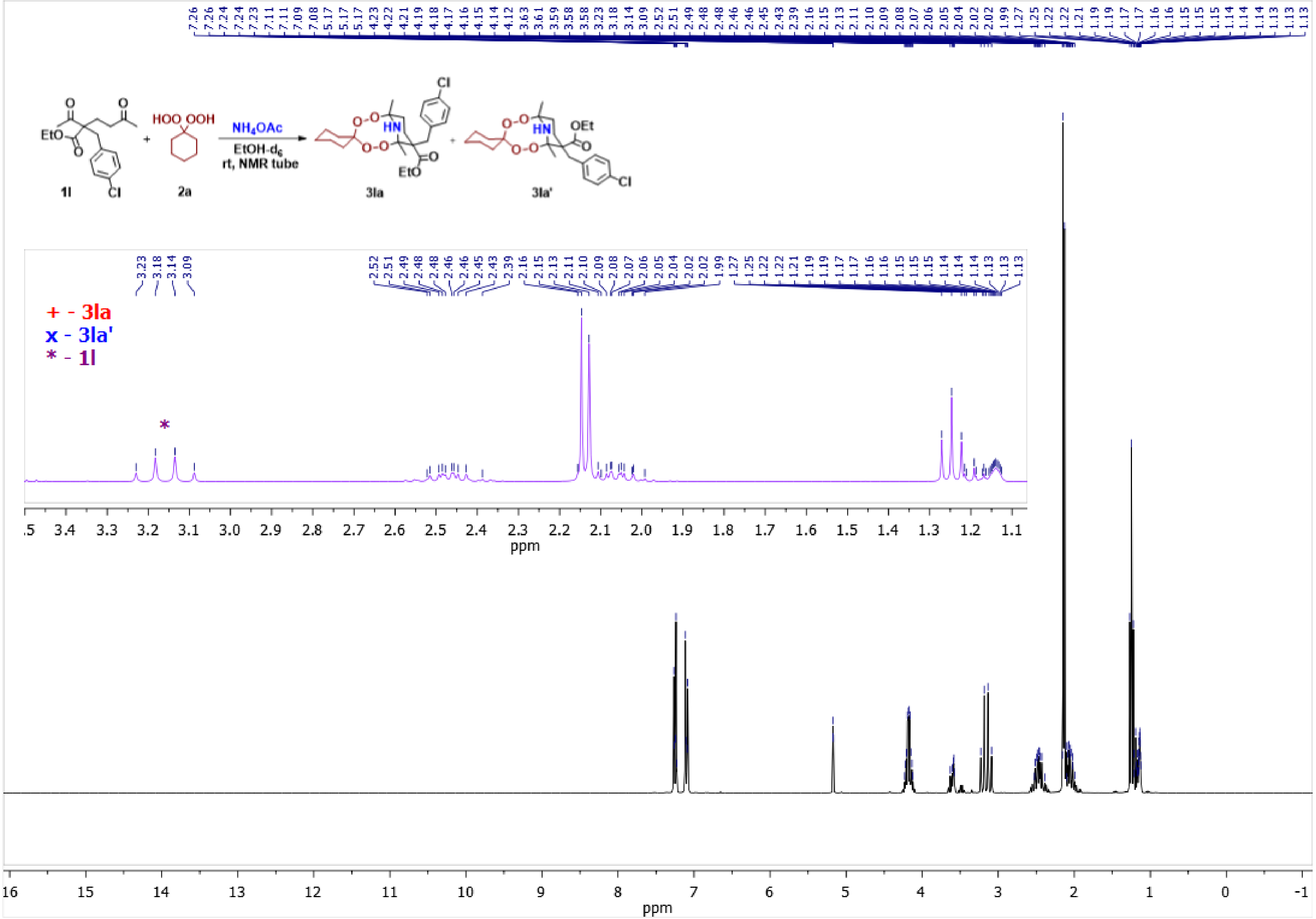

^{1}H NMR (300.13 MHz, EtOH-d_6). Reaction of 1l with 2a and NH_4OAc at rt after 10 min

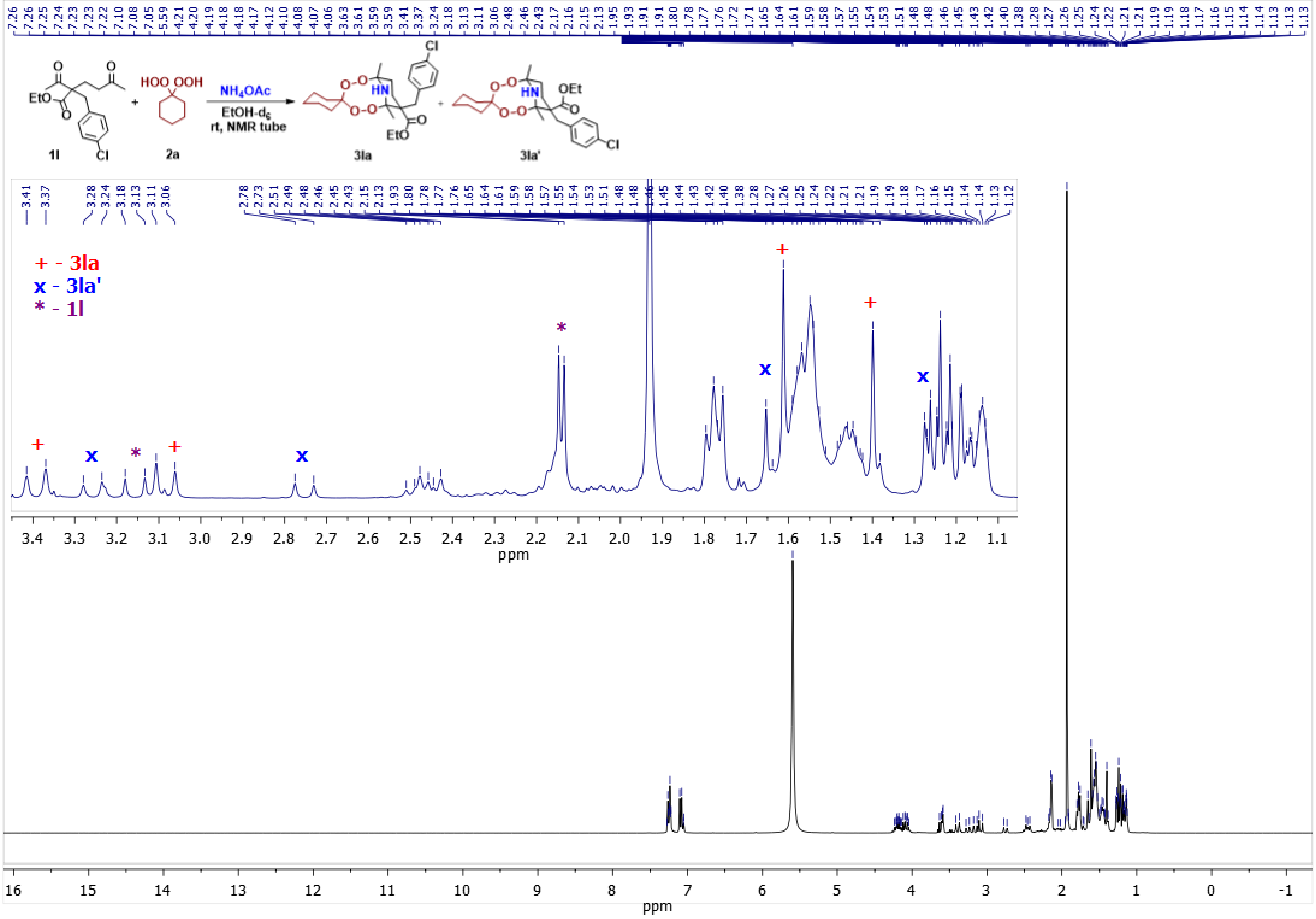

^{1}H NMR (300.13 MHz, EtOH-d_6). Reaction of 1l with 2a and NH_4OAc at rt after 30 min

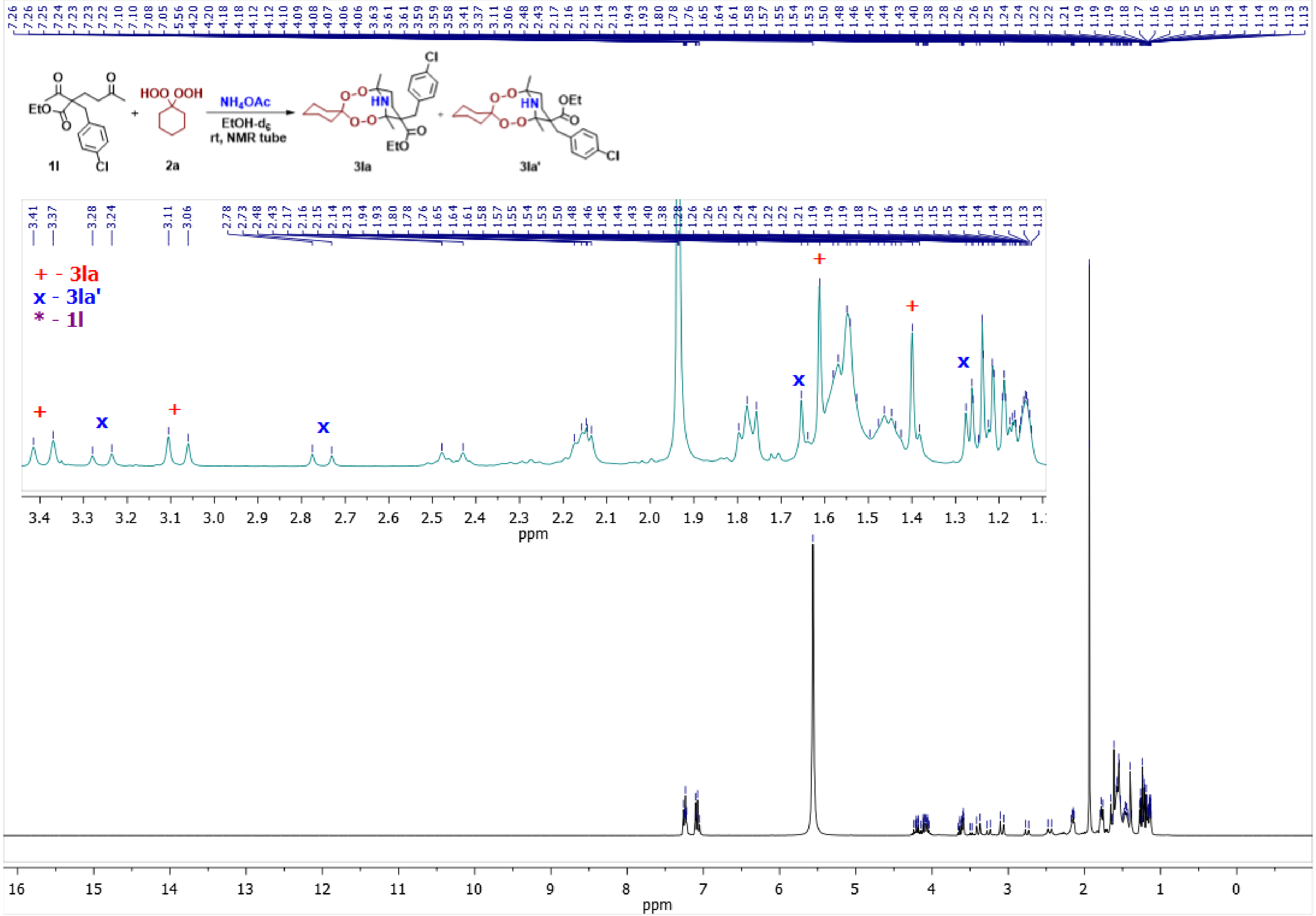

^{1}H NMR (300.13 MHz, EtOH-d_6). Reaction of 1l with 2a and NH_4OAc at rt after 50 min

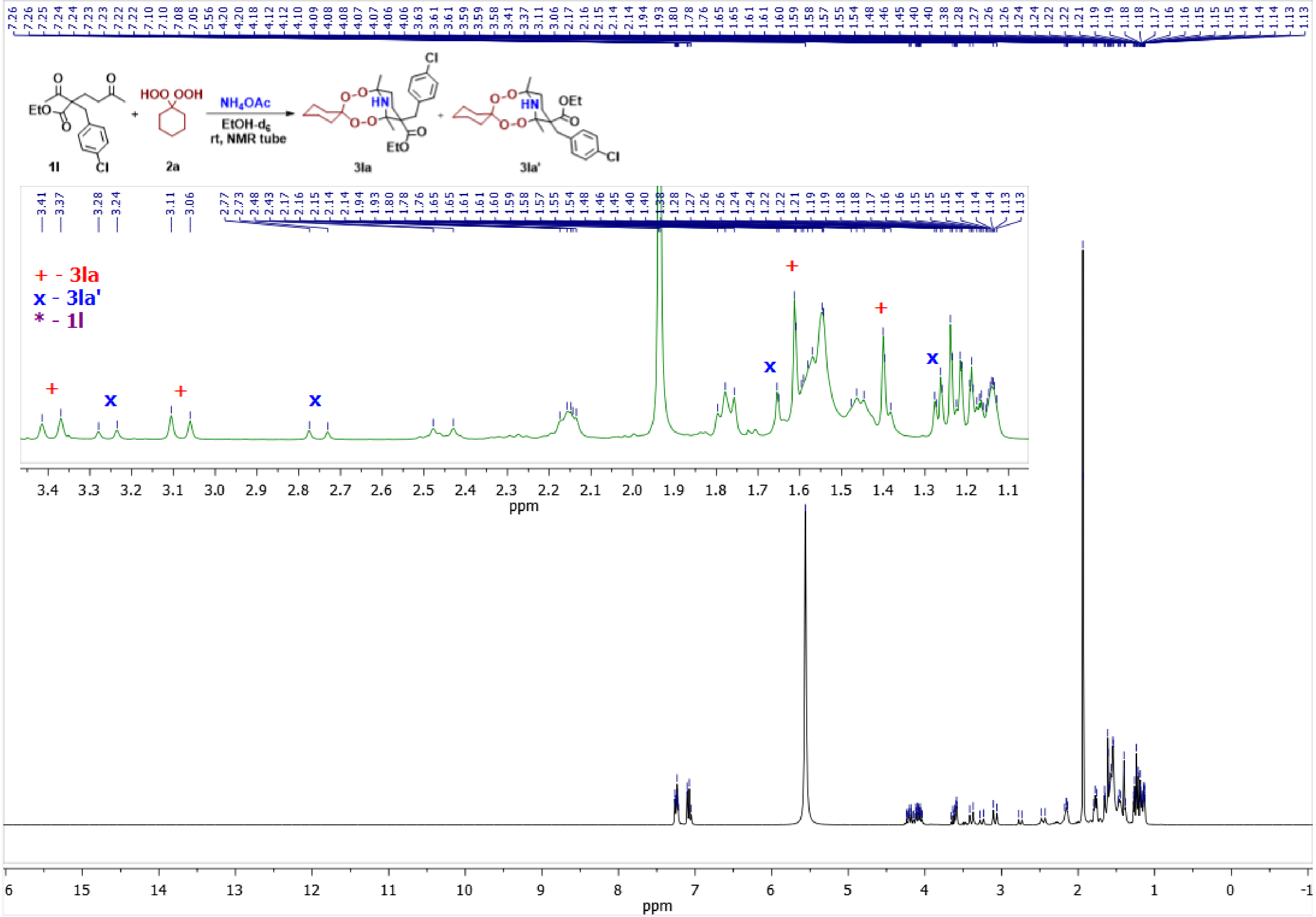

^{15}N NMR (30.41 MHz, EtOH-d_6). Reaction of 1l with 2a and NH_4OAc at rt after 50 min

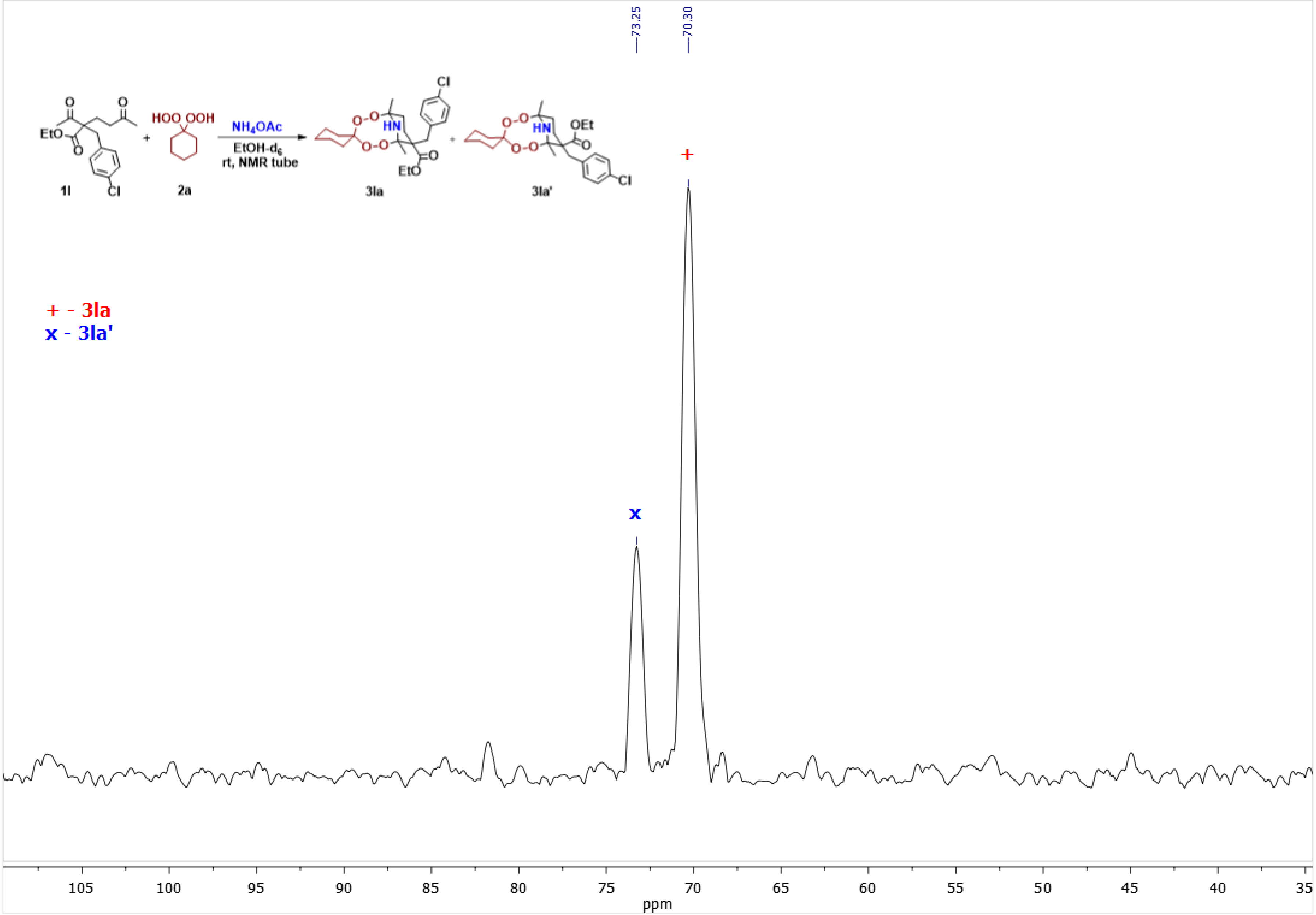

Reaction of 1l with 2a and NH_4OAc at rt after 50 min. ^1H-^{15}N HMBC

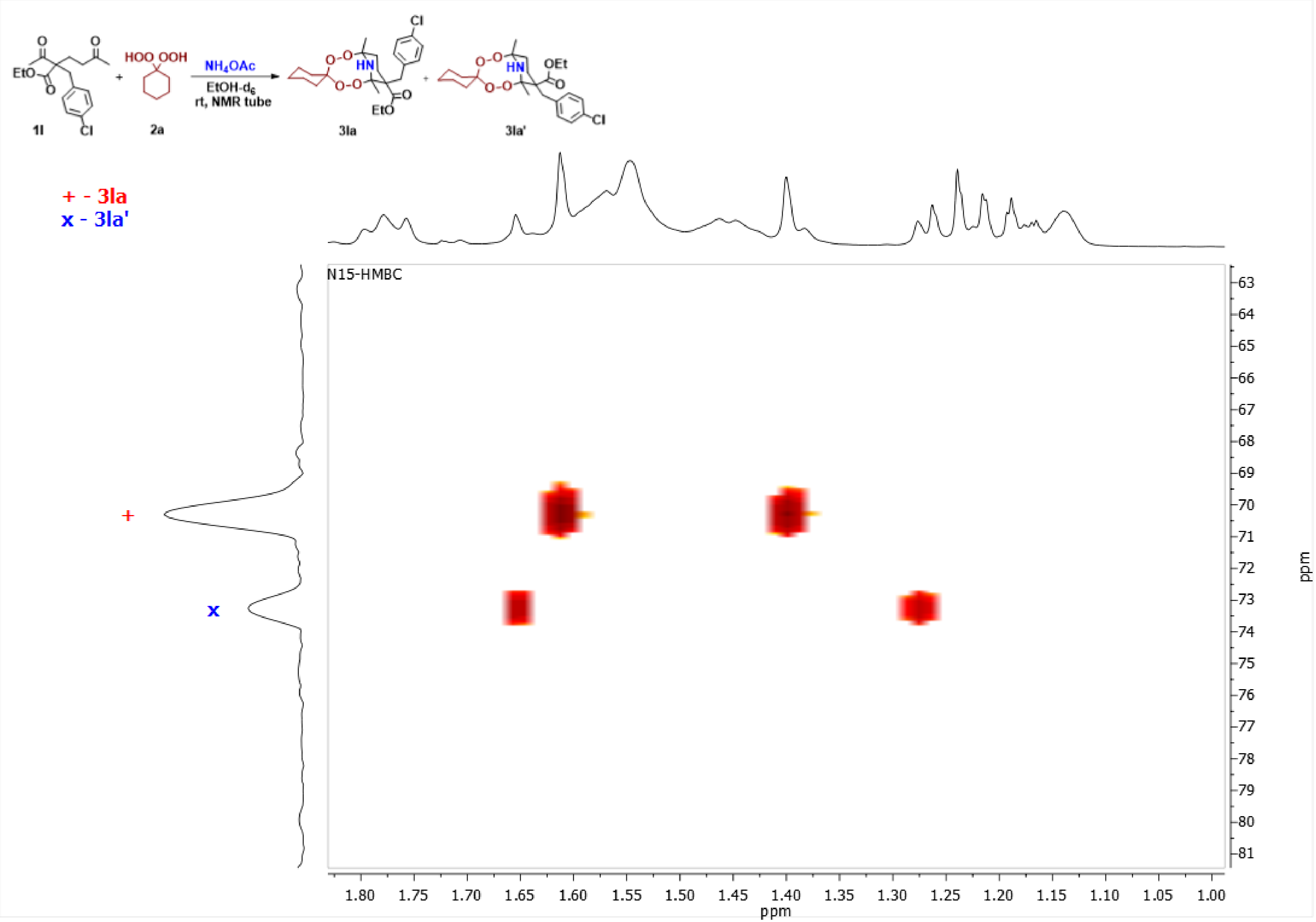

^{1}H NMR (300.13 MHz, EtOH-d_6). Reaction of 1l with 2a and NH₄OAc at rt after 1.5 h

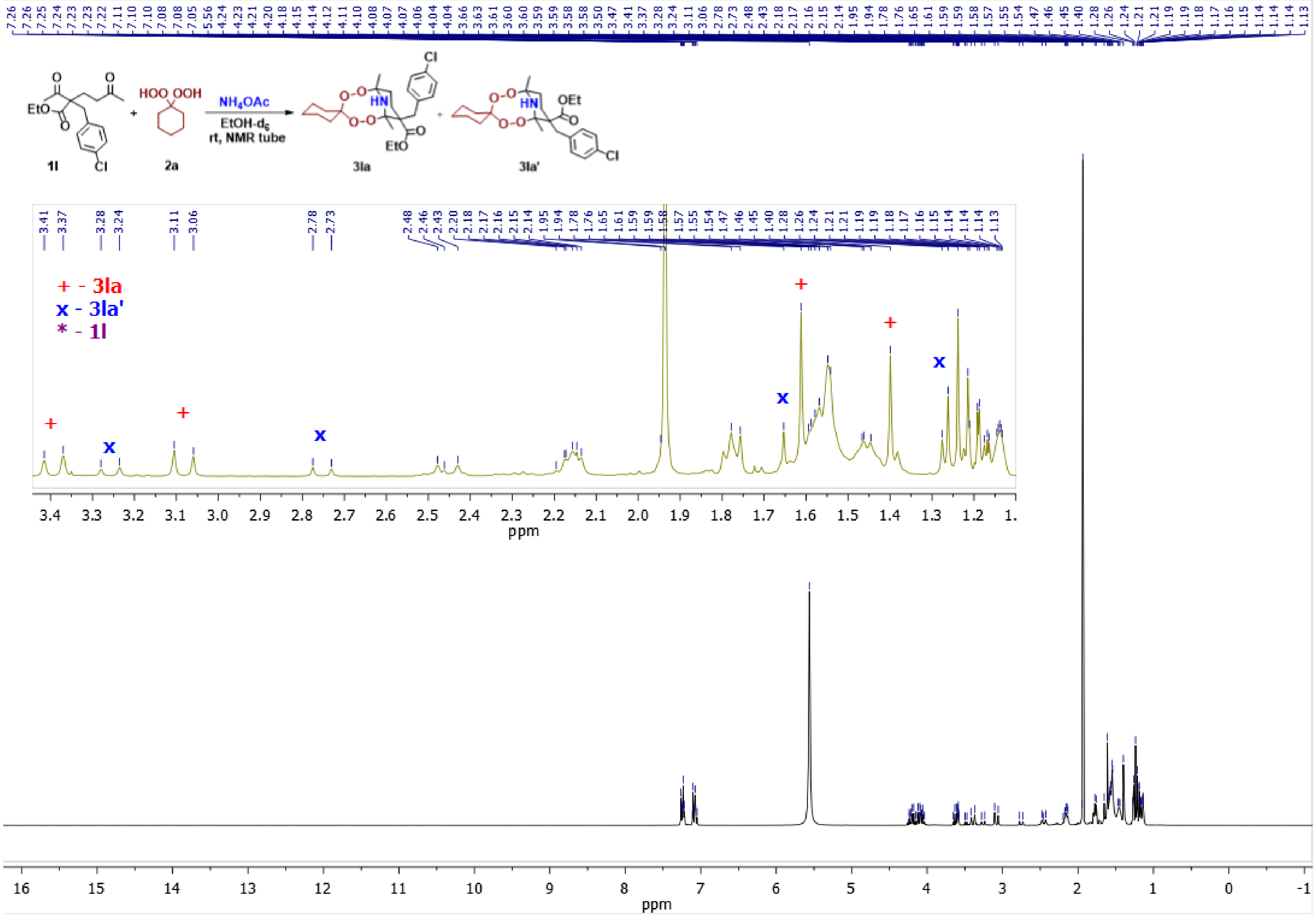

^{1}H NMR (300.13 MHz, EtOH-d_6). Reaction of 1l with 2a and NH_4OAc at rt after 20 h

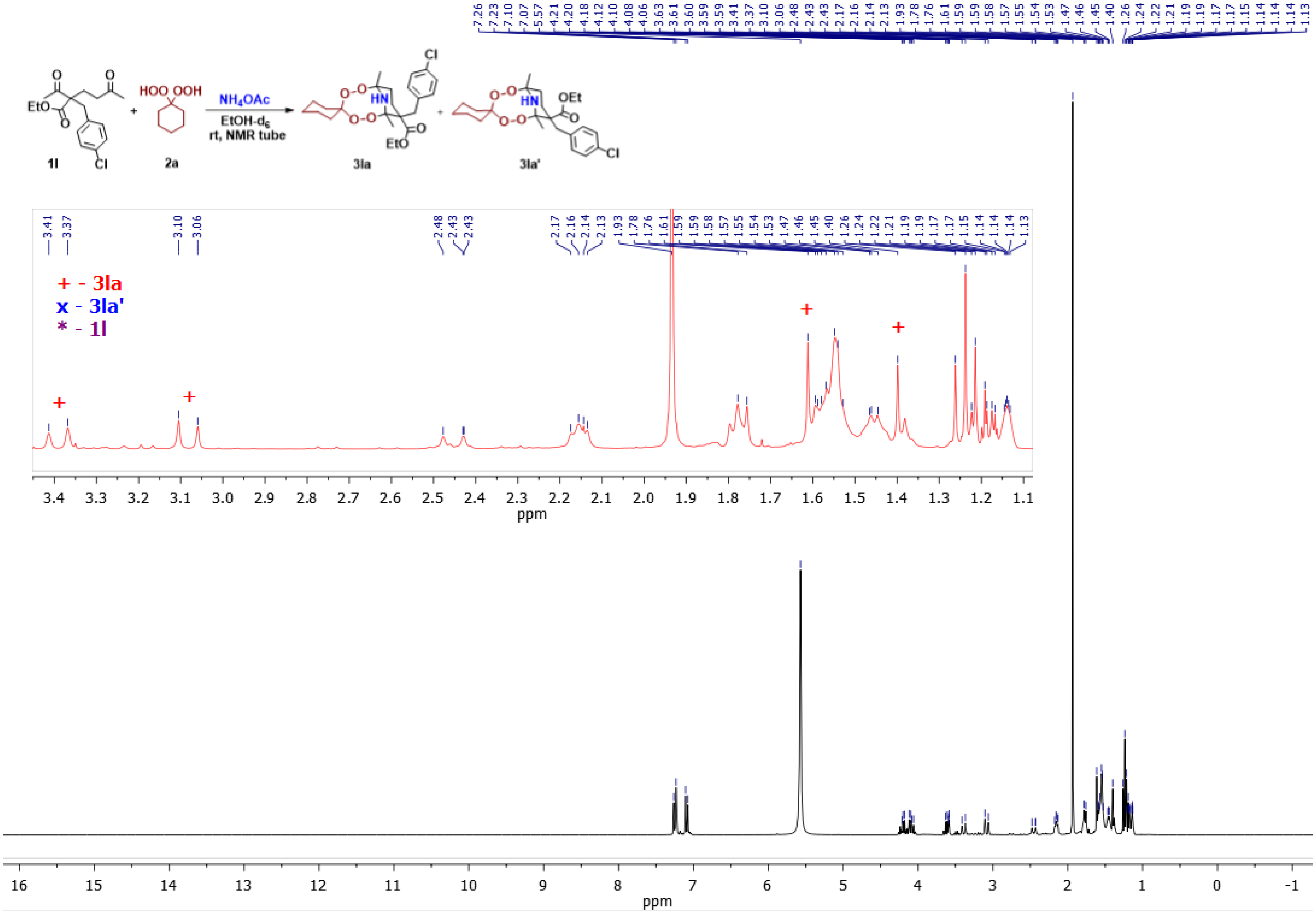

^{15}N NMR (30.41 MHz, EtOH-d_6). Reaction of 1l with 2a and NH_4OAc at rt after 20 h

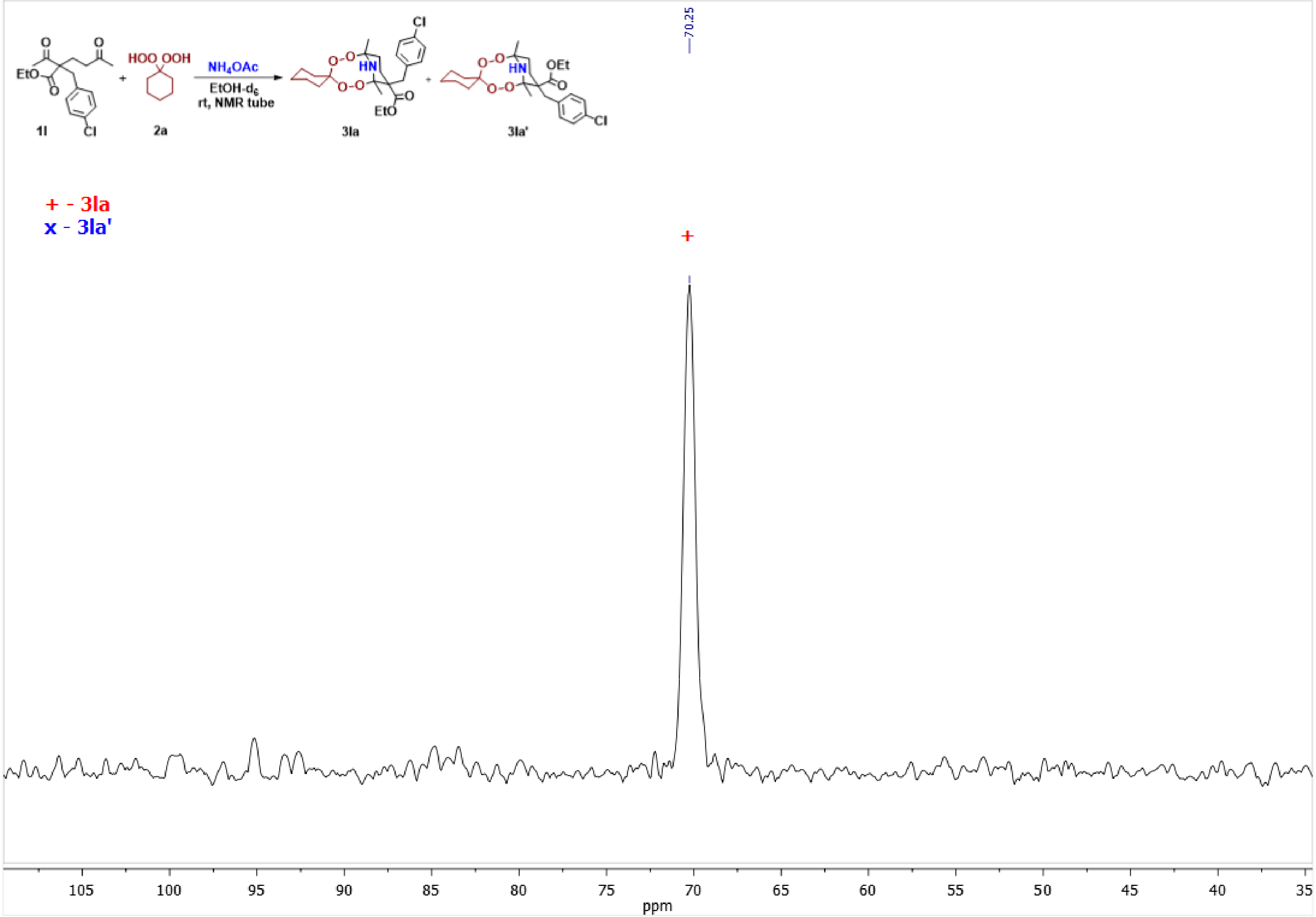

Reaction of 1l with 2a and NH_4OAc at rt after 20 h. ^{1}H-^{15}N HMBC

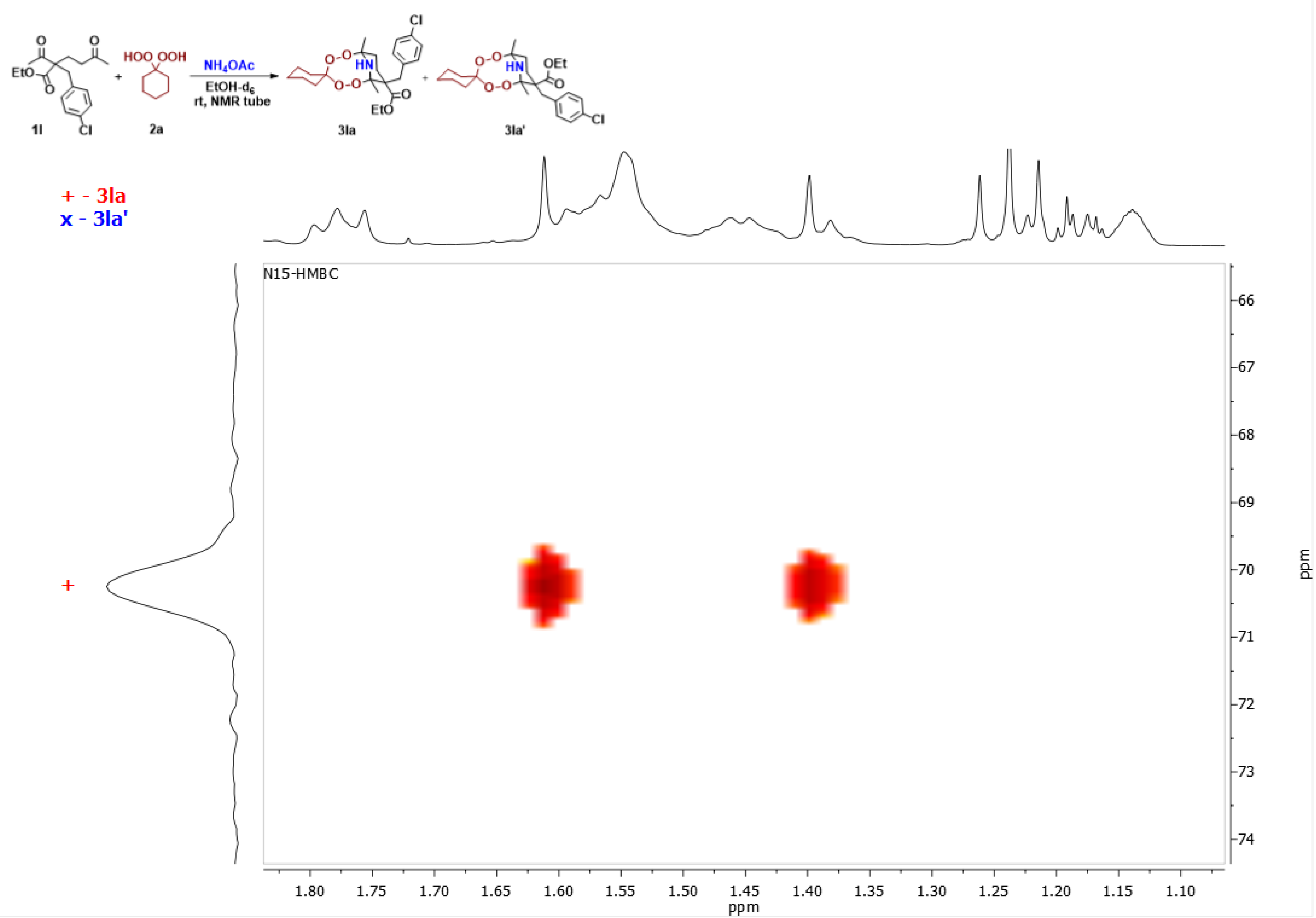

HRMS spectra of aminodiperoxides 3aa, 3da, 3ia, 3ka, 3la, 3kb, 3lb, 3ac–3hc, 3jc-3nc

(1*R,7*S**)-1,7-dimethyl-2,3,5,6-tetraoxa-11-azaspiro[bicyclo[5.3.1]undecane-4,1'-cyclohexane], 3aa**

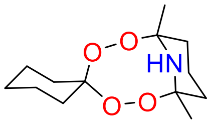

Display Report

Analysis Info

Analysis Name	D:\Data\Kolotyrkina\2024\Belyakova\0305014.d
Method	tune_low.m
Sample Name	/IYAR BL-1589
Comment	C13H23NO4 mH258.1699 calibrant added CH3CN
Acquisition Date	05.03.2024 13:15:04
Operator	BDAL@DE
Instrument / Ser#	micrOTOF 10248

Acquisition Parameter

Source Type	ESI	Ion Polarity	Positive	Set Nebulizer	0.4 Bar
Focus	Not active			Set Dry Heater	180 °C
Scan Begin	50 m/z	Set Capillary	4500 V	Set Dry Gas	4.0 l/min
Scan End	3000 m/z	Set End Plate Offset	-500 V	Set Divert Valve	Waste

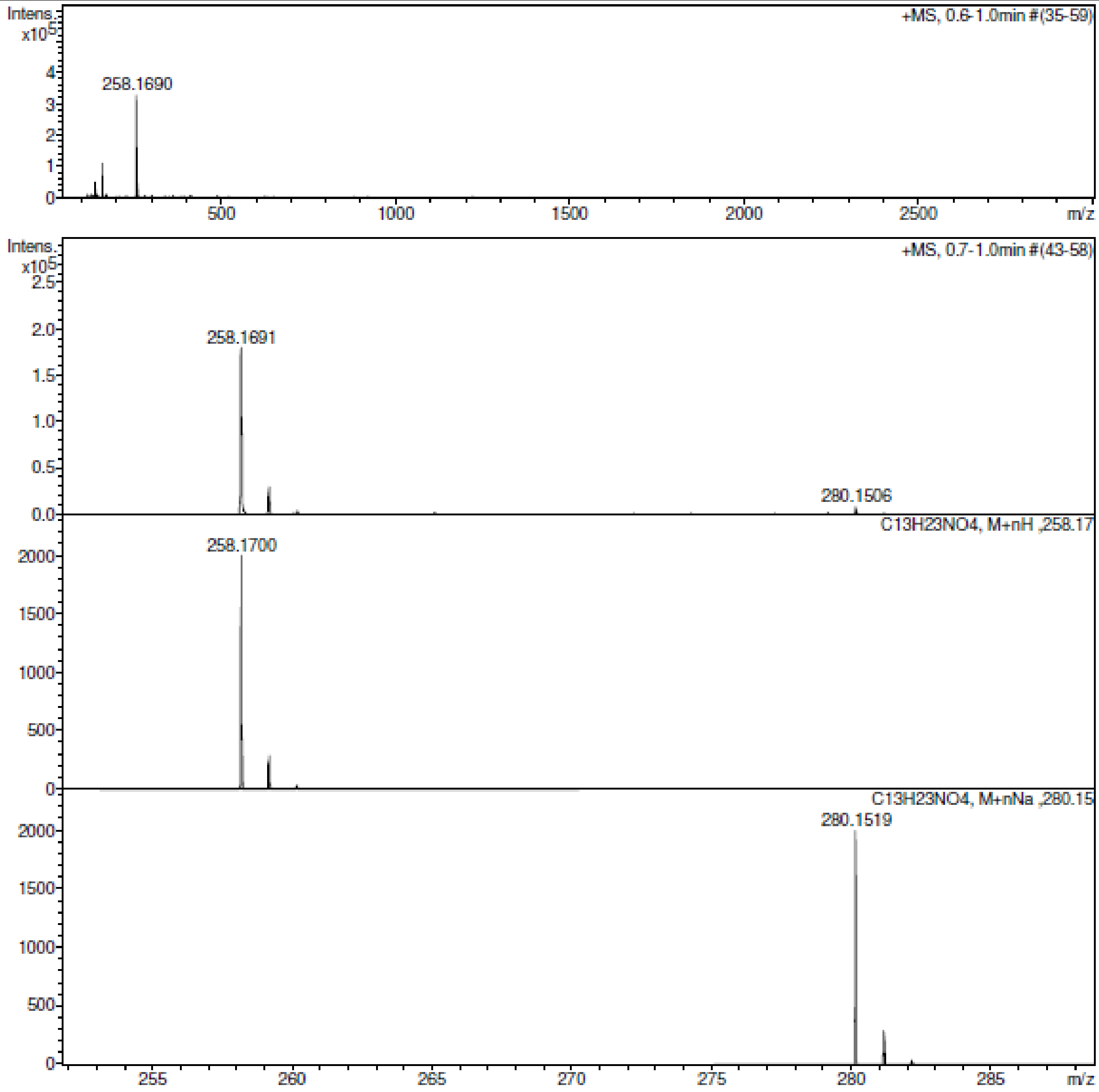

Ethyl (1*R**,7*S**,8*S**)-8-butyl-1,7-dimethyl-2,3,5,6-tetraoxa-11-azaspiro[bicyclo[5.3.1]undecane-4,1'-cyclohexane]-8-carboxylate, 3da

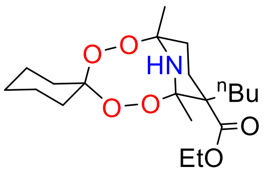

Display Report

Analysis Info

Analysis Name	D:\Data\Kolotyrkina\2022\Belyakova\0301018.d
Method	tune_50-1600_pos_15_12.m
Sample Name	/TERN BL-1319
Comment	C20H35NO6 mH 386.2537 calibrant added CH3OH

Acquisition Date	01.03.2022 12:25:08	
Operator	BDAL@DE	
Instrument / Ser#	micrOTOF	10248

Acquisition Parameter

Source Type	ESI	Ion Polarity	Positive	Set Nebulizer	0.4 Bar
Focus	Not active			Set Dry Heater	180 °C
Scan Begin	50 m/z	Set Capillary	4500 V	Set Dry Gas	4.0 l/min
Scan End	1600 m/z	Set End Plate Offset	-500 V	Set Divert Valve	Waste

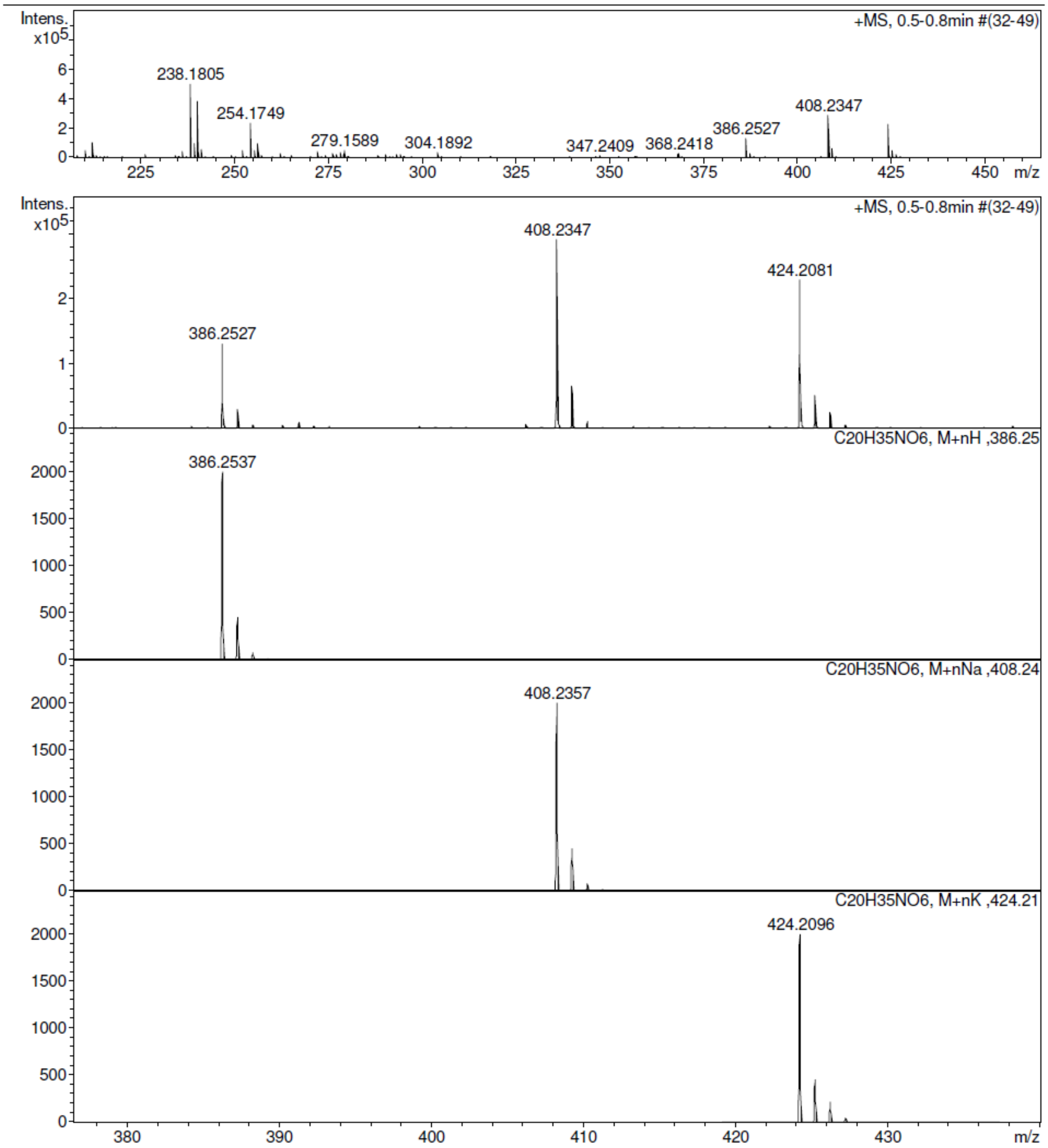

Bruker Compass DataAnalysis 4.0	printed: 01.03.2022 12:30:11	Page 1 of 1

Ethyl (1*R,7*S**,8*R**)-1,7-dimethyl-8-(4-methylbenzyl)-2,3,5,6-tetraoxa-11-azaspiro[bicyclo[5.3.1]undecane-4,1'-cyclohexane]-8-carboxylate, 3ia**

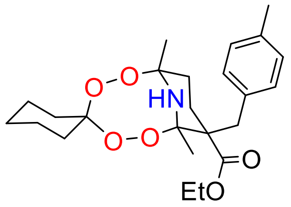

Display Report

Analysis Info

Analysis Name	D:\Data\Chizhov\Terentiev\Belyakova\bl-1341_&clblow.d	Acquisition Date	18.03.2022 14:52:06
Method	tune_low.m	Operator	BDAL@DE
Sample Name	/TERN BL-1341	Instrument / Ser#	micrOTOF 10248
Comment	CH3CN 100 %, dil. 200, calibrant added		

Acquisition Parameter

Source Type	ESI	Ion Polarity	Positive	Set Nebulizer	0.4 Bar
Focus	Not active			Set Dry Heater	180 °C
Scan Begin	50 m/z	Set Capillary	4500 V	Set Dry Gas	4.0 l/min
Scan End	3000 m/z	Set End Plate Offset	-500 V	Set Divert Valve	Waste

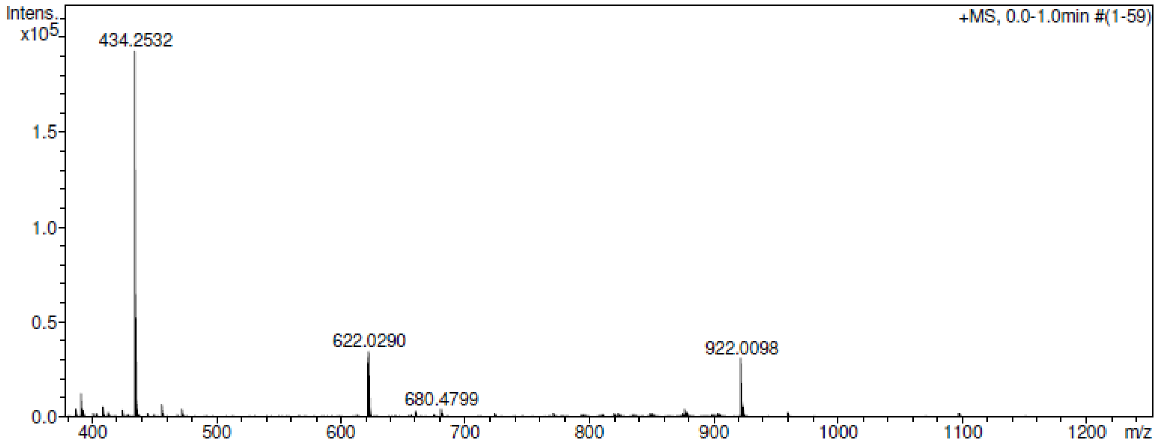

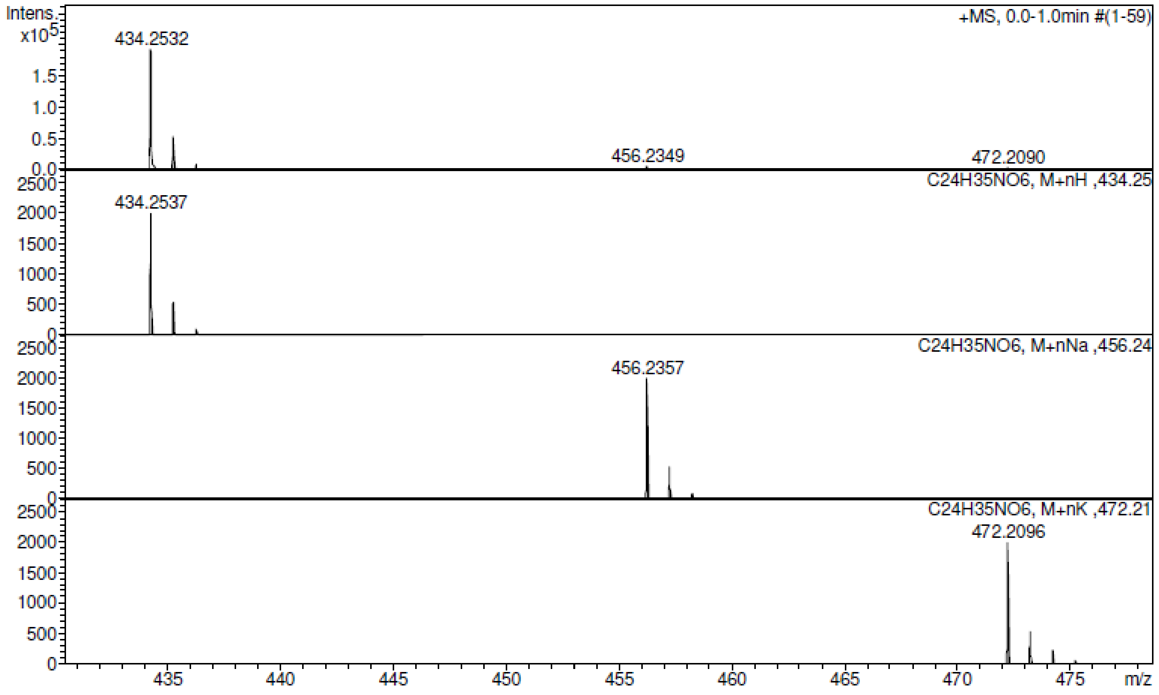

Bruker Compass DataAnalysis 4.0 printed: 18.03.2022 15:00:01 Page 1 of 1

Ethyl (1*R,7*S**,8*R**)-8-(4-fluorobenzyl)-1,7-dimethyl-2,3,5,6-tetraoxa-11-azaspiro[bicyclo[5.3.1]undecane-4,1'-cyclohexane]-8-carboxylate, 3ka**

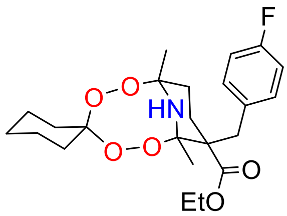

Display Report

Analysis Info

Analysis Name	D:\Data\Kolotyrkina\2022\Belyakova\0322038.d	Acquisition Date	22.03.2022 17:06:32
Method	tune_50-1600_pos_15_12.m	Operator	BDAL@DE
Sample Name	/TERN BL-1345	Instrument / Ser#	micrOTOF 10248
Comment	C23H32FNO6 mH 438.2286 calibrant added CN3CN		

Acquisition Parameter

Source Type	ESI	Ion Polarity	Positive	Set Nebulizer	0.4 Bar
Focus	Not active			Set Dry Heater	180 °C
Scan Begin	50 m/z	Set Capillary	4500 V	Set Dry Gas	4.0 l/min
Scan End	1600 m/z	Set End Plate Offset	-500 V	Set Divert Valve	Waste

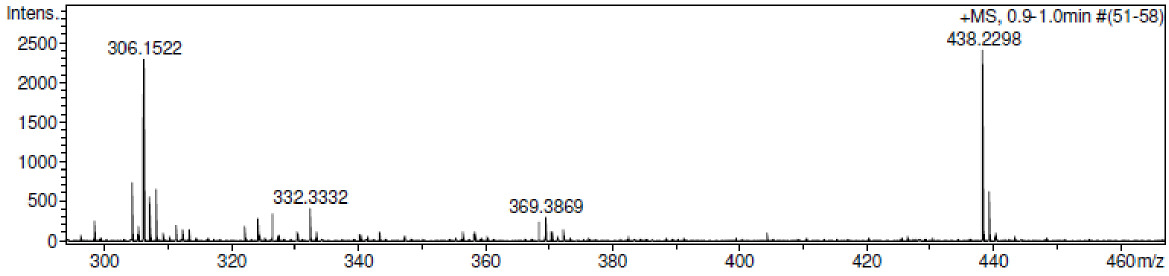

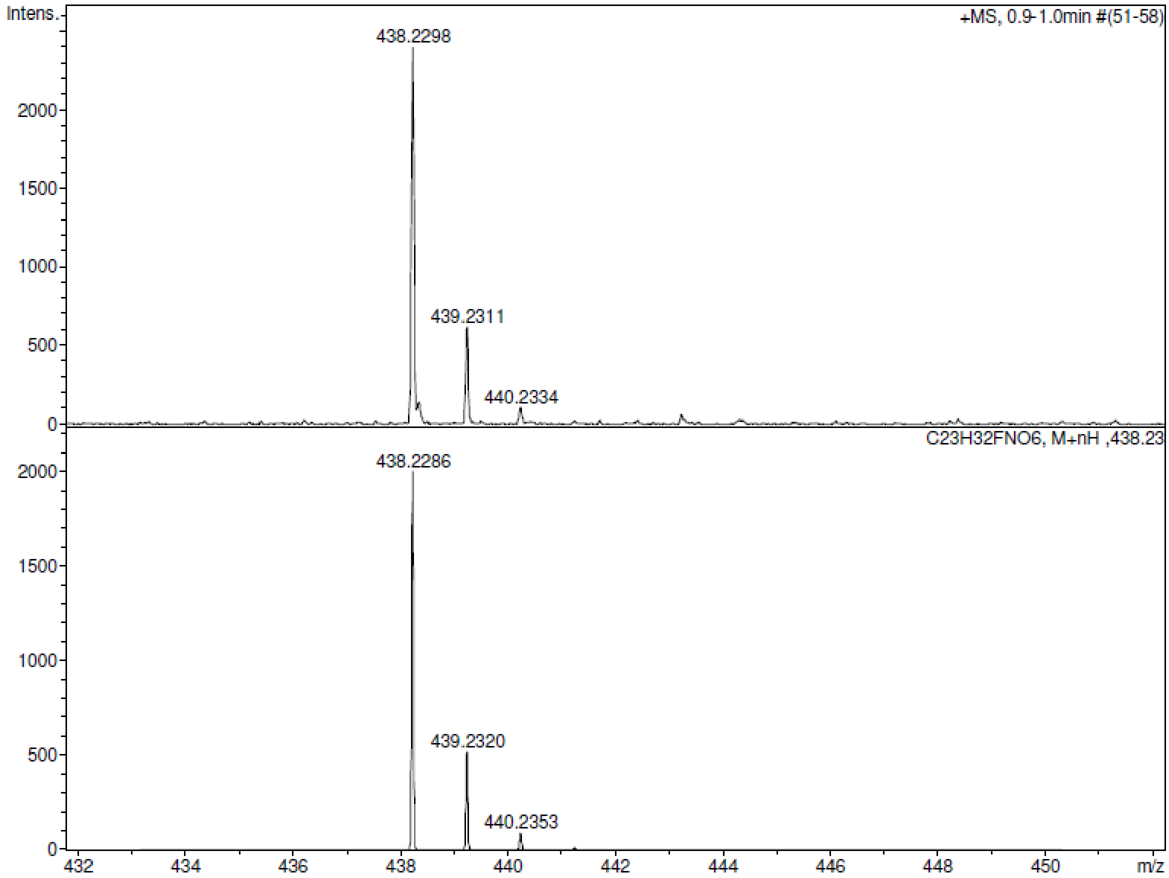

Bruker Compass DataAnalysis 4.0 printed: 22.03.2022 17:15:40 Page 1 of 1

Ethyl (1*R,7*S**,8*R**)-8-(4-chlorobenzyl)-1,7-dimethyl-2,3,5,6-tetraoxa-11-azaspiro[bicyclo[5.3.1]undecane-4,1'-cyclohexane]-8-carboxylate, 3la**

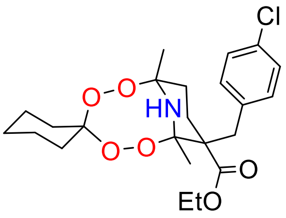

Display Report

Analysis Info

Analysis Name	D:\Data\Chizhov\Terentiev\Belyakova\bl-1273_&clblow.d	Acquisition Date	14.01.2022 11:57:55
Method	tune_low.m	Operator	BDAL@DE
Sample Name	/TERN BL-1273	Instrument / Ser#	micrOTOF 10248
Comment	CH3OH 100 %, dil. 200, calibrant added		

Acquisition Parameter

Source Type	ESI	Ion Polarity	Positive	Set Nebulizer	0.4 Bar
Focus	Not active			Set Dry Heater	180 °C
Scan Begin	50 m/z	Set Capillary	4500 V	Set Dry Gas	4.0 l/min
Scan End	3000 m/z	Set End Plate Offset	-500 V	Set Divert Valve	Waste

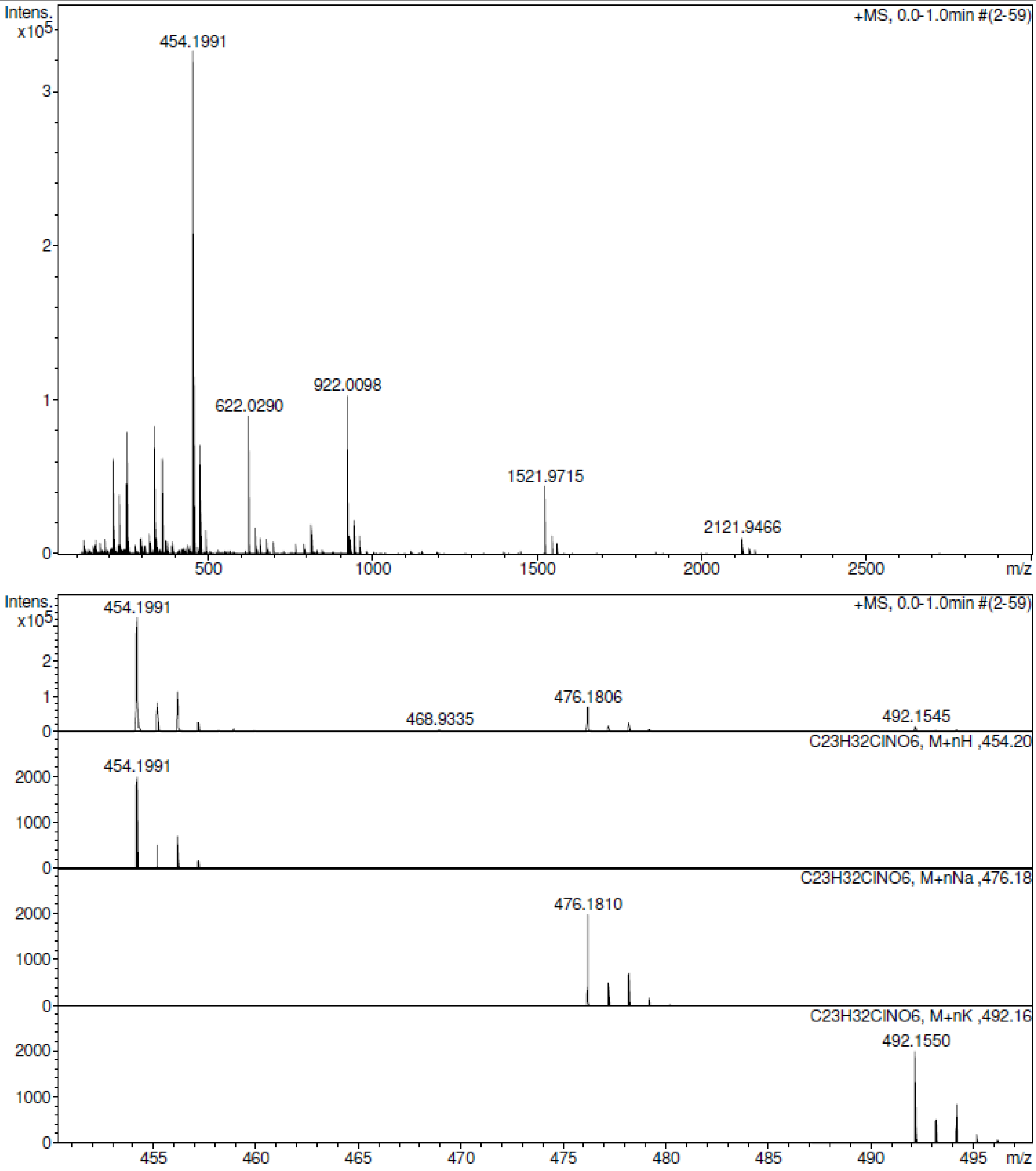

Ethyl (1*R,7*S**,8*R**)-8-(4-fluorobenzyl)-1,7-dimethyl-2,3,5,6-tetraoxa-11-azaspiro[bicyclo[5.3.1]undecane-4,1'-cycloheptane]-8-carboxylate, 3kb**

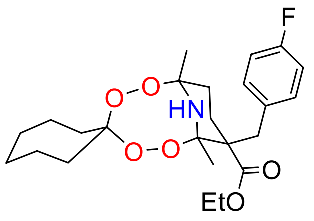

Display Report

Analysis Info			
Analysis Name	D:\Data\Chizhov\Terentiev\Belyakova\bl-1348_&clb.d	Acquisition Date	25.03.2022 14:13:38
Method	tune_wide.m	Operator	BDAL@DE
Sample Name	/TERN BL-1348	Instrument / Ser#	micrOTOF 10248
Comment	CH3CN 100 %, dil. 200, calibrant added		

Acquisition Parameter

Source Type	ESI	Ion Polarity	Positive	Set Nebulizer	0.4 Bar
Focus	Not active			Set Dry Heater	180 °C
Scan Begin	50 m/z	Set Capillary	4500 V	Set Dry Gas	4.0 l/min
Scan End	3000 m/z	Set End Plate Offset	-500 V	Set Divert Valve	Waste

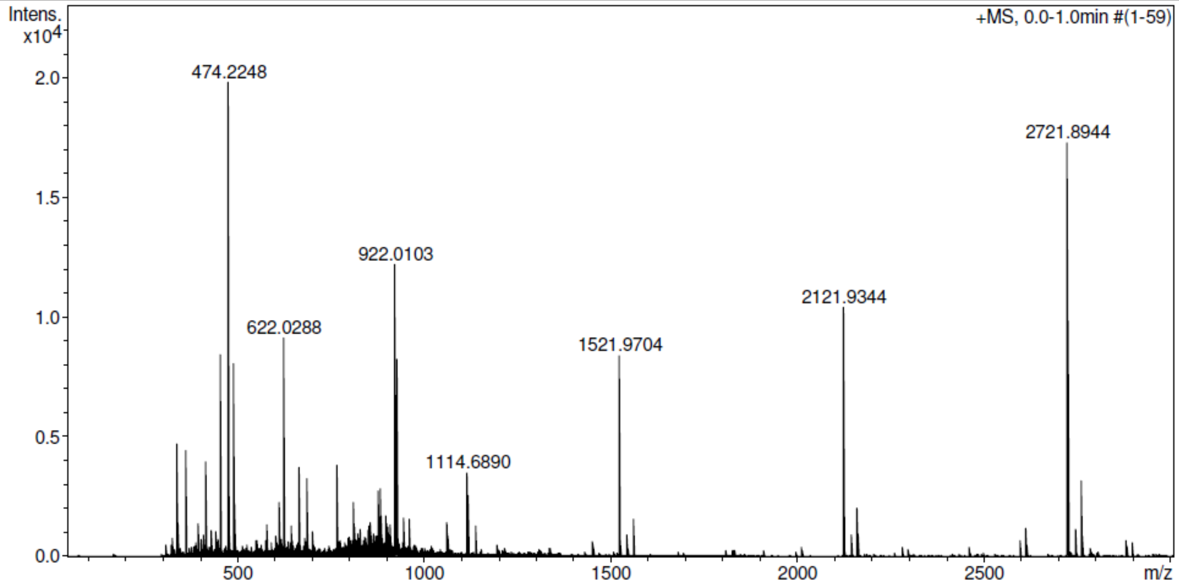

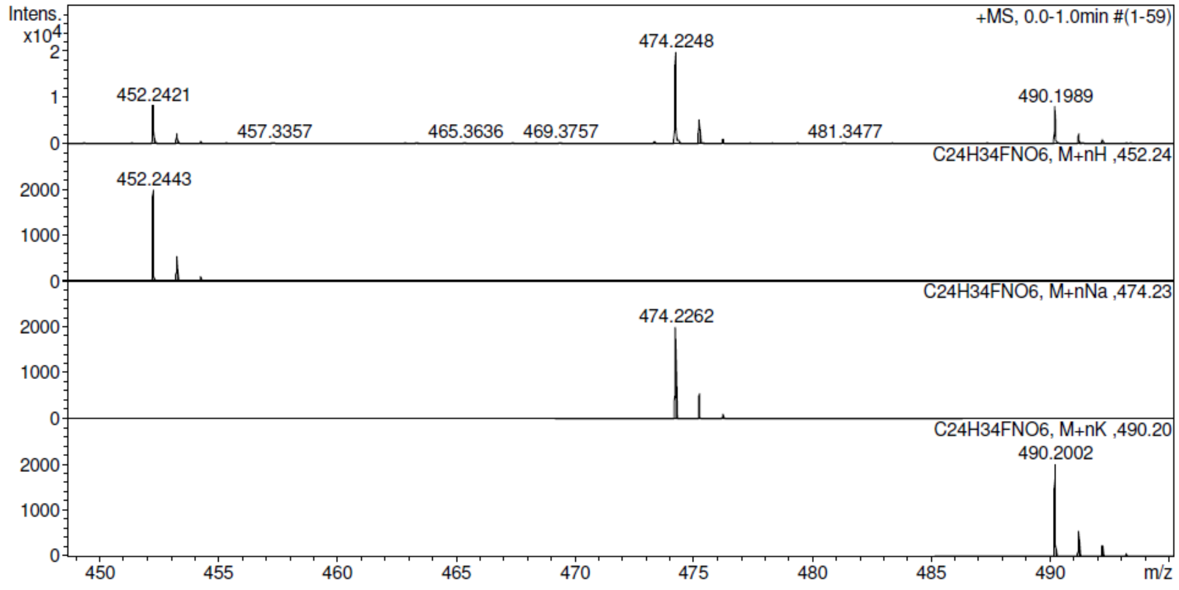

Bruker Compass DataAnalysis 4.0 printed: 25.03.2022 14:31:27 Page 1 of 1

Ethyl (1*R,7*S**,8*R**)-8-(4-chlorobenzyl)-1,7-dimethyl-2,3,5,6-tetraoxa-11-azaspiro[bicyclo[5.3.1]undecane-4,1'-cycloheptane]-8-carboxylate, 3lb**

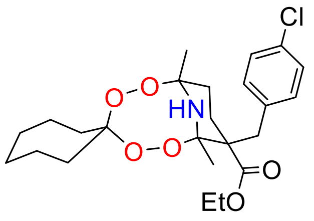

Display Report

Analysis Info

Analysis Name	D:\Data\Kolotyrkina\2022\Belyakova\0322039.d	Acquisition Date	22.03.2022 17:18:10
Method	tune_50-1600_pos_15_12.m	Operator	BDAL@DE
Sample Name	/TERN BL-1346	Instrument / Ser#	micrOTOF 10248
Comment	C24H34ClNO6 mH 468.2147 calibrant added CN3CN		

Acquisition Parameter

Source Type	ESI	Ion Polarity	Positive	Set Nebulizer	0.4 Bar
Focus	Not active			Set Dry Heater	180 °C
Scan Begin	50 m/z	Set Capillary	4500 V	Set Dry Gas	4.0 l/min
Scan End	1600 m/z	Set End Plate Offset	-500 V	Set Divert Valve	Waste

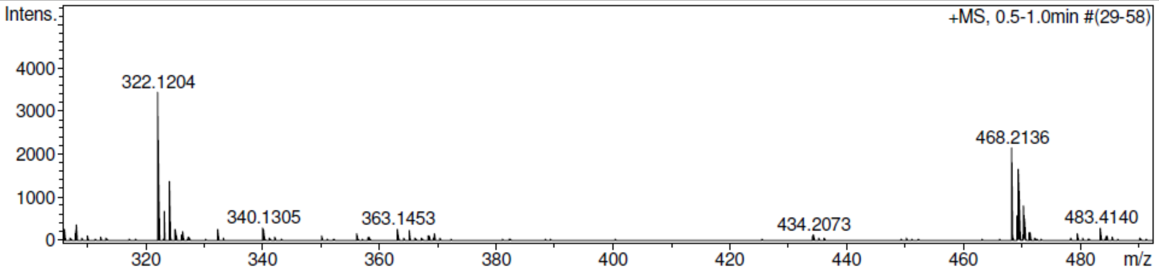

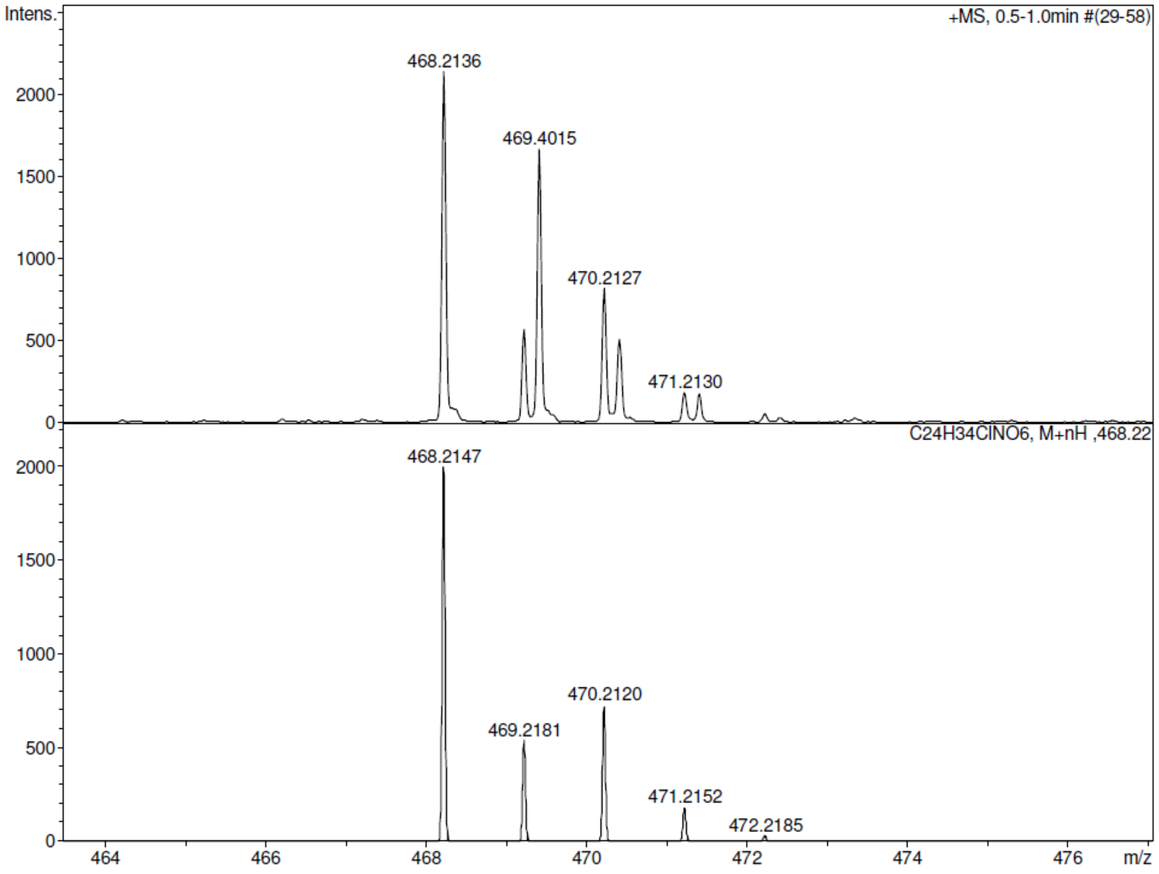

(1*S,1'*R**,2*S**,5*R**,7'*S**)-1',7'-dimethyl-2',3',5',6'-tetraoxa-11'-azaspiro[adamantane-2,4'-bicyclo[5.3.1]undecane], 3ac**

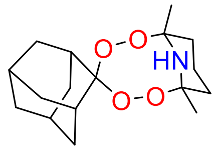

Display Report

Analysis Info

Analysis Name	D:\Data\Kolotyrkina\2024\Belyakova\0312022.d	Acquisition Date	12.03.2024 13:56:39
Method	tune_low.m	Operator	BDAL@DE
Sample Name	/IYAR BL-1604	Instrument / Ser#	micrOTOF 10248
Comment	C17H27NO4 mH 310.2012 calibrant added CH3CN		

Acquisition Parameter

Source Type	ESI	Ion Polarity	Positive	Set Nebulizer	0.4 Bar
Focus	Not active			Set Dry Heater	180 °C
Scan Begin	50 m/z	Set Capillary	4500 V	Set Dry Gas	4.0 l/min
Scan End	3000 m/z	Set End Plate Offset	-500 V	Set Divert Valve	Waste

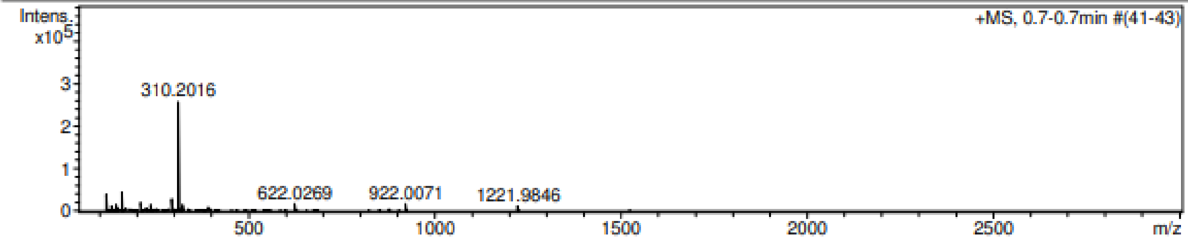

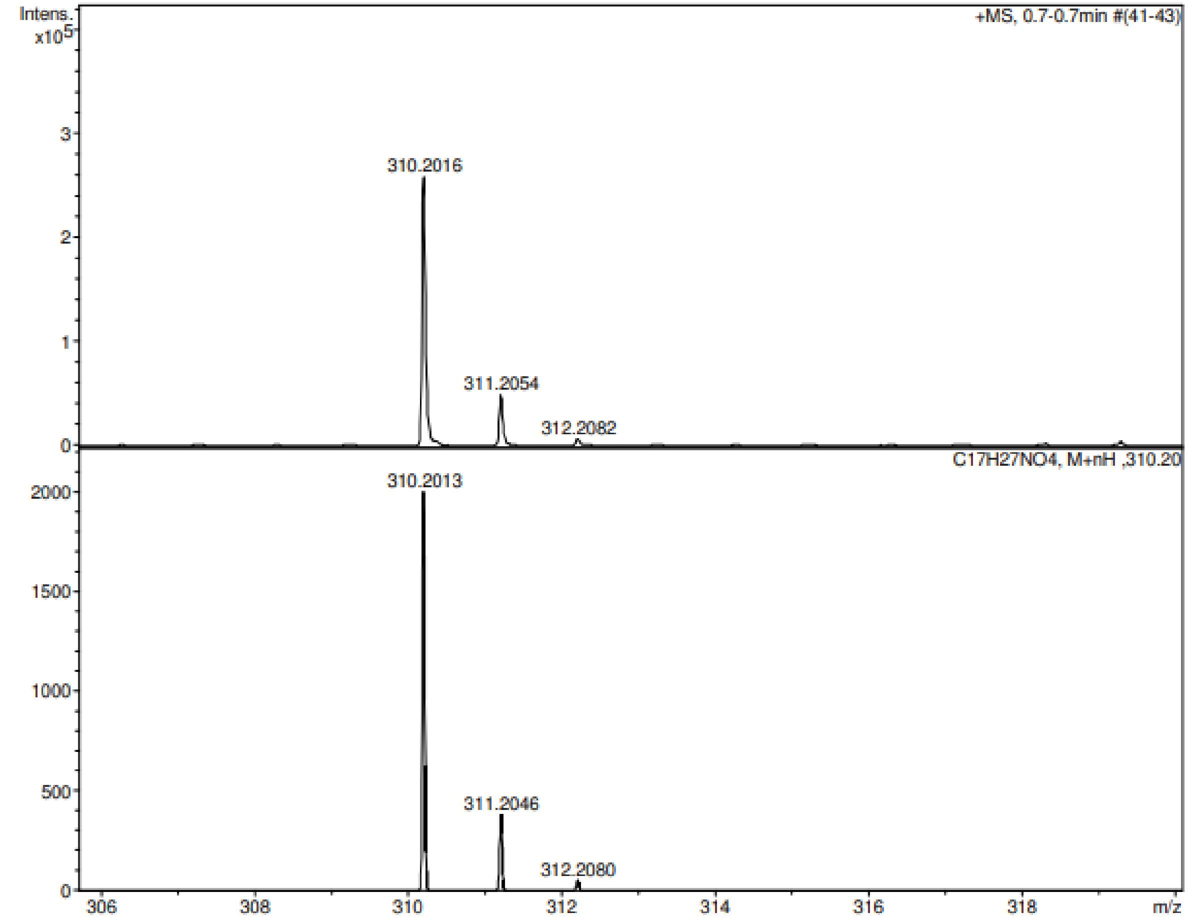

Bruker Compass DataAnalysis 4.0 printed: 12.03.2024 13:59:27 Page 1 of 1

Ethyl (1*S,1'*R**,2*R**,5*R**,7'*S**,8'*S**)-1',7',8'-trimethyl-2',3',5',6'-tetraoxa-11'-azaspiro[adamantane-2,4'-bicyclo[5.3.1]undecane]-8'-carboxylate, 3bc**

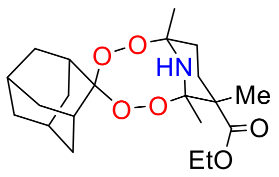

Display Report

Analysis Info

Analysis Name	D:\Data\Kolotyrkina\2024\Belyakova\0423038.d	Acquisition Date	23.04.2024 16:23:22
Method	tune_low.m	Operator	BDAL@DE
Sample Name	/IYAR BL-1362	Instrument / Ser#	micrOTOF 10248
Comment	C21H33NO6 mH396.2380 calibrant added CH3CN		

Acquisition Parameter

Source Type	ESI	Ion Polarity	Positive	Set Nebulizer	0.4 Bar
Focus	Not active			Set Dry Heater	180 °C
Scan Begin	50 m/z	Set Capillary	4500 V	Set Dry Gas	4.0 l/min
Scan End	3000 m/z	Set End Plate Offset	-500 V	Set Divert Valve	Waste

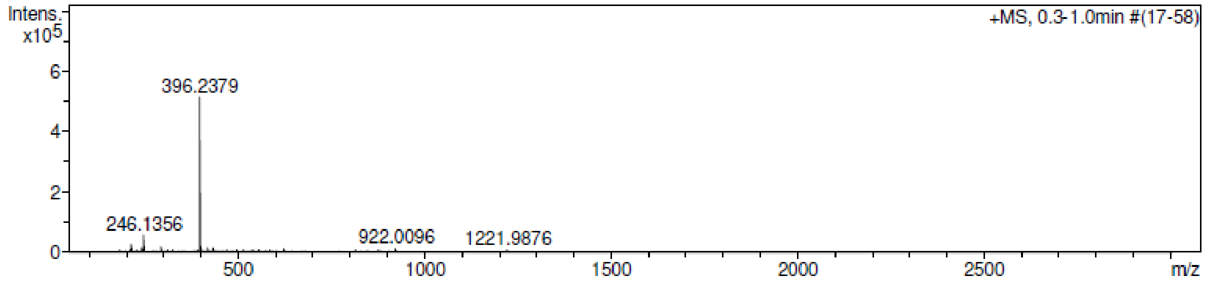

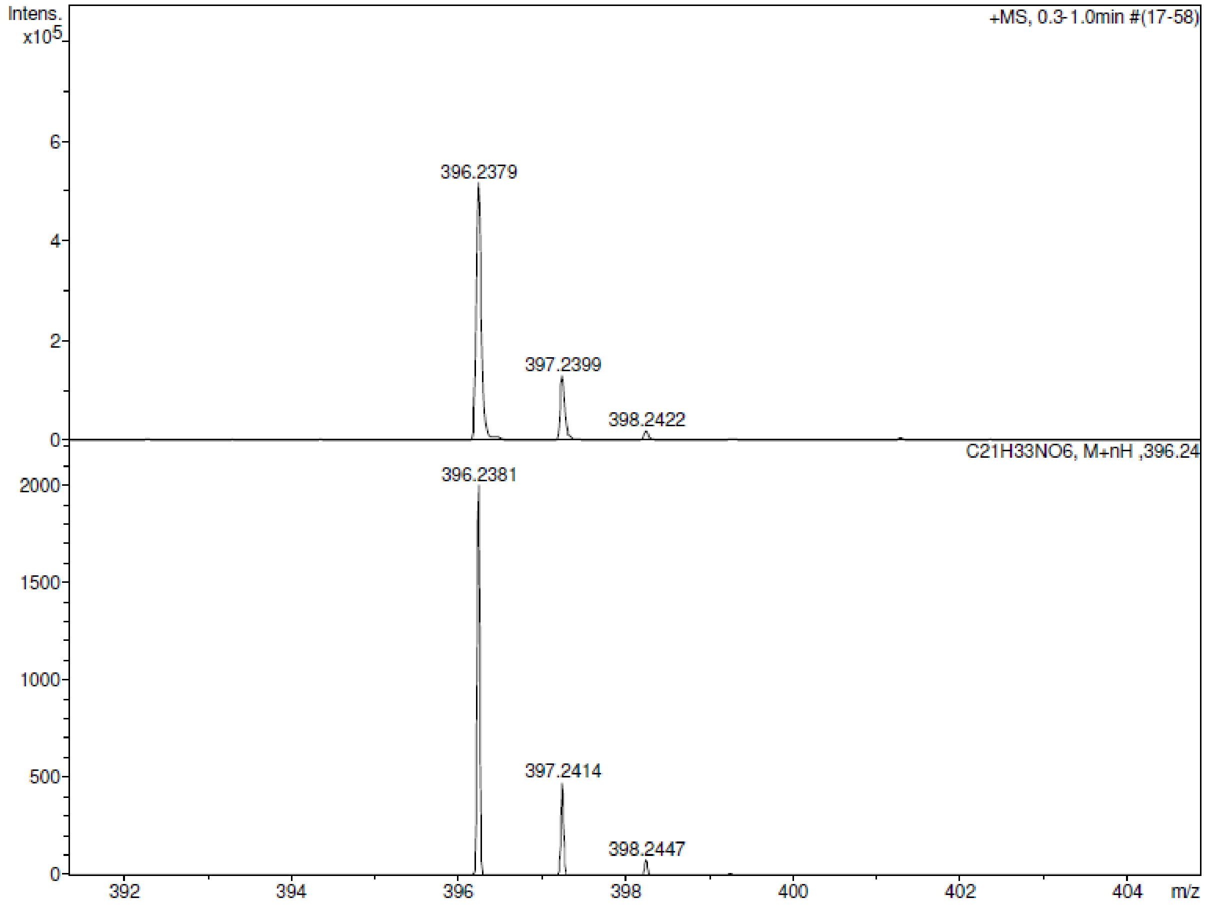

Ethyl (1*S,1'*R**,2*R**,5*R**,7'*S**,8'*S**)-8'-ethyl-1',7'-dimethyl-2',3',5',6'-tetraoxa-11'-azaspiro[adamantane-2,4'-bicyclo[5.3.1]undecane]-8'-carboxylate, 3cc**

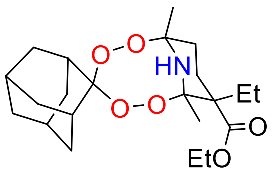

Display Report

Analysis Info

Analysis Name	D:\Data\Kolotyrkina\2024\Belyakova\0423042.d	Acquisition Date	23.04.2024 16:41:00
Method	tune_low.m	Operator	BDAL@DE
Sample Name	/IYAR BL-1361	Instrument / Ser#	micrOTOF 10248
Comment	C22H35NO6 mH410.2537calibrant added CH3CN		

Acquisition Parameter

Source Type	ESI	Ion Polarity	Positive	Set Nebulizer	0.4 Bar
Focus	Not active			Set Dry Heater	180 °C
Scan Begin	50 m/z	Set Capillary	4500 V	Set Dry Gas	4.0 l/min
Scan End	3000 m/z	Set End Plate Offset	-500 V	Set Divert Valve	Waste

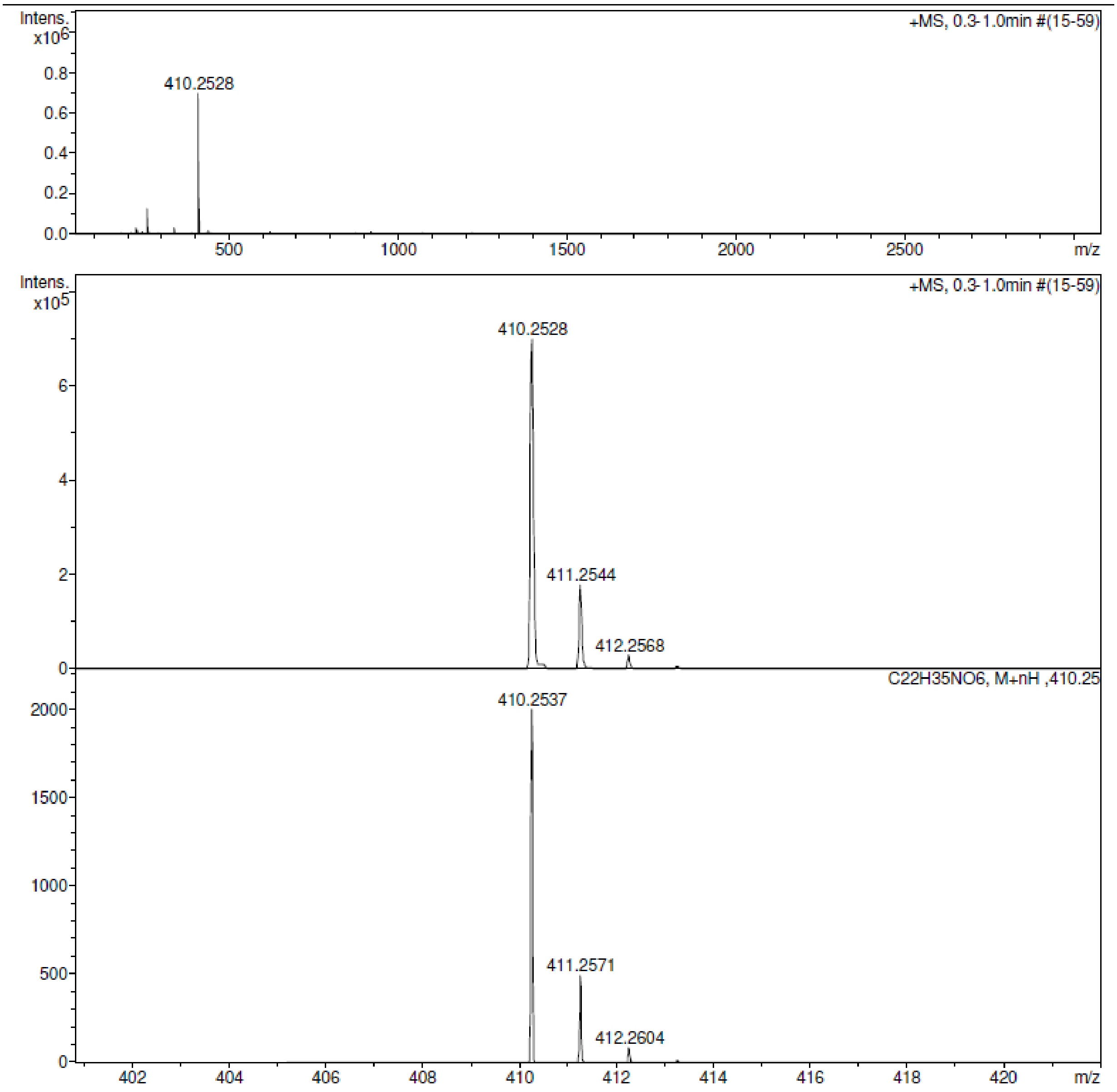

Bruker Compass DataAnalysis 4.0 printed: 23.04.2024 16:43:20 Page 1 of 1

Ethyl (1*S,1'*R**,2*R**,5*R**,7'*S**,8'*S**)-8'-butyl-1',7'-dimethyl-2',3',5',6'-tetraoxa-11'-azaspiro[adamantane-2,4'-bicyclo[5.3.1]undecane]-8'-carboxylate, 3dc**

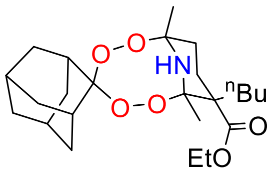

Display Report

Analysis Info

Analysis Name	D:\Data\Kolotyrkina\2024\Belyakova\0423040.d	Acquisition Date	23.04.2024 16:32:01
Method	tune_low.m	Operator	BDAL@DE
Sample Name	/IYAR BL-1358	Instrument / Ser#	micrOTOF 10248
Comment	C24H39NO6 mH438.2850 calibrant added CH3CN		

Acquisition Parameter

Source Type	ESI	Ion Polarity	Positive	Set Nebulizer	0.4 Bar
Focus	Not active			Set Dry Heater	180 °C
Scan Begin	50 m/z	Set Capillary	4500 V	Set Dry Gas	4.0 l/min
Scan End	3000 m/z	Set End Plate Offset	-500 V	Set Divert Valve	Waste

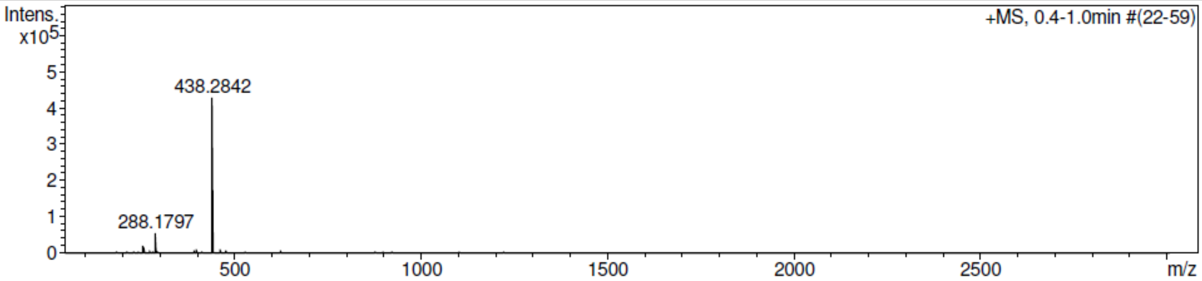

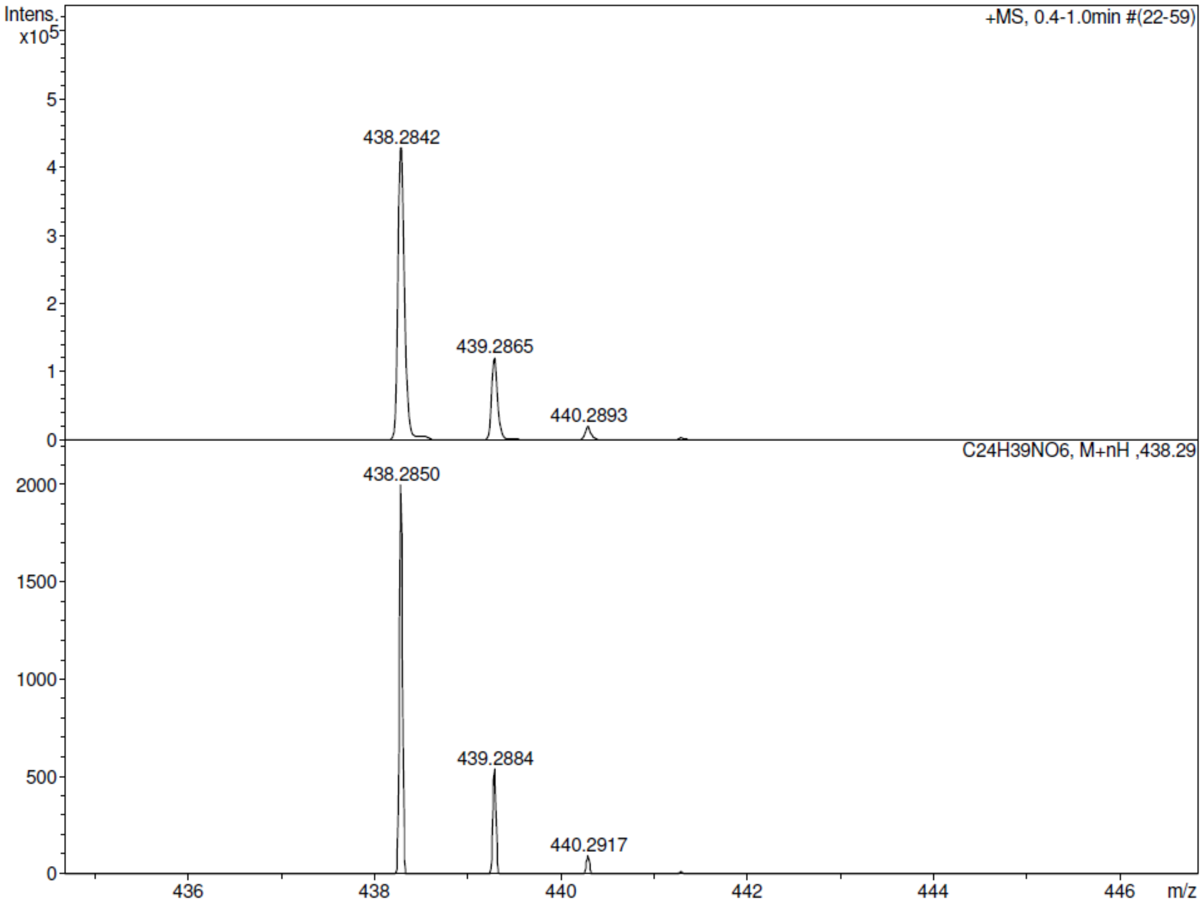

Ethyl (1*S,1'*R**,2*R**,5*R**,7'*S**,8'*R**)-8'-(3-ethoxy-3-oxopropyl)-1',7'-dimethyl-2',3',5',6'-tetraoxa-11'-azaspiro[adamantane-2,4'-bicyclo[5.3.1]undecane]-8'-carboxylate, 3ec**

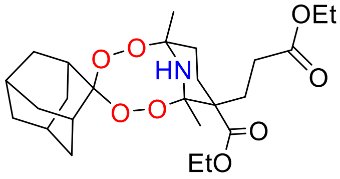

Display Report

Analysis Info

Analysis Name	D:\Data\Kolotyrkina\2024\Belyakova\0423043.d	Acquisition Date	23.04.2024 16:45:36
Method	tune_low.m	Operator	BDAL@DE
Sample Name	/IYAR BL-1369	Instrument / Ser#	micrOTOF 10248
Comment	C25H39NO8 mH482.2748 calibrant added CH3CN		

Acquisition Parameter

Source Type	ESI	Ion Polarity	Positive	Set Nebulizer	0.4 Bar
Focus	Not active			Set Dry Heater	180 °C
Scan Begin	50 m/z	Set Capillary	4500 V	Set Dry Gas	4.0 l/min
Scan End	3000 m/z	Set End Plate Offset	-500 V	Set Divert Valve	Waste

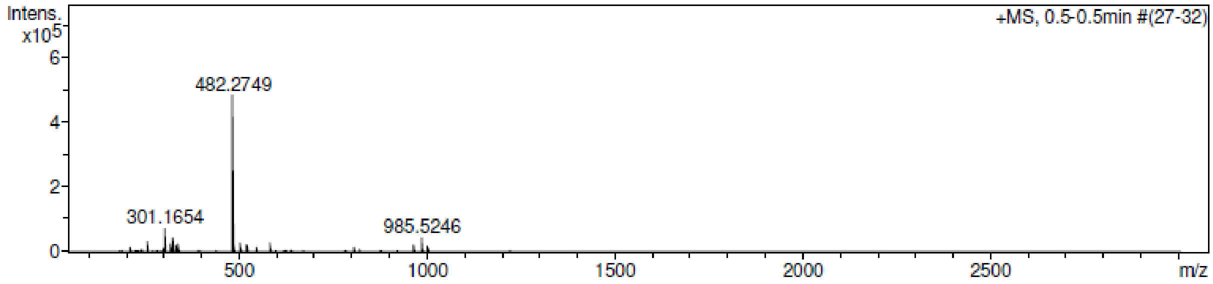

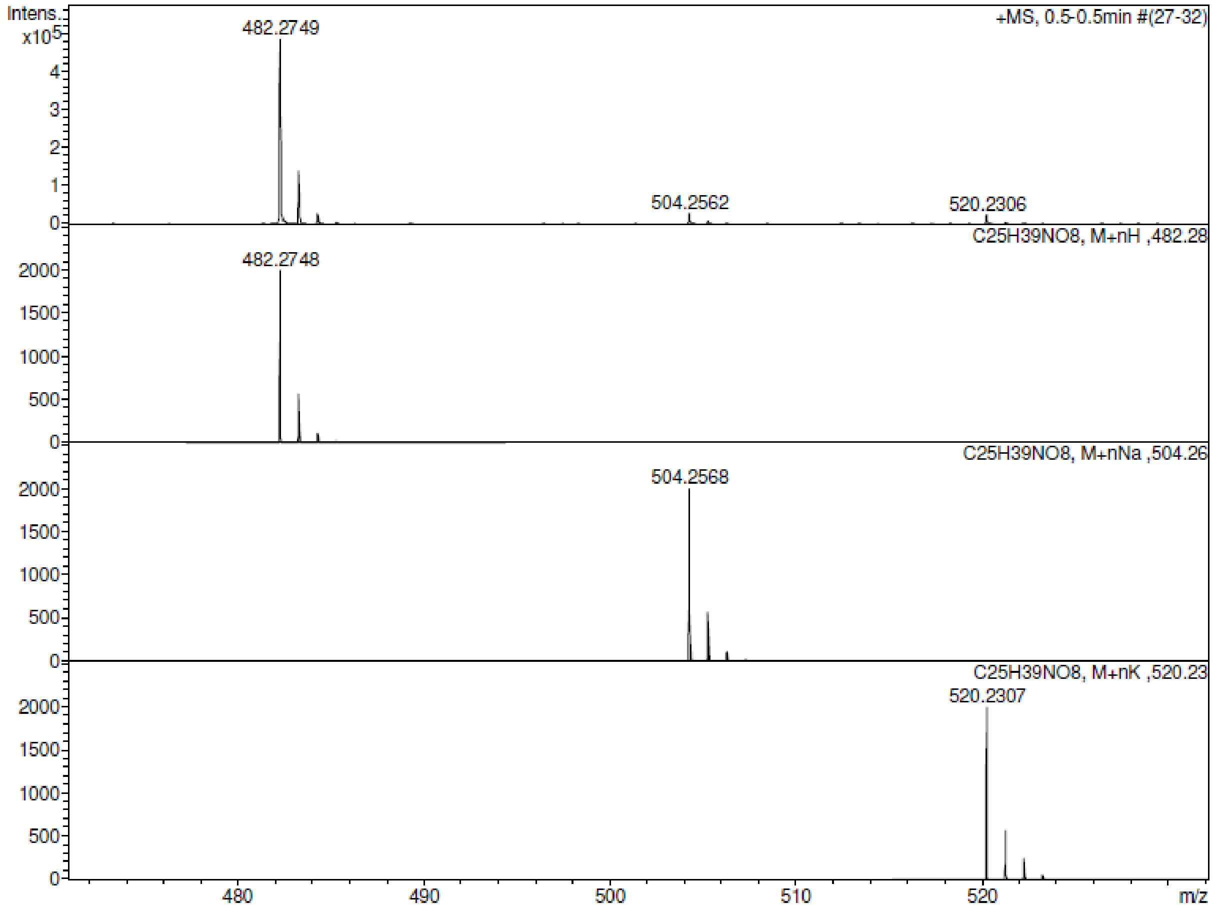

Ethyl (1*S,1'*R**,2*R**,5*R**,7'*S**,8'*R**)-8'-allyl-1',7'-dimethyl-2',3',5',6'-tetraoxa-11'-azaspiro[adamantane-2,4'-bicyclo[5.3.1]undecane]-8'-carboxylate, 3fc**

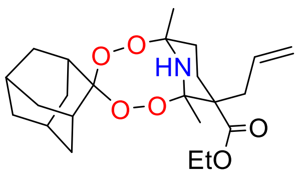

Display Report

Analysis Info

Analysis Name	D:\Data\Chizhov\Terentiev\Belyakova\bl-1324_&clb.d	Acquisition Date	25.03.2022 14:08:58
Method	tune_wide.m	Operator	BDAL@DE
Sample Name	/TERN BL-1324	Instrument / Ser#	micrOTOF 10248
Comment	CH3CN 100 %, dil. 200, calibrant added		

Acquisition Parameter

Source Type	ESI	Ion Polarity	Positive	Set Nebulizer	0.4 Bar
Focus	Not active			Set Dry Heater	180 °C
Scan Begin	50 m/z	Set Capillary	4500 V	Set Dry Gas	4.0 l/min
Scan End	3000 m/z	Set End Plate Offset	-500 V	Set Divert Valve	Waste

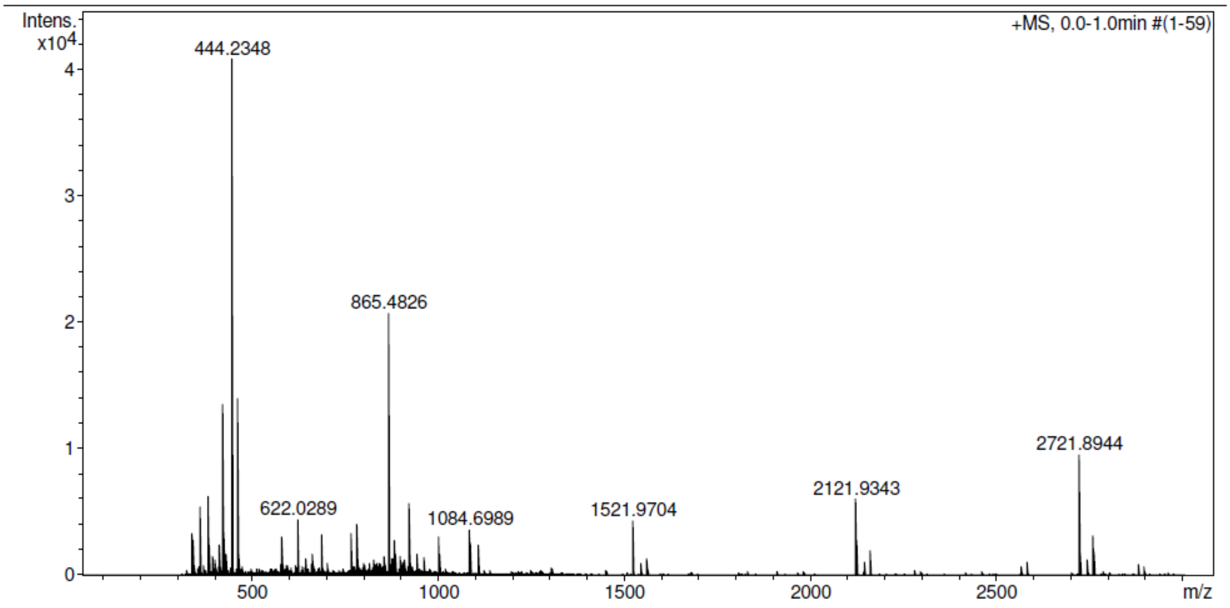

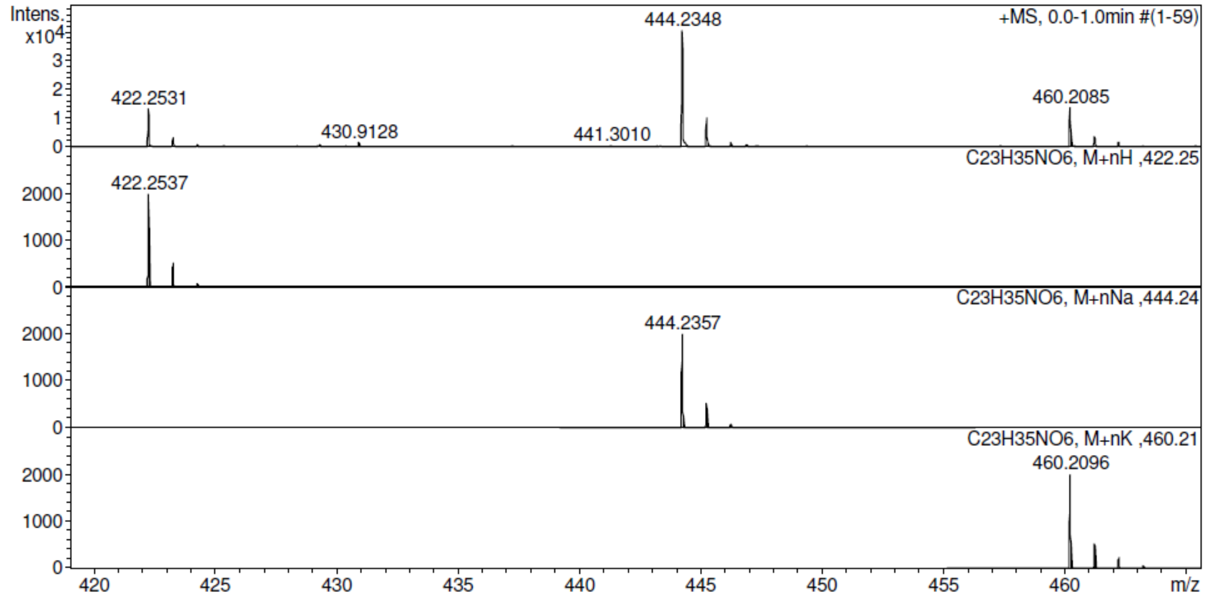

Ethyl (1*S,1'*R**,2*R**,5*R**,7'*S**,8'*R**)-1',7'-dimethyl-8'-(prop-2-yn-1-yl)-2',3',5',6'-tetraoxa-11'-azaspiro[adamantane-2,4'-bicyclo[5.3.1]undecane]-8'-carboxylate, 3gc**

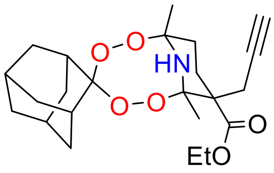

Display Report

Analysis Info

Analysis Name	D:\Data\Chizhov\Terentiev\Belyakova\bl-1353_&clb.d	Acquisition Date	28.03.2022 14:08:54
Method	tune_wide.m	Operator	BDAL@DE
Sample Name	/TERN BL-1353	Instrument / Ser#	micrOTOF 10248
Comment	CH3CN 100 %, dil. 200, calibrant added		

Acquisition Parameter

Source Type	ESI	Ion Polarity	Positive	Set Nebulizer	0.4 Bar
Focus	Not active			Set Dry Heater	180 °C
Scan Begin	50 m/z	Set Capillary	4500 V	Set Dry Gas	4.0 l/min
Scan End	3000 m/z	Set End Plate Offset	-500 V	Set Divert Valve	Waste

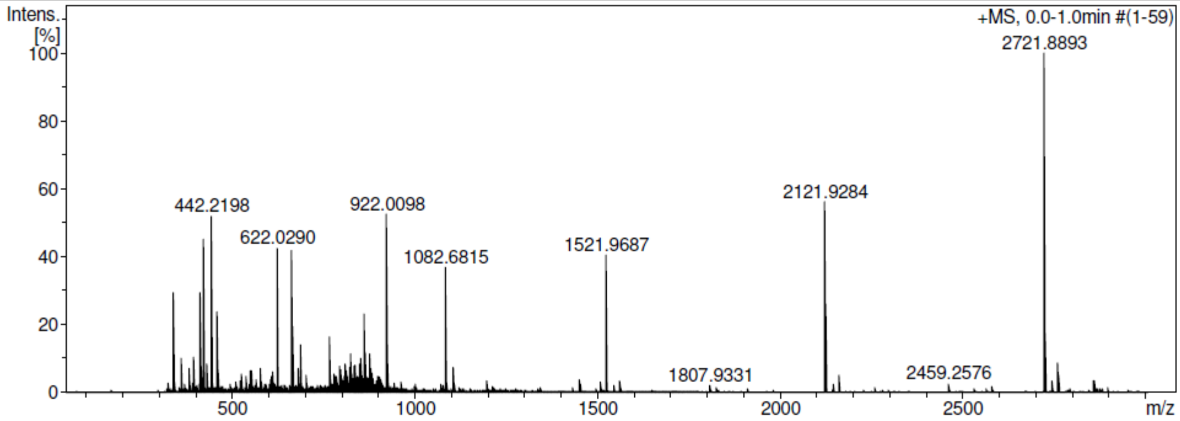

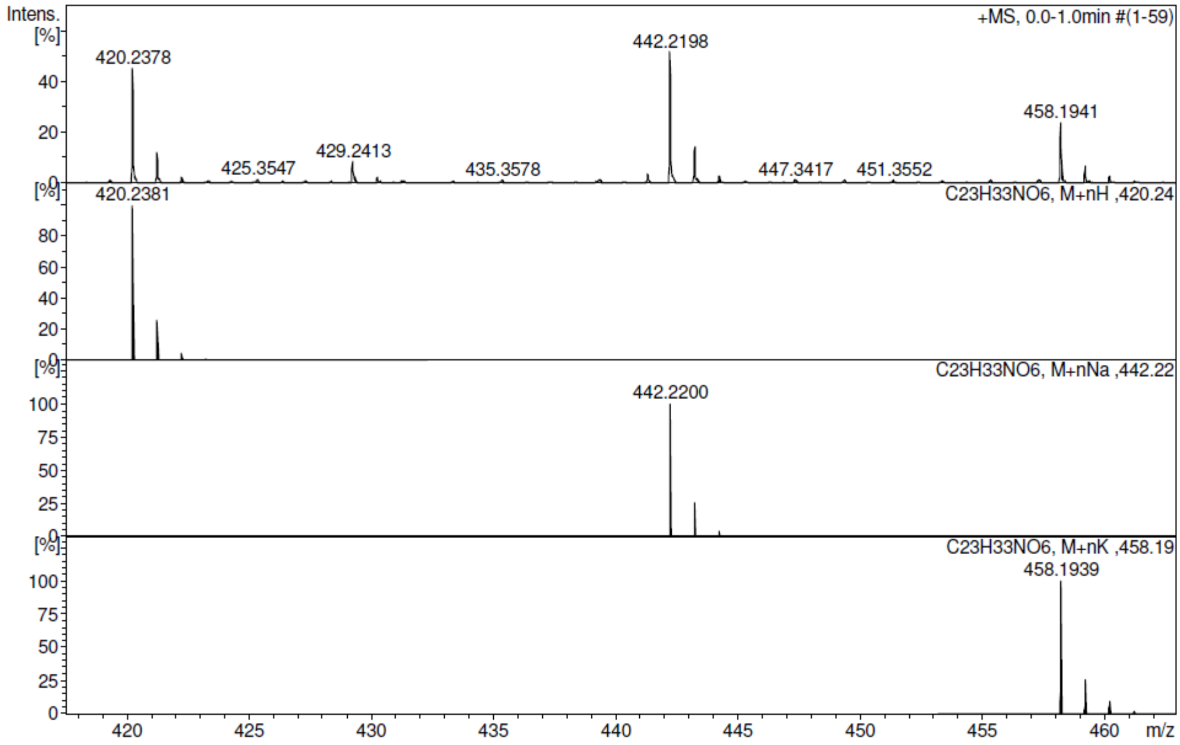

Ethyl (1*S,1'*R**,2*R**,5*R**,7'*S**,8'*R**)-8'-benzyl-1',7'-dimethyl-2',3',5',6'-tetraoxa-11'-azaspiro[adamantane-2,4'-bicyclo[5.3.1]undecane]-8'-carboxylate, 3hc**

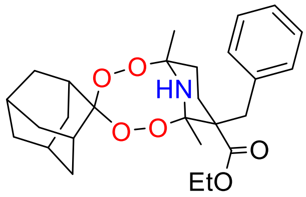

Display Report

Analysis Info

Analysis Name	D:\Data\Chizhov\Terentiev\Belyakova\bl-1351_&clb.d	Acquisition Date	25.03.2022 14:23:28
Method	tune_wide.m	Operator	BDAL@DE
Sample Name	/TERN BL-1351	Instrument / Ser#	micrOTOF 10248
Comment	CH3CN 100 %, dil. 200, calibrant added		

Acquisition Parameter

Source Type	ESI	Ion Polarity	Positive	Set Nebulizer	0.4 Bar
Focus	Not active			Set Dry Heater	180 °C
Scan Begin	50 m/z	Set Capillary	4500 V	Set Dry Gas	4.0 l/min
Scan End	3000 m/z	Set End Plate Offset	-500 V	Set Divert Valve	Waste

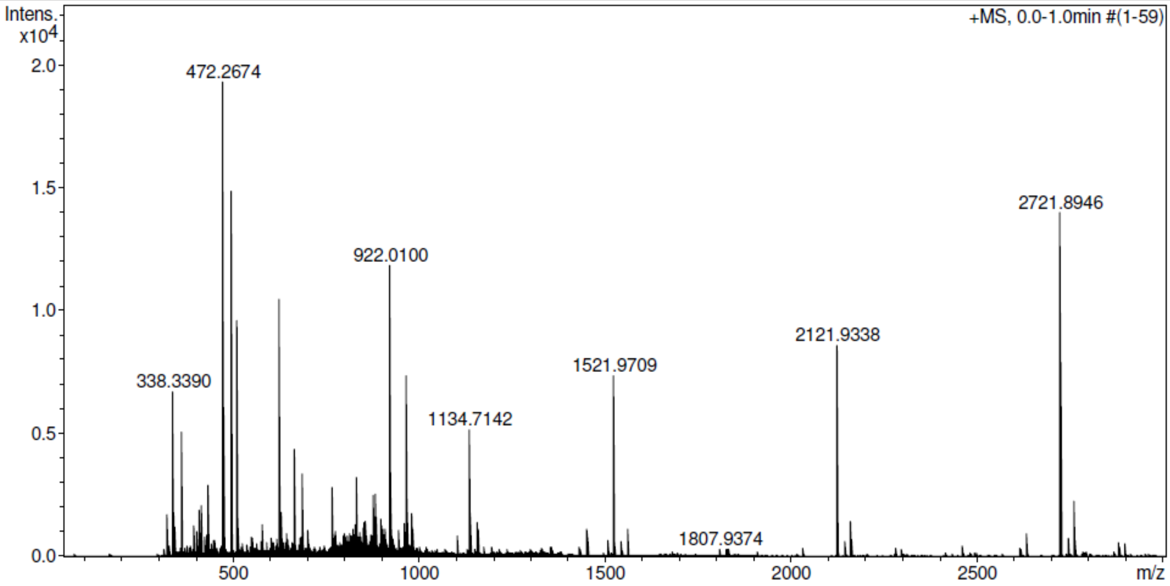

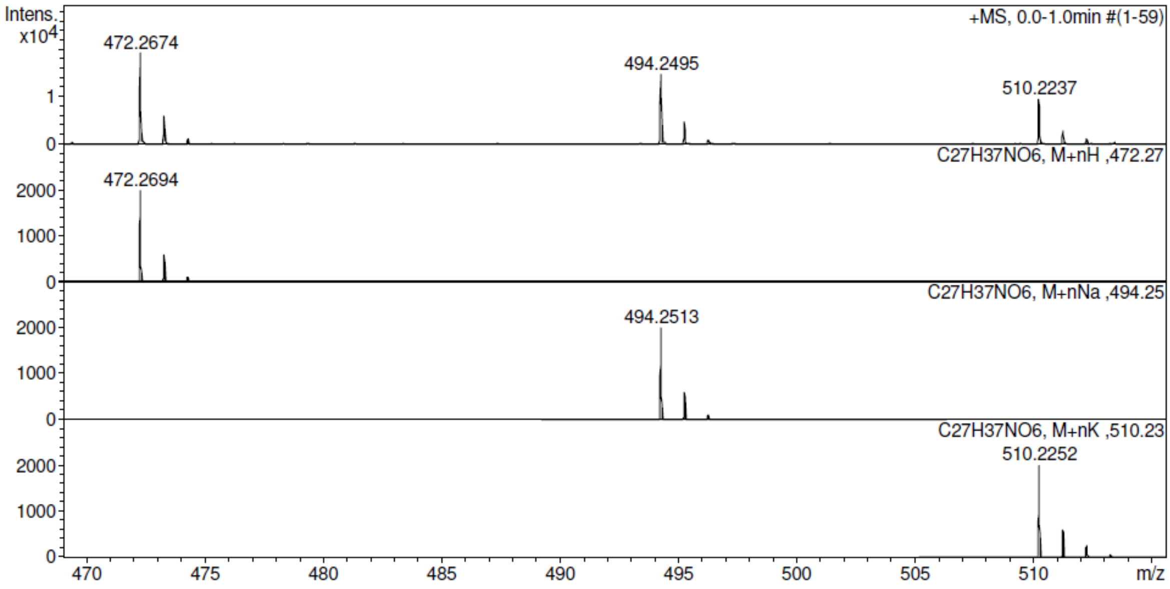

Bruker Compass DataAnalysis 4.0 printed: 25.03.2022 14:34:59 Page 1 of 1

Ethyl (1*S,1'*R**,2*R**,5*R**,7'*S**,8'*R**)-8'-(4-(tert-butyl)benzyl)-1',7'-dimethyl-2',3',5',6'-tetraoxa-11'-azaspiro[adamantane-2,4'-bicyclo[5.3.1]undecane]-8'-carboxylate, 3jc**

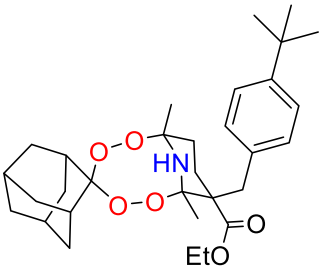

Display Report

Analysis Info

Analysis Name	D:\Data\Kolotyrkina\2024\Belyakova\0423039.d	Acquisition Date	23.04.2024 16:27:46
Method	tune_low.m	Operator	BDAL@DE
Sample Name	/IYAR BL-1367	Instrument / Ser#	micrOTOF 10248
Comment	C31H45NO6 mH528.3319 calibrant added CH3CN		

Acquisition Parameter

Source Type	ESI	Ion Polarity	Positive	Set Nebulizer	0.4 Bar
Focus	Not active			Set Dry Heater	180 °C
Scan Begin	50 m/z	Set Capillary	4500 V	Set Dry Gas	4.0 l/min
Scan End	3000 m/z	Set End Plate Offset	-500 V	Set Divert Valve	Waste

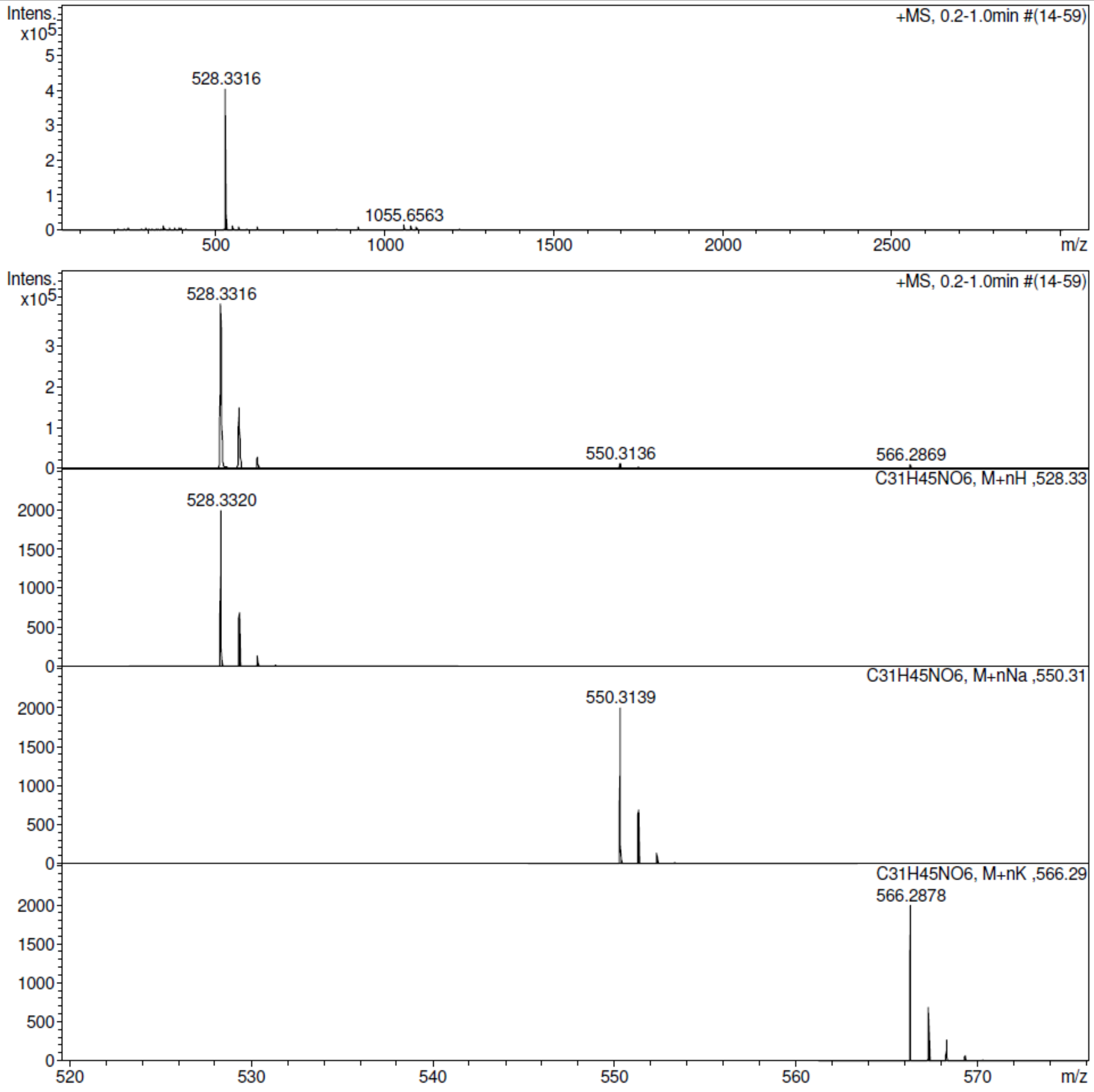

Ethyl (1S*,1'R*,2R*,5R*,7'S*,8'R*)-8'-(4-fluorobenzyl)-1',7'-dimethyl-2',3',5',6'-tetraoxa-11'-azaspiro[adamantane-2,4'-bicyclo[5.3.1]undecane]-8'-carboxylate, 3kc

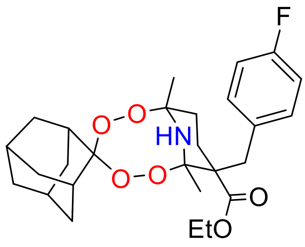

Display Report

Analysis Info

Analysis Name	D:\Data\Chizhov\Terentiev\Belyakova\bl-1350_&clb.d	Acquisition Date	25.03.2022 14:18:14
Method	tune_wide.m	Operator	BDAL@DE
Sample Name	/TERN BL-1350	Instrument / Ser#	micrOTOF 10248
Comment	CH3CN 100 %, dil. 200, calibrant added		

Acquisition Parameter

Source Type	ESI	Ion Polarity	Positive	Set Nebulizer	0.4 Bar
Focus	Not active			Set Dry Heater	180 °C
Scan Begin	50 m/z	Set Capillary	4500 V	Set Dry Gas	4.0 l/min
Scan End	3000 m/z	Set End Plate Offset	-500 V	Set Divert Valve	Waste

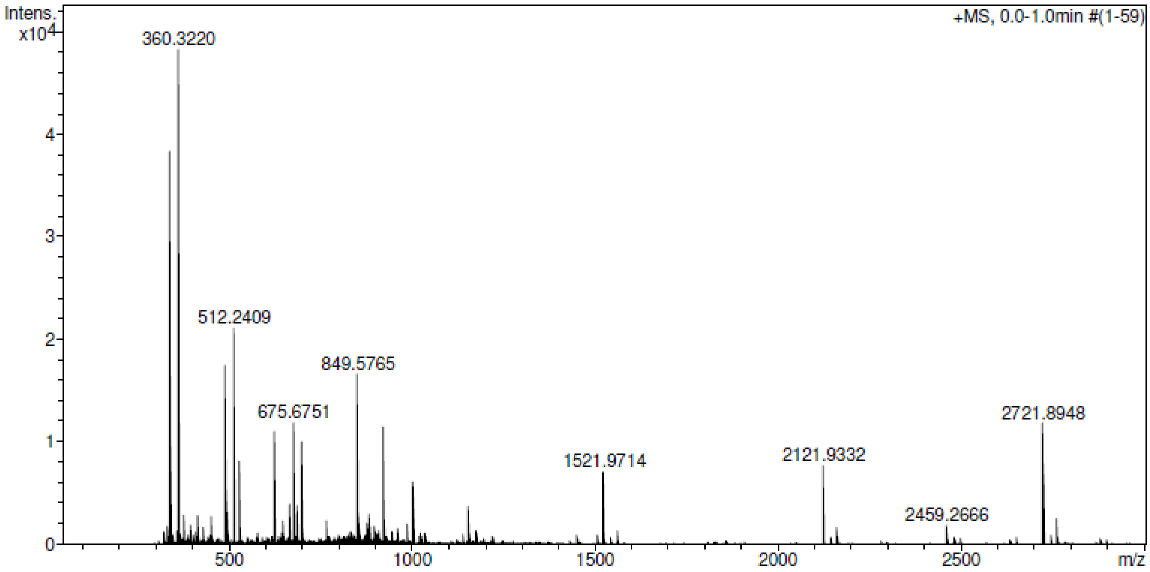

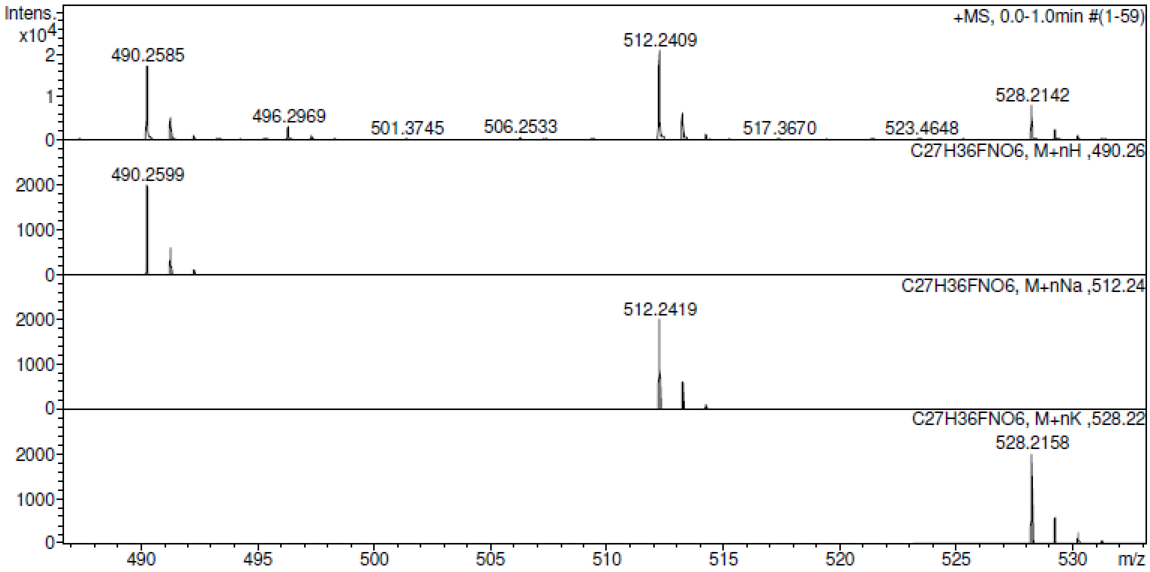

Ethyl (1*S,1'*R**,2*R**,5*R*,7'*S**,8'*R**)-8'-(4-chlorobenzyl)-1',7'-dimethyl-2',3',5',6'-tetraoxa-11'-azaspiro[adamantane-2,4'-bicyclo[5.3.1]undecane]-8'-carboxylate, 3lc**

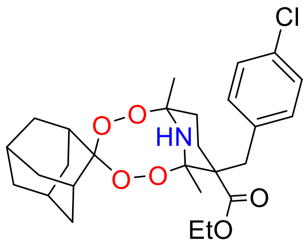

Display Report

Analysis Info

Analysis Name	D:\Data\Chizhov\Terentiev\Belyakova\bl-1289_&clblow.d	Acquisition Date	27.01.2022 16:46:07
Method	tune_low.m	Operator	BDAL@DE
Sample Name	/TERN BL-1289	Instrument / Ser#	micrOTOF 10248
Comment	CH3CN 100 %, dil. 2000, calibrant added		

Acquisition Parameter

Source Type	ESI	Ion Polarity	Positive	Set Nebulizer	0.4 Bar
Focus	Not active			Set Dry Heater	180 °C
Scan Begin	50 m/z	Set Capillary	4500 V	Set Dry Gas	4.0 l/min
Scan End	3000 m/z	Set End Plate Offset	-500 V	Set Divert Valve	Waste

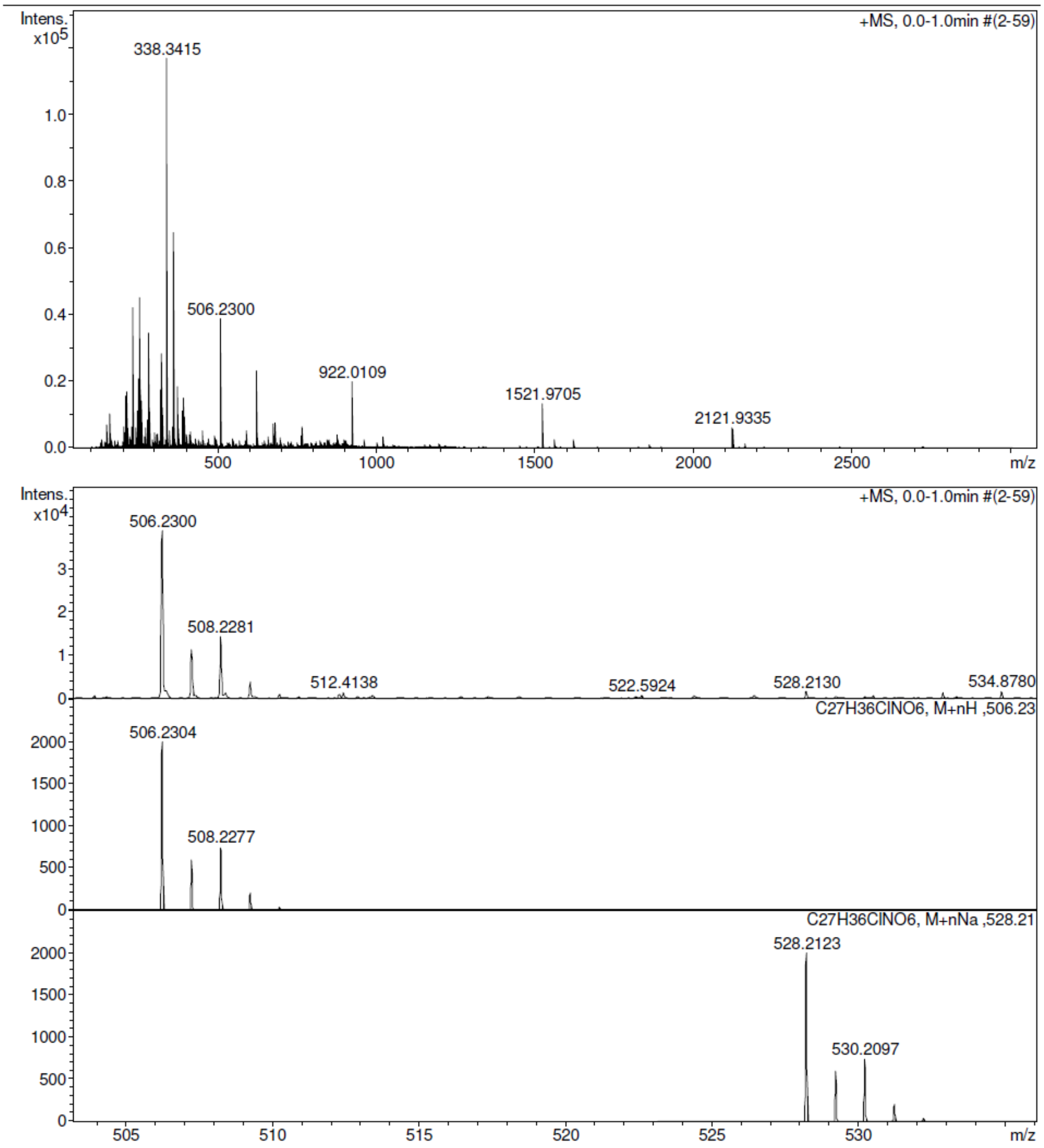

Allyl (1*S,1'*R**,2*R**,5*R**,7'*S**,8'*R**)-8'-allyl-1',7'-dimethyl-2',3',5',6'-tetraoxa-11'-azaspiro[adamantane-2,4'-bicyclo[5.3.1]undecane]-8'-carboxylate, 3mc**

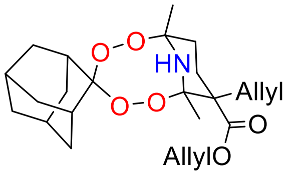

Display Report

Analysis Info

Analysis Name	D:\Data\Kolotyrkina\2024\Belyakova\0423041.d
Method	tune_low.m
Sample Name	/IYAR BL-1355
Comment	C24H35NO6 mH434.2537calibrant added CH3CN
Acquisition Date	23.04.2024 16:36:22
Operator	BDAL@DE
Instrument / Ser#	micrOTOF 10248

Acquisition Parameter

Source Type	ESI	Ion Polarity	Positive	Set Nebulizer	0.4 Bar
Focus	Not active			Set Dry Heater	180 °C
Scan Begin	50 m/z	Set Capillary	4500 V	Set Dry Gas	4.0 l/min
Scan End	3000 m/z	Set End Plate Offset	-500 V	Set Divert Valve	Waste

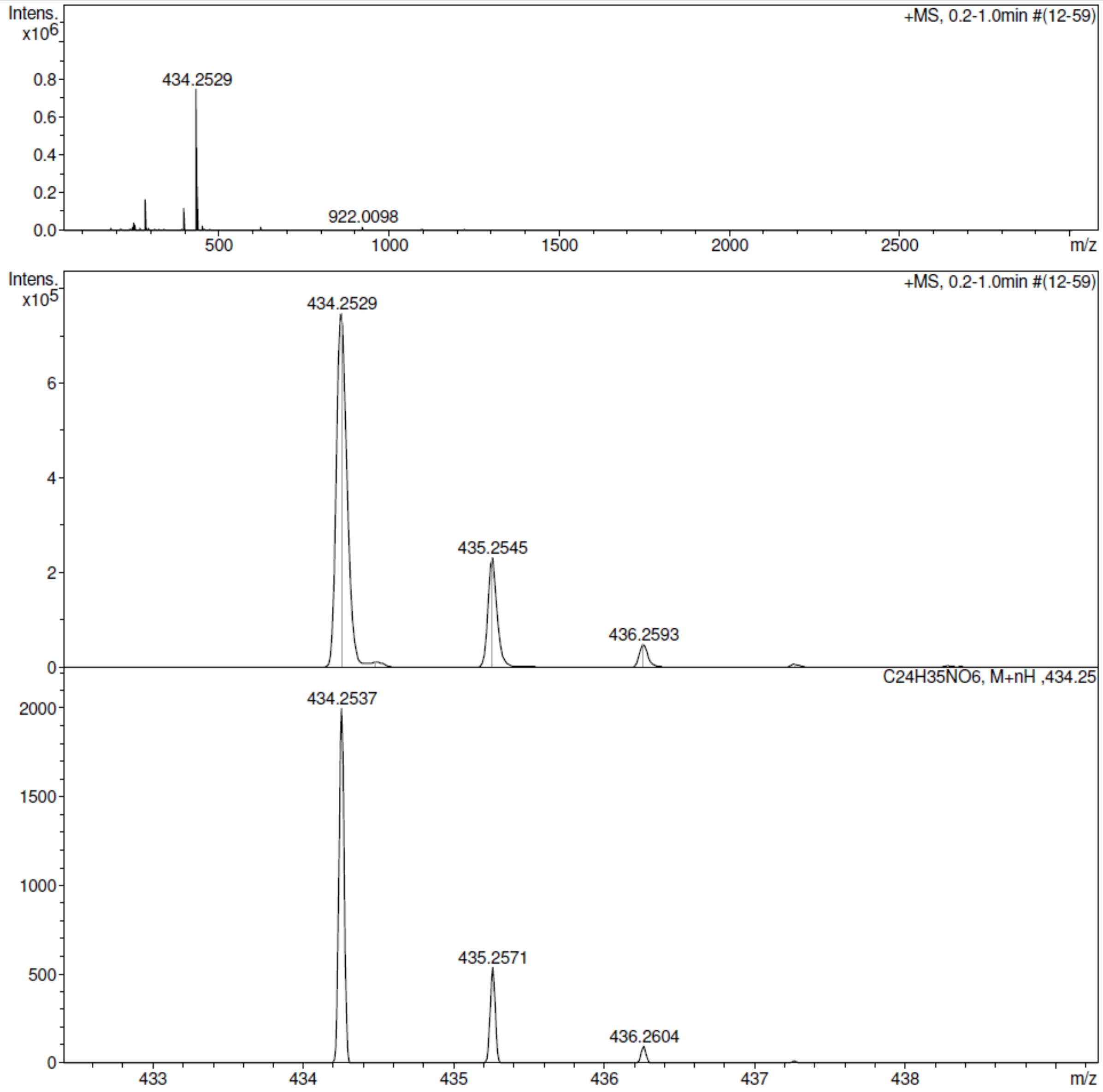

Benzyl (1*S,1'*R**,2*R**,5*R**,7'*S**,8'*R**)-8'-allyl-1',7'-dimethyl-2',3',5',6'-tetraoxa-11'-azaspiro[adamantane-2,4'-bicyclo[5.3.1]undecane]-8'-carboxylate, 3nc**

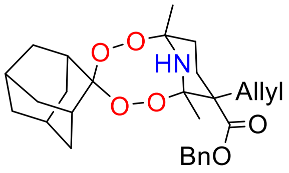

Display Report

Analysis Info

Analysis Name	D:\Data\Kolotyrkina\2022\Belyakova\0331030.d
Method	tune_50-1600_pos_15_12.m
Sample Name	/TERN BL-1359
Comment	C28H37NO6 mH 484.2693 calibrant added CH3CN

Acquisition Date	31.03.2022 15:03:42	
Operator	BDAL@DE	
Instrument / Ser#	micrOTOF	10248

Acquisition Parameter

Source Type	ESI	Ion Polarity	Positive	Set Nebulizer	0.4 Bar
Focus	Not active			Set Dry Heater	180 °C
Scan Begin	50 m/z	Set Capillary	4500 V	Set Dry Gas	4.0 l/min
Scan End	1600 m/z	Set End Plate Offset	-500 V	Set Divert Valve	Waste

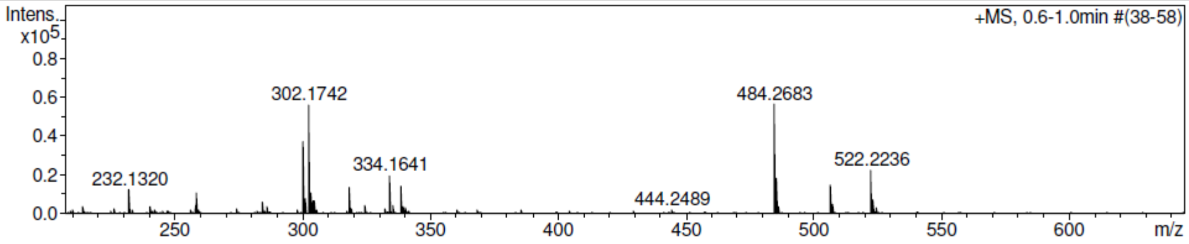

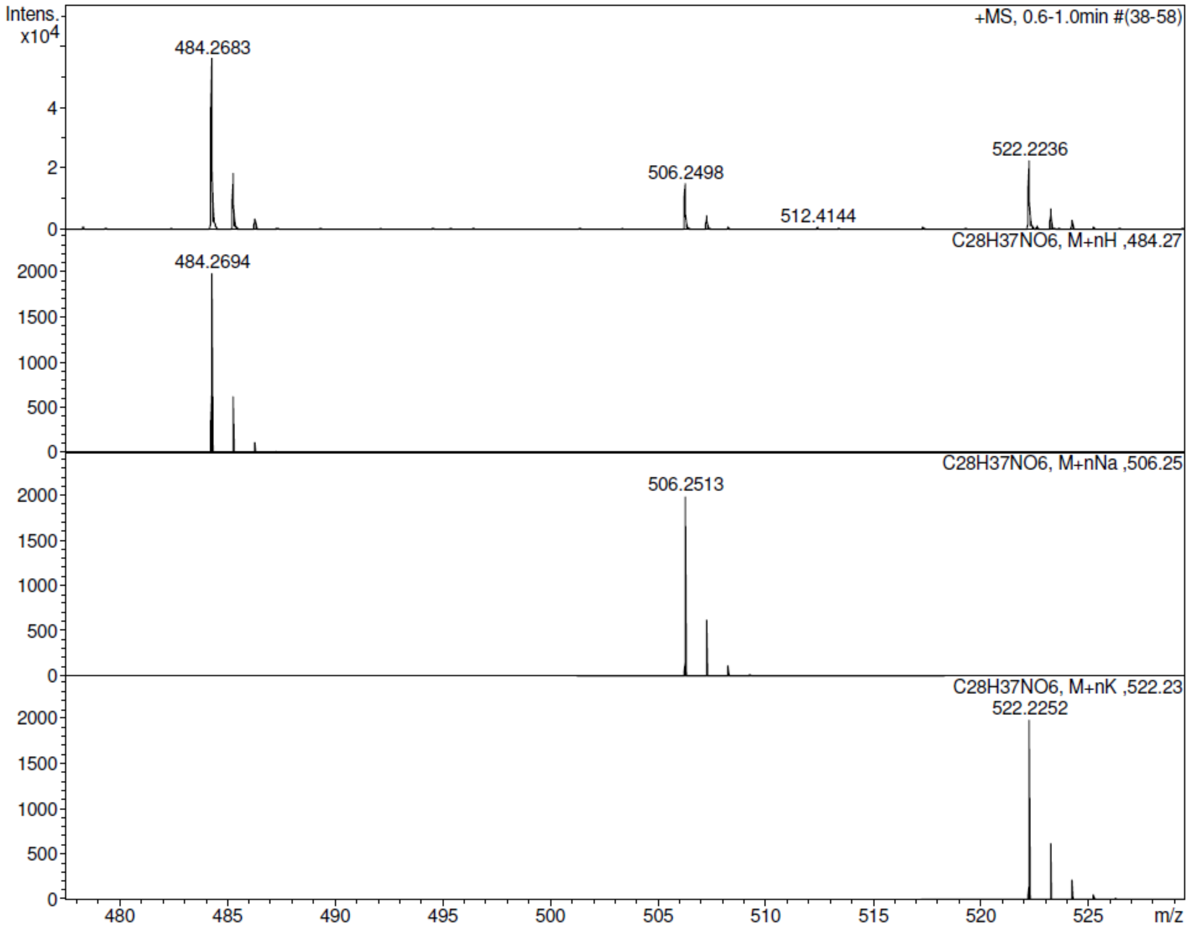

Bruker Compass DataAnalysis 4.0 printed: 31.03.2022 15:08:20 Page 1 of 1

HRMS spectra of intermediates 4, 5, 6, and 8/8' or products 3/3'

High-resolution mass spectra were recorded on a Bruker solariX XR instrument (ECR mass analyzer, superconducting magnet with a field strength of 15 T) (Germany), equipped with an electrospray ionization source (ESI). The scanning range of m / z 50–1500. The number of scans was 16, the number of points was 2 M. External calibration of the mass scale was performed using a sodium trifluoroacetate solution (0.1 mg / mL in a 1:1 acetonitrile : water mixture). Samples were introduced using a Hamilton RN 1750 syringe (Switzerland) with a capacity of 500 µL. Measurements were carried out in the positive ion (+) registration mode (grounded spray needle, high-voltage capillary - 4500 V; potential difference with a protective spray screen -500 V). The injection flow rate was controlled by a syringe pump (3 µL/min). Nitrogen was used as a nebulizer gas (1.0 bar) and a drying gas (4.0 L/min, 200°C). Data were processed using Bruker Data Analysis 5.0 software.

HRMS spectra of intermediate 4 or 5

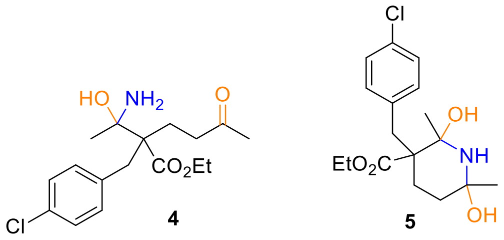

Generic Display Report (all)

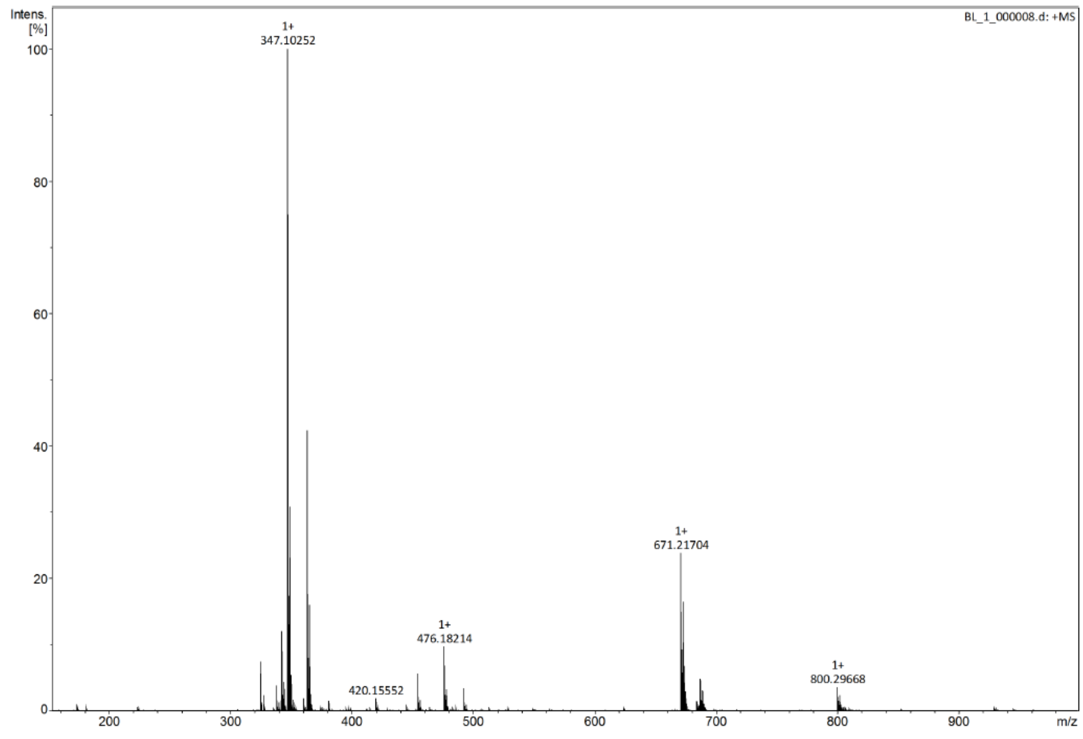

Bruker Compass DataAnalysis 5.0 printed: 11/12/2025 6:34:24 PM by: demo Page 1 of 1

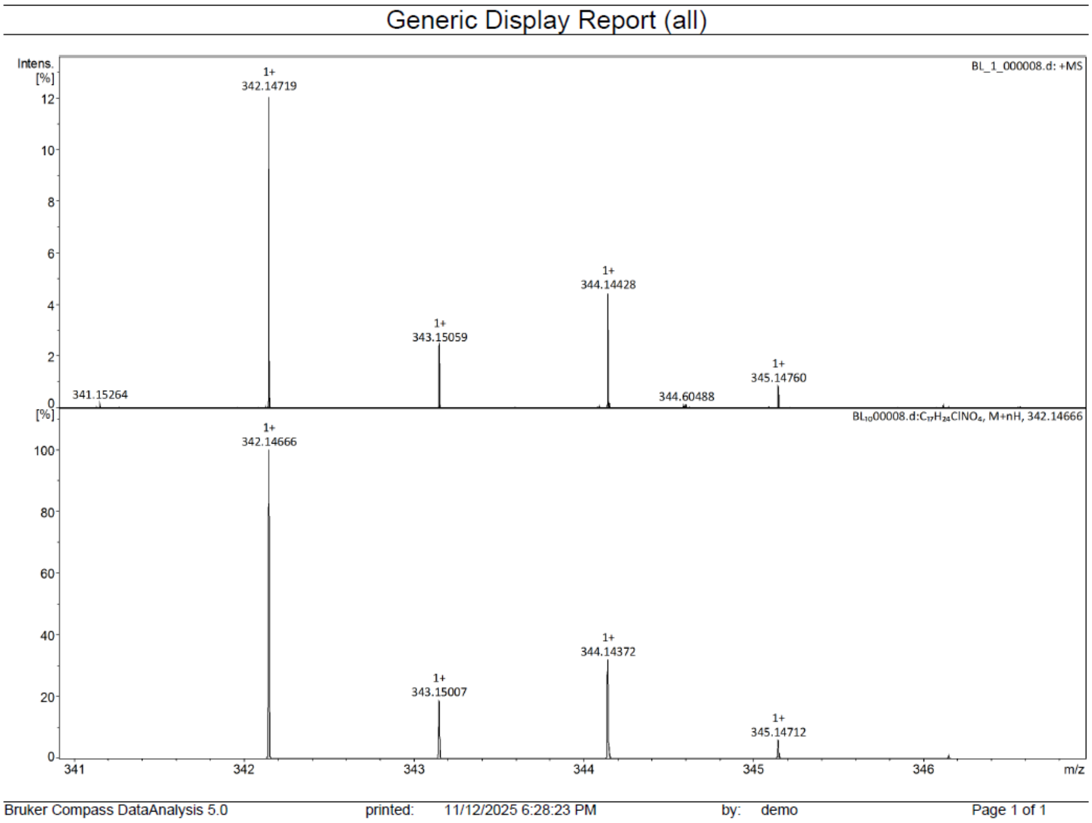

HRMS spectra of intermediate 6

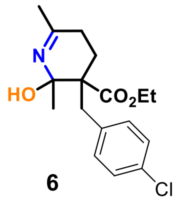

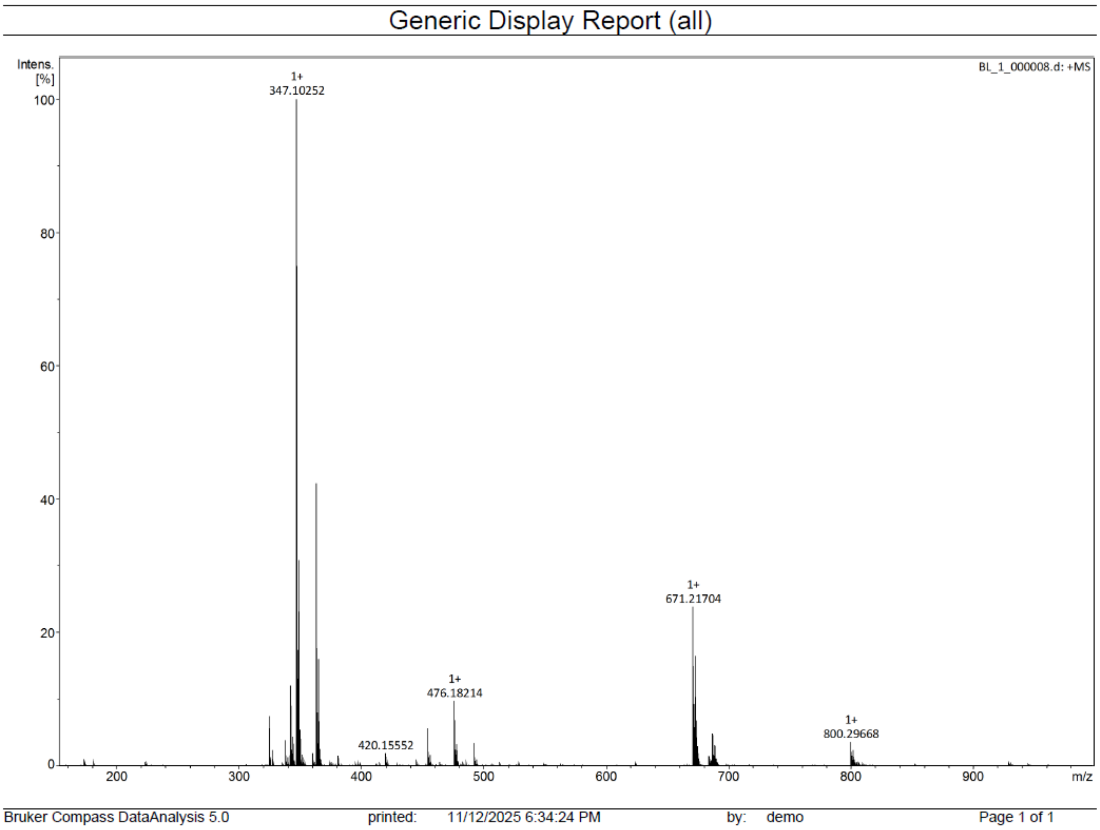

Generic Display Report (all)

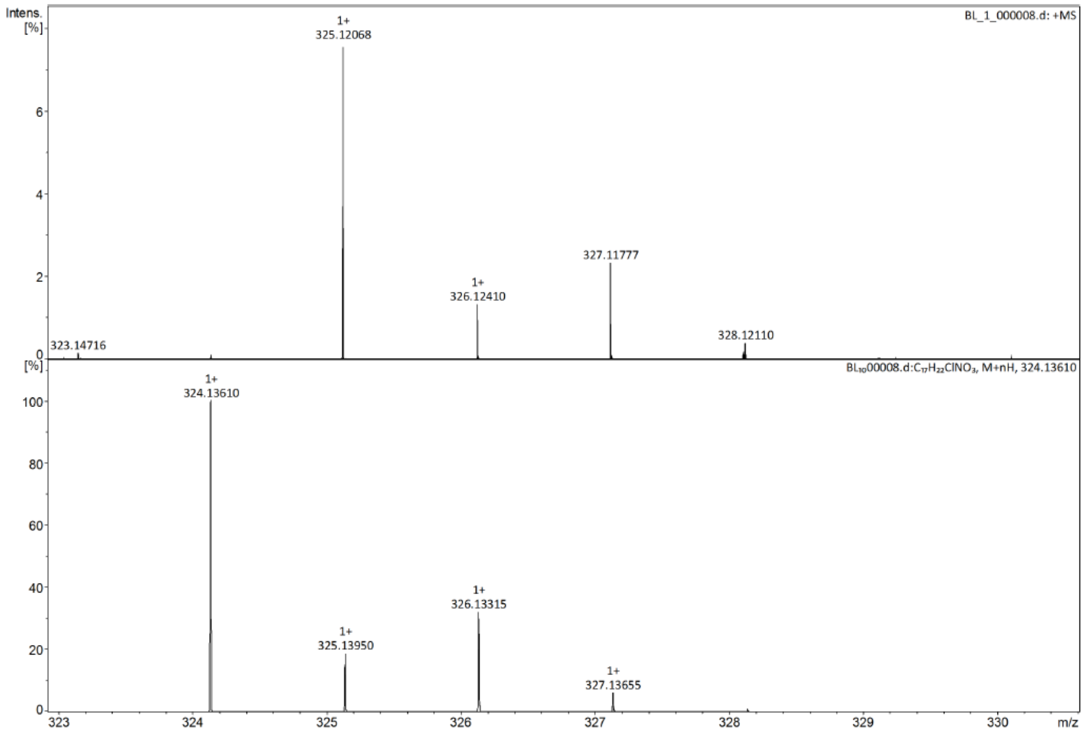

Bruker Compass DataAnalysis 5.0 printed: 11/12/2025 6:32:10 PM by: demo Page 1 of 1

Generic Display Report (all)

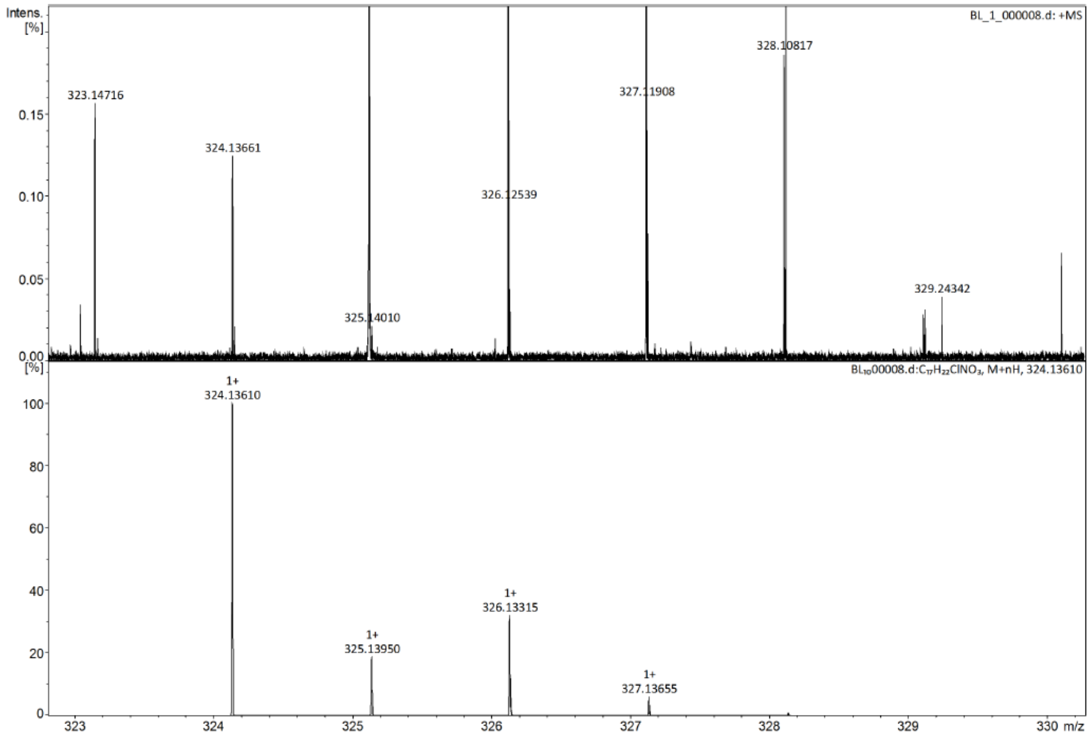

Bruker Compass DataAnalysis 5.0 printed: 11/12/2025 6:31:16 PM by: demo Page 1 of 1

HRMS spectra of intermediate 8/8’ or product 3/3’

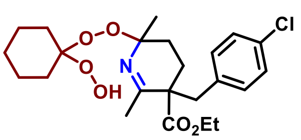

8/8'

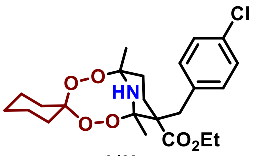

3/3'

Generic Display Report (all)

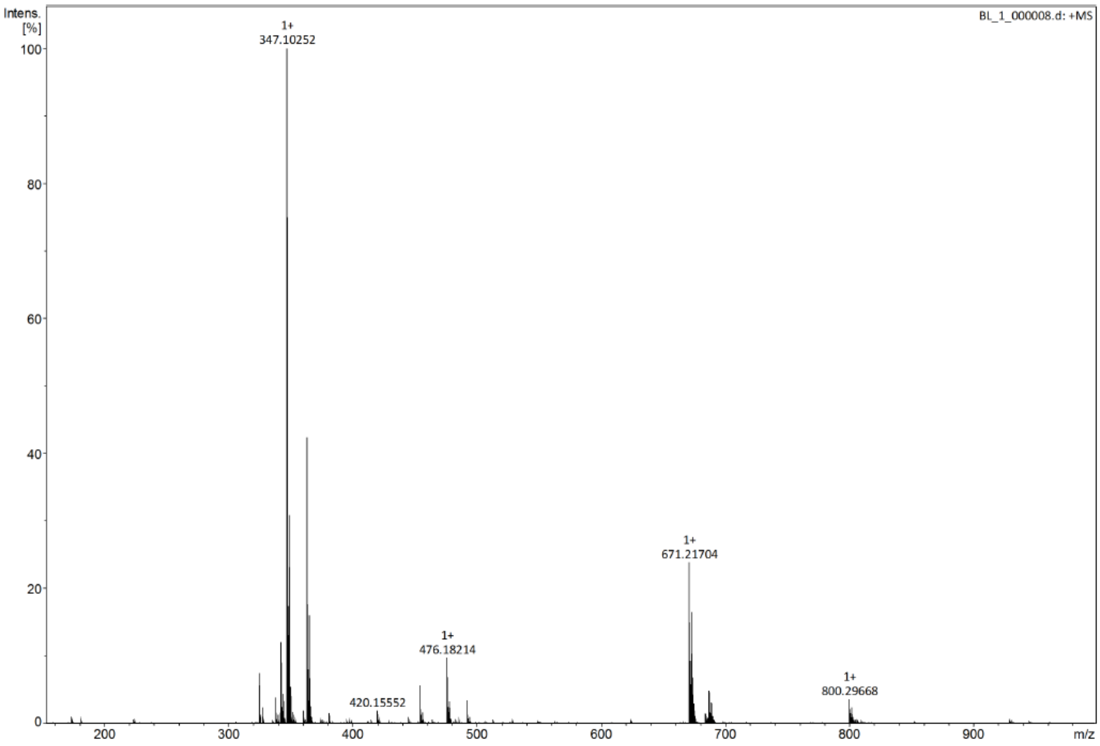

Bruker Compass DataAnalysis 5.0 printed: 11/12/2025 6:34:24 PM by: demo Page 1 of 1

Generic Display Report (all)

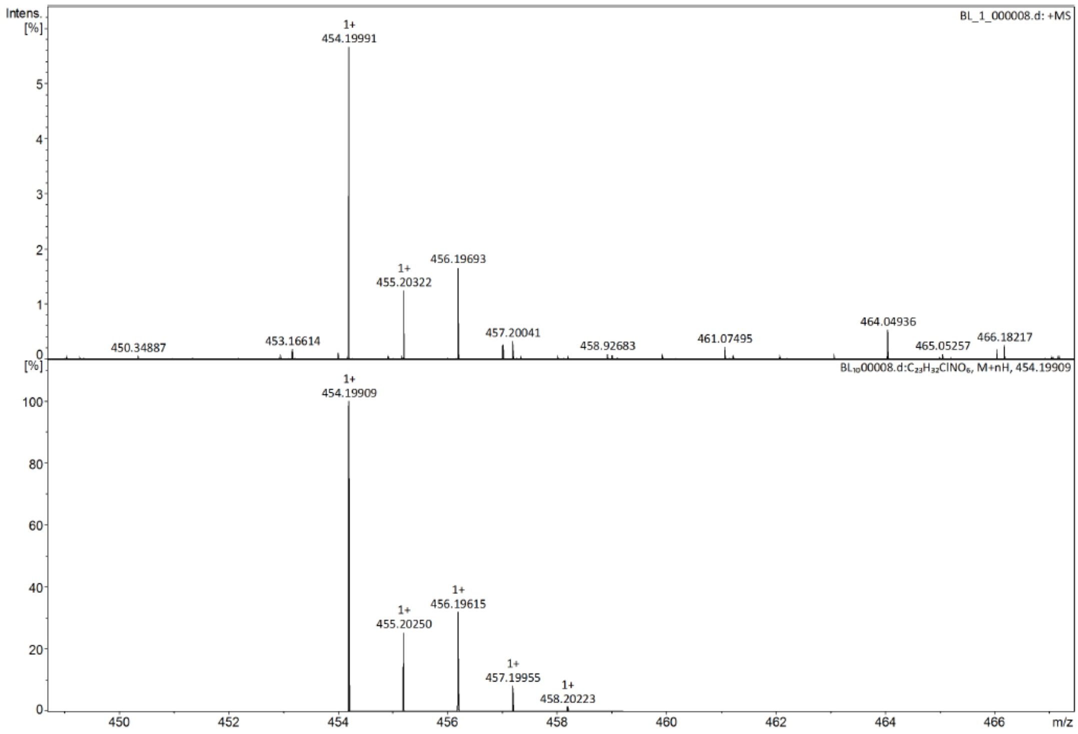

Bruker Compass DataAnalysis 5.0 printed: 11/12/2025 6:25:40 PM by: demo Page 1 of 1

X-Ray data of 3la, 3lb and 3lc

Crystal Growth Procedure.

Amino diperoxides **3la**, **3lb** or **3lc** (40 mg) were dissolved in a minimum amount of ethyl acetate, the vial was tightly closed with cotton wool, placed in a container with petroleum ether (20 ml), tightly closed with a cap and left at room temperature until the solvent evaporated and crystals formed.

X-ray crystallographic data and refinement details

X-ray diffraction data were collected at 100K on a four-circle Rigaku Synergy S diffractometer equipped with a HyPix6000HE area-detector (kappa geometry, shutterless ω-scan technique), using graphite monochromatized Cu K_{α}-radiation. The intensity data were integrated and corrected for absorption and decay by the CrysAlisPro program.[1] The structure was solved by direct methods using SHELXT[2] and refined on F^2 using SHELXL-2018[3] in the OLEX2 program. [4] All non-hydrogen atoms were refined with individual anisotropic displacement parameters. The location of hydrogen atom H11 was found from the electron density-difference map; this hydrogen atom was refined with an individual isotropic displacement parameter. All other hydrogen atoms were placed in ideal calculated positions and refined as riding atoms with relative isotropic displacement parameters. The Mercury program suite[5] was used for molecular graphics. A rotating group model was applied for methyl groups.

CCDC 2480403, 2480408 and 2480409 contains the supplementary crystallographic data for **3la**, **3lb** and **3lc**. These data can be obtained free of charge from The Cambridge Crystallographic Data Centre via https://www.ccdc.cam.ac.uk/structures.

X-ray crystallographic for 3la

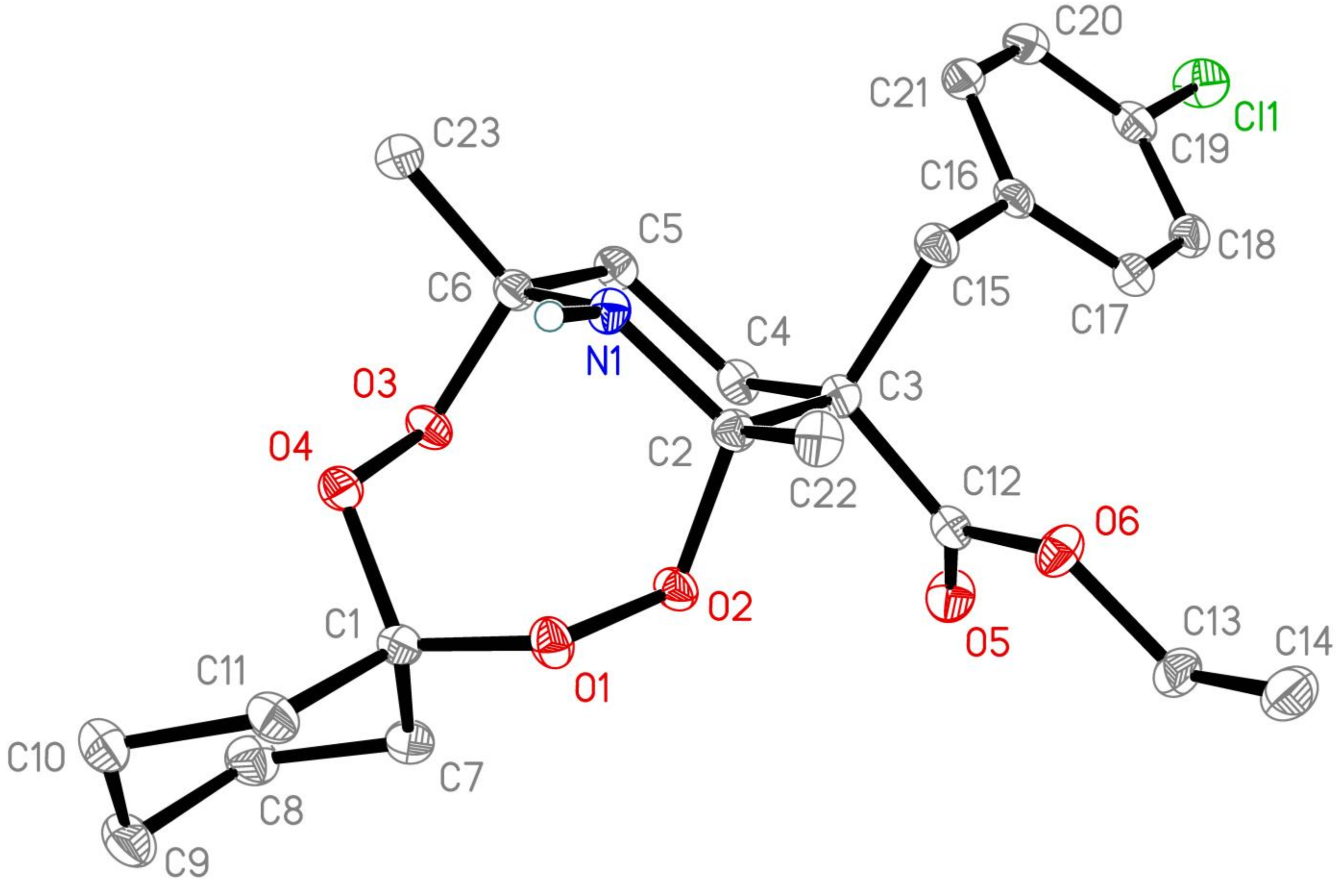

Figure S1. Molecular structure of **3la** presented in thermal ellipsoids (50% probability).

Table S1. Crystal data and structure refinement for **3la**.

Identification code	bl-1273	
Empirical formula	C23 H32 Cl N O6	
Formula weight	453.94	
Temperature	99.98(10) K	
Wavelength	1.54184 Å	
Crystal system	Monoclinic	
Space group	P 2_1/n	
Unit cell dimensions	a = 11.88540(5) Å	a= 90°.
	b = 7.38572(3) Å	b= 90.7407(4)°.
	c = 25.62494(10) Å	g = 90°.
Volume	2249.225(16) Å^3	
Z	4	
Density (calculated)	1.341 g/cm^3	
Absorption coefficient	1.836 mm^{-1}	
F(000)	968	
Crystal size	0.12 x 0.08 x 0.02 mm^3	
Theta range for data collection	3.450 to 79.584°.	

Index ranges	-15<=h<=15, -9<=k<=9, -32<=l<=32
Reflections collected	48378
Independent reflections	4885 [R(int) = 0.0226]
Observed reflections	4876
Completeness to theta = 67.684°	100.0 %
Absorption correction	Semi-empirical from equivalents
Max. and min. transmission	1.00000 and 0.85545
Refinement method	Full-matrix least-squares on F^2
Data / restraints / parameters	4885 / 0 / 287
Goodness-of-fit on F^2	1.067
Final R indices [I>2sigma(I)]	R1 = 0.0340, wR2 = 0.0862
R indices (all data)	R1 = 0.0340, wR2 = 0.0862
Largest diff. peak and hole	0.267 and -0.396 e.Å^{-3}

Table S2. Atomic coordinates (x 10^4) and equivalent isotropic displacement parameters (Å^{2}x 10^3)
for BL-1273. U(eq) is defined as one third of the trace of the orthogonalized U^{ij} tensor.

	x	y	z	U(eq)
Cl(1)	6661(1)	-569(1)	3970(1)	26(1)
O(1)	421(1)	9347(1)	3881(1)	15(1)
O(2)	1043(1)	7631(1)	3911(1)	14(1)
O(3)	1845(1)	8773(1)	2944(1)	14(1)
O(4)	1164(1)	10359(1)	3080(1)	15(1)
O(5)	1640(1)	3380(1)	4124(1)	20(1)
O(6)	1948(1)	5013(1)	4849(1)	17(1)
N(1)	2770(1)	9316(1)	3780(1)	13(1)
C(1)	186(1)	9768(2)	3346(1)	14(1)
C(2)	2180(1)	8056(1)	4103(1)	13(1)
C(3)	2747(1)	6144(1)	4066(1)	13(1)
C(4)	2836(1)	5627(2)	3485(1)	15(1)
C(5)	3432(1)	7072(2)	3165(1)	15(1)
C(6)	2916(1)	8941(2)	3235(1)	14(1)
C(7)	-428(1)	8246(2)	3062(1)	17(1)
C(8)	-828(1)	8868(2)	2521(1)	21(1)
C(9)	-1579(1)	10543(2)	2560(1)	24(1)
C(10)	-972(1)	12085(2)	2846(1)	21(1)
C(11)	-524(1)	11492(2)	3383(1)	18(1)
C(12)	2035(1)	4696(2)	4335(1)	14(1)

C(13)	1322(1)	3703(2)	5154(1)	19(1)
C(14)	1253(1)	4458(2)	5697(1)	24(1)
C(15)	3928(1)	6208(2)	4340(1)	15(1)
C(16)	4624(1)	4520(2)	4259(1)	15(1)
C(17)	4358(1)	2887(2)	4503(1)	17(1)
C(18)	4972(1)	1324(2)	4411(1)	18(1)
C(19)	5888(1)	1401(2)	4082(1)	17(1)
C(20)	6194(1)	3002(2)	3843(1)	19(1)
C(21)	5555(1)	4545(2)	3931(1)	18(1)
C(22)	2096(1)	8902(2)	4645(1)	16(1)
C(23)	3603(1)	10442(2)	2989(1)	18(1)

Table S3. Bond lengths [Å] and angles [°] for BL-1273.

Cl(1)-C(19)	1.7460(12)
O(1)-O(2)	1.4691(10)
O(1)-C(1)	1.4293(13)
O(2)-C(2)	1.4656(12)
O(3)-O(4)	1.4679(10)
O(3)-C(6)	1.4719(12)
O(4)-C(1)	1.4240(13)
O(5)-C(12)	1.2052(14)
O(6)-C(12)	1.3425(13)
O(6)-C(13)	1.4545(13)
N(1)-H(1)	0.850(17)
N(1)-C(2)	1.4338(14)
N(1)-C(6)	1.4362(13)
C(1)-C(7)	1.5210(15)
C(1)-C(11)	1.5304(15)
C(2)-C(3)	1.5685(14)
C(2)-C(22)	1.5272(14)
C(3)-C(4)	1.5421(14)
C(3)-C(12)	1.5338(15)
C(3)-C(15)	1.5621(14)
C(4)-H(4A)	0.9900
C(4)-H(4B)	0.9900
C(4)-C(5)	1.5246(15)
C(5)-H(5A)	0.9900
C(5)-H(5B)	0.9900
C(5)-C(6)	1.5221(15)
C(6)-C(23)	1.5189(15)

C(7)-H(7A)	0.9900
C(7)-H(7B)	0.9900
C(7)-C(8)	1.5286(16)
C(8)-H(8A)	0.9900
C(8)-H(8B)	0.9900
C(8)-C(9)	1.5296(17)
C(9)-H(9A)	0.9900
C(9)-H(9B)	0.9900
C(9)-C(10)	1.5294(18)
C(10)-H(10A)	0.9900
C(10)-H(10B)	0.9900
C(10)-C(11)	1.5328(16)
C(11)-H(11A)	0.9900
C(11)-H(11B)	0.9900
C(13)-H(13A)	0.9900
C(13)-H(13B)	0.9900
C(13)-C(14)	1.5030(16)
C(14)-H(14A)	0.9800
C(14)-H(14B)	0.9800
C(14)-H(14C)	0.9800
C(15)-H(15A)	0.9900
C(15)-H(15B)	0.9900
C(15)-C(16)	1.5120(15)
C(16)-C(17)	1.3966(15)
C(16)-C(21)	1.3984(16)
C(17)-H(17)	0.9500
C(17)-C(18)	1.3879(16)
C(18)-H(18)	0.9500
C(18)-C(19)	1.3872(16)
C(19)-C(20)	1.3830(16)
C(20)-H(20)	0.9500
C(20)-C(21)	1.3894(16)
C(21)-H(21)	0.9500
C(22)-H(22A)	0.9800
C(22)-H(22B)	0.9800
C(22)-H(22C)	0.9800
C(23)-H(23A)	0.9800
C(23)-H(23B)	0.9800
C(23)-H(23C)	0.9800
C(1)-O(1)-O(2)	109.27(7)
C(2)-O(2)-O(1)	107.14(7)

O(4)-O(3)-C(6)	106.75(7)
C(1)-O(4)-O(3)	108.93(7)
C(12)-O(6)-C(13)	117.19(9)
C(2)-N(1)-H(1)	111.6(11)
C(2)-N(1)-C(6)	120.14(9)
C(6)-N(1)-H(1)	111.0(11)
O(1)-C(1)-C(7)	112.62(9)
O(1)-C(1)-C(11)	102.86(8)
O(4)-C(1)-O(1)	112.07(8)
O(4)-C(1)-C(7)	112.76(9)
O(4)-C(1)-C(11)	103.23(9)
C(7)-C(1)-C(11)	112.50(9)
O(2)-C(2)-C(3)	100.47(8)
O(2)-C(2)-C(22)	108.77(8)
N(1)-C(2)-O(2)	113.61(8)
N(1)-C(2)-C(3)	109.63(8)
N(1)-C(2)-C(22)	107.31(9)
C(22)-C(2)-C(3)	117.17(9)
C(4)-C(3)-C(2)	108.43(8)
C(4)-C(3)-C(15)	111.65(9)
C(12)-C(3)-C(2)	111.15(8)
C(12)-C(3)-C(4)	107.95(8)
C(12)-C(3)-C(15)	108.44(8)
C(15)-C(3)-C(2)	109.24(8)
C(3)-C(4)-H(4A)	109.1
C(3)-C(4)-H(4B)	109.1
H(4A)-C(4)-H(4B)	107.8
C(5)-C(4)-C(3)	112.55(9)
C(5)-C(4)-H(4A)	109.1
C(5)-C(4)-H(4B)	109.1
C(4)-C(5)-H(5A)	109.1
C(4)-C(5)-H(5B)	109.1
H(5A)-C(5)-H(5B)	107.9
C(6)-C(5)-C(4)	112.40(9)
C(6)-C(5)-H(5A)	109.1
C(6)-C(5)-H(5B)	109.1
O(3)-C(6)-C(5)	102.17(8)
O(3)-C(6)-C(23)	108.53(8)
N(1)-C(6)-O(3)	113.21(8)
N(1)-C(6)-C(5)	110.06(9)
N(1)-C(6)-C(23)	109.62(9)

C(23)-C(6)-C(5)	113.15(9)
C(1)-C(7)-H(7A)	109.5
C(1)-C(7)-H(7B)	109.5
C(1)-C(7)-C(8)	110.69(10)
H(7A)-C(7)-H(7B)	108.1
C(8)-C(7)-H(7A)	109.5
C(8)-C(7)-H(7B)	109.5
C(7)-C(8)-H(8A)	109.4
C(7)-C(8)-H(8B)	109.4
C(7)-C(8)-C(9)	111.00(10)
H(8A)-C(8)-H(8B)	108.0
C(9)-C(8)-H(8A)	109.4
C(9)-C(8)-H(8B)	109.4
C(8)-C(9)-H(9A)	109.4
C(8)-C(9)-H(9B)	109.4
H(9A)-C(9)-H(9B)	108.0
C(10)-C(9)-C(8)	111.21(10)
C(10)-C(9)-H(9A)	109.4
C(10)-C(9)-H(9B)	109.4
C(9)-C(10)-H(10A)	109.2
C(9)-C(10)-H(10B)	109.2
C(9)-C(10)-C(11)	111.85(10)
H(10A)-C(10)-H(10B)	107.9
C(11)-C(10)-H(10A)	109.2
C(11)-C(10)-H(10B)	109.2
C(1)-C(11)-C(10)	111.60(9)
C(1)-C(11)-H(11A)	109.3
C(1)-C(11)-H(11B)	109.3
C(10)-C(11)-H(11A)	109.3
C(10)-C(11)-H(11B)	109.3
H(11A)-C(11)-H(11B)	108.0
O(5)-C(12)-O(6)	123.24(10)
O(5)-C(12)-C(3)	124.96(10)
O(6)-C(12)-C(3)	111.72(9)
O(6)-C(13)-H(13A)	110.4
O(6)-C(13)-H(13B)	110.4
O(6)-C(13)-C(14)	106.57(9)
H(13A)-C(13)-H(13B)	108.6
C(14)-C(13)-H(13A)	110.4
C(14)-C(13)-H(13B)	110.4
C(13)-C(14)-H(14A)	109.5

C(13)-C(14)-H(14B)	109.5
C(13)-C(14)-H(14C)	109.5
H(14A)-C(14)-H(14B)	109.5
H(14A)-C(14)-H(14C)	109.5
H(14B)-C(14)-H(14C)	109.5
C(3)-C(15)-H(15A)	108.8
C(3)-C(15)-H(15B)	108.8
H(15A)-C(15)-H(15B)	107.7
C(16)-C(15)-C(3)	113.83(9)
C(16)-C(15)-H(15A)	108.8
C(16)-C(15)-H(15B)	108.8
C(17)-C(16)-C(15)	121.53(10)
C(17)-C(16)-C(21)	117.78(10)
C(21)-C(16)-C(15)	120.68(10)
C(16)-C(17)-H(17)	119.3
C(18)-C(17)-C(16)	121.31(10)
C(18)-C(17)-H(17)	119.3
C(17)-C(18)-H(18)	120.4
C(19)-C(18)-C(17)	119.21(11)
C(19)-C(18)-H(18)	120.4
C(18)-C(19)-Cl(1)	118.96(9)
C(20)-C(19)-Cl(1)	119.89(9)
C(20)-C(19)-C(18)	121.15(11)
C(19)-C(20)-H(20)	120.6
C(19)-C(20)-C(21)	118.81(10)
C(21)-C(20)-H(20)	120.6
C(16)-C(21)-H(21)	119.1
C(20)-C(21)-C(16)	121.71(10)
C(20)-C(21)-H(21)	119.1
C(2)-C(22)-H(22A)	109.5
C(2)-C(22)-H(22B)	109.5
C(2)-C(22)-H(22C)	109.5
H(22A)-C(22)-H(22B)	109.5
H(22A)-C(22)-H(22C)	109.5
H(22B)-C(22)-H(22C)	109.5
C(6)-C(23)-H(23A)	109.5
C(6)-C(23)-H(23B)	109.5
C(6)-C(23)-H(23C)	109.5
H(23A)-C(23)-H(23B)	109.5
H(23A)-C(23)-H(23C)	109.5
H(23B)-C(23)-H(23C)	109.5

Table S4. Anisotropic displacement parameters ($Å^2$x 10^3) for BL-1273. The anisotropic displacement factor exponent takes the form: $-2p^2[h^2 a^{*2}U^{11} + ... + 2 h k a^* b^* U^{12}]$

	U^{11}	U^{22}	U^{33}	U^{23}	U^{13}	U^{12}
Cl(1)	23(1)	17(1)	38(1)	-2(1)	2(1)	5(1)
O(1)	16(1)	14(1)	15(1)	0(1)	0(1)	5(1)
O(2)	12(1)	11(1)	19(1)	1(1)	-2(1)	2(1)
O(3)	13(1)	14(1)	16(1)	-3(1)	-1(1)	4(1)
O(4)	13(1)	13(1)	18(1)	1(1)	1(1)	3(1)
O(5)	26(1)	15(1)	20(1)	-1(1)	-3(1)	-6(1)
O(6)	23(1)	15(1)	15(1)	0(1)	2(1)	-5(1)
N(1)	15(1)	11(1)	13(1)	-1(1)	0(1)	-1(1)
C(1)	12(1)	17(1)	15(1)	1(1)	0(1)	1(1)
C(2)	13(1)	13(1)	13(1)	-1(1)	-1(1)	-1(1)
C(3)	15(1)	11(1)	14(1)	-1(1)	-1(1)	0(1)
C(4)	16(1)	14(1)	13(1)	-2(1)	-2(1)	1(1)
C(5)	14(1)	17(1)	14(1)	-2(1)	0(1)	2(1)
C(6)	12(1)	16(1)	13(1)	0(1)	-1(1)	0(1)
C(7)	15(1)	18(1)	19(1)	-1(1)	-1(1)	-2(1)
C(8)	19(1)	26(1)	19(1)	-2(1)	-4(1)	-2(1)
C(9)	18(1)	31(1)	23(1)	3(1)	-5(1)	2(1)
C(10)	18(1)	22(1)	23(1)	4(1)	-1(1)	5(1)
C(11)	15(1)	18(1)	21(1)	-1(1)	0(1)	4(1)
C(12)	14(1)	14(1)	15(1)	0(1)	-3(1)	1(1)
C(13)	20(1)	17(1)	19(1)	3(1)	1(1)	-4(1)
C(14)	23(1)	32(1)	18(1)	2(1)	2(1)	-7(1)
C(15)	15(1)	13(1)	16(1)	-1(1)	-3(1)	-1(1)
C(16)	13(1)	15(1)	16(1)	0(1)	-5(1)	-1(1)
C(17)	15(1)	18(1)	16(1)	2(1)	-2(1)	-1(1)
C(18)	19(1)	16(1)	19(1)	3(1)	-4(1)	-2(1)
C(19)	16(1)	15(1)	21(1)	-2(1)	-5(1)	2(1)
C(20)	15(1)	21(1)	21(1)	1(1)	-1(1)	0(1)
C(21)	16(1)	16(1)	21(1)	3(1)	-2(1)	-2(1)
C(22)	20(1)	14(1)	14(1)	-2(1)	0(1)	0(1)
C(23)	16(1)	20(1)	19(1)	2(1)	0(1)	-2(1)

Table S5. Hydrogen coordinates (x 10^4) and isotropic displacement parameters (Å^{2}x 10 ^{3}) for BL-1273.

	x	y	z	U(eq)
H(1)	2541(13)	10390(20)	3826(6)	22(4)
H(4A)	3253	4472	3455	18
H(4B)	2071	5436	3338	18
H(5A)	4235	7115	3271	18
H(5B)	3393	6736	2791	18
H(7A)	-1083	7858	3268	21
H(7B)	82	7195	3025	21
H(8A)	-1253	7876	2349	25
H(8B)	-168	9152	2304	25
H(9A)	-1800	10948	2205	29
H(9B)	-2273	10227	2750	29
H(10A)	-1499	13111	2890	26
H(10B)	-337	12511	2632	26
H(11A)	-60	12476	3536	21
H(11B)	-1165	11272	3617	21
H(13A)	1713	2520	5158	22
H(13B)	558	3532	5003	22
H(14A)	913	5665	5684	37
H(14B)	2012	4541	5850	37
H(14C)	791	3658	5912	37
H(15A)	4348	7264	4205	18
H(15B)	3823	6391	4719	18
H(17)	3745	2846	4737	20
H(18)	4766	214	4571	21
H(20)	6830	3047	3622	23
H(21)	5756	5645	3764	21
H(22A)	1743	10098	4616	24
H(22B)	2851	9028	4798	24
H(22C)	1639	8123	4868	24
H(23A)	3713	10173	2619	27
H(23B)	4337	10525	3166	27
H(23C)	3203	11595	3023	27

Table S6. Torsion angles [°] for BL-1273.

Cl(1)-C(19)-C(20)-C(21)	178.95(8)
O(1)-O(2)-C(2)-N(1)	-58.42(10)
O(1)-O(2)-C(2)-C(3)	-175.40(7)
O(1)-O(2)-C(2)-C(22)	61.01(10)
O(1)-C(1)-C(7)-C(8)	170.80(9)
O(1)-C(1)-C(11)-C(10)	-174.33(9)
O(2)-O(1)-C(1)-O(4)	-73.50(10)
O(2)-O(1)-C(1)-C(7)	54.90(10)
O(2)-O(1)-C(1)-C(11)	176.26(7)
O(2)-C(2)-C(3)-C(4)	67.33(10)
O(2)-C(2)-C(3)-C(12)	-51.17(10)
O(2)-C(2)-C(3)-C(15)	-170.79(8)
O(3)-O(4)-C(1)-O(1)	78.81(10)
O(3)-O(4)-C(1)-C(7)	-49.51(10)
O(3)-O(4)-C(1)-C(11)	-171.18(7)
O(4)-O(3)-C(6)-N(1)	49.55(11)
O(4)-O(3)-C(6)-C(5)	167.85(7)
O(4)-O(3)-C(6)-C(23)	-72.39(10)
O(4)-C(1)-C(7)-C(8)	-61.16(12)
O(4)-C(1)-C(11)-C(10)	68.95(11)
N(1)-C(2)-C(3)-C(4)	-52.56(11)
N(1)-C(2)-C(3)-C(12)	-171.07(8)
N(1)-C(2)-C(3)-C(15)	69.32(11)
C(1)-O(1)-O(2)-C(2)	112.51(8)
C(1)-C(7)-C(8)-C(9)	-56.73(12)
C(2)-N(1)-C(6)-O(3)	60.91(13)
C(2)-N(1)-C(6)-C(5)	-52.71(12)
C(2)-N(1)-C(6)-C(23)	-177.77(9)
C(2)-C(3)-C(4)-C(5)	53.88(11)
C(2)-C(3)-C(12)-O(5)	119.92(12)
C(2)-C(3)-C(12)-O(6)	-63.37(11)
C(2)-C(3)-C(15)-C(16)	-171.42(9)
C(3)-C(4)-C(5)-C(6)	-52.74(12)
C(3)-C(15)-C(16)-C(17)	-71.75(13)
C(3)-C(15)-C(16)-C(21)	107.52(11)
C(4)-C(3)-C(12)-O(5)	1.13(15)
C(4)-C(3)-C(12)-O(6)	177.84(8)
C(4)-C(3)-C(15)-C(16)	-51.50(12)
C(4)-C(5)-C(6)-O(3)	-72.48(10)
C(4)-C(5)-C(6)-N(1)	48.04(11)

C(4)-C(5)-C(6)-C(23)	171.05(9)
C(6)-O(3)-O(4)-C(1)	-115.91(8)
C(6)-N(1)-C(2)-O(2)	-55.75(13)
C(6)-N(1)-C(2)-C(3)	55.75(12)
C(6)-N(1)-C(2)-C(22)	-176.01(9)
C(7)-C(1)-C(11)-C(10)	-52.89(12)
C(7)-C(8)-C(9)-C(10)	56.67(13)
C(8)-C(9)-C(10)-C(11)	-54.41(13)
C(9)-C(10)-C(11)-C(1)	52.20(13)
C(11)-C(1)-C(7)-C(8)	55.10(12)
C(12)-O(6)-C(13)-C(14)	-174.53(9)
C(12)-C(3)-C(4)-C(5)	174.39(8)
C(12)-C(3)-C(15)-C(16)	67.31(11)
C(13)-O(6)-C(12)-O(5)	-1.37(16)
C(13)-O(6)-C(12)-C(3)	-178.15(9)
C(15)-C(3)-C(4)-C(5)	-66.51(11)
C(15)-C(3)-C(12)-O(5)	-119.99(11)
C(15)-C(3)-C(12)-O(6)	56.72(11)
C(15)-C(16)-C(17)-C(18)	177.24(10)
C(15)-C(16)-C(21)-C(20)	-178.69(10)
C(16)-C(17)-C(18)-C(19)	2.00(17)
C(17)-C(16)-C(21)-C(20)	0.62(16)
C(17)-C(18)-C(19)-Cl(1)	179.65(8)
C(17)-C(18)-C(19)-C(20)	-0.47(17)
C(18)-C(19)-C(20)-C(21)	-0.93(17)
C(19)-C(20)-C(21)-C(16)	0.85(17)
C(21)-C(16)-C(17)-C(18)	-2.06(16)
C(22)-C(2)-C(3)-C(4)	-175.12(9)
C(22)-C(2)-C(3)-C(12)	66.38(11)
C(22)-C(2)-C(3)-C(15)	-53.24(12)

Table S7. Hydrogen bonds for BL-1273 [Å and °].

D-H...A	d(D-H)	d(H...A)	d(D...A)	<(DHA)
N(1)-H(1)...O(5)#1	0.850(17)	2.572(17)	3.4089(13)	168.4(14)

Symmetry transformations used to generate equivalent atoms:
#1 x,y+1,z

X-ray crystallographic for 3lb

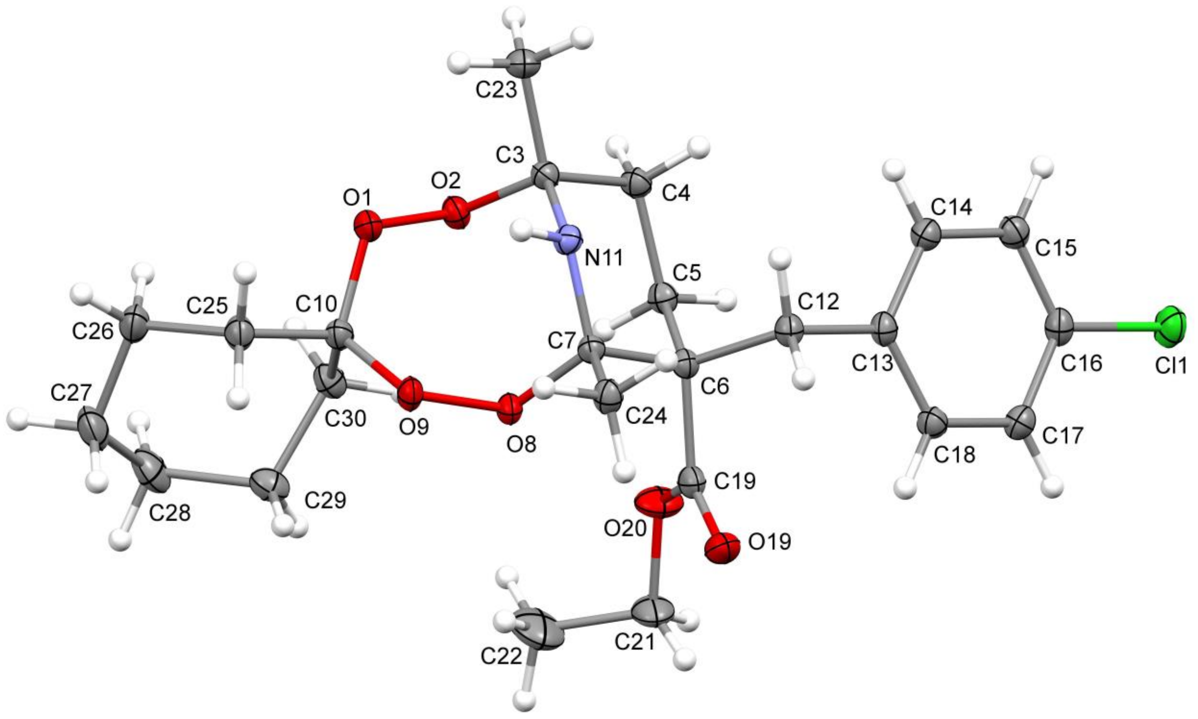

Figure S2. Molecular structure of **3lb** presented in thermal ellipsoids (50% probability).

Table S8. Crystal data and structure refinement for **3lb**.

Identification code	BL-1352	
Empirical formula	C24 H34 Cl N O6	
Formula weight	467.97	
Temperature	103(5) K	
Wavelength	1.54184 Å	
Crystal system	Monoclinic	
Space group	P 2_1/c	
Unit cell dimensions	a = 13.10198(9) Å	a= 90°.
	b = 14.61105(10) Å	b= 106.5782(8)°.
	c = 12.85876(9) Å	g = 90°.
Volume	2359.27(3) Å^3	
Z	4	
Density (calculated)	1.317 g/cm^3	
Absorption coefficient	1.766 mm^{-1}	
F(000)	1000	
Crystal size	0.48 x 0.29 x 0.06 mm^3	
Theta range for data collection	3.520 to 79.996°.	
Index ranges	-16<=h<=15, -18<=k<=18, -16<=l<=16	
Reflections collected	32732	
Independent reflections	5140 [R(int) = 0.0366]	

Observed reflections	4879
Completeness to theta = 67.684°	100.0 %
Absorption correction	Gaussian
Max. and min. transmission	1.000 and 0.348
Refinement method	Full-matrix least-squares on F^2
Data / restraints / parameters	5140 / 2 / 304
Goodness-of-fit on F^2	1.085
Final R indices [I>2sigma(I)]	R1 = 0.0343, wR2 = 0.0880
R indices (all data)	R1 = 0.0355, wR2 = 0.0888
Largest diff. peak and hole	0.279 and -0.278 e.Å^{-3}

Table S9. Atomic coordinates (x 10^4) and equivalent isotropic displacement parameters (Å^{2}x 10^3)
for BL-1352. U(eq) is defined as one third of the trace of the orthogonalized U^{ij} tensor.

	x	y	z	U(eq)
Cl(1)	-1400(1)	6414(1)	10057(1)	25(1)
O(1)	4044(1)	7767(1)	5380(1)	18(1)
O(2)	3646(1)	7884(1)	6331(1)	17(1)
O(8)	3084(1)	6049(1)	5845(1)	16(1)
O(9)	3617(1)	6207(1)	5001(1)	17(1)
O(19)	1445(1)	4635(1)	6468(1)	24(1)
O(20)	2723(1)	5280(1)	7810(1)	28(1)
N(11)	1951(1)	7358(1)	5154(1)	15(1)
C(3)	2497(1)	8042(1)	5912(1)	16(1)
C(4)	2132(1)	7979(1)	6934(1)	17(1)
C(5)	2219(1)	6999(1)	7363(1)	16(1)
C(6)	1628(1)	6293(1)	6510(1)	15(1)
C(7)	2000(1)	6413(1)	5461(1)	15(1)
C(10)	4459(1)	6860(1)	5388(1)	17(1)
C(12)	399(1)	6414(1)	6213(1)	16(1)
C(13)	-39(1)	6418(1)	7186(1)	16(1)
C(14)	-369(1)	7231(1)	7562(1)	18(1)
C(15)	-792(1)	7243(1)	8442(1)	19(1)
C(16)	-871(1)	6423(1)	8951(1)	18(1)
C(17)	-550(1)	5601(1)	8606(1)	18(1)
C(18)	-138(1)	5605(1)	7720(1)	17(1)
C(19)	1907(1)	5307(1)	6911(1)	18(1)
C(21)	3143(8)	4398(7)	8260(11)	31(1)

C(21A)	3241(8)	4393(7)	8108(11)	31(1)
C(22)	4209(3)	4368(3)	8058(3)	47(1)
C(22A)	4299(3)	4573(3)	8914(3)	47(1)
C(23)	2314(1)	8975(1)	5372(1)	20(1)
C(24)	1380(1)	5862(1)	4479(1)	18(1)
C(25)	4958(1)	6890(1)	4442(1)	20(1)
C(26)	6141(1)	7141(1)	4725(1)	25(1)
C(27)	6896(1)	6339(1)	5157(1)	30(1)
C(28)	6967(1)	6035(1)	6309(1)	30(1)
C(29)	5907(1)	5783(1)	6511(1)	24(1)
C(30)	5183(1)	6607(1)	6504(1)	19(1)

Table S10. Bond lengths [Å] and angles [°] for BL-1352.

Cl(1)-C(16)	1.7503(11)
O(1)-O(2)	1.4697(10)
O(1)-C(10)	1.4305(13)
O(2)-C(3)	1.4645(13)
O(8)-O(9)	1.4662(10)
O(8)-C(7)	1.4643(13)
O(9)-C(10)	1.4362(13)
O(19)-C(19)	1.2068(15)
O(20)-C(19)	1.3325(15)
O(20)-C(21)	1.456(9)
O(20)-C(21A)	1.463(9)
N(11)-H(11)	0.873(17)
N(11)-C(3)	1.4360(14)
N(11)-C(7)	1.4326(14)
C(3)-C(4)	1.5236(15)
C(3)-C(23)	1.5181(15)
C(4)-H(4A)	0.9900
C(4)-H(4B)	0.9900
C(4)-C(5)	1.5283(15)
C(5)-H(5A)	0.9900
C(5)-H(5B)	0.9900
C(5)-C(6)	1.5431(15)
C(6)-C(7)	1.5691(14)
C(6)-C(12)	1.5549(15)
C(6)-C(19)	1.5397(15)
C(7)-C(24)	1.5220(15)
C(10)-C(25)	1.5372(15)

C(10)-C(30)	1.5232(16)
C(12)-H(12A)	0.9900
C(12)-H(12B)	0.9900
C(12)-C(13)	1.5191(15)
C(13)-C(14)	1.3959(16)
C(13)-C(18)	1.3975(16)
C(14)-H(14)	0.9500
C(14)-C(15)	1.3940(16)
C(15)-H(15)	0.9500
C(15)-C(16)	1.3838(17)
C(16)-C(17)	1.3860(16)
C(17)-H(17)	0.9500
C(17)-C(18)	1.3923(16)
C(18)-H(18)	0.9500
C(21)-H(21A)	0.9900
C(21)-H(21B)	0.9900
C(21)-C(22)	1.492(11)
C(21A)-H(21C)	0.9900
C(21A)-H(21D)	0.9900
C(21A)-C(22A)	1.498(11)
C(22)-H(22A)	0.9800
C(22)-H(22B)	0.9800
C(22)-H(22C)	0.9800
C(22A)-H(22D)	0.9800
C(22A)-H(22E)	0.9800
C(22A)-H(22F)	0.9800
C(23)-H(23A)	0.9800
C(23)-H(23B)	0.9800
C(23)-H(23C)	0.9800
C(24)-H(24A)	0.9800
C(24)-H(24B)	0.9800
C(24)-H(24C)	0.9800
C(25)-H(25A)	0.9900
C(25)-H(25B)	0.9900
C(25)-C(26)	1.5327(16)
C(26)-H(26A)	0.9900
C(26)-H(26B)	0.9900
C(26)-C(27)	1.5315(19)
C(27)-H(27A)	0.9900
C(27)-H(27B)	0.9900
C(27)-C(28)	1.524(2)

C(28)-H(28A)	0.9900
C(28)-H(28B)	0.9900
C(28)-C(29)	1.5295(18)
C(29)-H(29A)	0.9900
C(29)-H(29B)	0.9900
C(29)-C(30)	1.5316(16)
C(30)-H(30A)	0.9900
C(30)-H(30B)	0.9900
C(10)-O(1)-O(2)	109.09(8)
C(3)-O(2)-O(1)	106.50(7)
C(7)-O(8)-O(9)	108.33(7)
C(10)-O(9)-O(8)	109.23(7)
C(19)-O(20)-C(21)	119.3(5)
C(19)-O(20)-C(21A)	116.8(5)
C(3)-N(11)-H(11)	110.8(11)
C(7)-N(11)-H(11)	111.1(11)
C(7)-N(11)-C(3)	120.58(9)
O(2)-C(3)-C(4)	102.49(8)
O(2)-C(3)-C(23)	108.73(9)
N(11)-C(3)-O(2)	113.68(9)
N(11)-C(3)-C(4)	109.01(9)
N(11)-C(3)-C(23)	109.33(9)
C(23)-C(3)-C(4)	113.56(9)
C(3)-C(4)-H(4A)	109.4
C(3)-C(4)-H(4B)	109.4
C(3)-C(4)-C(5)	110.98(9)
H(4A)-C(4)-H(4B)	108.0
C(5)-C(4)-H(4A)	109.4
C(5)-C(4)-H(4B)	109.4
C(4)-C(5)-H(5A)	108.9
C(4)-C(5)-H(5B)	108.9
C(4)-C(5)-C(6)	113.48(9)
H(5A)-C(5)-H(5B)	107.7
C(6)-C(5)-H(5A)	108.9
C(6)-C(5)-H(5B)	108.9
C(5)-C(6)-C(7)	108.73(9)
C(5)-C(6)-C(12)	111.93(9)
C(12)-C(6)-C(7)	109.26(9)
C(19)-C(6)-C(5)	111.34(9)
C(19)-C(6)-C(7)	106.89(9)
C(19)-C(6)-C(12)	108.55(9)

O(8)-C(7)-C(6)	100.30(8)
O(8)-C(7)-C(24)	108.96(9)
N(11)-C(7)-O(8)	113.92(9)
N(11)-C(7)-C(6)	110.11(9)
N(11)-C(7)-C(24)	108.08(9)
C(24)-C(7)-C(6)	115.50(9)
O(1)-C(10)-O(9)	111.15(9)
O(1)-C(10)-C(25)	102.46(9)
O(1)-C(10)-C(30)	111.36(9)
O(9)-C(10)-C(25)	101.71(9)
O(9)-C(10)-C(30)	111.98(9)
C(30)-C(10)-C(25)	117.48(9)
C(6)-C(12)-H(12A)	108.8
C(6)-C(12)-H(12B)	108.8
H(12A)-C(12)-H(12B)	107.6
C(13)-C(12)-C(6)	114.00(9)
C(13)-C(12)-H(12A)	108.8
C(13)-C(12)-H(12B)	108.8
C(14)-C(13)-C(12)	121.08(10)
C(14)-C(13)-C(18)	118.18(10)
C(18)-C(13)-C(12)	120.73(10)
C(13)-C(14)-H(14)	119.2
C(15)-C(14)-C(13)	121.69(11)
C(15)-C(14)-H(14)	119.2
C(14)-C(15)-H(15)	120.9
C(16)-C(15)-C(14)	118.29(11)
C(16)-C(15)-H(15)	120.9
C(15)-C(16)-Cl(1)	119.42(9)
C(15)-C(16)-C(17)	121.86(11)
C(17)-C(16)-Cl(1)	118.71(9)
C(16)-C(17)-H(17)	120.6
C(16)-C(17)-C(18)	118.84(11)
C(18)-C(17)-H(17)	120.6
C(13)-C(18)-H(18)	119.4
C(17)-C(18)-C(13)	121.12(11)
C(17)-C(18)-H(18)	119.4
O(19)-C(19)-O(20)	123.80(11)
O(19)-C(19)-C(6)	124.33(11)
O(20)-C(19)-C(6)	111.87(10)
O(20)-C(21)-H(21A)	111.2
O(20)-C(21)-H(21B)	111.2

O(20)-C(21)-C(22)	102.9(7)
H(21A)-C(21)-H(21B)	109.1
C(22)-C(21)-H(21A)	111.2
C(22)-C(21)-H(21B)	111.2
O(20)-C(21A)-H(21C)	110.3
O(20)-C(21A)-H(21D)	110.3
O(20)-C(21A)-C(22A)	107.1(7)
H(21C)-C(21A)-H(21D)	108.6
C(22A)-C(21A)-H(21C)	110.3
C(22A)-C(21A)-H(21D)	110.3
C(21)-C(22)-H(22A)	109.5
C(21)-C(22)-H(22B)	109.5
C(21)-C(22)-H(22C)	109.5
H(22A)-C(22)-H(22B)	109.5
H(22A)-C(22)-H(22C)	109.5
H(22B)-C(22)-H(22C)	109.5
C(21A)-C(22A)-H(22D)	109.5
C(21A)-C(22A)-H(22E)	109.5
C(21A)-C(22A)-H(22F)	109.5
H(22D)-C(22A)-H(22E)	109.5
H(22D)-C(22A)-H(22F)	109.5
H(22E)-C(22A)-H(22F)	109.5
C(3)-C(23)-H(23A)	109.5
C(3)-C(23)-H(23B)	109.5
C(3)-C(23)-H(23C)	109.5
H(23A)-C(23)-H(23B)	109.5
H(23A)-C(23)-H(23C)	109.5
H(23B)-C(23)-H(23C)	109.5
C(7)-C(24)-H(24A)	109.5
C(7)-C(24)-H(24B)	109.5
C(7)-C(24)-H(24C)	109.5
H(24A)-C(24)-H(24B)	109.5
H(24A)-C(24)-H(24C)	109.5
H(24B)-C(24)-H(24C)	109.5
C(10)-C(25)-H(25A)	108.1
C(10)-C(25)-H(25B)	108.1
H(25A)-C(25)-H(25B)	107.3
C(26)-C(25)-C(10)	116.65(10)
C(26)-C(25)-H(25A)	108.1
C(26)-C(25)-H(25B)	108.1
C(25)-C(26)-H(26A)	108.7

C(25)-C(26)-H(26B)	108.7
H(26A)-C(26)-H(26B)	107.6
C(27)-C(26)-C(25)	114.04(11)
C(27)-C(26)-H(26A)	108.7
C(27)-C(26)-H(26B)	108.7
C(26)-C(27)-H(27A)	108.4
C(26)-C(27)-H(27B)	108.4
H(27A)-C(27)-H(27B)	107.5
C(28)-C(27)-C(26)	115.48(11)
C(28)-C(27)-H(27A)	108.4
C(28)-C(27)-H(27B)	108.4
C(27)-C(28)-H(28A)	108.4
C(27)-C(28)-H(28B)	108.4
C(27)-C(28)-C(29)	115.41(11)
H(28A)-C(28)-H(28B)	107.5
C(29)-C(28)-H(28A)	108.4
C(29)-C(28)-H(28B)	108.4
C(28)-C(29)-H(29A)	108.8
C(28)-C(29)-H(29B)	108.8
C(28)-C(29)-C(30)	113.64(11)
H(29A)-C(29)-H(29B)	107.7
C(30)-C(29)-H(29A)	108.8
C(30)-C(29)-H(29B)	108.8
C(10)-C(30)-C(29)	114.51(10)
C(10)-C(30)-H(30A)	108.6
C(10)-C(30)-H(30B)	108.6
C(29)-C(30)-H(30A)	108.6
C(29)-C(30)-H(30B)	108.6
H(30A)-C(30)-H(30B)	107.6

Table S11. Anisotropic displacement parameters ($Å^2$x 10^3) for BL-1352. The anisotropic displacement factor exponent takes the form: $-2p^2[h^2 a^{*2}U^{11} + ... + 2 h k a^* b^* U^{12}]$

	U^{11}	U^{22}	U^{33}	U^{23}	U^{13}	U^{12}
Cl(1)	31(1)	27(1)	24(1)	-4(1)	18(1)	-4(1)
O(1)	18(1)	17(1)	22(1)	0(1)	10(1)	0(1)
O(2)	15(1)	19(1)	19(1)	-3(1)	6(1)	0(1)
O(8)	14(1)	18(1)	18(1)	1(1)	8(1)	1(1)
O(9)	15(1)	19(1)	18(1)	-3(1)	9(1)	-3(1)

O(19)	29(1)	16(1)	33(1)	-1(1)	15(1)	-2(1)
O(20)	32(1)	20(1)	29(1)	7(1)	1(1)	7(1)
N(11)	16(1)	15(1)	14(1)	1(1)	5(1)	0(1)
C(3)	14(1)	14(1)	20(1)	-1(1)	5(1)	1(1)
C(4)	17(1)	16(1)	20(1)	-3(1)	6(1)	0(1)
C(5)	16(1)	18(1)	15(1)	-1(1)	5(1)	0(1)
C(6)	15(1)	15(1)	15(1)	0(1)	6(1)	0(1)
C(7)	13(1)	15(1)	17(1)	-1(1)	5(1)	0(1)
C(10)	14(1)	16(1)	22(1)	-2(1)	6(1)	-1(1)
C(12)	15(1)	18(1)	16(1)	-1(1)	6(1)	-1(1)
C(13)	13(1)	20(1)	17(1)	-1(1)	5(1)	-2(1)
C(14)	17(1)	17(1)	21(1)	1(1)	6(1)	-1(1)
C(15)	17(1)	19(1)	22(1)	-4(1)	7(1)	-1(1)
C(16)	15(1)	24(1)	18(1)	-3(1)	7(1)	-3(1)
C(17)	17(1)	19(1)	20(1)	0(1)	6(1)	-3(1)
C(18)	16(1)	17(1)	20(1)	-2(1)	6(1)	-1(1)
C(19)	20(1)	18(1)	21(1)	2(1)	12(1)	2(1)
C(21)	35(2)	22(1)	39(3)	16(1)	15(2)	10(1)
C(21A)	35(2)	22(1)	39(3)	16(1)	15(2)	10(1)
C(22)	42(1)	40(1)	58(1)	18(1)	12(1)	24(1)
C(22A)	42(1)	40(1)	58(1)	18(1)	12(1)	24(1)
C(23)	22(1)	14(1)	26(1)	2(1)	9(1)	2(1)
C(24)	17(1)	20(1)	18(1)	-4(1)	7(1)	-3(1)
C(25)	17(1)	23(1)	23(1)	-2(1)	10(1)	-1(1)
C(26)	20(1)	24(1)	34(1)	-3(1)	14(1)	-4(1)
C(27)	18(1)	35(1)	41(1)	-5(1)	14(1)	3(1)
C(28)	16(1)	36(1)	35(1)	-5(1)	4(1)	7(1)
C(29)	22(1)	22(1)	25(1)	0(1)	4(1)	5(1)
C(30)	16(1)	22(1)	20(1)	-3(1)	4(1)	1(1)

Table S12. Hydrogen coordinates (x 10^4) and isotropic displacement parameters ($Å^2$x 10^3) for BL-1352.

	x	y	z	U(eq)
H(11)	2080(13)	7427(12)	4528(14)	26(4)
H(4A)	1384	8188	6769	21
H(4B)	2576	8388	7498	21
H(5A)	1926	6974	7992	19

H(5B)	2981	6827	7623	19
H(12A)	204	6997	5813	19
H(12B)	57	5911	5720	19
H(14)	-305	7790	7209	22
H(15)	-1020	7800	8685	23
H(17)	-611	5045	8967	22
H(18)	80	5044	7475	21
H(21A)	2684	3890	7883	38
H(21B)	3209	4365	9045	38
H(21C)	3340	4083	7459	38
H(21D)	2801	3996	8432	38
H(22A)	4119	4381	7275	71
H(22B)	4578	3804	8367	71
H(22C)	4629	4898	8401	71
H(22D)	4732	4956	8579	71
H(22E)	4665	3991	9145	71
H(22F)	4191	4891	9546	71
H(23A)	2632	9448	5906	30
H(23B)	1547	9086	5085	30
H(23C)	2643	8993	4778	30
H(24A)	1699	5949	3884	27
H(24B)	638	6070	4251	27
H(24C)	1403	5211	4671	27
H(25A)	4863	6281	4089	24
H(25B)	4554	7336	3900	24
H(26A)	6294	7387	4067	30
H(26B)	6289	7633	5277	30
H(27A)	6663	5810	4665	36
H(27B)	7618	6513	5129	36
H(28A)	7299	6534	6813	36
H(28B)	7446	5498	6487	36
H(29A)	6053	5472	7222	28
H(29B)	5526	5344	5947	28
H(30A)	4734	6474	6989	23
H(30B)	5633	7142	6806	23

Table S13. Torsion angles [°] for BL-1352.

Cl(1)-C(16)-C(17)-C(18)	179.43(9)
O(1)-O(2)-C(3)-N(11)	52.58(11)

O(1)-O(2)-C(3)-C(4)	170.05(8)
O(1)-O(2)-C(3)-C(23)	-69.45(10)
O(1)-C(10)-C(25)-C(26)	92.57(12)
O(1)-C(10)-C(30)-C(29)	-162.99(9)
O(2)-O(1)-C(10)-O(9)	78.71(10)
O(2)-O(1)-C(10)-C(25)	-173.33(8)
O(2)-O(1)-C(10)-C(30)	-46.90(11)
O(2)-C(3)-C(4)-C(5)	-69.44(10)
O(8)-O(9)-C(10)-O(1)	-74.93(10)
O(8)-O(9)-C(10)-C(25)	176.63(8)
O(8)-O(9)-C(10)-C(30)	50.33(11)
O(9)-O(8)-C(7)-N(11)	-55.67(11)
O(9)-O(8)-C(7)-C(6)	-173.25(7)
O(9)-O(8)-C(7)-C(24)	65.08(10)
O(9)-C(10)-C(25)-C(26)	-152.39(10)
O(9)-C(10)-C(30)-C(29)	71.86(12)
N(11)-C(3)-C(4)-C(5)	51.32(12)
C(3)-N(11)-C(7)-O(8)	-56.81(13)
C(3)-N(11)-C(7)-C(6)	54.95(12)
C(3)-N(11)-C(7)-C(24)	-178.05(9)
C(3)-C(4)-C(5)-C(6)	-54.39(12)
C(4)-C(5)-C(6)-C(7)	52.09(12)
C(4)-C(5)-C(6)-C(12)	-68.70(11)
C(4)-C(5)-C(6)-C(19)	169.60(9)
C(5)-C(6)-C(7)-O(8)	71.42(10)
C(5)-C(6)-C(7)-N(11)	-48.93(11)
C(5)-C(6)-C(7)-C(24)	-171.67(9)
C(5)-C(6)-C(12)-C(13)	-53.03(12)
C(5)-C(6)-C(19)-O(19)	169.26(10)
C(5)-C(6)-C(19)-O(20)	-10.81(13)
C(6)-C(12)-C(13)-C(14)	105.02(12)
C(6)-C(12)-C(13)-C(18)	-75.97(13)
C(7)-O(8)-O(9)-C(10)	112.78(9)
C(7)-N(11)-C(3)-O(2)	58.07(13)
C(7)-N(11)-C(3)-C(4)	-55.56(12)
C(7)-N(11)-C(3)-C(23)	179.77(9)
C(7)-C(6)-C(12)-C(13)	-173.51(9)
C(7)-C(6)-C(19)-O(19)	-72.12(13)
C(7)-C(6)-C(19)-O(20)	107.80(10)
C(10)-O(1)-O(2)-C(3)	-116.40(9)
C(10)-C(25)-C(26)-C(27)	81.92(14)

C(12)-C(6)-C(7)-O(8)	-166.16(8)
C(12)-C(6)-C(7)-N(11)	73.49(11)
C(12)-C(6)-C(7)-C(24)	-49.25(12)
C(12)-C(6)-C(19)-O(19)	45.62(14)
C(12)-C(6)-C(19)-O(20)	-134.46(10)
C(12)-C(13)-C(14)-C(15)	178.79(10)
C(12)-C(13)-C(18)-C(17)	-179.28(10)
C(13)-C(14)-C(15)-C(16)	0.58(17)
C(14)-C(13)-C(18)-C(17)	-0.24(17)
C(14)-C(15)-C(16)-Cl(1)	-179.90(9)
C(14)-C(15)-C(16)-C(17)	-0.42(17)
C(15)-C(16)-C(17)-C(18)	-0.04(18)
C(16)-C(17)-C(18)-C(13)	0.38(17)
C(18)-C(13)-C(14)-C(15)	-0.25(17)
C(19)-O(20)-C(21)-C(22)	110.7(6)
C(19)-O(20)-C(21A)-C(22A)	163.4(5)
C(19)-C(6)-C(7)-O(8)	-48.88(10)
C(19)-C(6)-C(7)-N(11)	-169.24(9)
C(19)-C(6)-C(7)-C(24)	68.02(12)
C(19)-C(6)-C(12)-C(13)	70.27(11)
C(21)-O(20)-C(19)-O(19)	4.3(6)
C(21)-O(20)-C(19)-C(6)	-175.7(6)
C(21A)-O(20)-C(19)-O(19)	15.6(6)
C(21A)-O(20)-C(19)-C(6)	-164.3(5)
C(23)-C(3)-C(4)-C(5)	173.46(9)
C(25)-C(10)-C(30)-C(29)	-45.32(14)
C(25)-C(26)-C(27)-C(28)	-73.28(15)
C(26)-C(27)-C(28)-C(29)	55.45(17)
C(27)-C(28)-C(29)-C(30)	-70.70(15)
C(28)-C(29)-C(30)-C(10)	87.67(13)
C(30)-C(10)-C(25)-C(26)	-29.79(15)

X-ray crystallographic for 3lc

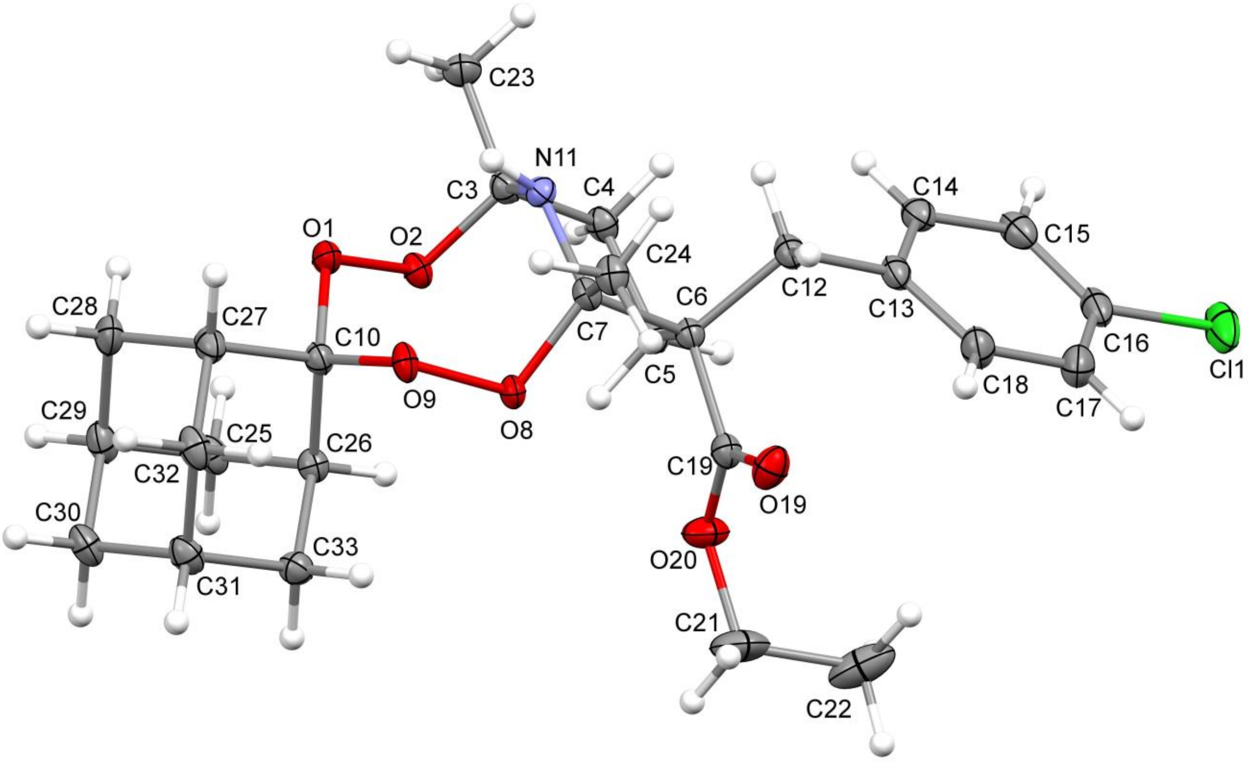

Figure S3. Molecular structure of **3lc** presented in thermal ellipsoids (50% probability).

Table S14. Crystal data and structure refinement for **3lc**.

Identification code	BL-1518	
Empirical formula	C27 H36 Cl N O6	
Formula weight	506.02	
Temperature	100.0(2) K	
Wavelength	1.54184 Å	
Crystal system	Monoclinic	
Space group	P 2_1/c	
Unit cell dimensions	a = 11.09277(10) Å	a= 90°.
	b = 11.50522(9) Å	b= 105.3317(9)°.
	c = 20.47109(18) Å	g = 90°.
Volume	2519.64(4) $Å^3$	
Z	4	
Density (calculated)	1.334 g/cm^3	
Absorption coefficient	1.697 mm^{-1}	
F(000)	1080	
Crystal size	0.25 x 0.11 x 0.09 mm^3	
Theta range for data collection	4.132 to 79.777°.	
Index ranges	-13<=h<=14, -14<=k<=14, -25<=l<=26	
Reflections collected	32047	

Independent reflections	5471 [R(int) = 0.0339]
Observed reflections	5139
Completeness to theta = 67.684°	100.0 %
Absorption correction	Gaussian
Max. and min. transmission	1.000 and 0.560
Refinement method	Full-matrix least-squares on F^2
Data / restraints / parameters	5471 / 0 / 323
Goodness-of-fit on F^2	1.088
Final R indices [I>2sigma(I)]	R1 = 0.0345, wR2 = 0.0873
R indices (all data)	R1 = 0.0363, wR2 = 0.0883
Largest diff. peak and hole	0.323 and -0.245 e.Å^{-3}

Table S15. Atomic coordinates (x 10^4) and equivalent isotropic displacement parameters (Å^{2}x 10^3)
for BL-1518. U(eq) is defined as one third of the trace of the orthogonalized U^{ij} tensor.

	x	y	z	U(eq)
Cl(1)	8408(1)	9077(1)	5754(1)	33(1)
O(1)	7132(1)	1991(1)	2089(1)	17(1)
O(2)	6714(1)	2651(1)	2602(1)	16(1)
O(19)	8832(1)	7076(1)	2536(1)	23(1)
O(8)	7764(1)	4468(1)	2029(1)	15(1)
O(9)	7982(1)	3522(1)	1595(1)	16(1)
O(20)	6814(1)	6742(1)	2470(1)	25(1)
N(11)	8866(1)	3311(1)	2996(1)	15(1)
C(3)	7809(1)	2829(1)	3178(1)	16(1)
C(4)	7326(1)	3710(1)	3604(1)	16(1)
C(5)	7122(1)	4907(1)	3272(1)	16(1)
C(6)	8297(1)	5361(1)	3088(1)	14(1)
C(7)	8763(1)	4415(1)	2661(1)	14(1)
C(10)	6990(1)	2701(1)	1504(1)	15(1)
C(12)	9384(1)	5623(1)	3726(1)	17(1)
C(13)	9102(1)	6485(1)	4224(1)	18(1)
C(14)	8737(1)	6119(1)	4793(1)	20(1)
C(15)	8531(1)	6908(1)	5267(1)	23(1)
C(16)	8670(1)	8083(1)	5164(1)	23(1)
C(17)	9034(1)	8480(1)	4606(1)	27(1)
C(18)	9259(1)	7676(1)	4143(1)	23(1)
C(19)	8028(1)	6482(1)	2666(1)	17(1)

C(21)	6477(2)	7808(1)	2080(1)	36(1)
C(22)	6578(2)	8842(1)	2542(1)	40(1)
C(23)	8160(1)	1674(1)	3541(1)	21(1)
C(24)	10004(1)	4677(1)	2501(1)	18(1)
C(25)	4710(1)	2270(1)	1127(1)	19(1)
C(26)	5689(1)	3249(1)	1259(1)	15(1)
C(27)	7228(1)	1871(1)	963(1)	19(1)
C(28)	6238(1)	901(1)	827(1)	22(1)
C(29)	4930(1)	1441(1)	582(1)	21(1)
C(30)	4841(1)	2116(1)	-78(1)	22(1)
C(31)	5829(1)	3086(1)	55(1)	20(1)
C(32)	7139(1)	2554(1)	306(1)	21(1)
C(33)	5598(1)	3914(1)	598(1)	18(1)

Table S16. Bond lengths [Å] and angles [°] for BL-1518.

Cl(1)-C(16)	1.7422(12)
O(1)-O(2)	1.4670(11)
O(1)-C(10)	1.4235(13)
O(2)-C(3)	1.4672(13)
O(19)-C(19)	1.2088(15)
O(8)-O(9)	1.4655(10)
O(8)-C(7)	1.4648(13)
O(9)-C(10)	1.4248(13)
O(20)-C(19)	1.3339(15)
O(20)-C(21)	1.4579(16)
N(11)-H(11)	0.879(18)
N(11)-C(3)	1.4329(14)
N(11)-C(7)	1.4342(14)
C(3)-C(4)	1.5229(15)
C(3)-C(23)	1.5215(16)
C(4)-H(4A)	0.9900
C(4)-H(4B)	0.9900
C(4)-C(5)	1.5255(16)
C(5)-H(5A)	0.9900
C(5)-H(5B)	0.9900
C(5)-C(6)	1.5408(15)
C(6)-C(7)	1.5681(15)
C(6)-C(12)	1.5540(15)
C(6)-C(19)	1.5365(16)
C(7)-C(24)	1.5254(15)

C(10)-C(26)	1.5319(15)
C(10)-C(27)	1.5374(15)
C(12)-H(12A)	0.9900
C(12)-H(12B)	0.9900
C(12)-C(13)	1.5141(15)
C(13)-C(14)	1.3956(17)
C(13)-C(18)	1.3958(17)
C(14)-H(14)	0.9500
C(14)-C(15)	1.3899(17)
C(15)-H(15)	0.9500
C(15)-C(16)	1.384(2)
C(16)-C(17)	1.386(2)
C(17)-H(17)	0.9500
C(17)-C(18)	1.3928(18)
C(18)-H(18)	0.9500
C(21)-H(21A)	0.9900
C(21)-H(21B)	0.9900
C(21)-C(22)	1.504(2)
C(22)-H(22A)	0.9800
C(22)-H(22B)	0.9800
C(22)-H(22C)	0.9800
C(23)-H(23A)	0.9800
C(23)-H(23B)	0.9800
C(23)-H(23C)	0.9800
C(24)-H(24A)	0.9800
C(24)-H(24B)	0.9800
C(24)-H(24C)	0.9800
C(25)-H(25A)	0.9900
C(25)-H(25B)	0.9900
C(25)-C(26)	1.5384(15)
C(25)-C(29)	1.5368(16)
C(26)-H(26)	1.0000
C(26)-C(33)	1.5351(15)
C(27)-H(27)	1.0000
C(27)-C(28)	1.5386(17)
C(27)-C(32)	1.5367(17)
C(28)-H(28A)	0.9900
C(28)-H(28B)	0.9900
C(28)-C(29)	1.5346(18)
C(29)-H(29)	1.0000
C(29)-C(30)	1.5381(17)

C(30)-H(30A)	0.9900
C(30)-H(30B)	0.9900
C(30)-C(31)	1.5370(17)
C(31)-H(31)	1.0000
C(31)-C(32)	1.5348(17)
C(31)-C(33)	1.5362(16)
C(32)-H(32A)	0.9900
C(32)-H(32B)	0.9900
C(33)-H(33A)	0.9900
C(33)-H(33B)	0.9900
C(10)-O(1)-O(2)	108.60(7)
O(1)-O(2)-C(3)	107.24(7)
C(7)-O(8)-O(9)	107.22(7)
C(10)-O(9)-O(8)	109.00(7)
C(19)-O(20)-C(21)	116.49(10)
C(3)-N(11)-H(11)	112.3(11)
C(3)-N(11)-C(7)	120.04(9)
C(7)-N(11)-H(11)	112.2(11)
O(2)-C(3)-C(4)	102.18(9)
O(2)-C(3)-C(23)	108.73(9)
N(11)-C(3)-O(2)	113.93(9)
N(11)-C(3)-C(4)	109.06(9)
N(11)-C(3)-C(23)	110.11(9)
C(23)-C(3)-C(4)	112.68(9)
C(3)-C(4)-H(4A)	109.1
C(3)-C(4)-H(4B)	109.1
C(3)-C(4)-C(5)	112.30(9)
H(4A)-C(4)-H(4B)	107.9
C(5)-C(4)-H(4A)	109.1
C(5)-C(4)-H(4B)	109.1
C(4)-C(5)-H(5A)	109.1
C(4)-C(5)-H(5B)	109.1
C(4)-C(5)-C(6)	112.29(9)
H(5A)-C(5)-H(5B)	107.9
C(6)-C(5)-H(5A)	109.1
C(6)-C(5)-H(5B)	109.1
C(5)-C(6)-C(7)	109.00(9)
C(5)-C(6)-C(12)	112.29(9)
C(12)-C(6)-C(7)	107.85(9)
C(19)-C(6)-C(5)	111.77(9)
C(19)-C(6)-C(7)	108.19(9)

C(19)-C(6)-C(12)	107.59(9)
O(8)-C(7)-C(6)	100.33(8)
O(8)-C(7)-C(24)	108.57(9)
N(11)-C(7)-O(8)	113.92(9)
N(11)-C(7)-C(6)	110.08(9)
N(11)-C(7)-C(24)	108.37(9)
C(24)-C(7)-C(6)	115.57(9)
O(1)-C(10)-O(9)	111.12(9)
O(1)-C(10)-C(26)	113.39(9)
O(1)-C(10)-C(27)	104.35(9)
O(9)-C(10)-C(26)	113.48(9)
O(9)-C(10)-C(27)	103.42(9)
C(26)-C(10)-C(27)	110.21(9)
C(6)-C(12)-H(12A)	108.3
C(6)-C(12)-H(12B)	108.3
H(12A)-C(12)-H(12B)	107.4
C(13)-C(12)-C(6)	116.14(9)
C(13)-C(12)-H(12A)	108.3
C(13)-C(12)-H(12B)	108.3
C(14)-C(13)-C(12)	121.45(11)
C(14)-C(13)-C(18)	117.93(11)
C(18)-C(13)-C(12)	120.55(11)
C(13)-C(14)-H(14)	119.2
C(15)-C(14)-C(13)	121.54(12)
C(15)-C(14)-H(14)	119.2
C(14)-C(15)-H(15)	120.5
C(16)-C(15)-C(14)	119.02(12)
C(16)-C(15)-H(15)	120.5
C(15)-C(16)-Cl(1)	119.30(10)
C(15)-C(16)-C(17)	121.10(11)
C(17)-C(16)-Cl(1)	119.59(10)
C(16)-C(17)-H(17)	120.5
C(16)-C(17)-C(18)	119.04(12)
C(18)-C(17)-H(17)	120.5
C(13)-C(18)-H(18)	119.3
C(17)-C(18)-C(13)	121.34(12)
C(17)-C(18)-H(18)	119.3
O(19)-C(19)-O(20)	123.50(11)
O(19)-C(19)-C(6)	123.66(11)
O(20)-C(19)-C(6)	112.82(10)
O(20)-C(21)-H(21A)	109.5

O(20)-C(21)-H(21B)	109.5
O(20)-C(21)-C(22)	110.78(12)
H(21A)-C(21)-H(21B)	108.1
C(22)-C(21)-H(21A)	109.5
C(22)-C(21)-H(21B)	109.5
C(21)-C(22)-H(22A)	109.5
C(21)-C(22)-H(22B)	109.5
C(21)-C(22)-H(22C)	109.5
H(22A)-C(22)-H(22B)	109.5
H(22A)-C(22)-H(22C)	109.5
H(22B)-C(22)-H(22C)	109.5
C(3)-C(23)-H(23A)	109.5
C(3)-C(23)-H(23B)	109.5
C(3)-C(23)-H(23C)	109.5
H(23A)-C(23)-H(23B)	109.5
H(23A)-C(23)-H(23C)	109.5
H(23B)-C(23)-H(23C)	109.5
C(7)-C(24)-H(24A)	109.5
C(7)-C(24)-H(24B)	109.5
C(7)-C(24)-H(24C)	109.5
H(24A)-C(24)-H(24B)	109.5
H(24A)-C(24)-H(24C)	109.5
H(24B)-C(24)-H(24C)	109.5
H(25A)-C(25)-H(25B)	108.2
C(26)-C(25)-H(25A)	109.7
C(26)-C(25)-H(25B)	109.7
C(29)-C(25)-H(25A)	109.7
C(29)-C(25)-H(25B)	109.7
C(29)-C(25)-C(26)	109.93(9)
C(10)-C(26)-C(25)	108.48(9)
C(10)-C(26)-H(26)	109.9
C(10)-C(26)-C(33)	109.33(9)
C(25)-C(26)-H(26)	109.9
C(33)-C(26)-C(25)	109.34(9)
C(33)-C(26)-H(26)	109.9
C(10)-C(27)-H(27)	109.6
C(10)-C(27)-C(28)	109.31(10)
C(28)-C(27)-H(27)	109.6
C(32)-C(27)-C(10)	109.22(10)
C(32)-C(27)-H(27)	109.6
C(32)-C(27)-C(28)	109.57(10)

C(27)-C(28)-H(28A)	109.8
C(27)-C(28)-H(28B)	109.8
H(28A)-C(28)-H(28B)	108.2
C(29)-C(28)-C(27)	109.46(10)
C(29)-C(28)-H(28A)	109.8
C(29)-C(28)-H(28B)	109.8
C(25)-C(29)-H(29)	109.5
C(25)-C(29)-C(30)	109.80(10)
C(28)-C(29)-C(25)	109.39(9)
C(28)-C(29)-H(29)	109.5
C(28)-C(29)-C(30)	109.08(10)
C(30)-C(29)-H(29)	109.5
C(29)-C(30)-H(30A)	109.8
C(29)-C(30)-H(30B)	109.8
H(30A)-C(30)-H(30B)	108.2
C(31)-C(30)-C(29)	109.55(9)
C(31)-C(30)-H(30A)	109.8
C(31)-C(30)-H(30B)	109.8
C(30)-C(31)-H(31)	109.6
C(32)-C(31)-C(30)	109.67(10)
C(32)-C(31)-H(31)	109.6
C(32)-C(31)-C(33)	109.20(9)
C(33)-C(31)-C(30)	109.30(10)
C(33)-C(31)-H(31)	109.6
C(27)-C(32)-H(32A)	109.8
C(27)-C(32)-H(32B)	109.8
C(31)-C(32)-C(27)	109.50(9)
C(31)-C(32)-H(32A)	109.8
C(31)-C(32)-H(32B)	109.8
H(32A)-C(32)-H(32B)	108.2
C(26)-C(33)-C(31)	110.13(9)
C(26)-C(33)-H(33A)	109.6
C(26)-C(33)-H(33B)	109.6
C(31)-C(33)-H(33A)	109.6
C(31)-C(33)-H(33B)	109.6
H(33A)-C(33)-H(33B)	108.1

Table S17. Anisotropic displacement parameters ($Å^2 x 10^3$) for BL-1518. The anisotropic displacement factor exponent takes the form: $-2p^2[h^2 a^{*2}U^{11} + ... + 2 h k a^* b^* U^{12}]$

	U^{11}	U^{22}	U^{33}	U^{23}	U^{13}	U^{12}
Cl(1)	29(1)	38(1)	30(1)	-19(1)	3(1)	9(1)
O(1)	24(1)	15(1)	13(1)	-2(1)	4(1)	1(1)
O(2)	17(1)	19(1)	12(1)	-2(1)	4(1)	-1(1)
O(19)	28(1)	17(1)	27(1)	0(1)	12(1)	-4(1)
O(8)	17(1)	14(1)	12(1)	-4(1)	2(1)	0(1)
O(9)	16(1)	19(1)	15(1)	-7(1)	6(1)	-3(1)
O(20)	20(1)	23(1)	29(1)	9(1)	2(1)	4(1)
N(11)	15(1)	15(1)	15(1)	-1(1)	4(1)	1(1)
C(3)	17(1)	17(1)	12(1)	0(1)	2(1)	-2(1)
C(4)	18(1)	19(1)	13(1)	-1(1)	5(1)	-3(1)
C(5)	14(1)	17(1)	15(1)	-2(1)	4(1)	0(1)
C(6)	14(1)	15(1)	13(1)	-2(1)	3(1)	-1(1)
C(7)	14(1)	16(1)	12(1)	-2(1)	2(1)	-1(1)
C(10)	17(1)	16(1)	12(1)	-2(1)	3(1)	-2(1)
C(12)	15(1)	20(1)	16(1)	-4(1)	2(1)	0(1)
C(13)	14(1)	21(1)	16(1)	-5(1)	0(1)	0(1)
C(14)	18(1)	22(1)	18(1)	-2(1)	1(1)	3(1)
C(15)	19(1)	31(1)	16(1)	-4(1)	2(1)	4(1)
C(16)	18(1)	28(1)	20(1)	-10(1)	-2(1)	6(1)
C(17)	29(1)	21(1)	27(1)	-7(1)	2(1)	-1(1)
C(18)	26(1)	22(1)	20(1)	-4(1)	4(1)	-4(1)
C(19)	20(1)	16(1)	15(1)	-3(1)	4(1)	0(1)
C(21)	34(1)	32(1)	39(1)	19(1)	4(1)	10(1)
C(22)	47(1)	28(1)	54(1)	19(1)	33(1)	16(1)
C(23)	25(1)	18(1)	18(1)	3(1)	4(1)	0(1)
C(24)	16(1)	20(1)	18(1)	-4(1)	7(1)	-2(1)
C(25)	17(1)	22(1)	16(1)	-2(1)	4(1)	-5(1)
C(26)	15(1)	16(1)	14(1)	-1(1)	4(1)	-1(1)
C(27)	18(1)	20(1)	16(1)	-6(1)	2(1)	2(1)
C(28)	31(1)	17(1)	18(1)	-6(1)	4(1)	-1(1)
C(29)	23(1)	22(1)	17(1)	-4(1)	4(1)	-8(1)
C(30)	21(1)	28(1)	14(1)	-4(1)	1(1)	-4(1)
C(31)	22(1)	25(1)	13(1)	0(1)	4(1)	-3(1)
C(32)	20(1)	27(1)	16(1)	-6(1)	6(1)	-3(1)
C(33)	19(1)	19(1)	16(1)	1(1)	3(1)	-1(1)

Table S18. Hydrogen coordinates (x 10^4) and isotropic displacement parameters ($Å^2$x 10 ^{3})

for BL-1518.

	x	y	z	U(eq)
H(11)	9256(16)	2794(15)	2811(8)	26(4)
H(4A)	7934	3777	4053	20
H(4B)	6527	3427	3674	20
H(5A)	6882	5462	3585	19
H(5B)	6423	4863	2856	19
H(12A)	9646	4882	3968	21
H(12B)	10103	5919	3574	21
H(14)	8626	5313	4858	24
H(15)	8298	6643	5656	27
H(17)	9129	9288	4540	32
H(18)	9524	7943	3765	28
H(21A)	5610	7745	1791	44
H(21B)	7038	7918	1781	44
H(22A)	6301	9542	2270	59
H(22B)	7449	8939	2804	59
H(22C)	6049	8719	2851	59
H(23A)	7428	1346	3657	31
H(23B)	8830	1798	3956	31
H(23C)	8448	1135	3244	31
H(24A)	10149	4106	2175	27
H(24B)	10683	4634	2919	27
H(24C)	9975	5458	2308	27
H(25A)	4769	1834	1552	22
H(25B)	3861	2608	975	22
H(26)	5542	3791	1612	18
H(27)	8080	1522	1127	23
H(28A)	6384	363	478	27
H(28B)	6301	452	1247	27
H(29)	4286	811	496	25
H(30A)	4982	1580	-429	26
H(30B)	3997	2457	-244	26
H(31)	5769	3527	-374	24
H(32A)	7776	3178	392	25
H(32B)	7301	2028	-43	25
H(33A)	6224	4547	680	22
H(33B)	4757	4267	436	22

Table S19. Torsion angles [°] for BL-1518.

Cl(1)-C(16)-C(17)-C(18)	179.04(10)
O(1)-O(2)-C(3)-N(11)	51.68(11)
O(1)-O(2)-C(3)-C(4)	169.16(8)
O(1)-O(2)-C(3)-C(23)	-71.52(10)
O(1)-C(10)-C(26)-C(25)	-56.36(11)
O(1)-C(10)-C(26)-C(33)	-175.52(9)
O(1)-C(10)-C(27)-C(28)	61.87(11)
O(1)-C(10)-C(27)-C(32)	-178.26(9)
O(2)-O(1)-C(10)-O(9)	78.79(10)
O(2)-O(1)-C(10)-C(26)	-50.44(11)
O(2)-O(1)-C(10)-C(27)	-170.38(8)
O(2)-C(3)-C(4)-C(5)	-70.20(11)
O(8)-O(9)-C(10)-O(1)	-76.77(10)
O(8)-O(9)-C(10)-C(26)	52.41(11)
O(8)-O(9)-C(10)-C(27)	171.81(8)
O(9)-O(8)-C(7)-N(11)	-55.71(10)
O(9)-O(8)-C(7)-C(6)	-173.26(7)
O(9)-O(8)-C(7)-C(24)	65.13(10)
O(9)-C(10)-C(26)-C(25)	175.62(9)
O(9)-C(10)-C(26)-C(33)	56.46(12)
O(9)-C(10)-C(27)-C(28)	178.20(9)
O(9)-C(10)-C(27)-C(32)	-61.94(11)
N(11)-C(3)-C(4)-C(5)	50.70(12)
C(3)-N(11)-C(7)-O(8)	-56.35(13)
C(3)-N(11)-C(7)-C(6)	55.43(12)
C(3)-N(11)-C(7)-C(24)	-177.31(9)
C(3)-C(4)-C(5)-C(6)	-53.80(12)
C(4)-C(5)-C(6)-C(7)	52.42(12)
C(4)-C(5)-C(6)-C(12)	-67.01(12)
C(4)-C(5)-C(6)-C(19)	171.96(9)
C(5)-C(6)-C(7)-O(8)	69.79(10)
C(5)-C(6)-C(7)-N(11)	-50.57(11)
C(5)-C(6)-C(7)-C(24)	-173.73(9)
C(5)-C(6)-C(12)-C(13)	-57.41(13)
C(5)-C(6)-C(19)-O(19)	170.36(10)
C(5)-C(6)-C(19)-O(20)	-8.37(13)
C(6)-C(12)-C(13)-C(14)	96.08(13)
C(6)-C(12)-C(13)-C(18)	-86.96(14)
C(7)-O(8)-O(9)-C(10)	114.19(9)
C(7)-N(11)-C(3)-O(2)	58.85(13)

C(7)-N(11)-C(3)-C(4)	-54.58(12)
C(7)-N(11)-C(3)-C(23)	-178.70(9)
C(7)-C(6)-C(12)-C(13)	-177.51(9)
C(7)-C(6)-C(19)-O(19)	-69.62(13)
C(7)-C(6)-C(19)-O(20)	111.64(10)
C(10)-O(1)-O(2)-C(3)	-115.26(9)
C(10)-C(26)-C(33)-C(31)	59.10(12)
C(10)-C(27)-C(28)-C(29)	59.39(12)
C(10)-C(27)-C(32)-C(31)	-60.15(12)
C(12)-C(6)-C(7)-O(8)	-168.06(8)
C(12)-C(6)-C(7)-N(11)	71.58(11)
C(12)-C(6)-C(7)-C(24)	-51.57(12)
C(12)-C(6)-C(19)-O(19)	46.65(14)
C(12)-C(6)-C(19)-O(20)	-132.09(10)
C(12)-C(13)-C(14)-C(15)	177.14(11)
C(12)-C(13)-C(18)-C(17)	-178.39(11)
C(13)-C(14)-C(15)-C(16)	1.22(18)
C(14)-C(13)-C(18)-C(17)	-1.33(18)
C(14)-C(15)-C(16)-Cl(1)	179.77(9)
C(14)-C(15)-C(16)-C(17)	-1.36(18)
C(15)-C(16)-C(17)-C(18)	0.17(19)
C(16)-C(17)-C(18)-C(13)	1.20(19)
C(18)-C(13)-C(14)-C(15)	0.10(17)
C(19)-O(20)-C(21)-C(22)	-85.13(15)
C(19)-C(6)-C(7)-O(8)	-51.95(10)
C(19)-C(6)-C(7)-N(11)	-172.31(9)
C(19)-C(6)-C(7)-C(24)	64.54(12)
C(19)-C(6)-C(12)-C(13)	65.99(12)
C(21)-O(20)-C(19)-O(19)	-0.10(18)
C(21)-O(20)-C(19)-C(6)	178.64(11)
C(23)-C(3)-C(4)-C(5)	173.30(9)
C(25)-C(26)-C(33)-C(31)	-59.53(12)
C(25)-C(29)-C(30)-C(31)	59.54(13)
C(26)-C(10)-C(27)-C(28)	-60.18(12)
C(26)-C(10)-C(27)-C(32)	59.68(12)
C(26)-C(25)-C(29)-C(28)	60.46(12)
C(26)-C(25)-C(29)-C(30)	-59.21(12)
C(27)-C(10)-C(26)-C(25)	60.18(11)
C(27)-C(10)-C(26)-C(33)	-58.98(12)
C(27)-C(28)-C(29)-C(25)	-59.66(12)
C(27)-C(28)-C(29)-C(30)	60.45(12)

C(28)-C(27)-C(32)-C(31)	59.55(12)
C(28)-C(29)-C(30)-C(31)	-60.32(13)
C(29)-C(25)-C(26)-C(10)	-60.23(12)
C(29)-C(25)-C(26)-C(33)	58.92(12)
C(29)-C(30)-C(31)-C(32)	59.96(13)
C(29)-C(30)-C(31)-C(33)	-59.72(13)
C(30)-C(31)-C(32)-C(27)	-59.44(12)
C(30)-C(31)-C(33)-C(26)	60.06(12)
C(32)-C(27)-C(28)-C(29)	-60.26(12)
C(32)-C(31)-C(33)-C(26)	-59.91(12)
C(33)-C(31)-C(32)-C(27)	60.30(12)

Table S20. Hydrogen bonds for BL-1518 [Å and °].

D-H...A	d(D-H)	d(H...A)	d(D...A)	<(DHA)
N(11)-H(11)...O(19)#1	0.879(18)	2.545(18)	3.3453(13)	151.8(15)

Symmetry transformations used to generate equivalent atoms:
#1 -x+2,y-1/2,-z+1/2

References

[1] *Rigaku Oxford Diffraction* **2021**.
[2] G. Sheldrick, *Acta Crystallographica Section A* **2015**, *71*, 3-8.
[3] G. Sheldrick, *Acta Crystallographica Section C* **2015**, *71*, 3-8.
[4] O. V. Dolomanov, L. J. Bourhis, R. J. Gildea, J. A. K. Howard, H. Puschmann, *J. Appl. Cryst.* **2009**, *42*, 339-341.
[5] C. F. Macrae, I. Sovago, S. J. Cottrell, P. T. A. Galek, P. McCabe, E. Pidcock, M. Platings, G. P. Shields, J. S. Stevens, M. Towler, P. A. Wood, *J. Appl. Cryst.* **2020**, *53*, 226-235.
